# Genomic and Transcriptomic Evidence Supports Methane Metabolism in *Archaeoglobi*

**DOI:** 10.1128/mSystems.00651-19

**Published:** 2020-03-17

**Authors:** Yi-Fan Liu, Jing Chen, Livia S. Zaramela, Li-Ying Wang, Serge Maurice Mbadinga, Zhao-Wei Hou, Xiao-Lin Wu, Ji-Dong Gu, Karsten Zengler, Bo-Zhong Mu

**Affiliations:** a State Key Laboratory of Bioreactor Engineering and School of Chemistry and Molecular Engineering, East China University of Science and Technology, Shanghai, People’s Republic of China; b School of Biological Sciences, The University of Hong Kong, Hong Kong, People’s Republic of China; c Department of Pediatrics, University of California San Diego, San Diego, California, USA; d Engineering Research Center of MEOR, East China University of Science and Technology, Shanghai, People’s Republic of China; e Shanghai Institute of Pollution Control and Ecological Security, Shanghai, People’s Republic of China; f Exploration and Development Research Institute of Daqing Oilfield Company Limited, PetroChina, Daqing, Heilongjiang, People’s Republic of China; University of Tennessee at Knoxville

**Keywords:** horizontal gene transfer, HGT, metatranscriptomics, methyl-coenzyme M reductase complex, MCR complex, oil reservoir, methanogens

## Abstract

Euryarchaeal lineages have been believed to have a methanogenic last common ancestor. However, members of euryarchaeal *Archaeoglobi* have long been considered nonmethanogenic and their evolutionary history remains elusive. Here, three high-quality metagenomic-assembled genomes (MAGs) retrieved from high-temperature oil reservoir and hot springs, together with three newly assembled *Archaeoglobi* MAGs from previously reported hot spring metagenomes, are demonstrated to represent a novel genus of *Archaeoglobaceae*, “*Candidatus* Methanomixophus.” All “*Ca*. Methanomixophus” MAGs encode an M methyltransferase (MTR) complex and a traditional type of methyl-coenzyme M reductase (MCR) complex, which is different from the divergent MCR complexes found in “*Ca*. Polytropus marinifundus.” In addition, “*Ca*. Methanomixophus dualitatem” MAGs preserve the genomic capacity for dissimilatory sulfate reduction. Comparative phylogenetic analysis supports a laterally transferred origin for an MCR complex and vertical heritage of the MTR complex in this lineage. Metatranscriptomic analysis revealed concomitant *in situ* activity of hydrogen-dependent methylotrophic methanogenesis and heterotrophic fermentation within populations of “*Ca*. Methanomixophus hydrogenotrophicum” in a high-temperature oil reservoir.

**IMPORTANCE** Current understanding of the diversity, biology, and ecology of *Archaea* is very limited, especially considering how few of the known phyla have been cultured or genomically explored. The reconstruction of “*Ca*. Methanomixophus” MAGs not only expands the known range of metabolic versatility of the members of *Archaeoglobi* but also suggests that the phylogenetic distribution of MCR and MTR complexes is even wider than previously anticipated.

## Abstract (precis)

Current understanding of the diversity, biology, and ecology of *Archaea* is very limited, especially considering how few of the known phyla have been cultured or genomically explored. The reconstruction of “*Ca*. Methanomixophus” MAGs not only expands the known range of metabolic versatility of the members of *Archaeoglobi* but also suggests that the phylogenetic distribution of MCR and MTR complexes is even wider than previously anticipated.

## INTRODUCTION

Methanogenic lifestyles were originally inferred to be restricted to a few “traditional” *Euryarchaeota* that were distributed within seven orders, including *Methanopyrales*, *Methanococcales*, *Methanobacteriales*, *Methanosarcinales*, *Methanocellales*, *Methanomicrobiales*, and *Methanomassiliicoccales* (1, 2). The recent discovery of Methanonatronarchaeia, a novel extreme halophilic methanogen which branches basally to *Haloarchaea*, provides new insights into the evolutionary transition from an anaerobic and methanogenic ancestor to haloarchaeal lineages (3, 4). In addition, culture-independent approaches revealed a growing number of archaeal groups outside the *Euryarchaeota* (“*Ca*. Bathyarchaeota,” “*Ca*. Verstraetearchaeota,” and “*Ca*. Helarchaeota”) whose genomes contain the key genes for methanogenesis (5–9), suggesting the the evolutionary origin of methanogenesis predates the *Euryarchaeota*.

As key enzymes in archaeal methane cycling, methyl-coenzyme M reductase (MCR) complexes can be divided into two main clusters, with one cluster being composed of MCR complexes from traditional euryarchaeal lineages and “*Ca*. Verstraetearchaeota.” MCR complexes in this cluster catalyze the final step of methanogenesis or the initial step in anaerobic methane oxidation (1). On the other hand, the MCR homologs found in “*Ca*. Bathyarchaeota” formed another deep divergent branch, together with a newly found MCR complex which catalyzed short-chain alkane (butane and, probably, propane) in a euryarchaeal lineage, “*Ca*. Syntrophoarchaeum” (10). The close association of MCR complexes and the shared metabolic features of the “*Ca*. Bathyarchaeota” and “*Ca*. Syntrophoarchaeum” suggest that the MCR complex in “*Ca*. Bathyarchaeota” may catalyze short-chain hydrocarbon oxidation rather than methane production (10).

Despite being phylogenetically close to methanogenic *Euryarchaeota*, members of *Archaeoglobi* have long been considered nonmethanogenic archaea (11, 12). Genes conserved in both hydrogenotrophic methanogenesis and archaeal type Wood-Ljungdahl (WL) pathway are present in *Archaeoglobi* genomes, suggesting the remnants of its ancestral methane-cycling lifestyle (13). For a long time, however, genes encoding methyl coenzyme M reductase complex (MCR) and a complete N 5-methyl-H_4_M(S)PT:coenzyme M methyltransferase (MTR) complex had not been found in representatives of *Archaeoglobi*, which are hypothesized to have been lost during evolution after receiving *dsrAB* genes from the bacterial members via horizontal gene transfer (HGT) (13, 14). The MTR complex, encoded by the *mtr* operon, catalyzes the energy-conserving (sodium-pumping) methyl transfer from H_4_M(S)PT to CoM (15), which is one of the key enzymes in hydrogenotrophic methanogenesis (16). Recently, Boyd et al. found two divergent MCR complexes in a metagenome-assembled genome (MAG) representing a basal member of the class *Archaeoglobi*, “*Ca*. Polytropus marinifundus,” which could utilize nitrate, iron, and sulfur compounds as electron acceptors (17). However, genes coding for MTR complex were largely missing in “*Ca*. Polytropus marinifundus” except for *mtrH*, ruling out the possibility of conserving energy from hydrogenotrophic methanogenesis (17). Further analysis revealed that the two divergent MCRs were most likely received from “*Ca*. Syntrophoarchaeum” and “*Ca*. Bathyarchaeota” via HGT, which suggests their potential role in hydrocarbon activation (17). Hence, data explaining what the lowest common ancestor (LCA) of *Archaeoglobi* looks like and how the evolution transition occurs is still elusive.

Here, we assembled three *Archaeoglobi* MAGs and collected another three newly assembled *Archaeoglobi* MAGs from a previous study (7). All these MAGs were retrieved from samples from subsurface hydrothermal environments, such as hot springs and oil reservoir, indicating an anoxic and thermophilic life style of this new lineage. The comparative genomic analysis of these six MAGs expanded the current knowledge about the evolution trend of *Archaeoglobi* members (17).

## RESULTS AND DISCUSSION

### Discovery of a novel *Archaeoglobaceae* genus, “*Ca*. Methanomixophus.”

**FIG 1:**
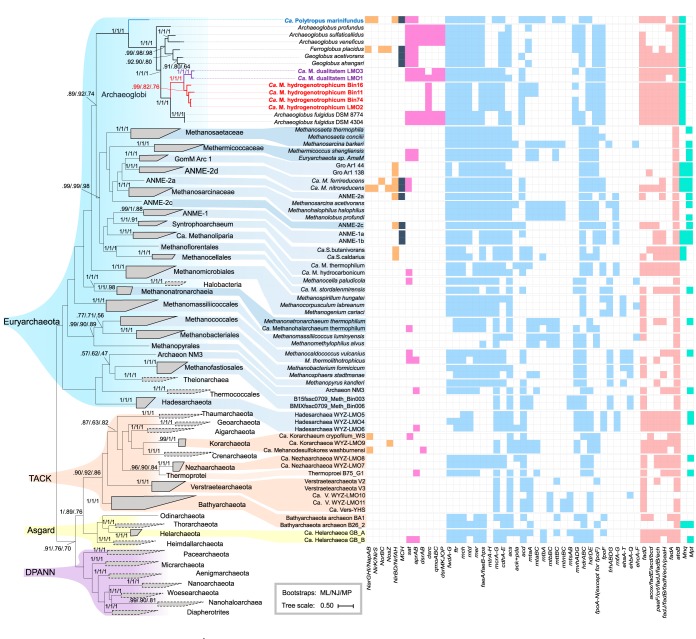
Phylogenomic tree showing the placement of “*Ca*. Methanomixophus” and the distribution of studied genes. The tree was inferred from the concatenation of 400 conserved marker genes using PhyloPhlAn (65), and only lineages containing MCR complex-bearing genomes for gene distribution are exhibited, whereas the lineages without MCR complex detected so far are outlined with dashed lines. Colored and blank squares represent the presence and absence of annotated genes, respectively. Genes associated with nitrate reduction and sulfate reduction are marked in brown and red, respectively; genes for iron reduction are marked in dark blue; genes involved in methanogenesis are marked in light blue; genes for beta-oxidation are marked in pink; genes associated with biosynthesis are marked in green. A functional complex consisting of multiple subunits was considered present if ≥75% of the genes comprising the complex were detected. Branches that represent “*Ca*. Methanomixophus” are marked in red, and the clade that represents “*Ca*. Polytropus marinifundus” is marked in blue. Abbreviations are as follows: *nar*, nitrate reductase/nitrite oxidoreductase; *nap*, periplasmic nitrate reductase; *nir*, nitrite reductase (NO-forming); *nor*, nitric oxide reductase; *sat*, sulfate adenylyltransferase; *apr*, adenylylsulfate reductase; *dsr*, dissimilatory sulfite reductase; *qmo*, quinone-modifying oxidoreductase; *dsrMKJOP*, membrane-bound heterodisulfide reductase; *fwd*, formylmethanofuran dehydrogenase; ftr, formylmethanofuran–tetrahydromethanopterin N-formyltransferase; *mer*, 5,10-methylenetetrahydromethanopterin reductase; *mtd*, methylenetetrahydromethanopterin dehydrogenase; *mch*, methenyltetrahydromethanopterin cyclohydrolase; *mcr*, methyl-coenzyme M reductase alpha subunit; *mtr*, methyl-H_4_M(S)PT:coenzyme M methyltransferase; *fae*, 5,6,7,8-tetrahydromethanopterin hydrolyase; *acs*, acetyl-CoA synthetase (EC 6.2.1.1); *pka*, phosphate acetyltransferase; *ack*, acetate kinase; *acd*, acetate-CoA ligase (ADP-forming) (EC 6.2.1.13); *cdh*, acetyl-CoA decarbonylase/synthase; *mta*, methanol-5-hydroxybenzimidazolylcobamide comethyltransferase; *mtm*, methylamine-corrinoid protein comethyltransferase; *mtb*, dimethylamine-corrinoid protein comethyltransferase; *mtt*, trimethylamine-corrinoid protein comethyltransferas; *mts*, methylthiol:coenzyme M methyltransferase; *mvh*, F_420_-nonreducing hydrogenase; *hdr*, heterodisulfide reductase; *fqo*, NADH-quinone-oxidoreductase; *frh*, coenzyme F_420_ hydrogenase; *eha*/*ehb*/*ehc*, energy-converting hydrogenase A/B/C; *rnf*, Na^+^-translocating ferredoxin:NAD^+^ oxidoreductase; *fadD*, long-chain acyl-CoA synthetase; *acox*/*fadE*/*acd*/*bcd*, acyl-ACP dehydrogenase; *paaF*/*crt*/*fadJ*/*fadB*/*ech*, enoyl-CoA hydratase; *fadJ*/*fadB*/*fadN*/*ech*/*paaH*, 3-hydroxyacyl-CoA dehydrogenase; MCH, multiheme c-type cytochromes; *fadA*, acetyl-CoA acyltransferase; *atoB*, acetyl-CoA C-acetyltransferase; Mnq, genes for biosynthesis of the menaquinone; Mpt, genes for biosynthesis of the methanophenazine.

**FIG 2:**
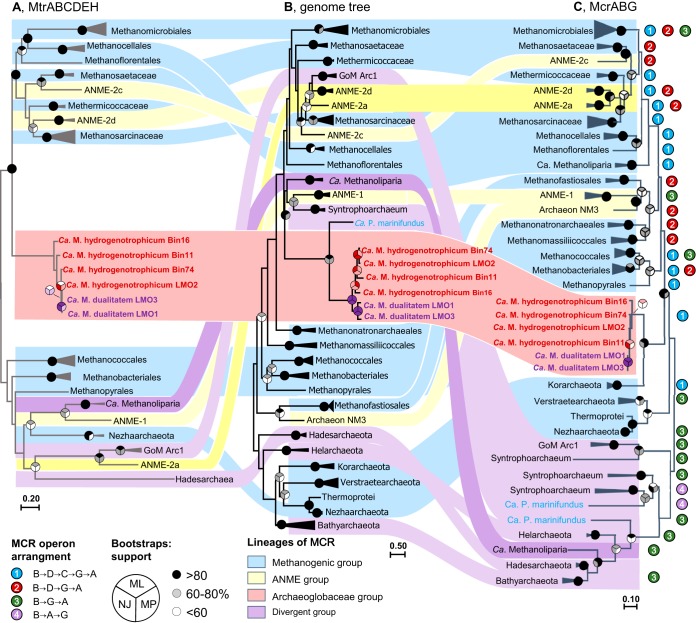
Comparison of the topologies of the concatenated MtrABCDEH gene tree (A) and genome tree (B) and the McrABG gene tree (C). The genome tree was constructed based on 3,075 aligned positions and included only genomes that contain MCR or MCR-like complexes (Table S2). McrABG and MtrABCDEH gene trees were constructed based on the alignments performed with 1,226 and 1,384 amino acid positions, respectively. Branches of the trees were collapsed as wedges and labeled according to the lowest common ancestor (LCA) of all sequences in the lineage. Genes from the same genome/lineage were linked to the corresponding branches in the genome tree. The topologies of the current trees were confirmed with previous studies (14, 15) and were supported by high bootstrap values. The *mcr* operon arrangements were determined by checking all available genomes affiliated with corresponding lineages in GenBank database. “*Ca*. Methanomixophus” is marked in red, and “*Ca*. Polytropus marinifundus” is marked in blue.

In a previous study, microbial biomass from formation waters was collected from the Jiangsu oil reservoir (18). The combined metagenomes were coassembled, and the resulting contigs were binned into 44 unique genomes (18). As revealed by the genome tree, one of the high-quality MAGs (Bin16) was phylogenetically placed into the *Archaeoglobi* clade (Fig. 1). However, the annotation of Bin16 demonstrated a distinct genotype of *Archaeoglobus* species. Surprisingly, nearly the whole set of genes associated with reversible hydrogenotrophic methanogenesis was found in Bin16, including a methyl–coenzyme M reductase (McrABG) complex (Fig. 1) and a methyl-H_4_M(S)PT:coenzyme M methyltransferase complex (MtrABCDEFGH) (Fig. 1; see also Table S4 in the supplemental material). Further phylogenetic analysis based on the concatenated amino acid alignment of McrABG placed Bin16 close to the traditional McrABGs rather than to the divergent cluster (Fig. 2C). In order to study the distribution of the new *Archaeoglobi* members in nature, the *mcrA* gene in Bin16 was used to screen metagenomes in IMG publicly available (for details, see Text S1 in the supplemental material), and the closely related *mcrA* genes were detected in two thermal aquatic metagenomes: an *in situ* cellulolytic enrichment in Great Boiling Spring (Integrated Microbial Genomes identifier [IMG-ID]: 3300000106, NV, USA) (19) and a water sample from Washburn Spring (IMG-ID: 3300005860, Yellowstone National Park, USA). These metagenomes were individually assembled and differentially binned, and then two additional *Archaeoglobi* MAGs, Bin11 and Bin74, were retrieved from metagenomes of Great Boiling Spring (IMG-ID: 3300000106, NV, USA) and Washburn Spring (IMG-ID: 3300005860, Yellowstone National Park, USA), respectively. Similarly to Bin16, Bin11 and Bin74 also contained MCR and MTR complexes. The completeness, contamination (redundancy), and number of total contigs meet the requirements for being ranked as nearly complete genomes as proposed previously by Parks et al. (20) and as high-quality draft genomes as proposed by Bowers et al. (21) (Table 1). Subsequently, examination of the contigs containing methanogenesis-related genes in these MAGs revealed that they have sequence composition characteristics (average GC content, sequencing coverage, and tetranucleotide frequencies) typical of their respective genomes (see Fig. S1 at https://figshare.com/articles/Fig_S1_Evaluation_of_the_statistical_properties_of_scaffolds_of_Ca_M_hydrogenus_Bin16_a_Bin11_b_and_Bin74_c_/9918200). To make a comprehensive study of *Archaeoglobi* MAGs, three newly assembled *Archaeoglobi* MAGs (LMO1, LMO2, and LMO3), which also contained MCR and MTR complexes homologous to those of Bin16 (Fig. 1; see also Fig. 2A and C), were downloaded from the NCBI database and included into this study (7).

**TABLE 1:** Summary statistics of new “*Ca*. Methanomixophus” MAGs

MAG	Completeness (%)	Redundancy (%)	No. of contigs	Total length (bp)	GC content (%)	No. of CDS	No. of RNAs	Relative abundance (%)^*a*^	Relative activity (%)^*a*^	RAST-ID or reference
Bin16	96.5	1.3	45	1,661,519	45.5	1,834	46	1.51	0.06	6666666.39
Bin11	94.6	1.6	133	1,403,156	47.6	1,389	31	0.35		6666666.23
Bin74	96.1	1.3	126	1,532,438	45.6	1,895	39	0.27		6666666.39
LMO1	88.89	1.31	140	1,557,307	43.8	1,851	46			7
LMO2	88.03	1.96	220	1,514,415	45.9	1,900	45			7
LMO3	97.6	0	135	1,567,523	43.9	1,872	37			7

a Data were calculated by mapping quality-controlled metagenome/metatranscriptome short reads to the MAG nucleotide files using Bowtie2 (69) and were filtered for MapQ values of >2.

**FIG 3:**
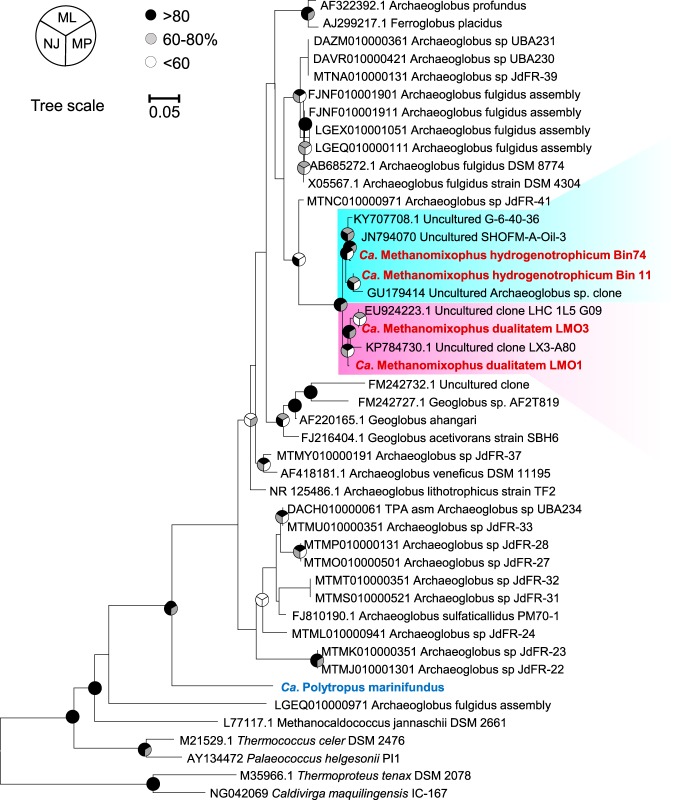
Consensus tree showing the placement of the 16S rRNA genes. “*Ca*. Methanomixophus” 16S rRNA gene sequences are marked in red, and the “*Ca*. Polytropus marinifundus” 16S rRNA gene sequence is marked in blue. The cluster contains “*Ca*. Methanomixophus hydrogenotrophicum” and “*Ca*. Methanomixophus dualitatem” sequences and is shaded in cyan and pink, respectively.

Phylogenetic analysis of taxonomic markers from Bin16, Bin11, Bin74, and LMO1 to LMO3 placed their position close to the Archaeoglobus fulgidus lineage, forming two separate clusters (Fig. 1; see also Table S8). These MAGs shared 71% to 74% of orthologous genes with the A. fulgidus genome (see Fig. S2 at https://figshare.com/articles/Fig_S2_Pairwise_comparison_of_shared_orthologous_gene_sequences_across_genomes_of_Archaeoglobi_isolates_and_Ca_Methanomixophus_MAGs_/9918206), and the average amino acid identities (AAI) between these MAGs and other *Archaeoglobus* proteomes were found to be below 65% (see Fig. S2 at https://figshare.com/articles/Fig_S2_Pairwise_comparison_of_shared_orthologous_gene_sequences_across_genomes_of_Archaeoglobi_isolates_and_Ca_Methanomixophus_MAGs_/9918206), hinting at a novel *Archaeoglobaceae* genus according to the category thresholds proposed by Konstantinidis et al. (22). The proposal for a novel genus was supported by analyses performed with the GTDBtk tool (https://github.com/Ecogenomics/GTDBTk), which uses a recently described relative evolutionary distance metric to predict the divergence of newly binned clades (23) (Table S5). The 16S rRNA gene fragments found in Bin11 (370 bp), Bin74 (918 bp), LMO1 (1,213 bp), and LMO3 (356 bp) showed 92% to 93% similarity to A. fulgidus DSM 4304 (GenBank accession number AE000782.1), which also suggested a novel genus-level lineage of *Archaeoglobaceae* for this clade (24). Phylogenetic analysis of these 16S rRNA gene sequences placed them into a monophyletic cluster with other uncultured *Archaeoglobus* clone sequences from similar environments from which these MAGs have been retrieved, such as oil reservoirs (GenBank accession numbers GU179414, KY707708, and JN794070) and hot springs (GenBank accession numbers EU924223 and KP784730); however, the results also showed that they were distantly related to A. fulgidus sequence clusters (Fig. 3). Consistent with the genome tree, 16S rRNA gene sequences from Bin11 and Bin74 were placed into a cluster separate from that containing LMO1 and LMO3 (Fig. 3). On the basis of these results, we proposed the name “*Ca*. Methanomixophus hydrogenotrophicum” gen. nov., sp. nov., for Bin11, Bin16, Bin74, and LMO2, in which “Methano” stands for methane, “mixo” means mixotrophy, and “hydrogenotrophicum” indicates the potential capability of utilizing hydrogen molecules in this lineage, and the “*Ca*. Methanomixophus dualitatem” sp. nov. for LMO1 and LMO3, in which “dualitatem” indicates the uncertain energy conservation strategy utilized in this lineage, considering that the genomic capacities of both anaerobic methane metabolism and dissimilatory sulfur metabolism were preserved (see below).

### Evolutionary history of “*Ca*. Methanomixophus” MCR complex and MTR complex.

**FIG 4:**
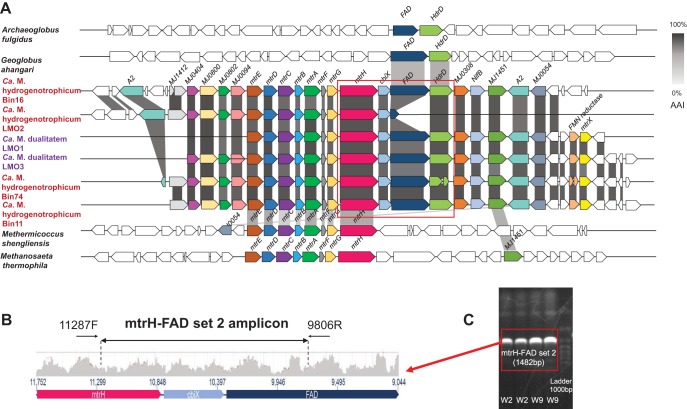
Unique gene organization in contigs from “*Ca*. Methanomixophus” MAGs. (A) Contigs containing genes most closely related to methanogens and members of the family *Archaeoglobaceae* from “*Ca*. Methanomixophus” MAGs. Annotated genes that have analogs in other contigs are shown with color coding. Percentages of amino acid identity between loci were calculated using blastp (E value < 1e−10) and are presented in grayscale. (B) Quality-filtered metagenomic short reads overlapped the contigs in the red box well, which confirms the assembly of Bin16. Black lines with arrows indicate amplicon products obtained using mtrH-FAD primer set 2. (C) Amplification of a gel band using mtrH-FAD primer set 2 confirmed the assembly of gene fusion from methanogens and members of the family *Archaeoglobaceae*. The primer information is listed in Table S3.

An operon (*mtrABCDEFGH*) consisting of genes that encode the complete methyl-H_4_M(S)PT:coenzyme M methyltransferase complex was found in all “*Ca*. Methanomixophus” MAGs (Fig. 1; see also Table S4). The blastp search revealed that these *mtrABCDEFGH* genes were predominantly associated with methanogens at 38% to 71% amino acid identity (Fig. 4A), while genes located upstream and downstream in the same contigs were found to be mostly related to *Archaeoglobi* species with average amino acid identity of 63% (Fig. 4A). Metagenomic short reads were mapped to these contigs, and the well-overlapped alignments confirmed the assembly (Fig. 4B). Furthermore, the assembly of the contig in “*Ca*. Methanomixophus hydrogenotrophicum” Bin16 was checked by PCR amplifications using DNA of W2 and W9 production water samples from the Jiangsu oil reservoir (18), and the amplicon sequences matched 99% to 100% to the partial *mtrH* gene and multiple genes located downstream in the contig (Fig. 4C; see Text S2). The emergence of colocated genes homologous to distinct microorganisms could be a result of HGT or, as suggested, a remnant character of the LCA before gene losses. To explore the evolutionary history of the MTR complex, the gene phylogeny of the MTR complex was compared with the genome phylogeny. To get a finer phylogenetic resolution of MCR and MTR complex, concatenated amino acid alignments of subunits for the same enzyme were chosen instead of individual protein sequence since these subunits were constitutive conserved in all MCR/MTR-bearing genomes and located in the same operon (25). Consistently, the genome tree and the *mtrABCDEH* gene tree both resulted in a monophyletic clade of “*Ca*. Methanomixophus,” and the branching order of “*Ca*. Methanomixophus” clade in *mtrABCDEH* gene tree is congruent with the genome tree, which branches off between Class I (*Methanopyrales*, *Methanococcales* and *Methanobacteriales*) and Class II (Methanomicrobia, comprised of *Methanosarcinales*, *Methanocellales* and *Methanomicrobiales*) methanogens (13) with strong bootstrap supports (Fig. 2A and B). No potential mobile genetic elements (such as integrons, transposons, repeat sequences and tRNAs), which are hallmarks for HGT, could be identified in the flanking regions of *mtr* genes (Fig. 4A; see also Table S6). Further analysis of GC content and 4mer frequencies of these *mtr* operons also showed consistent profiles with the surrounding gene context in the contigs (see Fig. S3 at https://figshare.com/articles/Fig_S3_Comparison_of_tetranucleotide_frequencies_and_GC_content_between_MCR_MTR_operons_and_surrounding_gene_context_in_the_scaffolds_/9918209), suggesting that either these *mtr* operons are inherited vertically or the acquirement of this operon through HGT did not occur in recent evolutionary history (17). These evidence collectively suggests a vertical inheritance of evolutionary history for “*Ca*. Methanomixophus” MTR.

Genes (*mcrABG*) encoding all subunits of methyl-coenzyme M reductase complex, where ligand-binding sites for CoB, CoM, and cofactor F_430_ are well conserved (see Fig. S4 at https://figshare.com/articles/Fig_S4_Conservation_of_functionally_important_McrA_residues_including_ligand_cavity_sites_and_F420_CoB_and_CoM_binding_sites_as_revealed_by_Ermler_et_al_21_/9918236), was found in these MAGs. As revealed by structure prediction, the McrA, McrB, and McrG within these genomes showed high similarity to the crystal structures from Methanopyrus kandleri. (see Fig. S5 at https://figshare.com/articles/Fig_S5_Structural_modelling_and_active_sites_of_the_MCR_complex_in_Ca_M_Bin16_/9918233). BLASTP search results demonstrated that the *mcrABG* genes in these MAGs are most similar to genes in “*Ca*. Methanodesulfokores washburnensis” (77% to 83% amino acid identity), which is a newly assembled MAG affiliated with the Korarchaeota phylum (26). Phylogenetic analysis of the concatenated McrABG sequences also placed them with “*Ca*. Methanodesulfokores washburnensis” (denoted “Korarchaeota” in Fig. 2C), forming a basal branch to euryarchaeal lineages (Fig. 2C), which is incongruent with the genome tree (Fig. 2B). However, it should be noted that despite the largely congruent branching order in the MCR tree and genome tree for most members, incongruent positions were also observed for some traditional methanogens in this study (Fig. 2B and C) and in several other studies (16, 17). Further analysis revealed that the *mcr* operons in this lineage contain two extra genes, *mcrC* and *mcrD*, with unknown function (27). This arrangement of *mcrBDCGA* in an operon resembles the *mcr* operons in most conventional euryarchaeal methanogens and “*Ca*. Methanodesulfokores washburnensis,” rather than the arrangement of *mcrBGA* in “*Ca*. Verstraetearchaeota” (6) or the divergent type *mcrBAG* in some “*Ca*. Syntrophoarchaeum” and “*Ca*. Polytropus marinifundus” isolates (Fig. 2C) (17). As a result, the *mcr* operon in “*Ca*. Methanomixophus” genomes is likely to be accepted through HGT but would likely be from a donor different from “*Ca*. Polytropus marinifundus” (17) (Fig. 2C), despite the fact that we did not find any mobile elements or divergent GC or 4mer profiles surrounding the gene context of the MCR operons (Table S6; see also Fig. S3 at https://figshare.com/articles/Fig_S3_Comparison_of_tetranucleotide_frequencies_and_GC_content_between_MCR_MTR_operons_and_surrounding_gene_context_in_the_scaffolds_/9918209).

### Metabolic potential and *in situ* activity of “*Ca*. Methanomixophus.”

In contrast to all *Archaeoglobi* known so far (11), including “*Ca*. Polytropus marinifundus,” genes encoding key enzymes associated with dissimilatory sulfate reduction, namely, the genes encoding sulfate adenylyltransferase (*sat*), adenylylsulfate reductase (*aprAB*), and dissimilatory sulfide reductase (*dsrAB*), are absent in “*Ca*. Methanomixophus hydrogenotrophicum” genomes (Fig. 1; see also Table S4). Further examination of metagenome contigs and short reads did not reveal any related sequences for these missing genes (see Text S1). To confirm that the loss of sulfate-reducing genes in these MAGs was not an artifact caused by an assembly mistake, we took the genes that have been found to locate upstream or downstream of sulfate-reducing genes in public Archaeoglobus fulgidus reference genomes (Table S2) as anchors, and the absence of genes encoding sulfate-reducing products in “*Ca*. Methanomixophus hydrogenotrophicum” Bin16 was validated by the presence of amplicons spanning those anchor genes and their adjacent genes (see Fig. S6 at https://figshare.com/articles/Fig_S6_Contigs_containing_sulfate-reducing_genes_in_Ca_Methanomixophus_MAGs_/9918203; see also Table S3). The only exception was the presence of DsrC, encoded by the *dsrC* gene, with two conserved cysteines in the C-terminal region (see Fig. S7 at https://figshare.com/articles/Fig_S7_Trimmed_alignment_of_DsrC_TsuE_AspA_amino_acid_sequences_/9918230). DsrC is a small protein functioning in the terminal step of sulfate reduction (28). However, without the dissimilatory sulfate reductase (*dsrAB*) gene, its role in this lineage remains unclear. In a previous study, *dsrC* genes were found in organisms that do not have the *dsrAB* genes (29) and their products were predicted to function like TusE proteins, which were shown to participate in a sulfur-relay system (30).

**FIG 5:**
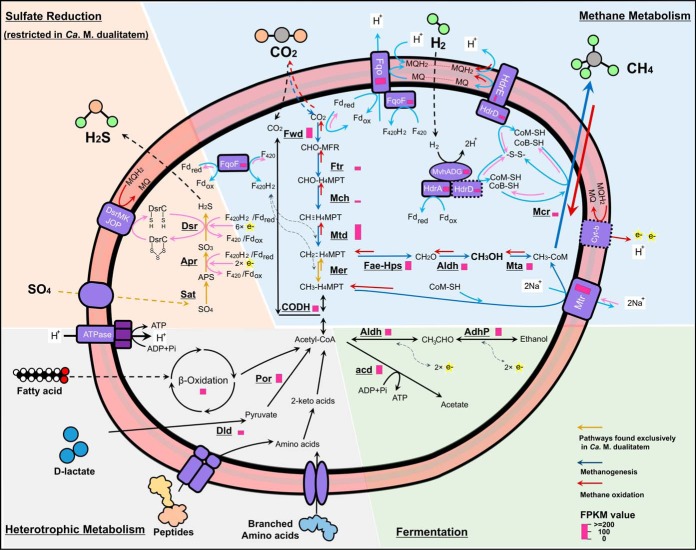
Metabolic reconstruction of “*Ca*. Methanomixophus.” The genes used to construct this metabolic network are listed in Table S4. Pathways for methanogenesis are depicted in blue, pathways for anaerobic methane oxidation coupled with sulfate reduction are depicted in red, and other pathways are depicted in black. The sulfate reduction pathway was found only in “*Ca*. Methanomixophus dualitatem,” whereas other pathways were found to be conserved in all “*Ca*. Methanomixophus” MAGs. FPKM values are represented as red bars close to the gene names, and an average FPKM value representing the transcribed genes was used for enzymes comprising multiple subunits. Complexes without proteomic evidence are depicted with dashed lines. (See Fig. 1 legend for definitions of abbreviations.)

“*Ca*. Methanomixophus hydrogenotrophicum” genomes also lack genes encoding the quinone-interacting membrane-bound oxidoreductase (QmoABC) complex. The QmoABC complex was previously proposed to link the electron transfer chain to the first reductive step of sulfate reduction in *Archaeoglobus* (31). The absence of *qmoABC* genes is consistent with the absence of the aforementioned dissimilatory sulfate reduction-related genes, indicating the inability of this clade to generate ATP through sulfate reduction. Furthermore, genes for reducing other electron acceptors, such as nitrate, thiosulfate, and iron, all of which have been shown to be widely utilized by other *Archaeoglobi* species, were all missing in this clade (Fig. 1). Therefore, this new lineage might not gain energy through respiration (Fig. 5).

“*Ca*. Methanomixophus hydrogenotrophicum” genomes encodes a nearly complete archaeal type Wood-Ljungdahl pathway, except for the gene encoding N^5^,N^10^-methylene-H_4_MPT reductase (Mer) (Table S4). The methylene-tetrahydrofolate reductase (MetF), which was previously suggested to act as a substitute for Mer in the reverse process of methanogenesis (32), was also missing in all “*Ca*. Methanomixophus hydrogenotrophicum” genomes (Fig. 1). Given the absence of *mer* and *metF* in both genomes and unassembled metagenomic contigs, a bypass pathway for methanol-utilizing methanogenesis in which methanol is oxidized to formaldehyde and subsequently to N^5^,N^10^-methylene-H_4_MPT has been proposed (32, 33) (Fig. 5). The presence of genes encoding a fusion protein of formaldehyde-activating enzyme and hexulose-6-phosphate synthase (FaeB-Hps) and encoding short-chain alcohol dehydrogenases (Aldh) and CoM methyltransferase (Mta) suggested the presence of this bypass pathway in this new clade (33, 34).

“*Ca*. Methanomixophus hydrogenotrophicum” genomes encode an F_420_-nonreducing hydrogenase (MvhADG) (Fig. 1). In hydrogenotrophic methanogens, this hydrogenase forms a complex with heterodisulfide reductase (HdrABC) which bifurcates electrons from H_2_ to reduce ferredoxin and heterodisulfide, likely coenzyme M (CoM) and coenzyme B (CoB), in methanogens and a gamma subunit of dissimilatory sulfate reductase (DsrC) in *Archaeoglob*us (35, 36). Only an HdrA subunit which contains flavin adenine dinucleotide (FAD) is present in the genomes, indicating the ability to reduce ferredoxin. However, no subunit of HdrB, which catalyzes the direct reduction of disulfide, was found. Instead, the genomes contain another gene encoding HdrD, which is a fusion of HdrB and HdrC (37), suggesting that HdrD may replace HdrB in the Mvh/Hdr complex (26) (Fig. 5). Noteworthy is the fact that the lack of an HdrB subunit has also been observed in an uncultured *Archaea* genome (26) and a sulfate-reducing bacteria (38), hinting at an underrepresented energy-conserving mechanism within anaerobic microorganisms. However, future studies on the proteome of “*Ca*. Methanomixophus hydrogenus” and *in vitro* investigations will be required to elucidate the role of the HdrD detected here. A gene cluster encoding an F_420_H_2_:quinone oxidoreductase (Fqo) was found in this clade (Fig. 1; see also Table S4). The FqoF subunit is usually considered bound to the membrane integral module of FqoBCDI, forming the Fqo complex which oxidizes F_420_H_2_ coupled with menaquinone (MQ) reduction in *Archaeoglobus* (39). Nevertheless, it has been proposed that the FqoF subunit might be soluble in cytoplasm and might independently catalyze the reduction of F_420_ with Fd_red_ oxidation in *Archaeoglobus* species (40). A similar mechanism has also been proposed for the homologous FqoF in the *Methanomixophus* genomes analyzed in this study, which also contain FAD and iron-sulfur clusters, that is, mediation of the electron transfer from Fd_red_ onto F_420_ (Fig. 5). Furthermore, as shown in another study, the Fpo/Fqo complex is capable of catalyzing a Fd:MP/Fd:MQ oxidoreductase reaction independently of the FpoF/FqoF subunit (41) (Fig. 5). Additionally, there is also a potential of the FqoF-associated Fqo complex to link Fd_red_ oxidation to both F_420_ reduction and menaquinone reduction by flavin-dependent electron bifurcation (42) (Fig. 5). In “*Ca*. Methanomixophus hydrogenotrophicum,” the electrons carried by menaquinone (MQ) might be then transferred through the membrane-bound heterodisulfide reductase (HdrDE) to reduce CoM-S-S-CoB (43, 44) (Fig. 5).

However, the key enzyme of hydrogenotrophic methanogenesis, F_420_-reducing hydrogenase, is missing in all MAGs, which rules out the possibility of hydrogenotrophic CO_2_-reducing methanogenesis in these organisms (1). On the other hand, the detection of methyltransferase (MtaABC) and Mvh:Hdr complex indicates a potential hydrogen-dependent methylotrophic (methanol) methanogenesis lifestyle in this clade, which is similar in mechanism to those seen with the members of *Methanomassiliicoccales* (43) (Fig. 5). In this case, electrons from H_2_ could be transferred to reduction of CoM-S-S-CoB facilitated by Fqo and Hdr complexes as mentioned above. The methyl branch of the Wood-Ljungdahl pathway and the MTR complex would function in anabolic production of acetyl coenzyme A (acetyl-CoA) from CO_2_ and CH_3_-CoM, respectively (Fig. 5).

The genomic features collectively suggest a methanogenic lifestyle for “*Ca*. Methanomixophus hydrogenotrophicum”; however, its potential role as an anaerobic methane oxidizer could not be ruled out. In such a case, “*Ca*. Methanomixophus hydrogenotrophicum” may oxidize methane anaerobically through a reversible methanogenesis pathway and would require syntrophic partners as electron sinks (45), likely the sulfate-reducing microorganisms detected in the same environments (18, 41) (Fig. 5). And the membrane-associated electron-transfer HdrDE complexes would mediate the electron flow in a reverse direction of methanogenesis (Fig. 5).

“*Ca*. Methanomixophus dualitatem” genomes displayed high similarity in genomic features with “*Ca*. Methanomixophus hydrogenotrophicum,” containing MTR, MCR, and Mvh/Hdr complexes (Fig. 1). Interestingly, the gene coding for N^5^,N^10^-methylene-H_4_MPT reductase (*mer*) was found in “*Ca*. Methanomixophus dualitatem” genomes, completing the WL pathway in this lineage (Fig. 5). Hence, “*Ca*. Methanomixophus dualitatem” may also conserve energy through hydrogenotrophic methanogenesis (Fig. 5). “*Ca*. Methanomixophus dualitatem” genomes possess extra genes (*sat*, *AprAB*, *dsrABC*, and *qmoABC*) for sulfate reduction, suggesting that “*Ca*. Methanomixophus dualitatem” members might alternatively perform sulfate-dependent anaerobic methane oxidation in single cells (45) (Fig. 5). Interestingly, a newly assembled Korarchaeota genome in “*Ca*. Methanodesulfokores washburnensis” carries genes that code for a partial dissimilatory sulfate reduction pathway, as well as the co-occurring genes coding for anaerobic methane oxidation, hinting at a close link between anaerobic methane metabolism and dissimilatory sulfur reduction in *Archaea* members (26).

In addition, genes involved in fatty acid degradation (*β*-oxidation) and in degradation of peptides/amino acids, d-lactate metabolism, and acetogenesis were found in all “*Ca*. Methanomixophus” MAGs (Fig. 5; see also Table S4) (see Text S1 for more details), which is consistent with results from the closely related *Archaeoglobus* species (11).

Metatranscriptomic analysis of “*Ca*. Methanomixophus hydrogenotrophicum” Bin16 in sample W15 obtained from the Jiangsu oil reservoir demonstrated that most genes associated with the common pathway of methanogenesis, namely, *mcrABCG*, *mtrA-H*, *ftr*, *fwdABDEFG*, *mtd*, and *fae-hps*, were transcribed to different degrees (Fig. 5; see also Table S7). Unexpectedly, the transcriptional levels of individual genes coding for the subunits of Mtr were found to be greatly uneven (Table S7). It is also noteworthy that the fragments per kilobase per million (FPKM) values of *mcrABG* genes were relatively low compared with those seen with other genes described here. Since these FPKM values were calculated based on the metatranscriptome data set representing a very complex microbial community, future research, likely performed on the basis of pure culture isolation, will be required to resolve these issues. Meanwhile, genes specific to methanogenesis from both methanol (*mtaABC*) and hydrogen (*mvhADG*) were transcribed (Fig. 5; see also Table S7), indicating an active hydrogen-dependent methylotrophic methanogenesis process of “*Ca*. Methanomixophus hydrogenotrophicum” in the oil reservoir. Transcripts of multiple genes involved in d-lactate degradation (*dld*) and in degradation of fatty acids and peptides were detected, as well as transcripts of those involved in degradation of acetyl-CoA synthetase (ADP-forming) (*acd*), aldehyde dehydrogenases (*aldh*), and alcohol dehydrogenases (*adhP*), suggesting that heterotrophic fermentation was also active in “*Ca*. Methanomixophus hydrogenotrophicum” Bin16 (Fig. 5).

### Evolutionary origin of *Archaeoglobi*.

**FIG 6:**
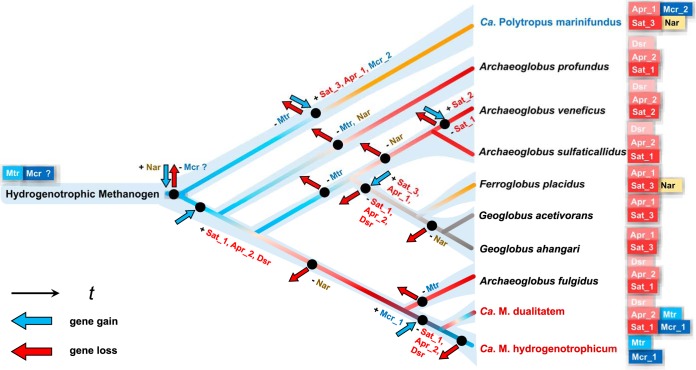
Proposed evolutionary transition of *Archaeoglobi* from methanogen mostly supported by this study. The backbone tree was simplified from the genome tree in Fig. 1. Branches representing methanogens and sulfate-reducing, nitrate-reducing, and iron-reducing microorganisms are shaded in blue and red, yellow, and gray, respectively. The putative events of gene loss/gain were inferred based on the LCA of the lineages. The subgroups of genes *mcr*, *apr*, and *sat* were determined according to their phylogenies (Fig. 2; see also Fig. S8 to 11 [see text for URLs]). Data representing genes for catabolism of fatty acids and lactate and proteins/peptides are not included in this figure, and only genome-representing isolates apart from “*Ca*. Polytropus marinifundus” and genomes discovered in this study were included for reasons of clarity. “*Ca*. Methanomixophus” genomes are marked in red, and “*Ca*. Polytropus marinifundus” is marked in blue.

The discovery of hyperthermophilic “*Ca*. Methanomixophus” sheds light on how the transition from a methanogenic LCA to nonmethanogenic *Archaeoglobi*, which was likely facilitated by multiple individual gene gains (through HGT) and losses, might have occurred (Fig. 6). Previous studies have shown that the *dsrAB* genes in *Archaeoglobus* species were accepted from a bacterial donor through HGT (see Fig. S8 at https://figshare.com/articles/Fig_S8_Phylogenetic_trees_showing_the_placement_of_concatenated_DsrAB_sequences_/9918215) (14), and phylogenetic analysis of *sat* and *aprAB* in *Archaeoglobus* species and of *narGHI* in Ferroglobus placidus and “*Ca*. Polytropus marinifundus” also suggested their bacterial origin (see Fig. S9, S10, and S11 at https://figshare.com/articles/Fig_S9_Phylogenetic_trees_showing_the_placement_of_concatenated_AprAB_sequences_/9918212, https://figshare.com/articles/Fig_S10_Phylogenetic_trees_showing_the_placement_of_concatenated_NarGHI_sequences_/9918221, and https://figshare.com/articles/Fig_S11_Phylogenetic_trees_showing_the_placement_of_the_Sat_sequences_/9918227, respectively) (14, 46). Furthermore, phylogenetic analysis demonstrated that the *sat* and *aprAB* genes in *Geoglobus* species, Ferroglobus placidus, and “*Ca*. Polytropus marinifundus” were located in separate clusters, distantly related to *Archaeoglobus* species (see Fig. S9 and S11 at https://figshare.com/articles/Fig_S9_Phylogenetic_trees_showing_the_placement_of_concatenated_AprAB_sequences_/9918212 and https://figshare.com/articles/Fig_S11_Phylogenetic_trees_showing_the_placement_of_the_Sat_sequences_/9918227, respectively), which indicates multiple HGT events and different donors of these genes during evolution (Fig. 6). Consistently, the contigs in “*Ca*. Methanomixophus dualitatem” genomes that flank *sat* and *apr* genes showed many hallmarks of HGT (see Fig. S6 at https://figshare.com/articles/Fig_S6_Contigs_containing_sulfate-reducing_genes_in_Ca_Methanomixophus_MAGs_/9918203), including repeat regions, transposons, and tRNA genes often found in association with genomic islands (47), and are common target sites for phage attachment and integration (48). Overall, sulfate-reducing ability was largely retained in *Archaeoglobus* members as well as in “*Ca*. Methanomixophus dualitatem,” as found in this study, which usually inhabit submarine hydrothermal environments (49, 50), hot springs (51), and deep oil reservoirs (52), with elevated concentrations of carbon dioxide, methane, hydrogen, hydrogen sulfide, and sulfate.

The recent discovered hydrogenotrophic methanogenesis pathway in “*Ca*. Verstraetearchaeota,” which had previously been considered a genus of strict methylotrophic methanogens, implies an ancient origin of hydrogenotrophic methanogenesis and a later adaptation of methylotrophic methanogenesis for all methanogens (53). On the basis of the detection of a MTR complex and a traditional MCR complex in this novel *Archaeoglobi* genus, as well as the widespread WL pathway within *Archaeoglobi* lineages, we propose a similar evolutionary trend for *Archaeoglobi*: hydrogenotrophic methanogenesis first evolved to support life in a nutrient-poor environment that required using only CO_2_ as a source of carbon, and then complex carbon source degradation ability evolved as more-complex nutrient environments became available, followed by accepting respiring ability using different electron sinks from bacterial members, which made them more metabolically flexible under different environmental conditions (Fig. 6). The previous discovery of the basal member of *Archaeoglobi*, “*Ca*. Polytropus marinifundus,” suggests the acquisition of the divergent MCR complex through a HGT event (17). In this study, however, we expanded the current knowledge by showing that (i) two separate HGT events of different types of MCR complexes, from different donors, may have occurred (Fig. 6) and that (ii) despite the fact that the original MCR and MTR complexes encoded in the *Archaeoglobi* LCA have been substantially lost in most *Archaeoglobi* lineages, the clade of “*Ca*. Methanomixophus” retains the original MTR complex (Fig. 6). While other scenarios in which the MTR complex in “*Ca*. Methanomixophus” could also be acquired via HGT remain possible, this condition is associated with the lowest number of MCR/MTR gene loss events during evolution (see Fig. S13a at https://figshare.com/articles/Fig_S13_Alternative_scenarios_that_might_explain_the_evolution_transition_of_Archaeoglobi_from_methanogen_/9918218). Also, the possibility that “*Ca*. Methanomixophus” originally inherited both the MCR and MTR complexes from the *Archaeoglobi* LCA cannot be excluded (see Fig. S13b). Considering the highly complex evolutionary history of the *Archaea* species that encode the MCR complex, recovering more MCR-encoding lineages throughout the archaeal tree of life would help to make their evolutionary history and metabolic role in the hydrothermal subsurface biosphere clear.

## MATERIALS AND METHODS

### Sample collection and data availability.

Production water was collected from the Jiangsu oil reservoir (Yangzhou, China) (18); 40 liters of the production water was collected for DNA extraction, and another 40 liters was collected for RNA extraction and was stabilized using a 10% (vol/vol) stop solution (95% ethanol, 5% TRIzol [Life Technology]). All samples were kept on ice and transported to the laboratory within 4 h, and then DNA/RNA was extracted using a PowerMicrobiome RNA isolation kit (MO BIO). After sequencing on an Illumina Miseq platform was performed, metagenomic and metatranscriptomic data sets were processed as previously described (18). In brief, raw reads were quality filtered using PRINSEQ v0.20.4 (54) and were then assembled using SPAdes v3.7.0 (55), and MAGs were generated by GroopM (56). Newly assembled *Archaeoglobi* MAGs, including MAGs of “*Ca*. Polytropus marinifundus” (Juan de Fuca Ridge, Northeast Pacific Ocean), *Archaeoglobi* WYZ-LMO1 (Washburn Spring, WY, USA), WYZ-LMO2 (Obsidian Pool, WY, USA), and WYZ-LMO3 (Obsidian Pool, WY, USA), were retrieved from previous studies (7, 17).

### Metagenome assembly and population genome binning.

Metagenomes of water samples from Great Boiling Spring, NV (IMG-ID: 3300000106), and Yellowstone National Park, WY (IMG-ID: 3300005860), were downloaded from IMG/M ER and were trimmed for quality control and adapter removal using Trim_galore (http://www.bioinformatics.babraham.ac.uk/projects/trim_galore/). The processed reads were *de novo* assembled using SPAdes v3.7.0 (55) with a ‘–meta’ model and with different k-mer settings (47, 79, 93, 127). The contigs generated by the different k-mer settings were evaluated using MetaQUAST v2.2 (57), and the k-mer of 127 was found to be the best (see Table S1 in the supplemental material). Scaffolds were then binned into population genomes using MetaBAT2 v0.32.4 with default settings (58).

10.1128/mSystems.00651-19.3 TABLE S1 Statistics of assemblies with different k-mers generated by MetaQUAST. Download Table S1, DOCX file, 0.02 MB. Copyright © 2020 Liu et al. 2020 Liu et al. This content is distributed under the terms of the Creative Commons Attribution 4.0 International license.

10.1128/mSystems.00651-19.4 TABLE S2 Genomes used for construction of the genome tree. Download Table S2, DOCX file, 0.02 MB. Copyright © 2020 Liu et al. 2020 Liu et al. This content is distributed under the terms of the Creative Commons Attribution 4.0 International license.

10.1128/mSystems.00651-19.5 TABLE S3 Primers for amplification of uniquely organized genes in “*Ca*. Methanomixophus” MAGs. Download Table S3, DOCX file, 0.01 MB. Copyright © 2020 Liu et al. 2020 Liu et al. This content is distributed under the terms of the Creative Commons Attribution 4.0 International license.

10.1128/mSystems.00651-19.6 TABLE S4 Annotated genes used for metabolic reconstruction of the “*Ca*. Methanomixophus” MAGs. Coloring corresponds to Fig. 1. Download Table S4, XLSX file, 0.02 MB. Copyright © 2020 Liu et al. 2020 Liu et al. This content is distributed under the terms of the Creative Commons Attribution 4.0 International license.

10.1128/mSystems.00651-19.7 TABLE S5 Taxonomic classification of *Archaeoglobi* MAGs using the GTDBtk tool. Download Table S5, DOCX file, 0.01 MB. Copyright © 2020 Liu et al. 2020 Liu et al. This content is distributed under the terms of the Creative Commons Attribution 4.0 International license.

10.1128/mSystems.00651-19.8 TABLE S6 Mobile elements found in “*Ca*. Methanomixophus” MAGs. Download Table S6, XLSX file, 0.02 MB. Copyright © 2020 Liu et al. 2020 Liu et al. This content is distributed under the terms of the Creative Commons Attribution 4.0 International license.

10.1128/mSystems.00651-19.9 TABLE S7 FPKM values of annotated genes in sample W15. For genes with multiple copies, an averaged value was used as representative. Coloring corresponds to Fig. 1. Download Table S7, XLSX file, 0.02 MB. Copyright © 2020 Liu et al. 2020 Liu et al. This content is distributed under the terms of the Creative Commons Attribution 4.0 International license.

10.1128/mSystems.00651-19.10 TABLE S8 Comparative analysis of KO orthologs shared by “*Ca*. Polytropus marinifundus” and “*Ca*. Methanomixophus.” KO orthologs in the pan-genome of Archaeoglobus fulgidus were used as an outgroup. Orthologs found in the “*Ca*. Polytropus marinifundus” and “*Ca*. Methanomixophus” pan-genome but not in the Archaeoglobus fulgidus pan-genome are indicated in blue, whereas orthologs streamlined in the “*Ca*. Polytropus marinifundus” and “*Ca*. Methanomixophus” pan-genome compared with the Archaeoglobus fulgidus pan-genome are indicated in red. Download Table S8, XLSX file, 0.04 MB. Copyright © 2020 Liu et al. 2020 Liu et al. This content is distributed under the terms of the Creative Commons Attribution 4.0 International license.

### Genome annotation.

The annotation of scaffolds in population genomes was confirmed using two parallel methods. First, nucleotide sequences of genomes were submitted to theRAST server for annotation using subsystem technology (59). Second, nucleotide sequences of genomes were translated into amino acid sequences using Prodigal v2.6 (60) with default settings, and the amino acid files were submitted to BlastKOALA server (61) in the prokaryotic species database for assigning knockout (KO) numbers. Only genes with accordant annotations from both methods were included in this study. To characterize the mobile elements in the population genomes, we also searched genomes for signatures of known integrons and transposons. A local database of integrons was created from the nucleotide sequences for all integrases available in the database INTEGRALL v1.2.8414 (10,533 records in total) (62). A gene was recognized as an integron or insertion if the BLAST hit (blastn) had a minimum of 30% identity over 75% of the gene length, according to the previously published threshold (7). Amino acid files of population genomes were submitted to the ISfinder online server (updated on 4 March 2019) (63) for searching for transposons using the ‘blastp’ tool (identity > 30%, coverage > 75%, E value < 1 × 10^−5^).

### Construction of consensus tree.

For the phylogenetic analysis of functional marker proteins (McrABG, MtrABCDEH, Sat, DsrAB, AprAB, and NarGHI), amino acid sequences of individual genes were extracted from assembled genome bins and reference genomes listed in Table S2. Sequences were aligned using MAFFT (64) with iterative refinement methods (‘G-INS-i’) and then refined (retained columns with <10% gaps), and alignments of subunits for the same enzyme were concatenated in a single alignment to get a higher level of phylogenetic resolution. To construct the genome tree, all reference genomes and assembled genome bins were pooled into PhyloPhlAn v0.99, which extracts and aligns 400 conserved protein sequences from the genomes (65). The concatenated alignment file was then extracted for phylogenomic tree building. Consensus trees were built based on three different methods similar to those described in a previous study (14). Maximum likelihood (ML) trees were reconstructed using IQ-tree v. 1.6.7 under standard conditions of model selection with 1,000 ultrafast bootstraps. Neighbor joining (NJ) trees were calculated in the PHYLIP software package (66) using the “NEIGHBOR” function based on the JTT matrix model (67), and bootstrap analysis was performed with 1,000 resamplings (PHYLIP SEQBOOT). Maximum parsimony (MP) trees were constructed in MEGA 7 (68) with 100 and 500 bootstrap replications for the genome tree and gene trees, respectively. The three trees were then combined into a consensus tree by using the extended majority rule in PHYLIP CONSENSE (66). Branch lengths of the consensus tree were inferred by using the JTT matrix model (PHYLIP PROML) (66).

### Metatranscriptome analysis.

Metatranscriptomes of sample W15 obtained from the Jiangsu oil reservoir were processed as previously described (18). In brief, raw reads were trimmed by quality analysis using Prinseq (with parameters identical to those used in the metagenome analysis), and the quality-controlled reads were mapped to coding DNA sequences (CDS) of the whole assembly file which has been used to generate the population genomes (18) using Bowtie2 (69) with default settings. Mapping reads are then filtered for MapQ values of >2 in order to remove ambiguously mapping reads (70). eXpress v1.5.1 (71) was used to calculate FPKM (fragments per kilobase per million fragments mapped).

### Data availability.

Amplicon sequences were deposited in the NCBI database under accession numbers MN891846 to MN891915. The genome files assembled in this study are available in the RAST server with the IDs mentioned in Table 1 and are also attached here as Text S2 in the supplemental material.

10.1128/mSystems.00651-19.1 TEXT S1 Supplemental methods and results. Download Text S1, DOCX file, 0.05 MB. Copyright © 2020 Liu et al. 2020 Liu et al. This content is distributed under the terms of the Creative Commons Attribution 4.0 International license.

10.1128/mSystems.00651-19.2 TEXT S2 Nucleotide sequences of contigs of the MAGs assembled in this study. Download Text S2, DOCX file, 1.3 MB. Copyright © 2020 Liu et al. 2020 Liu et al. This content is distributed under the terms of the Creative Commons Attribution 4.0 International license.
