# Supplementary material for: Genomic and Transcriptomic Evidence Supports Methane Metabolism in Archaeoglobi
Source: mSystems. 2020 Mar 17;5(2):e00651-19. doi: 10.1128/mSystems.00651-19 (PMC7380581; doi:10.1128/mSystems.00651-19)
Supplement: TABLE S1 [file mSystems.00651-19-st001.docx]

**Table S1. Statistics of assemblies with different k-mer generated by MetaQUAST v2.2** (24)

| **Metagenome** | **3300000106_** | | | | **3300005860** | | | |
| --- | --- | --- | --- | --- | --- | --- | --- | --- |
| **Kmer** | **K33** | **K75** | **K99** | **K125** | **K49** | **K75** | **K99** | **K125** |
| # contigs (>= 0 bp) | 1025094 | 976548 | 602533 | **201608** | 1122118 | 878291 | 2572064 | **591391** |
| # contigs (>= 1 k bp) | 21433 | 24659 | 25948 | **28200** | 60278 | 63381 | 70600 | **76330** |
| # contigs (>= 5 k bp) | 2415 | 2478 | 3064 | **3862** | 9277 | 10147 | 8730 | **12017** |
| # contigs (>= 10 k bp) | 649 | 588 | 868 | **1182** | 3540 | 4086 | 2799 | **4758** |
| # contigs (>= 25 k bp) | 42 | 47 | 89 | **149** | 795 | 1076 | 400 | **1018** |
| # contigs (>= 50 k bp) | 1 | 9 | 12 | **23** | 200 | 313 | 65 | **284** |
| Tot. len. (>= 0 bp) | 1.71E+08 | 2.21E+08 | 1.99E+08 | **1.5E+08** | 4.03E+08 | 4.23E+08 | 6.86E+08 | **4.59E+08** |
| Tot. len. (>= 1 k bp) | 59026295 | 65433853 | 74155731 | **88342178** | 2.17E+08 | 2.45E+08 | 2.12E+08 | **2.83E+08** |
| Tot. len. (>= 5 k bp) | 22137684 | 22179562 | 29730562 | **39465321** | 1.16E+08 | 1.39E+08 | 92584722 | **1.57E+08** |
| Tot. len. (>= 10 k bp) | 10186989 | 9441837 | 14797455 | **21201269** | 76817086 | 97259749 | 51926550 | **1.06E+08** |
| Tot. len. (>= 25 k bp) | 1400946 | 1796225 | 3492368 | **5989452** | 36554903 | 52471331 | 17215522 | **51042353** |
| Tot. len. (>= 50 k bp) | 56566 | 591997 | 973576 | **1780509** | 16443516 | 26177805 | 5886260 | **26199871** |
| # contigs | 51359 | 57461 | 60278 | **59885** | 130147 | 124623 | 165886 | **161448** |
| Largest contig | 56566 | 95533 | 146185 | **146185** | 327070 | 314113 | 442465 | **829067** |
| Tot. len. | 79436061 | 87850589 | 97640745 | **1.1E+08** | 2.66E+08 | 2.87E+08 | 2.77E+08 | **3.41E+08** |
| GC (%) | 41.49 | 41.55 | 41.98 | **42.3** | 44.98 | 45.01 | 45.03 | **45.1** |
| N50 | 2186 | 2119 | 2363 | **3000** | 3788 | 4653 | 2537 | **4149** |
| N75 | 980 | 985 | 1027 | **1206** | 1309 | 1525 | 1053 | **1372** |
| L50 | 7947 | 9430 | 8780 | **7949** | 13066 | 11051 | 21951 | **15122** |
| L75 | 21990 | 25117 | 25036 | **22930** | 44548 | 39614 | 66287 | **53176** |
| # N's per 100 kbp | 0 | 0 | 0 | **0** | 0 | 0 | 0 | **0** |
