# Supplementary material for: Genomic and Transcriptomic Evidence Supports Methane Metabolism in Archaeoglobi
Source: mSystems. 2020 Mar 17;5(2):e00651-19. doi: 10.1128/mSystems.00651-19 (PMC7380581; doi:10.1128/mSystems.00651-19)
Supplement: TABLE S2 [file mSystems.00651-19-st002.docx]

**Table S2. Genomes used for construction of the genome tree.**

| **Organism** | **Accession No.** |
| --- | --- |
| Arc I group archaeon B15fssc0709_Meth_Bin003 | LNGF00000000.1 |
| Arc I group archaeon BMIXfssc0709_Meth_Bin006 | LNJC00000000.1 |
| Hadesarchaea archaeon YNP_N21 | LQMP00000000.1 |
| Hadesarchaea archaeon YNP_45 | LQMQ00000000.1 |
| Verstraetearchaeota V2 | GCA_001717035.1 |
| Verstraetearchaeota V3 | MAGU00000000.1 |
| Candidatus Bathyarchaeota archaeon BA1 | LIHJ00000000.1 |
| Candidatus Bathyarchaeota archaeon BA2 | LIHK00000000.1 |
| miscellaneous Crenarchaeota group-6 archaeon AD8-1 | LFWW00000000.1 |
| Candidatus Bathyarchaeota archaeon B26_2 | LUCE00000000.1 |
| *Methanopyrus kandleri* AV19 | AE009439.1 |
| *Methanobacterium formicicum* DSM 3637 | AMPO00000000.1 |
| *Methanobrevibacter olleyae* ASM156324v1 | CP014265.1 |
| *Methanothermobacter thermautotrophicus* str. Delta H | AE000666.1 |
| *Methanothermus fervidus* type strain V24S | CP002278.1 |
| *Methanosphaera stadtmanae* DSM 3091 | CP000102.1 |
| *Methanocaldococcus vulcanius* M7 | CP001787.1 |
| *Methanocaldococcus infernus* ME | CP002009.1 |
| *Methanocaldococcus jannaschii* DSM 2661 | L77117.1 |
| *Methanotorris formicicus* Mc-S-70 ctg188 | AGJL00000000.1 |
| *Methanotorris igneus* Kol 5 | CP002737.1 |
| *Methanothermococcus thermolithotrophicus* DSM 2095 | AQXV00000000.1 |
| *Methanomassiliicoccus luminyensis* B10 | CAJE00000000.1 |
| Candidatus *Methanomassiliicoccus intestinalis* Issoire-Mx1 | CP005934.1 |
| Candidatus *Methanoplasma termitum* strain MpT1 | CP010070.1 |
| Candidatus *Methanomethylophilus alvus* Mx1201 | CP004049.1 |
| Candidatus *Syntrophoarchaeum butanivorans* | LYOR00000000.1 |
| Candidatus *Syntrophoarchaeum caldarius* | LYOS00000000.1 |
| *Archaeoglobus profundus* type strain AV18 | CP001857.1 |
| *Archaeoglobus veneficus* SNP6 | CP002588.1 |
| *Archaeoglobus sulfaticallidus* PM70-1 | CP005290.1 |
| *Ferroglobus placidus* DSM 10642 | CP001899.1 |
| *Geoglobus ahangari* strain 234 | CP011267.1 |
| *Geoglobus acetivorans* strain SBH6 | CP009552.1 |
| *Archaeoglobus fulgidus* DSM 8774 | CP006577.1 |
| *Archaeoglobus fulgidus* DSM 4304 | AE000782.1 |
| *Methanofollis liminatans* DSM 4140 | CM001555.1 |
| *Methanoculleus thermophilus* strain CR-1 | BCNX00000000.1 |
| *Methanospirillum hungatei* JF-1 | CP000254.1 |
| *Methanocorpusculum labreanum* Z | CP000559.1 |
| *Methanomicrobium mobile* BP | JOMF00000000.1 |
| *Methanogenium cariaci* JCM 10550 | BBBG00000000.1 |
| *Methanoregula boonei* 6A8 | CP000780.1 |
| *Methanolinea tarda* NOBI-1 | AGIY02000001.1 |
| *Methanocella paludicola* SANAE | AP011532.1 |
| *Methanosaeta harundinacea* 6AC | CP003117.1 |
| *Methanosaeta thermophile* PT | CP000477.1 |
| *Methanosaeta concilii* GP-6 | CP002565.1 |
| *Methanosarcina barkeri* MS | CP009528.1 |
| *Methanosarcina mazei* S-6 | CP009512.1 |
| *Methanosarcina acetivorans* C2A | AE010299. |
| *Methanohalophilus halophilus* ASM188940v1 | CP017921.1 |
| *Methanococcoides burtonii* DSM 6242 | CP000300. |
| *Methanolobus profundi* strain Mob M | FOUJ00000000.1 |
| *Methanohalobium evestigatum* Z-7303 | CP002069.1 |
| *Methanomethylovorans hollandica* DSM 15978 | CP003362.1 |
| *Ca.* Methanohalarchaeum thermophilum HMET1 | ASM191440v1 |
| *Methanonatronarchaeum thermophilum* AMET1 | ASM215391v1 |
| *Ca.* Polytropus marinifundus | GCA_003935005.1 |
| Archaeoglobi archaeon WYZ-LMO1 | ASM434782v1 |
| Archaeoglobi archaeon WYZ-LMO2 | ASM434786v1 |
| Archaeoglobi archaeon WYZ-LMO3 | ASM434784v1 |
| *Candidatus*_Korarchaeota_WYZ-LMO9 | ASM434797v1 |
| *Candidatus* Nezhaarchaeota WYZ-LMO7 | ASM434791v1 |
| *Candidatus* Nezhaarchaeota WYZ-LMO8 | ASM434796v1 |
| Hadesarchaea WYZ-LMO4 | ASM434781v1 |
| Hadesarchaea WYZ-LMO6 | ASM434792v1 |
| Hadesarchaea WYZ-LMO5 | ASM434783v1 |
| *Candidatus* Methanodesulfokores washburnensis NM4 | GCA_004212055 |
| *Candidatus* Methanoliparum thermophilum NM1a | GCA_004212075 |
| *Candidatus* Methanolliviera hydrocarbonicum NM1b | GCA_004212085 |
| Methanophagales archaeon ANME-1-THS | GCA_004212135 |
| Methanophagales archaeon ANME-1a | GCA_003194435 |
| Methanophagales archaeon ANME-1b | FP56147 |
| Methanosarcinales archaeon ANME-2a | GCA_003194445 |
| Methanosarcinales archaeon ANME-2c-GOS | GCA_004211975 |
| Archaeon NM3 | GCA_004212155 |
| *Candidatus* Methanodesulfokores washburnensi MDKW | GCA_003947435 |
| *Candidatus* Helarchaeota ASGARD archaeon Hel_GB_A | GCA_005191415 |
| *Candidatus* Helarchaeota ASGARD archaeon Hel_GB_B | GCA_005191425 |
| Thermoprotei archaeon B75_G16 | GCA_003649565 |
