# Supplementary material for: Genomic and Transcriptomic Evidence Supports Methane Metabolism in Archaeoglobi
Source: mSystems. 2020 Mar 17;5(2):e00651-19. doi: 10.1128/mSystems.00651-19 (PMC7380581; doi:10.1128/mSystems.00651-19)
Supplement: TABLE S3 [file mSystems.00651-19-st003.docx]

**Table S3. Primers for amplification of uniquely organized genes in *Ca*. Methanomixophus MAGs.**

| **Primer pair** | **Strand** | **Sequence (5’-3’)** | **Product length** |
| --- | --- | --- | --- |
| mtrH-FAD primer 2 | Forward | TCCACTGAAGTCGGCAACTC | 1482 |
|  | Reverse | AACCCAACAGACAACACCGT |  |
| SAM-sirA primer 1 | Forward | CTTTTCCACGAAGCGCACAA | 1100 |
|  | Reverse | TAAGGGCTTTCGCAGAGTGG |  |
| SAM-sirA primer 8 | Forward | GGAATGTTCGCACATGCTGG | 1222 |
|  | Reverse | GATGCGAACACTGCCAGAAC |  |
| CP-queC primer 1 | Forward | GACATGGTCTTTCCTCGCCA | 1203 |
|  | Reverse | GTTTTGCTCGTCACGGCTTT |  |
