# Supplementary material for: Genomic and Transcriptomic Evidence Supports Methane Metabolism in Archaeoglobi
Source: mSystems. 2020 Mar 17;5(2):e00651-19. doi: 10.1128/mSystems.00651-19 (PMC7380581; doi:10.1128/mSystems.00651-19)
Supplement: TABLE S5 [file mSystems.00651-19-st005.docx]

**Table S5. Taxonomic classification of Archaeoglobi MAGs using GTDBtk** (25)

| **User_genome** | **Classification** | **aa_percent** | **red_value** |
| --- | --- | --- | --- |
| *Ca*. Methanomixophus hydrogenotrophicum Bin11 | d__Archaea;p__Halobacterota;c__Archaeoglobi;o__Archaeoglobales;f__Archaeoglobaceae;g__;s__ | 84.87 | 0.79726 |
| *Ca*. Methanomixophus hydrogenotrophicum LMO2 | d__Archaea;p__Halobacterota;c__Archaeoglobi;o__Archaeoglobales;f__Archaeoglobaceae;g__;s__ | 95.22 | 0.79538 |
| *Ca*. Methanomixophus hydrogenotrophicum Bin16 | d__Archaea;p__Halobacterota;c__Archaeoglobi;o__Archaeoglobales;f__Archaeoglobaceae;g__;s__ | 93.35 | 0.7952 |
| *Ca*. Methanomixophus hydrogenotrophicum Bin74 | d__Archaea;p__Halobacterota;c__Archaeoglobi;o__Archaeoglobales;f__Archaeoglobaceae;g__;s__ | 93.29 | 0.74863 |
| *Ca*. Methanomixophus dualitatem LMO3 | d__Archaea;p__Halobacterota;c__Archaeoglobi;o__Archaeoglobales;f__Archaeoglobaceae;g__;s__ | 89.15 | 0.79527 |
| *Ca*. Methanomixophus dualitatem LMO1 | d__Archaea;p__Halobacterota;c__Archaeoglobi;o__Archaeoglobales;f__Archaeoglobaceae;g__;s__ | 93.57 | 0.79386 |
