# Supplementary material for: Genomic and Transcriptomic Evidence Supports Methane Metabolism in Archaeoglobi
Source: mSystems. 2020 Mar 17;5(2):e00651-19. doi: 10.1128/mSystems.00651-19 (PMC7380581; doi:10.1128/mSystems.00651-19)
Supplement: TEXT S1 [file mSystems.00651-19-s0001.docx]

**Genomic and transcriptomic evidence supports methane metabolism in Archaeoglobi**

**Supplementary Information**

Yi-Fan Liu^1,4,5^, Jing Chen^1,4^, Livia S. Zaramela^3^, Li-Ying Wang^1,4^, Serge Maurice Mbadinga^1,4^, Zhao-Wei Hou^6^, Xiao-Lin Wu^6*^, Ji-Dong Gu^2^, Karsten Zengler^3*^ and Bo-Zhong Mu^1,4*^

**Correspondence:**

Bo-Zhong Mu, E-mail: bzmu@ecust.edu.cn

Or Karsten Zengler, E-mail: [kzengler@ucsd.edu](mailto:kzengler@ucsd.edu)

**Affiliations:**

^1^ State Key Laboratory of Bioreactor Engineering and School of Chemistry and Molecular Engineering, East China University of Science and Technology, 130 Meilong Road, Shanghai 200237, P.R. China

^2^ School of Biological Sciences, The University of Hong Kong, Pokfulam Road, Hong Kong, PR China.

^3^ Department of Pediatrics, University of California, San Diego, CA 92093, USA.

^4^ Engineering Research Center of MEOR, East China University of Science and Technology, 130 Meilong Road, Shanghai 200237, P.R. China

^5^ Shanghai Institute of Pollution Control and Ecological Security, Shanghai 200092, P.R. China

^6^ Exploration and Development Research Institute of Daqing Oilfield Company Limited, PetroChina, Daqing, Heilongjiang 163712, P. R. China

**Supplementary Methods**

*Genome sequences*

The genome sequences, including nucleotide sequences and protein sequences, used in this study were downloaded from the NCBI database (see Supplementary Table S2).

*Screening metagenomes using Ca. Methanomixophus mcrA sequence*

Amino acid sequence of *mcrA* gene from Bin16 was submitted to Integrated Microbial Genomes Expert Review (IMG-ER) server (1) and searched for homologues using ‘blastp’ function against all available metagenomes prior to May 12, 2018. Metagenomes containing hits with E-value less than 1e-10 were downloaded and processed as described above.

*Comparison of MCR sequence active site conservation and conformation*

The predicted tertiary structure of McrA, McrB, and McrG from the Bin16 genomes was predicted using I-TASSER v5.0 with default parameters (2). C-score of the top models for McrA, McrB, and McrG were 2, 2, and 0.93, respectively. Protein-ligand binding sites were verified with the COACH package available as part of the I-TASSER web service (3). Methanopyrus kandleri crystal structure (PDB ID: 1E6V) was the best fit to the Mcr complex in Bin 16 according to the result from I-TASSER. Individual predicted model of McrA, McrB and McrG were superimposed on the 1E6V model using TM-align (4). For McrA, McrB, and McrG, the TM-score for the alignment to the 1E6V structure were 0.977, 0.983, and 0.927, respectively.

*MAG quality control*

Deviations in GC-content, tetranucleotide signatures and sequencing coverage of scaffolds in Bin16, Bin11 and Bin74 were calculated using RefineM v0.0.23 (5). And scaffolds, identified as outliers, were removed when setting percentile of divergent GC, sequencing coverage and tetranucleotide frequencies to 95. Subsequent manual check was performed using anvio5 (6), briefly, MAG were split as major clusters calculated based on splits coverage and GC content and then merged back if the separated clusters were placed parallel in the same position of a genome tree.

*Proteome comparison*

A bidirectional top-scoring blastp (7) approach with E-value < 10^-5^ was used to identify the pairwise orthologs between two genomes. Then, a matrix of average identity was generated with the percentage of orthologous genes shared between two genomes.

*Comparative analysis of orthologous functions*

A local genome database was constructed mainly composed of isolates genomes and MAGs with more than 70% completeness and less than 5% contamination were also added. Proteins from the genomes were submitted to KEGG for GhostKOALA annotation. The presence/absence of each KO annotation in each genome was used as input for Principal Component Analysis. One-way analysis of variance (ANOVA) were conducted to analyze functional difference between different and differences at the p < 0.05 level (95% confidence interval) were considered statistically significant.

*Sliding window GC and tetranucleotide frequencies*

A custom script (https://github.com/geronimp/window_sequence) from a previous study (8) was used to fragment scaffolds into short sequences of 100 bp length, in a sliding window of 10 bp. Function of ‘scaffold_stats’ in RefineM v0.0.23 (9) was used to calculate the percent GC and tetranucleotide frequency for each fragment. The ‘outliers’ function in RefineM v0.0.23 (9) was used to plot the fragments’ tetranucleotide distance against *Δ* percent GC, as well as the principal component analysis of the sliding window GC content and 4mer profile.

*Primers design*

Selected DNA sequences with unique gene organization in the recovered genomes bins (Bin16, Bin11 and Bin74) were searched against non-redundant database using ‘blastn’ function in NCBI for similar gene arrangement. Sequences that do not have significant similarity with sequences in NCBI database were submitted to Primer-BLAST in NCBI (10), and primers were generated by setting minimal primer melting temperatures (Tm) and maximum primer melting temperatures to 52 °C and 65 °C respectively. The detail information of primers was listed in Table S3.

*Phylogeny of 16S rRNA genes*

16S sequence found in LMO1 which is longer than 1000 (bp) were aligned with full-length reference 16S rRNA gene sequences using MAFFT (iterative refinement methods ‘Q-INS-i’ which considers secondary structure of RNA) (11). Sequences shorter than 1000 bp including 16S rRNA gene sequence from Bin11, Bin74 and LMO3 were inserted into the existing alignment using the function “mafft--addfragments”.Conserved positions of alignments were kept on Gblock Server (<http://molevol.cmima.csic.es/castresana/Gblocks_server.html>) using less stringent selection settings (12). Neighbor-Joining phylogenetic tree was constructed in MEGA 7.0 (13), and bootstrap support values were determined with non-parametric bootstrapping (1000 replicates).

*Screening for missing genes*

To search for *mer* gene and genes related to sulfate reduction, namely, *sat*, *aprA*, *aprB*, *dsrA*, *dsrB*, *qmoA*, *qmoB*, *qmoC*, two methods were used. Firstly, missing gene sequences mentioned above in type strain *Archaeoglobus* *fulgidus* VC-16 were downloaded and tblastx searched against the metagenomes in this study. Hit sequences were extracted and assembled with the same setting in 16S rRNA gene sequence reconstruction using SPAdes v3.7.0 [18]. Contigs were search against ‘nr/nt’ database in GeneBank to find out the most similar sequences and their corresponding similarities. Secondly, all uncultured/environmental sample sequences that are closely related to those missing genes in *Archaeoglobus* species were downloaded from GeneBank. Then, metagenome datasets were mapped to downloaded sequences using Bowtie2 (14)with default parameters. Sequences with mapped reads were search against ‘nr/nt’ database in GeneBank to find out the most similar sequences and their corresponding similarities.

*Clone library construction*

DNA extracted from samples W2 and W9 from Jiangsu oil reservoir (15) were used as templates for PCR amplification, nearly 30 clones were picked for each library for sequencing, and the procedure of PCR amplification and and library construction was modified from (16), which includes: initial denaturation of DNA templates at 95 °C for 5 min, followed by 30 cycles of denaturation at 95 °C for 30 s, annealing at 55 °C for 30 s, elongation at 72 °C for 60 s, and a final elongation step at 72 °C for 10 min.

**Supplementary Results**

*KO Profiles*

To make a comprehensive comparison of the metabolic capabilities of *Ca*. Methanomixophus and publically available archaeal genomes from RefSeq and GenBank, a global analyses of KEGG Orthologous (KO) genes was conducted. The KO profile revealed that the metabolic features of *Ca*. Methanomixophus was most similar to other members of the Archaeoglobi, Methanonatronarchaeia and *Methanomassiliicoccales* but distant from the *Ca.* Verstraetearchaeota (Fig. S12). To search for the genomic features that drive *Ca*. Methanomixophus proximal to *Ca.* P. marinifundus, we compared the common KO orthologs shared between *Ca*. P. marinifundus and *Ca*. Methanomixophus with the KO orthologs in the most closely related *Archaeoglobus* *fulgidus* pan-genomes (including *A*. *fulgidus* DSM8774 and DSM4304). The result revealed that *Ca*. Methanomixophus and *Ca.* P. marinifundus are enriched in genes involved in methane metabolism, coenzyme transport and metabolism and inorganic ion transport and metabolism compared with *A*. *fulgidus* pan-genome, whereas genes associated with heterotrophic metabolisms such as carbohydrate transport and metabolism, amino acid transport and metabolism and energy production and conversion, were streamlined (Table S8). These results indicated a transitional state between autotrophic euryarchaeal methanogens and heterotrophic Archaeoglobi for *Ca*. Methanomixophus.

*Other Potential Metabolic Capacities*

Genes for ADP-dependent glucokinase (*pfkC*) and pyruvate kinase (*pyk*) in the modified Embden-Meyerhof pathway (17) were missing, ruling out the metabolic capability of sugar degradation. Gene for D-lactate dehydrogenase (*dld*), rather than L-lactate dehydrogenase (*lld*) was detected, suggesting a selective utilization of configuration of lactate. In *Archaeoglobus fulgidus* species, *dld* genes are co-located with gene encoding heterodisulfide reductase subunit D (*hdrD*), and this arrangement in gene cluster indicates the potential energy-conserving process coupling lactate oxidation with heterodisulfide reduction (18). However, no *hdrD* gene close to *dld* gene was found in these genomes (Fig. 5), which suggests a different energy-conserving mechanism with lactate utilization. Several genes encoding ABC-type peptide transporter and amino acid transporters (*aroP*, *livFGHKM*, *sepRS*, *sepcysS*) were found, as well as genes encoding peptidases (*pepA*, *pepP*, *map*, *flaK* and *sipW*), aminotransferases (*aspB*, *hisC*, *glmS*, *argD*, *ilvE*, *gabT*), 2-oxoacid:ferredoxin oxidoreductases (*korAB*, *iorAB*, *porABDG* and *vorC*) and aldehyde-ferredoxin oxidoreductases (*aor*) (19, 20), indicating that *Ca*. Methanomixophus are capable of importing and degrading peptides step wisely to amino acids, then to 2-keto acids and finally to acetyl-CoA (21) (Fig. 5).

Genes of *Acd* which encode acetyl-CoA synthetase (ADP-forming) were found in these genomes, unraveling an alternative ATP generating pathway by acetate formation, or ATP-dependent acetate utilization (22). This new genus also possess genes encoding aldehyde dehydrogenases (*aldh*) and alcohol dehydrogenases (*adhP*). Therefore, the resulting acetyl-CoA generated form heterotrophic metabolism of fatty acids, peptides and lactates could either be processed to acetate with concomitant production of ATP via an acetyl-CoA synthetase (ADP-forming) or be fermented into ethanol by aldehyde and alcohol dehydrogenases. The various fermentative strategies and broad substrate spectrum used by this new clade suggest that it is adapted to a fluctuating availability of organic compounds in extreme environments.

**References**

1. Markowitz VM, Chen IMA, Chu K, Szeto E, Palaniappan K, Pillay M, Ratner A, Huang J, Pagani I, Tringe S, Huntemann M, Billis K, Varghese N, Tennessen K, Mavromatis K, Pati A, Ivanova NN, Kyrpides NC. 2014. IMG/M 4 version of the integrated metagenome comparative analysis system. Nucleic Acids Res 42:568–573.

2. Yang J, Zhang Y. 2015. I-TASSER server: new development for protein structure and function predictions. Nucleic Acids Res 43:W174–W181.

3. Yang J, Roy A, Zhang Y. 2013. Protein-ligand binding site recognition using complementary binding-specific substructure comparison and sequence profile alignment. Bioinformatics 29:2588–2595.

4. Zhang Y, Skolnick J. 2005. TM-align: A protein structure alignment algorithm based on the TM-score. Nucleic Acids Res 33:2302–2309.

5. Parks DH, Imelfort M, Skennerton CT, Hugenholtz P, Tyson GW. 2015. CheckM : assessing the quality of microbial genomes recovered from isolates , single cells , and metagenomes. Genome Res 25:1043–1055.

6. Eren AM, Esen ÖC, Quince C, Vineis JH, Morrison HG, Sogin ML, Delmont TO. 2015. Anvi’o: an advanced analysis and visualization platform for ‘omics data. PeerJ 3:e1319.

7. Altschul SF, Madden TL, Schäffer AA, Zhang J, Zhang Z, Miller W, Lipman DJ. 1997. Gapped BLAST and PS I-BLAST: a new generation of protein database search programs. Nucleic Acids Res 25:3389–3402.

8. Boyd JA, Jungbluth SP, Leu AO, Evans PN, Woodcroft BJ, Chadwick GL, Orphan VJ, Amend JP, Rappé MS, Tyson GW, Tyson GW. 2019. Divergent methyl-coenzyme M reductase genes in a deep-subsea floor Archaeoglobi. ISME J 13:1269–1279.

9. Parks DH, Rinke C, Chuvochina M, Chaumeil PA, Woodcroft BJ, Evans PN, Hugenholtz P, Tyson GW. 2017. Recovery of nearly 8,000 metagenome-assembled genomes substantially expands the tree of life. Nat Microbiol 2:1533–1542.

10. Ye J, Coulouris G, Zaretskaya I, Cutcutache I, Rozen S, Madden TL. 2012. Primer-BLAST: A tool to design target-specific primers for polymerase chain reaction. BMC Bioinformatics 13:134.

11. Yamada KD, Tomii K, Katoh K. 2016. Application of the MAFFT sequence alignment program to large data - Reexamination of the usefulness of chained guide trees. Bioinformatics 32:3246–3251.

12. Talavera G, Castresana J. 2007. Improvement of phylogenies after removing divergent and ambiguously aligned blocks from protein sequence alignments. Syst Biol 56:564–577.

13. Kumar S, Stecher G, Tamura K. 2016. MEGA7: Molecular Evolutionary Genetics Analysis version 7.0 for bigger datasets. Mol Biol Evol 33:1870–1874.

14. Langmead B, Salzberg SL. 2012. Fast gapped-read alignment with Bowtie 2. Nat Methods 9:357–359.

15. Liu Y-F, Galzerani DD, Mbadinga SM, Zaramela LS, Gu J-D, Mu B-Z, Zengler K. 2018. Metabolic capability and in situ activity of microorganisms in an oil reservoir. Microbiome 6:5.

16. Liu YF, Mbadinga SM, Gu JD, Mu BZ. 2017. Type II chaperonin gene as a complementary barcode for 16S rRNA gene in study of Archaea diversity of petroleum reservoirs. Int Biodeterior Biodegrad 123:113–120.

17. Labes A, Schönheit P. 2001. Sugar utilization in the hyperthermophilic, sulfate-reducing archaeon Archaeoglobus fulgidus strain 7324: Starch degradation to acetate and CO2 via a modified Embden-Meyerhof pathway and acetyl-CoA synthetase (ADP-forming). Arch Microbiol 176:329–338.

18. Hocking WP, Stokke R, Roalkvam I, Steen IH. 2014. Identification of key components in the energy metabolism of the hyperthermophilic sulfate-reducing archaeon Archaeoglobus fulgidus by transcriptome analyses. Front Microbiol 5:1–20.

19. Schut GJ, Menon AL, Adams MWW. 2001. 2-Keto acid oxidoreductases from Pyrococcus furiosus and Thermococcus litoralis. Methods Enzymol 331:144–158.

20. Lloyd KG, Schreiber L, Petersen DG, Kjeldsen KU, Lever MA, Steen AD, Stepanauskas R, Richter M, Kleindienst S, Lenk S, Schramm A, Jørgensen BB. 2013. Predominant archaea in marine sediments degrade detrital proteins. Nature 496:215–218.

21. Seitz KW, Lazar CS, Hinrichs K-U, Teske AP, Baker BJ. 2016. Genomic reconstruction of a novel, deeply branched sediment archaeal phylum with pathways for acetogenesis and sulfur reduction. ISME J 10:1–10.

22. Musfeldt M, Schönheit P. 2002. Novel type of ADP-forming acetyl coenzyme A synthetase in hyperthermophilic Archaea: Heterologous expression and characterization of isoenzymes from the sulfate reducer Archaeoglobus fulgidus and the methanogen Methanococcus jannaschii. J Bacteriol 184:636–644.
