# Supplementary material for: Genomic and Transcriptomic Evidence Supports Methane Metabolism in Archaeoglobi
Source: mSystems. 2020 Mar 17;5(2):e00651-19. doi: 10.1128/mSystems.00651-19 (PMC7380581; doi:10.1128/mSystems.00651-19)
Supplement: TEXT S2 [file mSystems.00651-19-s0002.docx]

>Bin11_1

agggatgttcattcctatgaaacccagctcagcagtcttcttgaacagctcgaacgggaatttctcctcccgatcgcactcctctgcatatttcggaaactccttttcagcgaactctcttgcagcctctttaatcgctttctgttcctccgtcaggagaaactccgacaacataccatatcacccttcagcactttttttaacagtatttaaagtttgctaaattttaaataattttttatcataagaaaaactcttctcacattttaatcttttccgtggctcttgcgtaaagagttcagcggaagagtggggttaaggttataaggtctctttcttagcgtcaccatggaagtgattccaattgcctacgattcaatgggagtcagaagcatggcaacttttgttagaacgaaagatctcgcaataacgatcgatccaagcgtaagcctagcgcccgtgagatacggactgccgccccacagacttgaaatcgagagaatgaaggagaagtggggagagataagggattttgtaagcagaagcgatttgatagcgatcacacactaccactacgaccaccacaatcctgaagaagttgaaattttcgatggaaaaaatctcttgctgaagaacccaagggagaagataaacagaagtcagcttggaagagcgagttatttcctcgagaagttgaaggaaatagacgtggagatcgatttctgcgacgggaagctctatgagtttggaaatacggttattgagttctccaaaccagtatttcatggagtaaaccagaagctcggcttcgtcgtcgagatttttatttcagagggtgatgacagttttgtcttctcttcagacgtggaggggccaattcacgaggatcaggctgcatttataatcgaaaaagagccagaggttgcttttatcgatggaccgatgagctacatgctcggctaccgcttttcagcggattcgttcagtaaagccattgaaaacatcaggaaaattctggaggtttcgaaaacagtggtgttggaccatcaccttttaagagatctcaaatggagggaaaagctgacagagctctttaactatgccgaacagctgggagtcaagcttcaatctgcttccgaatttctcggaaaacctgaagagcttttggaggcaagaagaaaagagctgtatcaggaatttcactgatcagttcactgctgtctcgatgcaggacttgaggtctccaaatgcaacttcaattccccttttcttcggcaaatactcaagagcctttccggagttaaccatgacgcaatcgaatctttcgctcgctggagagacgaccatgcaggtatccttgaataccttagcgccgagttcttccagtttgcctattatctctttgctatcctcagcaacgctgcgagaagtgaagatccacagctctcttttcaccctccttcctgcaatcagtctcagaatttcctttagctcctcttttgagcagtgagggcagccaattgcaattagctgtgcttcacagctcttttcaattttaccctctatctcgacagtctcgcttggcttttcgaagtctctccactcaggcgtaatttcttcggcatggaacatcgcgatattcccgctggcagccagtgaagcggataggagctttaactcgtccatcgatacccttctgtcgaatatgaaatagggtatctctccaggaagttccagtccagctttgtagcccacgatcgaaaggtccccagttgccttcacgaccaccgtgggtgccctgttctctttaatgtggagcccgtaatagggtgtccggcccgtgattgccgaagccagtgcgctgattccactctctctgtttgttcttgccccgataacggagttcgcataaaccacggcagagctttcggcccaggccagatgttcgccgaatttcggcttttggacgtagtaaggagtgcaggtcagtgttatttcgacttcaagatttttaaaaatctcaattatctccatttgctttctgtaaaactcttccgcgattcccatttccctccatctctccaagtccattcctgctgggttcagagtcgtcctcaccgacaccctgccctttatcgttctcagccattcaagaccctcatcgcctatgttgtcgtagctaaccccggagatgtgggcagaggagatctttacgagtctttcagcatcaaaaaccctcgcgagtgcgaggacgattttcatgcagtctcttgtttcttcattttcaagcatctcctcttcttccttagtaagcattttccacctcttttttcagccactcgggtagagtaaatgccgagaggtgtacttcaggattataatgtcgcgttttaccgctgatctttttaaaattccttttaacgtatctgaagtcatagtctccagcaagaatgaaactccagagagccatcgggtacgtcgggatagttgccagaaaaactgctctgtttttcagcaccgatgaattttccagcatcttcctgaagtattccttctgaatcagcggagactgggactgggagcagaagaccctgcacttttccctgcaaagttcgaagaagtctcttccgaacaggggatcactcacaccgactggatccgtgctgtcgactattattacgtcaaaggcctcgcaatgctccagaaattgtcttccgtcttctatcacaatttccactctcggatcctcaaacgccccgccatctatgcccagatgctttttgcacagctctattacgttccggtctatttcgacgagaacggctctctccacgtcatgctttaaaacctctctcagcgctccaccatctccaccgccaatgaccagaatttttttcgcctttctacagctattaagcggcacatggacgagcatctcgtggtaaaaacattcatcgaactctgtgagctggatcttgccgtctataacgagcatttttccgaatctctcggtctcatagacctctatcctctggatcccctcagcctccgctatctttcccttaacagatatcatcagccctgaacaatcgtatctctcgacgaaccattccatgaaaagagtccgagaagtatatataacaagtttgccttctcttatccgtgatttatcttggtggagaggcggaggtaaggataggggatatcgttgtaaagaagagaaaaccaaagcgctacaggctgaaggaaatcgatgaaatgctcagaataaaacggacaagagccgaggcgaagctcatatcaatggcgaggcgcatcggggtgccgaccccaatcatattagacgttgaggaggacacgatagtgatggagagaatctacggggttccagtaaaggactgcatgagcgaagatatcagcagggaaatcggaaggcttgtcgcgaaactgcactcggccaacataatccatggcgacataacgccgatgaatttgatccgcagcaacggaaagatctactttgtcgattttgggctcgcattcattgacagcagaatcgaggctaagggagtcgacgtgcacgtttacttcgaggcagttaaggctggatttgaaaactgggagagtttgagagaggcgttcgttcagggttacctcgaatccgggagcgaggaagtcataagaagggcaagggaaattgaggagaggggcagatacgtagatagaagctgaaatgaaggtcaaataatcctttttgcactttctaagtactcctttattccctctattgcctctgcgagtgtcttcctgttatcgtagttcgaaacgatgctctcgagttcatctctgctgtgatcccgcaattcccttgcaaattccaccattttcggaatagccctagttacgttgtccacaatcgtaacgtctgccattctcgcagttcttgagagtggattcaggtctatcgctatgaccttctttccatgccttttcagtatctcgcacctgtctccatcttctaaggggacgagaacgacatccgcgatgaatattcccctccggtcaacgattctcctcgcggagctcaaaccctcaagctcggcatcccccgcacaaagggctgagacgccgaatttcagcagatggtcggcaatcctgttcaccctttccctgctgtagtgaaagacgttcacttcgactcgtgcattcacgatctctgcgagttctccgatctcctttggtacgagaacggccgtatttccatttacactgatcacaggattttttgcaagcagcatcatcgccacagccaccttctcagccttcagggcaaattctctcgttctctccccgaggatgtagtcaaaagcctcgcccctgccgtgggctattaacccctctaaggctgtgatcccctctctcactccttcgacgagcttttcccttgtcacgagggaatcataccttgggtgatccttcggaatcatatctaccctcgagaaattcaccgagctcctttaaagagttaaaaacgtagtcagcgtggattttaaagctttctatcatctccctgttcctttcgtgcacgataagagcagttttaactccagcacccttcccagacaggaggtcgaacagatagtcgccaacaaccagtgcggagctcggttcaacgttaaacttttttagaatcattcttatcggctctggcgaaggtttaggatttgcatcctccctgctcacgacgaagtcgaactcaagtccaaacttctttgagattatctccacgctttttctgctgttcctcgtcacgactccgtaaatgatttttctctctctgagaaattgaagtaatttctcggcgtaaaacgcaagccttgcattttctgcacttcttagctcgtagctttcgagaatttccagcatcttcctcctcttaaactcgtctttctccgataaaatgctttccagcacgaatctatctccgatccctaaaactgcccttatttcttcgaagggtatgttaaattcgaccaaggtgccatcgaggtcaaaagctataagtttgaacatactccctgaacctccttgcttcctgtcttggattatctgcctcgtaaatgctcctgccaactattattccatctgcatacctgagagactccaaactgccaccctgggctccaacgccagggcagagaattcgcatctcgccggcagtttctcttatcgcccttagcctttcgagcctcgtggctggagcgatgaggccatgacatccgatctccttcgcttttttggcgatctcaagcgagaacttactcatgaactcctcgccgcccgggctgctgagctcggtcactacgtaaacttcgccgttaaaacgttttgctacgttcacaaccgatttcaaggagtcgctccccacgaaaccgtgagctataactgcctctgcgtcgttacggaaggctaaatctgcgatcttcgagctaacgtgcggaacatccgcgatttttaaatctgcaattactggcttcatttctgagagttctgagatgattctgatgcctgaggacagtataagcggatagtttaccttgaacctgtcaacgagatcgcagacctcttcggctatcctcagcgaaattcttctctcctctacatctaaggccagaatcagctccttcacgaattcagttctttcggtcgaaaataaatgttttccaaaaacgatttttagcaaagcgatgattcgatgtatggatttacctttgctgtatcacaggcttggaaagaactcctggaaaattctggatgccattttcaggaatctctggaattacaaatacgtgcccctgaacgtaatcagcaatttttcaagggttaatgaggaaaa

>Bin11_2

ccatatgttgtcaagggcatggacttctctttcagcggtatggtaaccgcggcgcaaaggctcaaaggaaaggcgagaattgaggacatagccttcagctttcaggagactgcttttgccatgctcacagaggtgacggagagggctcttgcctacttaggctacgatgaagtgctcctcgtcggaggagttggagtcaacaaaaggcttcagagaatgctcggcatcatgtgcgacgatcgcggtgcgaggctttacgctccgccggcagagctaatgggagacaatggcgcaatgatagcatacacgggtctgctgatgctgaaacacggctacagcacacccttagaggaatcattcgtaaggccaaacttcaggatcgaggaagtcgagataagatggtaaaatgctcagagcggagctgcacgttcactctaacttcagcgacggaagagacagtgttgagaaaattttgagggcggcgatggagaagagaatagacgttctctccattacggaccacgacacgatagatggctcgctcagcgcaatggagatcgttgcagccgaaaagcttccggtaatcgtaatcccgggaatggagatctcgacgagaagcggccatcttctcgcattcgggatttcaaaaagagttgagaagggaactggaatggcggaggcctgcgaagccgtaagggctctcggaggaatagcagtcctcgcgcatcccttcgacttcctcagaaaggggtctgtgagaaagagggacttcgctcttgtggactgcgtcgaggttttcaatgctaaaagctacttcaacttccttgcaagaaggtatgctgagaaatacgggaagagtggaatcggaggtagcgatgcgcacacggcgaaggagattgggatcgtgataaactatatcgagagtcctgataagaaatcgatcctcaatgcattttacgacggaagaaggcagacgatcagggagagactatcctttctactatctcggataggtccaagaccctaatcctgtttccaccaaccctcgaaaggttgtactcgcagaacgggcatgaggttattatgacattcgtccccgtttccaaggcttccctgatcctttcttcggcaattgccatggc

>Bin11_3

ggaattgagatctctgagaggttcacaaccgaaatcctggaggaaatctcgagaaggagaagagaggttcgcgaaggcttttgcatccgctatctcgtcatggacgacgctgtctctacaagcccaatagcaaccgttctttcccgctacttgatgcccgataaaccattgatagttttgaacataaaaccggatggaaaagtaaaggtttcggcgagagcgacggaaaagatcgcccggagactggatctggctgaggtcatgcgtttgtctgccttgaaagtaaacggcgtaggcggtgggcacagagtggcagcaggagcaaacattgataaagacagggtggaagaattcttaaaggaggtcgacaggctttgctgtgctatgctcgcttagaaatagaaatcgaggatgccgataaaattgcaagagccctgagggttgacgacccgcactggtgcagctgtcagtttgaggatgggaaaatattaatcgagataagggcgagaaagctgagctcactgctctatgcaatagacgactatctaatgcacttaaggatgtgcgaaaggatatgatgctctcgttcgaggattacatcgagctcagaagaaatctgaacagaatttccgacttcgataggttcaaatttccgaggggcattctacacgcaatactgatgcagaaaaaggtcgaaagcgtcaaaaggaagtatcatatcttctctggcaggaccaaggagatcctacagttctggaaggagagaaagaggtttcctgagtggctgacactgaccccggttttaaaggtcaggttactgctcaagggaatggatttcacgacgaagcagataaacaatgcgctcagaaatccgaacgagctcgaagatgagctttcgaaggttgtctacaatgcggtctcaagggattttgtctactctcctatagcggcaaaacttcagggcgtgctcggaaagatcggagaaaggatcatcgaggaaaagctcaaagacctggggatagagttcaaaaccgaaaaggatctaaaaacgcagaagacacccgatttcttttttgaggagccactggaactttttggccggaagataaggtggatcgagagcaaggctctttttgcagacctgagaacctatgagctttacagaaaaaagcagatctcgaaatatcaggagcttttcggcgatgggattgttgtttactggcggggttgcattaaagggctacctgtaagcgatggaagcgaatttgatggagatttgaaaagaaaacttctcgaaatgagtttatttttctcaaaatcagaagaggtggacggagatccgttgaagcttgccgaagaattcatcggagactacataacgagagatacgttcccttacaaccgcgaagccgttagaatcctgaggaacatgggttttagggttttattcagggaaacctaagagtcgatccaaagttcggggaaaggtttaaatgtactaaagaaaaattgtatagattgcagtagatttgtcgcaattgggggtataaaaatggaaagaaagaccgcaattatctgtttttcggtatttttagctggagctctattcttgggctgtgcacaggctccttccgaaacaacgcccgcaacaaccactccaatacagacttcgaagacagaggaaaaggtaacctgtccagtagggtctttcataaggacgccagaaggtgaattcaaggttacggggatcgagaagcagacagttgctggaaaaagtatggaaatgtgctgtatggagttctcctctggagagatcaagcaaaaattttgccacgatatgttgaaagctgagttaggtatgtgggggtacagaaacgctattttctggaccaccgacgatgaaacaatgaaattctacaaagcggctgagggattcgagctggatggaaaatactgcctacagaactacgatatttctggaaaggctaaagaaaaggtctgcatgtacaaagagggttcacagacatgtatgattatatacgatgaagagggtaaagtcgagatggaaggatgccagtagctttatttttttaaccaaacttctccaagcatcttcgaacaatcgacaaggttttttagtcgaagcagaagttaaactgtgataagcctgagcaaattggtcggcggaacttcaactgtttcgaaagagctaacatacgaaggcgacgagagttttattcccaaaaagctactggatttctcctcaaagctcgttccaatcgtggtttggaacataacaagtagatgcaacctgaaatgcatgcactgctatgcaaaggcaggagttaagggcagggagctctccaccgagcagtgcaagaagatcatcgcagatctgtcttctttcaaggtccctctgatcttgttcagcggtggcgaacctctgcttcggaatgacatttttgaacttgccgagttcgccaaaaagaggaggatgaaggtcgttctctcaacaaacggcacgctgatagaggacgacatcgccgatcagctgagggttttcgattacgttggaataagcttggacggcattaaaacgcacgactccttccggggtttgaagggggcgtttgaagcttcacttaggggtctgaaaatagcaaacgaagttgtattgacaggtataaggttcacgctgacaaaatacaactttttggagctttccgacctcgttaatctggcaagagagctcgaaataccgagattctgcatgtatcatctggttccctcgggaaatgccacgtttgaagacgacgtcgacaacctttcgagaaggagggctgtggattacctcatcggagaggccaaaaaggagggaatggagattctcacagtcgacaatccctccgacggaatatacctctaccagaggactaaagacgagaagatactggaatttctcagatacaggggtggagacagcacgggcattagacttgtatgcatcgactccgaagggaacgtgcatccaaaccagttctggatggattacacagcaggaaatctcctgaagcaggatttcgggactatctggtatggagacccgctttttaaaatgttaagagaaaaagaaaaatatcttgaaggccagtgtggaatctgcaaatacaagcatctttgcggaggattcagagttagggcctacagaaagggaaatctctggggatgggacccgagctgctacgtttcgattgtatgataatgatgattgtaataacagatttttacatataaggaaaaggtttatatattttgacatagatccattattatgagctcgctgatggacatgagcgaaatagtagccttagtggcattagctatagctttactcgcacttcagtttatcttcttggcctaattttttaaaaatagtctacttccccaaattcctactgaatctggaattccagaaggtctctcgccacttccacgttatcgaacacttccattgcctccctgaggagcagctctggattcttagaatacctcgcacttacgtgcgtgagaaccagctttccgacgtttgccctcttcgccacctccgccgcctcctttgctgtagagtgtcctgtttcgatagcccagtccttcttctcttcagagaacgatgcgtcatggatgagcagattcgcattcattgcaacttctacagttttctcgcagggtctcgtgtctcccgtgtaaacgagctttctccctctcttaacctctccgagaaccatgtctggagtgatcagctctcccttccagtaaacgctttcgccaccctttaaccttgcatagagtggccccgggggaattccaagagctaaagccttctctctgtcaaattttcttgtatcctcctcgatcagggcataacctacgctcggaactatgtgctccgttttgaaaaccctgaccttgaagcctctaaaagctatctcatccccgtccctcgcccttttaaccgctatggggtagtcaagcttgtcgtagccgaatatttcgaatagggccctgagcatctccggatttggggtgtagaaattgagttccctcttcctgccgttaagtgatagagtctctatcagaccaaaaacccctatgaagtgatccgtgtgcatgtgggttatgaaaacgtggtcaagattcctgaaaccgagctttgcagagatgatctgtctctgcgttccttcaccgcagtcaaaaaggaagcgaaaatttgaatgctgaacaaaaatcgcagaagtgttccggtctaaggaaggaattgtacctgcggtgccgagaaaaacgaccctgaatagcatcaggattccaaaacgatctcgatattcacatccctcggcacctgaattctcattatctgcctcaaagtcctctcatccgccgcaatgtcgatgagcctcttgtgaacccttatctcccagtgatcccaggtattgctcccctcaccatccggggcctttctcgaggggacgaccagtcgcttcgttggcaacggtattggcccgctcatttcaacgccagttttcagcacgatttcctttatctgtgcgcagattctgtcgagatccgctgggttcagactcgatagctttatcctggcctttggaccctttatagccataaaaccaccaaaaaaatggggtttattttcttggagttacatccagaaccattccagctccaaccgtcatgcccatgtctctcactgcaaacctgccaagtggcggtatgtccttgaccctctcgataaccatcggcttcgttggctcaagcttcacaacagccgcatcgcctgtcttcaggaactgcggattctcctccttcgcctgtcctgttctcggatcgatcttcttgatcagctgtacgaatttgcatgcaacctgcgcggtgtgcgcatgaacaactggtgtgtagccaaccgttatcgcggtgggatgctgcagtaccacgatctgagccgtgaagtccttgaccaccgttggaggattcgtcggatgccctgcgacatctcctctcttgaggtctttcttcgcaacgcctctcacgttaaagcctatgttgtctcctgggtacgcctcctgaatgggttcatggtgcatttcaatgctcttgacttctccgctaacccctgcaggctcaaatacgaccttgtcacctgtcttaagcacgccactctcaaccctgccaacaggcacggtacctacgccgctgatcgagtaaacgtcctgaatcggaattctcaagggcttatctaccagcttttcaggaggcttcagcaagttaaaggcttcaagcagagtgggtccgttgtaccatggcatcttctggcttttcttggttatgttctccccgtagtaggacgatgctggaatgaagggtatttcctcgaccttgtatccaacactcctcaggactttcgcaagagcttcctttgctgcttcgtattccttcttctcgaaattcaccctgtccatcttgtttatgaggcatatcagctggttaatgccgagagtccttgccaagaatatgtgctcccttgtctgtggctgtaccttgtcgacgacgtccatgacgagaactgcagcatcagcctgcgatgcgccggtgatcatgttcttgatgaagtccctgtggcccgggcagtccacgatcgttatgtagtacttatctgtctggaactttctgtgtgcaacgtctatcgtcagtcctctttctctctcctccttgagcccatccataatccaggcaaactcgaaggtcgccttacctttctcctgagcctcctttctgaatttctctagtatatgctccggaatttctcccatttcgtacagcagtctgcctattgtcgtgctctttccgtgatccacgtgccctatgaaggcaacattgatgtgctccttctccttcggcatactcatcacctaccgtattttgatataatccactttcaagagtgcatataaagctttcacatatcagatgaacatcctcgcaatcctcagggattcgaggatgctctcctttgcgttgccctccacaacttcttcgtgcccgggataaagatttacaacatcaagcctggaaagcctttcgatcgattctaagagcaatcttccatctcctccgggaaggtcgtatctaccaaagctcccgtaggcgaacacagtgtcaccgctgaagagccacttcttttctggctcgtaaaagcatacgcttcccggagagtgaccgggggtgtggatgacttcaaggctgatctccccaagctcaaaaacttcaccgcctttaagcaagatgtcgggctcgaaggctttaaatctaattccaaagactggagcgaatttttgcagtcttaaaagcctcatttcttcctcgtggattgcaatccttgcaattcccttaaagtatccagccgccgccgcatggtcatagtgggagtgtgtgagaaaaacgtagtcgagctctttcggatcgatatatctttttatcgcccttaaaatgaactccgggtcgcctccaacgtctattatggcttttttttcatcgagcaaaaggtagcagttcgcggcaagcggaggggcaacgatccttgtaaccctcataggagctccatgtcctcgatttcaacgctttcgacgccctcgatcttccctacttcttctacaaaacgatctgagattccttcagtgtcgggcattacggcaaggactaaaagagccttcaatccgaatgcaatcggtctaacggcaatatcccgaatctcgacggcttcgagattaagccttaaaattttctccctgatcgattccagatcgacatctacatccgcgggcataaccctcagctttaaaaagactctgcccattctcaccacctcatggtccaacgaatccgcatgagcatctgtacttattcgaaagccttctgcatctcttgcagcgatttatcgtttctccacaattcggacacgggaatgaaacgtgttctgcaccgacaagcattgccccgcagcttatgcaccttgcgatctccactcaatcaccccctacggacgtgcccttcgctctccctctcaatatataattttttagctcctctggggagcagacaaaaagttccaaattattttttttgagcaaaaagggagtatagcgatcaaccaagtctccagcttcgaaagcccctgccctctcaacgatttccccgttcctgataacgccacccaaagccgtcaccttaacgactctcctataccctattttcgcggcaatccagacggctattgcgtctgaggtcacgtcccaagaatgagggagttcgtcaaactttctaaggagcctgtatggcagtacgatgaattttcccctcctttcaaagaaattaaattcctcgggttcggctatttcaaagccatatttctttccgaattcgtgcatgtagtatccgtagatgctcatcgcaagaattgccatccagtgcgacgcttcttcgccaggattaagttccctgacgagatctgcaaagatccatcctccgggaattattttcacatcctcaagatttcttagagcgtcgaaaacaatctcgagtctatctgctacgcttcctccaactttaacgatcatatcttcctcatctcatagaactcccaccacttcctcagctcgtactttcccacgatctcgtagccttcaagacatccgaaatcgcgctgcacgacaaaaacatagtcgtatccctgcgggcaagagtttgtgaatagcaaattgcccttatacttatccacttcatgcctgagcatccacgggagaggccagtagtggccatcgactgcgaagactgcaaccttgtttccctcttgcaatagctccctgattctatccgacatctcaaccgcaccccactgcgtctgcacgtagatcagcggctgctcgacgttgttgaagtccacataagtcacgtgcaaggaaatgaatgtggtggcaacgagaccgaaaacgtataaaagcctgaaatcttttctggagaaagcctcctttccgatgaagactgctgcgaggaaagcaaggggagttgcgagatggacgacaagccaggaagttttataggacatggcgttgtagaaaataaatgctattagcgtccagcagagtgcgaagacctcaataaggctcagccctcttaacttcctcctaattgcaggaatcgatgcaactgcgagggcaacggggagaaactcgtaacgtaggagaatgttgaagaagtagtataatggcttgtcgtgtggcgattctatgccctgagagagccagtatggaagggagcgatcgatgaacctctggatccattcccaacagaacatgacttcaaaccagttcctaccatgtaaggcgtaagcaaaggccgaggagtagaggaaggaactgaaaaggacgaagagaatggctgaagggatcagcgcgctgatgttgaccttaa

>Bin11_4

ctgaatcctgctcccatctggcatcgtcgcatccaccatcggctccgcaatgcttatatgcttcccgcacttctgcgccagatttatcacgaaggagtcaagctcatcctcatcctcgaagaccacgttcgttctcaggttagtatacctgcggtgaaacacgtagatcggtatcttgtatccgttgcatgagatgtcctctatgttcgggtcgtacatgagcggagtgatgcggtcgtagaggatggttctcttcattatgtggtagagaattcgtggataattcgagacgtccagttctattctgaagtcgtcgagtatcttgttcaccttattcctgagtgcagagaacttgtcccagatctcctggggttttatcgtgtaatactcgagcttcttcacgatatcccttacgagagaatcttcttcctgacttagagggggctcatttatcgcgtaagcatattcgttttcctcgtcgctgtagaggatatgagcgcttgcaaaaatgaatctcgagtatatgggatatttttcaacctcataccatccttcagggggtttaaattcctgcattaacttcttggaaagattttttgaagcttcttcgtcgagccactcgccccttgcactctttcttatgacttcaaaaactcccctcaattccttagttgttctcggcgcccccttgctgatcctgaatcttgtctgcatggccgatcttaaactttaaagaataaaagatttttcacggcacaggcacgattcagaagacaaaaattttatttacactttcggaaaatcgacctgcatggcaagaaaaccagcgaggatgtggaggagaattaagagaccttacacgagggtcgagtacatagacggcgctccggggacgaggataaagatgttcgacatgggaaatctgcaggctgactttccagtcatgctgaccctcgttgccaaggacgcggttcagataagggacaacgcattagaggctgcgagagtgattgcgaacaagtacatcgcaaagtatgctggatcgagcaactacaagctaaaggtaaggattttcccccaccatgttctcagggagcacaagatggccgttggagctggagctgacagagtc

>Bin11_5

caagctctccgatcgtcgagatacctccgtttaccccgggccttactttcctgaaattcgtgatgaggtaaacgttctttgctgtctttccgatttcgctggccatcgtatctccgagtgctgtggcgactgaggctacgaatgccatggcaaagatttcttctttagtcatcccgtagttcatggcgaaaaaaagaggtgccaggctgtttccaaagacgttcgaaaatcccctcgcaccgccagagggttccgctatgcccaaattttccttgagagaatacctgtatttcgttaccgctgagccggaaagatagaagaagaggagcacggcgaagaagttcaaattcgtgaacaggatcgttatggttccaactagcgttgcgctcatcagtccgctctcgtctgcgattccagccttgagtgccatgaggcttaggacaaaagaaagggcaaaagctattgccagagcctcgactgaggctgaggggatgtatatcttgaagatcgcaaaaacggttgccaaggctattaggaggagcaccctcctgtccgattcggttttcacagtttccattaaagccgcagtaagcccgccagcgagggatatgaaaatgatgtttgaggccgggatgaaaatgccgttcatcaatgagtaaagggaaaagaagaggactccggcacacgtataaattggaatatcccaaatagggcttctcttcagctcatggaagaatgcaacaaaaatggacgcatatattatttcctttgccatgaggcacgcaagggcagaaaggattgtggctgcgaaaagaaaattgaagtattcgccgtttctccagaatacgtatacggctgtaagagtaaaaaagagtgcaaatattatcctcggatctgagattgggtgaggcgaaatgccggcaaggctcacaagcgaggcgatcgcaggaggaagcattgaaaagggttaagtattcagacatttaaattttgacgtgctccgattgctataccagctcatgctcgagcggaagataaaaaaagtgccgagacacataacgcttatctgttccgaggtaaatgagaatttcaagaggtttttggaatggtgcaggaagttcgggattcgggaagtaactgtctgcacgggttcttttcctttaaagatcgaaaacgttagattgaattttatagaggaagaccgctcatacggcacaggagagggagaccttaccatcaacgtggtcaggagcgaaggcagaaaggagatcgtggctgcgataagagagatcgcgagaaaggtagtgaatggagagctgagaagcgaggaaatcgacgaaaaaacattcgagtccttcttgaaaattagatcccagccggacatgataataaaagcgggctcggaggtcccggaattcctgctttggcagagtatttacagcgagctctacttcacggacatagactggaacaccttgagatatgtcgactttctgagaatcctcagggagtatcagaagagggagagaaggtatggtcgataatctcaggattgttagggtcatagccgactaccagttcggaaaaaatgcaggaattgcgctttttccagagacatgcacgtttcttttctcaaaaaatggaaaaataaggcagattcttgatggtgggaaaagaatagctacccttaaggcagattcaggattgcttaccctaagcatcgagggggctaggagacttcacaatgctttcccatttcccaggctgagagtcgtggtgatgaacgaggtctcgcagttcatcgcaagcgggaagagcgttttcgcaaagcatgtagttgccgtggatgagaggataagggcaaatgacgaggtgcttgtcgtaaacgaaaaagatgagttgctcgcaacgggaaaagcgttgctttccgcttttgaaatgctcgaagtcaagaaaggcgttgccgtgaagaccagacaggggtcggaatgaaaatcgacaggaaaaggggtttgcttctgattgagagcttttcaaacctgaaaaagcctgtaaggtttgaaggctttgtctccctcggcgtaggctgtaaagtttccgaaatctatgcgagggagatagtaactgcaaagggttgcgttctcgggaagatcatctgtgatcgaatctttctcggcgcattttccacttttaactcgatcgaggcggaagaggcgataatttcgaactcgtgcgagggtgaaaggatcgtcgccaaagccgtcagaattggatgcaattgcgatatctctgaaataaaggccgaactgcttgaaatgaggggtatttccagggttcagaggatggaagctttaaaaataaggtgtatggactttatataaaacctttcctgataaaatccaggggtatcgcttttctgtggactgcggtcgaaatcaggactccggagcaacccaattccttaagcttctccaaatcctcgatcccacttactccgcctcccaagtaaaccggatgctccgaaagatcgagcacctcagccagcagctcgaaatccgccttgccagagccgacacgtgtaatgttgagcactatgaggccacgcagcgggatggagttgagaaaagcaacggcctttttgaaatccgcgaattttccggaagcgtcgaggaagctatcttcacggaagtcgaggcttacatagcagttccctttagtttcggagatctttgtcagatcaaaggtttctgttccaagaacgggaatgaattctagttctgccagctcttctggtcggcgaaacccgcagtctgccatcatctcctcgacccttgctgagagagatttcagaatttcggcgttatctcccctcccgaggattctgtccagatcggcaacgtaaagaaatcttggctttattgccattaaaacttccaatggatcacttttcttaacgactgcactcttctcagccaccggtctgtatttttgcctctctcctcgctctgcaagaacagcgagactcttcattacgtccattacaaatacgagcctcatagaaatgggccaagcagaggatataaaatccggagcttgagcgggatcctgcagtggaggatctcctccaagtctctggcgctcgcaaggttcatcagcctgagtagagcttcttttctcgcaaataggtcccagatcttccttatcgagtaagccttgctgagggattcgatgaggtattttttccagagtaagtcgtattcatcgaggctttttccctcaaagtatacgttaacgactctcgcggcgagatcgccagcgatcatcgatatcggaattccgccgccagtatgactgatgatcatgctcgcggagtctccagcgaagataacgttgccgtggaccgctttttcaagtggtctatcaattggaacgaccgcaccgaccttgctcacgacttccgcccttttcaggaagcgagaagaatgcggatactccctgacaaagcgatcgagaaccctgtgaacgctttcgttcgaaaactctctcctgaatccaacgccaacattcgcatatcctccacccttcggtattatccaggcgtatgcacctggagctatttcccttcctatgaacatgtaaacggtgtcctcctcacattccacgcccctcatcacatactgcttcgctgaggagagttcgtatctccagagacccaggtctttcgctatcctcgaatttgcaccatcacacgcgagcagaacttttggctcgaatgagcctttgttcgtgatcagccttccgttgcggaatccccttatcaccgttttcagctctatcctgtgcttagattcgaatgctattttctggatcatctcatcccttctaacgacgtggaactcgaagtcgaaccagaattcctttcttccagtgttgaagcaaaccatctttgtcctgttgctttcaaacttctttggaacctcaaaaagggagtaatcgtaaaggttgggaagcagggattccatctcccttctcgtcggaattatttccccgcactttacgggaaagccaagcctctctcttttgtctattccaagcacatcgagatcctttgagagaccactcagtgcaaatgctcccgcgatgccaatgcctgcaacgacaacgtccacgggaataatcttttcctcagattaaaaatcatctgctcaggcttttctcagcacaagcctttcattgtgcctgaccgagatcttcttcccgagctccccgaccagcttgcaggcctgacagacgtccttagaggtgggctctccacagatcttacagcgatttagctcgatctgcgggtttgtaacccttaaacaatttgaaagcttttcgaaacttcttaaaatcgatatcttccttcccggatatttgctttcgagctcgtatatgaaatccctcacaaatgccctgacgggaaagtagctgtaggggcactcgtcgaagatcatgggatatctcttcaggaatccgtatgttacgacttctttttcgtacaattccatgaatggctttatcctcatgataagtccttcctggtctctctgcggtatgagccgggctaatctgtctgtgtcagcatttatgaagttcatcagaatcgtttgcgactcgtcgtcgaggttgtgtgcagtcgcgagcttggttgcggaaagttcccttgctgatctgttcagcaaatatttcctgaaaaccccgcagtatgagcagggaagcttttccccgaccttgaccatctcgtcgagctttgcaccgaagttatcctcgaatttcgcaacgtagtgctctatgctcaaagacctcgtcacatctctcgcaacctcaagggtcttatccctgtatccttctatgccctcatctatggttattgccagaaattctaggtctcttcggccaccgtaaagctccagaagcagttttgacagcgtaacactgtcttttccaccgcttaaggctattgcaatcctgtcaccggattcgatcatcccgtactttttgatgactgctctgaccttcttttcgaaaaaagacgtgaagtgcctcctgcaaagatgctttccggaatacctctggtaatacacagcttctctgctgcagtaagagcacttcatggtttcagatttgccctgctgtagattattctcaagtctttaaccatttccagccctgcgatctggtgtccaaggcgttctatcgtttccctctgcgctgcatggaccatgaagtagatggggtaattccacctatcgctcggaatcctctctatgagatgcgttatctgcctgaactcgagaagccttctcgcaagtctttcgggctgttccgtgcttataacgttcattccgttaaagttaaagccaattttagactcgcttagcaccccgctgaagtccctgacaactcccttcttcttaagctcctctattaggcttacggcctcttcctctcccatccccagttcctgaaacggtctcctcacgataggcagggattcaagcctttttacttcctccccaagtccgagctcttcaattgtgtagactttctcgggctcagatccgtagctccaagaaactcccctgtaaagatcgtacttcacatccattttgtaaacctttttcgtgggcagaataacgtattccctaactccgagctcccttgcaagtttcaggacgatttcctcgatttcggaaatatctctccctttgagcgtgaaccaaacgttgaattcagcatccctgaggtagttatgcttaaccctttctccgagctcgttgatcttcttagcagatccctcttcaaccttaaagccgacaagagaggactgcctgtggtttgggaatgccctgtagttgaggtttgcaccgtatctcttcagaactccctcagcctgatactcccgaagtttcctctcaacatcctcgtatcttcttccgagcctctctgccagatcaaccaaaggagttgatgacagagggagatcgtactgaatcgccattaagagttcagaatccatcctcgacacccttgagctccttggctttctcctcagccacagtctcaacgaacttc

>Bin11_6

agtattttgaagaactttttaagactgcgagggccttcaatgagttcctgttaactatatcggatccaagaacgtatttcaaggagctaagcgatcaggaatttgaggagttttacaaaaacagcggtaagctgattttggagcttaatagagcctactggggctttttgttcgaactatcaaaggccctcgccaagggggacagtgatgaaatagcaagggcagtgggagatgctatggccagattcgaggagatttatgctgattacatggacaatccggtggtttcagcatggatcaactccattaacagcgcatacatgcgctctttgcttaacctgcagaactttacaagcgccatgcttcacgctctcggcatggtgtcgagaagggacatcatagccctttcagaggcatacgttgatctgaagggggacataaagaaggagtcgagaaaaatactcgaggaggtcagggcgttgaaggagaaaattgaaaagatggagaagccgagggagggtggttccaatgctcagtgaggttttaagaaattacagaagggctttgaagctcactgaatggttcataaagaatcccagttttttgcctgtaacagaggacaagccatacctgataccagacgtcaagataggggcgactccaaggatcgagatctcctccgacgatggcgttaagctatatcgctacgagccgatgactgaaaagcagtacgaaatccctctgctaatagtttatgcgttgataaacaagccctacattctcgacctgacaccacagaggagcgtggtgcgaaagctgctccaggcaggattcaatgtctacatgatcaagtggggggatgcaacgatagcggatcagttctccttagacagctacatcgacatattcctcgcggacttcgttgaagacgtgaagaaggactctggatccgaaaaagtttcgatcctcggctactgcatgggtggagggatgagtgcaatgtacacggccctttacccgcagaacgtgaagaacatcctcttcttagcctcaacgctctacttcgaaaagaagatcggcggtcttgtcacccttgcggacaagagattcttcaatcctgaagagatcgttgcccccttcggctacgtgccgtcttggttcctgacggagagattcaagatcctcgagccctggggaaactacttcggcaagtacataaatctgttcattaatgctgagaacgaggaattcctggacgacttcttcagaatggaacgctggatccacgacggcgtaaatgttgcccctggagcttatgtgagatacaatcaggagctctaccagaacaacgcgctcgctgaagggaagttatacataaagggaaagagggtggatccgaaaaggattaccatgcccgccgcagcaatcgttggactgagagaccatctggctccaccggagaacacattgaaattcctcgacgtgatagggagcaaggacaaggctgtgtttcaggctgatgtggggcatgttggtctcgtcgtctctcgaagggggatggccctgtgggacgatgtagctaagtggcttgcagagagaagcggaaggcttgtgaatacgaaagagatctgaatgtttgagccgaaggcaatagcgatcgatctggatggcacaataaccgatcgaagaagagcgctgaatcttagagctgtcgaagcgctgagaaagttgaagatcccagtgatacttgccactggaaacgctccatgcttcgccaaggccgttgcgaaggtaatgggggtctctgatgtcgttatctgcgaaaatggaggagtggtcagattcgcttacgacggcgaa

>Bin11_7

aagacatgcggatttcctgcacatcgacgaggacttcgttttagcgatgttaaaagccagaaaaaaagagtcagacctgggaataaacgcacatctgaacttttgcgcgcttttagaggcaaaggaaagaattgagatcgcgagaaagtgtgaagatttgatggctgaagccgtagatatggcaagaaactacggggtgcacatcatcaagccggagttttttggagagtcggataagcgagactgcccttacagagactcgatattcataaggtccgatggcttcgtctctccttgcatgccattcgcctacactcatgaggagttcgtgaacaggagatacaacagagttcgagagttcgtattgggacatttaaatgaggggatcgacgaagttgtcaagaggaaagatcaattcgaagagctgcgcaaaaatatggactttccttggtgcggagactgcggacacacggcaggatgttggtatctcgaaaacggaatggactgctatggaaacattccttcctgctcccagtgcctttacagcactggcatcgcaaaatgtatgatttagaaaaatcgaaaaagtttttaaaaactcgccagagatatttgcatgcccttaaccccgatggaagtatacaaacttctaccaaaaacaaattgcaagaaatgcggggagcaaacttgcatgagcttcgccttcaagctcataaacagggaaaagaaactcgaagactgcaaacctctatttgaggataaaaaatacgaggtccaactcaaaaagctccaagaaatggtaaagcctcttgcagaagcaactgaaacagggctaatcgtgaaaagtgagaagtgcaatggatgcggtaactgcatagtcgtctgccccgttcacgttgagaaggacccgcatggtgctggaattggaaggggaccaacgataaccgacccgatttacaggatagagaacggaaagctcgtaatactcaacatgcacgcatgcaggaggtatggaaggaacagaattctctgtgtggtttgcagggaaaattgcccaacagatgcgataagctttttggaggggtgaagatgatctgcacagcctgctcctgtctttgcgacgacgtcgaaatcgtggacggaaaggtattgaacgcatgcgagagggggtacaggcacatctccaggtatagggaaagcagagcaaaaccgatggttgaaggaaaggaagtcgacgtggacagtgcgatagaggcagcagtcgaactgctaaagtccgctaagagtccagcgatctacgggctcgacacttccacggttgaggcgcaaaaaatcgctgtggagattgcgaaaaagcttaactgctacatcgacgacaactcctccttctgtctcggagagttcgttgaggcgatattgaagaaggaaattccaagttcaactctcgatgatgtcagggacagggcttacgtcgtcatctactggggaacgaatccctatcacagccttcctaggcacatgtcacgctacgcgtattacccgaggggcgcaaagaggcaaaggggatacgatgaggatcgctaccttgtggtgatcgatgtcagaaggagcgaagcggcaaaactcgcaaaaaagaacgcgaagttcattcaggtcgatagtgacttagagcttgtggattccttcatgagggctttagagggaaaagccgggaaatacgaggttgcaagcatcctcagggagatgaaaaaggcagacttcaacgtgatctttggcggtctggggctcaaatacggattaaggggaaacttcagggccttctgtgagcttttgaggaatatcaacaagttcgcgccgctctactttattccttctggcttccatgcaaacatgaggggttttaacgagactctttttgaagcggtaaatgctgtgaacagctacagcttctcttctgcagcttcttcacctgaattcgctttcaccgagcttttgaaaagggaaagaatcgacaccgctctgatcgtcggaacggatccgatctcctctttgcccctcgaagcctcaggcaagcttccgaagttgaagaagatcttagtcgatcccagaaatacgctaacctcgaaaattgccgaagtcgtcatcccctcagccttttcaggaatcgaaagcgggggagagatggttagaagtgatggcgtaagggttaagctaaagcccattgccaaagcggaggtcgatgatgtttatgtgctgaaaagaattctggagggattgtgatgatgcgtttaacgaggctaatgaggttcgaagcaaagattgtggttgcaagggtttcggaggagaccagaaaggccacaatttacctgaatcctgaatttgcaaagaggtgcggcataaaggaaggagacgttgtaacggtttcaagagccggtagggagctcaagttcagggtaaaacttctcgaaacggctccagaaaacggcggaattattccgaattcgatattctcgaacttcttggccgacttcgatggctttaagagcttcagggcagatatagagctgagcgagggtgatgagagcacagaagaagatgtgattagcattatcatgcaaaagaagtgatccccagccttaaaataaaaaatgggcaatgataaacgcttcagcgtgcaaaaagatttatttattcatgctaaattgcagatatggacgctactcttgacttatttttccctttgttcgataaagagcaagataacgatcgctgggccctcgatggcgttagggagtatcacaaaatgctaatagaaaagcttgaagaagcctacaggattgcagagactgccagggctaaaggtcttgatcccgagccaagggttgagattctactcgcaaaagacatggctgaaagggttgagaagctgataggccttaacggggttgccaagaggataagggagcttgaggaggagggtcttgaaagggacaagatatgcttcaggatcgccgacgagataatagagggaaagttcggtaaaatggatgttctgcaagcaatagacaaatctgtccgaactgcagtcgcaataatgaccgaaggtgttgttgcagctccaatagagggcattgcaaaggtttcaactgacaagaatgccgatggaagcacttttttgaaagtatactacgctggcccgataagaagtgctggcgggactgcgcaggttatttcagttctgattgcggattacgtgagaagaaaactcggattggggaaatatatcccgacggaggaggagattctcaggtactgcgaggagatacagctatacaaaagggttgcaaacctgcagtatttgccaacagacgaagaaataaggctgatcgtttcaaactgtccagtttgcatcgacggagaaccgactgaagaggcagaggtatcgggatacagaaatctgcctagggttgagacaaacagagtaaggggaggaatggcacttataattgccgaagggatagccctgaaagctccaaagctgaagaagatggtggattcgctgaaaatagatggctgggaatggcttgagaggctgataaaaaaggactccgaggaagaggtagacgtaaagcccaagagcaagtacctatcagatcttgttgcgggaaggcctgtactttgtcatccatccaaaaaaggtggctttagactgaggtatggaagggctagaaattctggactcgcgactgttgggataaacccggcaacgatggccctgattgatttcatcgccgtagggacccagctgaaaattgaaaggcctggaaaggctggaagtgtagttcctgtaacaacaatcgaggggccgactgtgagactgaagaacggagatgttgtgagaattaacagcgttaaggaggctttcgagctgaaaaacgacgttgagaagatcctcgatctgggggagattctgataaactacggggactttctggagaacaaccacccgttgatgccagccccctacacaagagaatggtgggctctcgaaaccggaataaaggatacgcatatcgacgaggagaccgctctcaagctctcggacgagcttggagtaccattgcatcccgacttcacgtatttatggcacgacataagcatcgaggacttctgctaccttcgcgactatatcagtgccaagggaaagatagagggtcagaaaaagagctgtctacttctacccttcgatggaagggtaaaggaaatcctcgagaaccttctcgtggagcacaaggtcagggaaggtatcgtgatcgagaaatggaaggcactcgtcagatgcctcggactcgacttcaaactcagaaaaatcggagaagttaagggagaaagcattttggaagtaataaagaacgtttcaggtatcactgtaaggccaaaggccctcacgagaatcggggcaaggatgggcaggcctgaaaagtccaaggaaagaaaaatgagccctccaccgcatttgctatttcctgtgagctttgcgggaggaaaaaggagggatgtgaagagtgcgcttgagcacagaaatggctataattctgcaaagggacagatagaggtcgaaattgcagtgagaaggtgcaagctctgcggcaaggagacattctggcttaaatgcgagtgcagcggagaaacggagcagatttactactgcccgagatgcaggatcaaggacacttcggagctctgcaagagatgcggaggggaagcaagaggttacatgaagcgtgccgttgatataagggctctatatgaaatggctatccagaatattggagaaactgatgacattgaggccgtaaagggcgtaatcgggcttacttcgaagaacaaggttcctgaaaggatcgagaaggggattctcagggccaaacacggtgtctacgtctttaaagacgggacgatcagatacgacatgacagatctcccactcactcacttcaagccgaaggaaatcggagtgagcgtggaaaagctcagagtgcttggatacacgagggactacatgggcagggagcttaaggctgaagaccagatcttggagctgaagccccaggacatcgtaatatcaaaggactgcgccgagtatttggttaaagtggcaaggttcatcgatgatcttctcgtgaaattctacaaaatggagcccttctacaaggctgaaaaacctgaggatttaataggacagctcgtgatcgccttagctccgcatacctctgctggggtccttggaagaatcgtgggattcgcagacattctcggatgttatgcgcatccctacttccacgctgcgaagcgcaggaactgcgatggcgatgaagactgcgttatgctccttctcgacggcctcctgaacttttcaaagcactttttgccggcgaagcgtgggggacagatggatgctccgctggtgctgacggtgatagtggacccgagagaggtggataaggaagtccacaacatggacatagttgcaaattatcccttggagttttacagggctaccctcgaatttgcgaatcccaaggtgtgcgaggatttaatagaaaaggtgaaggatcgcctaagcctcgaaacaaggttttgcatgctgaatttcacccacgataccgatataactctcggtgtaaaggaaagcgcttacaaaaggctaaagacgatggaggaaaaggttgagaagcagatggaggttgcaaggaagatctctgcagttgacgaacacgatgtggcggagaggataataacgatccacttcctgccagacatcattggaaatctgagggcattctcgagacaggaattccgatgcgttgactgcaatgagaagtatcgaagactgccgctgacaggaaaatgcagaaagtgtggcggaaatctgacgcttacagttcacagcagctccatagtcaaatatctcgagctatcgaagaggctttgtgagcagtacaacgtcaccgaatacacgaggcaaaggctgaagcttatagagtatgagatacggtcgctgattcaggaaaacaagcaggtaactctcgttgacgttttttaagcctttaagtaccaaaatcagccttaagagtatccgattaaccaaaaaaaccatgcgccgaccgggatttgaacccggggcaggggctcggcaagcccctgtgttagaccaggctacaccatcggcgcttgcaacctttagtatgtggcagtttaataagattttcgcttacatctgccctccctcggttgatcgcagctccatttgaatgttaccagacttttgccaagaacagtgctgcgattttaagacgcctgaaaaattcctcagactttaaatacttcccctgagtcaaaagcctcatgatttcgggtaaaggtgcaaaacttcaaaaataaaaaatttaaaaatattttcagccccagacgacctttccttcgctgtccgtgtaagtgtattcctgtccccagaacttcggcgttatacccaagatcacgatcaattcgccattgtcaaagactatatccgaggagggcatgtcgaggacaccgatggttccgtatggtgcaactcgtagaagtgccccatggaggtacttcggtactcccctatatccatcagccgggaacacctttttctccatcgcttccattattttctccggatctgtggaaccagccttctcaatggcctttgctatcagcatagtcgtttcgtagtttagcacgtgctcccaatttaagggaatatttccgagtctttgtggcagtctttcacacatgtatttgtagaacggggtgtatggatcaaccgaagcttcgggaagcaccgccaagatgcatggagaagtcgttccaatgctcttgtccactaagcttatgtctcctttcaaaacttttttgttggcgaaatcgtactctatgccgagatagtacatgatctcgtccaacttagcctgatctgcaactataaagcctcctgtgtagcctagggctcttgcagtcttaatgactccagccagaggcaatgaagggcctgcgatcattatgacttcaggctttgcagccaacactttcgatacattcgctgtgtaatttgttgctgtgtaatagttaatgggctcttctgcaactatttctccgcccttacgagtccagtattccttaaaggcttttgcccattcctttgcatactcatgcgttcctggcaacaaggcagctttcttcatccccttcagaaggaagtagtcggccattgtaggtatgtggagaatttcgaagtttggtggaatgagaagagtcatcttgtttccagtttttaaaactatgggactggatgtgtaagctgcgacgatgaacttattctctcccttttcatttatcttctgcagctccaatattccaccggagtgcgggcagaagatcacgggagttccatttttggatatgttcacagcatttgccgcggaaagcggaggatagtacttgtcatccattgcgataacgtccaactcatacctcttgtctccgatcacaattcctcccgccttgtttatgtcttcgatcgcaaattttatcccattgagagcgctctcgccgtatggagctgcaagtccgctcaggggcccagtgtatccgagatagatcttaccaccgtactcgagtggcttgggcgtggttggagcttctggaggtgctgaaagattcacagcccagtagactgccaaaatcgcaactattgcaaataaagccagtacaatccagttcctggcctgcattctatcaccttttaacgctatcactatcgaatatataaattttgtgtttttgatataaaggctttttgtgagatgctgctgtgacaaagacgcgcggccaaacgggctcatccctttaccttttcaacaactgatataccctctattcttgtctcgggatagtttcccgtgctgtccttttcgagtgattttaccatgtcccaaattgtgagcagggctacgcttgctccagtgagggcctccatctcaactccggtttttcctattgacttgactgtgcatcgaactctgattccactttcctcggtttcgaagtcaaacgaaatcgatgttatcggtattggatggcacattggtatgagctcggaggttttcttaacagccaaaactcctgcaatgtttgccgtcgcgatcacgtttcccttagccactttgtttttaactatcgcatcaacagtttcccttttcagcctgataaagccttcagctgtggccattctcgggacatcctccttttcggagacatcgaccattctgactctctcgccctcgatgtgagagaactccatgcaatttgttggcagttaaaaatatatcccttatgccagacgatagcccatgagggcccttgtagttgtggacatgcagaaggatttctgctacaaaaatggcgccttgtacattcagggagctgagaaaatcttcgaggcgacaagaagggttgtggtgaatgcaaggggaaaaatacctgtgatcttcacgcaggactggcacagggaggacgacgaggaattcaagatctggcccaggcactgcatcgcgaacacggaaggcgcggagatcatcgaagagcttgaggccagcccgaaggattacttcgttaaaaagagaaggtattctgcatttttcggcactgaccttgatttgctgctcagagagttgaaagttgatgaactcgtaatctgcggcgttgctgcgaatatctgcgtgcttcacaccgcaggagatgcggctattaggggatatagggtctctgttatcggagactgcacaaaagcattgagcgactacgattatgaatacgccatgaagcacatgaaaagtgtcttcaacgcgaggattatgagctcagagcaattcgtcaaagaactctgagccgtaaatatttaaatcataagatggtgactttgtttatgaagagcgtaataatcgttggatgtggtagctacatggactctacctacggatgcccgggagagtggagatgtttaaaggccgcaacctttggagagggtgaattctcggaaccttcaagggttatagccttcgtaagatgtgaatgcccgggaagggctacgcttgcaacgataaaaatggcgatcaagctttcaggctcaaaggtagacgagatatatttgagcaactgtttctacaagcccaagccagagtgcccctactttaaagccgaagagtttgcgaaaaccctcgaggaaaaccttggaattaaggtgcacttcgggacgcattcctacgcttagcctacccgcaaaaatttttatcaagtggagaataactttctgcgatgagcttaatcgatgaggctaagaggggaataataaacgatttgattgaaagggtctcggaatgcgaaggagtagaggctgaaaagcttgttaaattggtggctaagggttacgtcgtaattcccaagaacgttgttcgagaagttgaaccaagagccatagggatgctcgtttcaacgaaggttaacgccaacgttggcacttctcaggaatacgtgaacttgaaggaggaaattgaaaaggcaattgttgcgcagaagtatggtgccgacgccgtcatggatttatccacgggaggcga

>Bin11_8

caaatgtattcgagtattccgtattttgaagccgtgggcaaaaacttgtccgcctcgctcgtcacgattgccgtaactcctgcgcttttcaatatcctcctgagagaggaaagtatctgatagcgctcggcatcattgaaaagcgtctccaaaacgcttatcgagtctatgagcattctgctcgcattcagatttcttattagctccggaagctcgctttcgattctttcgaagctctttttcacgcttagtgcctctaagcggatcacttgaactttttcgaacttttcgagatccattccgacgcttttcgcattcctcactatactctcctcatcctcgtcaaagcttagaattatgcagctttcccctcttttcagaccctcgtaaacgaagtgaagtccaagtgtggtctttccagtgccgtagcttccgatcaccgccactacatagccttttggaattcctccgcccaaaattgcgtccaagccatctatgccagtggaaagcctcataaaaccctcatgattcgcgagatagtaaagccttgcgccgggtctattttcgtgctgtacttcgaaaccctctccttttcgaggatcggcattattccgagcatctttctgaagtacatccatctcgtgatcgcatctctctcggcaatccactcgaaaacgatgaccccgtcagcggtttcaaaaagctcttcttcgagccccttttcgagaacgttggaggtgagcagcgccagaaggagtatgttcctttttatgcaaacagccttgaagcccttcagtaggtctatgagcgactcccagccgagccttttcgagacccttgcgagatccgtgatggagtccagaactatgattcccttcaccccgtcaaaaacctcaacaagttttgagaggacgttcttctcgtctttaaggacacccaaaacgctcttctctgaaatccacttcatcggaattattgagtccctgaaatagtactctgcaaggcttatgatatttatgtttgcctccctcctcatctcgggaaaggaaagcctgatctccctctggacttcttcagccgtctttgcaatcgctaagtagttcagctcgcatctgtttgcattgttgaagagcattgtaagggcgaattcccttgcaccagctccgctgttctcatagagtattaccagaccatcgtgaaatccgccgatctgcttgtccaagaactccaggccagtgggaatcatgcctcgatcacttcgtcgcacatgagggagatctgcgagtagtaagtctcatccatttccttttcaagcgtgaagatcgtgcccac

>Bin11_9

taacagaatgggggccatgaatgtggcgatcctgaacatagatggaagggattttccaaaactgggaataaaggcagacaaagttctcctcgatgctccctgcagcggggagggaataatttttaaagatgaatcgaggaaaagggataggggcatggaagatatagccttctgttcctcgctgcagagagagctaataatcagcgcattcgactccttgaagcctggcgggattctggtttactccacatgctcactggctccggaggaaaacgagttagttatcgaatacctcttagataagagagaggccgagcttcttgaagtcgaatacggtgaaaaggccctcaatttgactaagagggatttgagcaaagcaaaaagattttatccacacatacacaggtgtgcgggcttttttgtcgccaagatcatcaagctttcctgaccttttccctaagttctctgagtaaaggatcgatcatcagcttagagaactccaccatccccttgctttctccaacgacgcctatggaaacgtggtcgataaagatcgcaaagaacttctcgccgtcgaagatgagaaggccgtgagaaacatctcttaccgccttttcacccttgtattcaagcttgtatgcttctgcctttagatcatcgggattttccgagatcacgaccagtttgcagttggctttggacaggatctcctttatttctggctttatgtgggatatcagtgcaattatttcctttttggaggataaaacaagttcttcgagcttttcgagcaccgattttccacggtaaagctttacgatctctatatcctctgttctaagcttcgagatttcctcgcgcaatacttcaaggttttctgcagtttttttcgagatcatcgagatcatctcctcattgcttatagccctgaacctcacgggctttccaaactcttccacgaaaccctttgctttaaggctgttcataacatcgtaaacggaagtcctcggaattccgctgagctctgccagctctcttgcgctcagcacaccctttgaaatcagcgttaccaaagcccttgcctcgtaatcgctaagcccgaaggacttcagcacttccacgatccgcatctcgaaatgctccatacacaaaacttaaatatctttcgttcctaaagctacgacaggtgaaaggatgagaaccgccgtgatccttctattgctattgctggtatcgattccagcgtgcggcctcaaatacgagagggagcctaccgttgaggtcttaagcgtgagctacgtggacatccagaagggaataacgagtccaataaattcgaattatcttgggaagggagaaaaattgcttgtcgttacgatctacaatccagccgcagtggaagaggtcagatacgacagcgttctggaggcaagctttttccactcccggcaggacctgcttttcaccgcctacaacatcgaggctgagctgatagggaatgatgatattaaagtgaagaccggaaaaatctcgattcctgctcttccagcgttaacccagcaaattcctttatactttctcattgaagttctggggaacaacgatacggagctgaagctcaaggtaaagtacgagaagatcgacggactcaggagccttataccttacgccacggttccaagcggagagtggacacagacggtcacacttgtaggaacaccaagcccaccacccgatacacctacttgggtacaaattcaaaacacgaccacgattaaatactactccacaagcaattacgagctcgattatcgcaccgagacaaaggagatcccgataaagcttttcatcgaagcgaaggaggttctgctcgaagttaaagatgtgaaggccgatccactgatcgcaggtggaaagggaagcattgagctcacgataaagaacgttggtaagaagaccgcaaggaatgcatatgcaacgctcgagctgccgaaatcacagcagagttcaccgcaaagcgctacgcttccaacatcaatgcttccacttatgatgccttctggcatttccactcccgccgcaacctcctcatcatcccagccagcttatttcatcggagatctcaagcccggagaaactgcgaaagtgagcttctacatcccagttgaggtctcaaagggaggagtatatcccgcaaagctcaagcttgtttatttagatgattacgggaatctgaaggagagcgatcccgtgtcctttggcatttcagtgctctcgaaaccagagatcagcgttaagactgtggagagcaggatgtacgtaaattccaagggggatctgatcgtcaaaatgacttcgaatctcgacttaagcggagcttctgcgaggatctccgtaagttctccgctttcggctttgagcagtgagtgctacattggagacattaaagcgggagaggaattctcagctttcttcaagctccgtgcatcgagcgaggctaaagcagggaagtatccagctgatctttacataaaattcaaggtgggggatgattttgttgaaaccgatgctgtaagaataggggtcgaagttctgcctgagatagagttcgaagtgatgggaactccagagataagagcaggagaggagaaggttgtaacctttgcagttcgcaacacggggagcgctgaagtgaaagatgcaactgcgaggcttgtgatagtctccccattcacctcttcggataatacagcttacataggtaatctgaatccaggggagatcgcgaatgcgagcttcaagctctcagtcgaccgagatgcaacgccaaagctttatgcccttaatctcgaggtaaagtacagggccgaaaatggagactgggttataagcgctccagcgaaggcagtgataagcgtgaagccatacgaggttagctacacgctttacctcgtgatagcaattctgatcattgcaggagccgtttactacctgagaaggagaggatgaggcttctccgaatagtttcagagaaacccggactcgtatttatttttgtaactgtaatcatacttctctctgcctactccgcgatgaacgttgagatgtcttctggcaccgagaacttcttcagcaaggacaacagggtctaccagcagtacaagctttacgagaaggacttcgtgcgcacaaccggggcagttttcattctcataaagggtgatgaggtcgtaagctatgaggcctacgatttcatgctcgccctcggtgaagagctgaagaagatcgagggagttgaaagcgtaacttcgccagcgtcgataatcagggagcaattcggattccttcccgctgacgagaaacttttgaaggacataaccgagtcctaccttttcgaccttgtgccaaagcgaacgctcgcaatgatgatgattcagctcgaacccatggattcaaaaaaagaggaggaggttgccaggaacatagaggagagcatggagagcataaaaatccctgcgggttacagggttgagataacgggcactcccgtgctcgggtatcagataaagggagagatcctgaagagtcttggcgttacaatgatggcttcggtcgttttgatgattctcttccttttcttaaccttcagtggcgtaacaagaagaaaatggacagccttccttcctctgctcatctcggtttttagtgtcaccatcatctacggaatgatgccacttctcggcataccgctctctgaacacacaaacggagctttgccgatgctcgtgggcttggcaattgaatatggcgttcagattcagaacaggtttgaggaagagatcaggaaacacggtctggacactgccttgaagtttgccgttgaaagaacaggaagagctctgctgctttctctgctcacaactgtcgttggtttcatgtcaatgctttcggtgggcattcccgcgatgacctggttcggagtcatcgcatctctcgggctcataatagcgtacaccctgagcataaccttcctgccagcccttttgaagataattgagaagcgcggaaaaggcaaaaaagaggagaaggctgagaaaaaagctaggctcgaacaggctctcgaaatggtatccgggcttacagcgagcagaccttttggtatactcgctgtagccctcgttatctctgcagtcggcctatacgtctcgccgatgataaagcttgaaacgaactacaacaagtatgtaccccagaatttgccctcgattcagaaattcagagagctcgaagaagttgcgggtggacaaacgatctacaccctcgttcttgaaaccgatggtgtggattcgaggactctggagaaatccaaggatttggcatactacattttgagcaaggaggaactcatttacagatacgattcggttaactcgctcttagaggcttacaatggtctcgagaatgtgccggaagagcagctcaaccgctatgtttcgggtagttcgcttgcgattcacttctactccaccgcagacagttacgaggagtacaaaagcactttgaattcgctttacgaggatcttgaattctacggctgggacgggaattactacgtaactggccaggcagttgtttccaccgaactcgggagcatcatgatcaacagccagacgaagatgaccttggtcgcctatgctggcgtccttgcgattttgctagtgatttacaggtctttgagaaaggccatagtgccgctgcttgcgataacgtcggttataggcgtaatgaaccttgtaatgttcatttcaggagtaaagcagacaatggtttcgatagcgctgaactcaatagtgctcgggttgggaatagacttctcgatcatgatcactgagaggtatctggaggaaaggcagagagtttccgctgctcaggcagtaaggaaagcaatcgaatacactggaaaggcgacaaccacttcagccttggccatgataggaggttttggagccttgatgctttccacgtttcccgttatgagggattttggatttttggctctggtttcaattgctctgagccttatagctgcatttacagtagttccagctttcctcatggtcaccgaaaaactcggagaaaggctaaagacaagtggactacctggaaaatccctgagaaataggttctaaaaagcttttatcttttcagaacaacatgttatatgaacgccgaagagtacgtgaagatctttgaaatcgtcgcgaaaagaaaaaa

>Bin11_10

acagaaataaccctgtagccacgtctcttcaactcttgtgcaacgctgctcttaccgcaaccaggtgtgccggttagtgctatcatagcaattccttcagcttctgaaggaagaaatcgttttcttccggttttccaacggtaacacggtatccggaaattccaaagcttctgcaatcccttattataactcccttctttagcagttcctctgcaagaccctcttttgacctgaagaatatgaagttcgcctgtgaaggataagcctccacgagcttttccagctcaccgaatagcctgcccctttctgacttaatcttatcaacacagcttttcatccattctaagtcctcaagagatgctattgcaaccctttctgccaatgtagagaccggaaaaggagtagaagctgagcagtagtatttaaccaaccaatccggaagcctggcatagcctattcgcagatttgcaagcccaaaggctttcgacatagttctggctatgattacattttcgagatccaaatcgagatttatgtctgcaaactctatgtaagcctcgtccaaaaatactatggccttcgtcgattctgcaatttccttcaccaatctgggatcctctatgtttcccgttgggttgtttggaaagggtattatgacgagcttggttttttcactcagaaggtcgattatctgctctggaatatggaagtctttccctctttgcactctgatctccttagcgcaatgtaagcgggcaagtatgctgtagtactgaaacgtggggacgggaattacaacttcgtcccccttttcaatgagcatcgtgaaaagcgtttccaatattccgtcgatacccgctccgatgactattctctccgcttcccagccagtatattcctcaagcctctctcttaattccctgtattctggattggggtacctgtgcagatccgtaaattttctgagctcttctatggcccttggacttgctccatagggattctcgttcgaagcgagtttcacaactttttcgaggccgtatagctcctttacctcatcgatactccttcccggaatgtagctctcgaattctatgcattctctcatctaaccacttctacttctatcctcttccactcctccctcacacccttgagcgatgtcgcgagagcttcgacaaccttcccgatgatcttcattacgaatggattgagctcgatttcctcaccatcaacaaccagcctgacctccattcataccaccttcctgaactcgccaatgaagttctttataacttcttccgttacatgaggcatgacaacgaatcttatagccctcggctcccttatcgtcgaaatcacccatctacgcctgtaaagctcggccttgatttcctcggctctctccgttttaaaggccacgacgttcatcacgggctctatgactggctcgaagcccaaggcggaaatttcctccacgaggattctcgtgtttttcagacacctcttcacgatttccctcattccgctgaagcccaagcctttgagaactgcgtatgccgaagcgactccagtgccaggccttgtccctgtgagtgtaaactgcatctttgaggttagatagggcgtctcaacctctaaggccctcagaaagctctcatccctgaatagtatgcctcccgctggaatcgtggccatgcccattttgtgcggatcgatggtgatggatgtgacgcctttgttggcgaagtcgaagggataaggcttatccatgaatggaatcacgagcccgccgaaggctgcgtcgacgtgcaacggtatatcgttctcttccgcaagctttgaaagttccacaatcggatctatctggcccagctccgtggtgcctgcgattccaacgatcgcgacggttctttcgtcaacaagtctctctacctcggcaagatcaaccctgtaatcttcgtcaacgttggccctcctcacctcaacgcaaagaatgtcgcatattttctcgaatgaaaaatgggcggtttttgggatcactatgttcgggtctttccttctcttcacgtttcttgcagctcttatcccctgaatgttcgcctccgtccccccggagcagatgtagccgacgcaatctttgcggtgcagaagctcgccaagcattgagattagccttctttcaagctccacagttccgggaaagatcccgggatcgccgaggtttgttccgatgaacagttgatgagctttaactgctaattcatgtggaatcgtgcacatggagctcaaaacccttgaatagggaatatccctctccctgaagctcttgagttcctctaacacgtccatggccatcgtcggataaaggtaataaatagataactaaaggagccggaagtgtgaagctcgcagttctaatgggtggcagggggaggagaataggagttgagaagcctgagcttacgatttgcggaaaaaagctgatagagattgcaattgaaaagtattctgattacgaccccgttttcgtatgccgggatcattctcaggctaaaaaatatgccgagaacttcggaatcaactgcatttgcgacttttacagggactctggaccgctcgcagggattcacgcagcacttaaacataacggaaacactgcagttgttgccatagatatgccattcgtaaagatagagcttctcgagttcatattcgaaaagggggttgagcttgactgcgacgcgctgattccgaagcacaggtttgccgagcctttgctcgcttactactctgaaagatcggtttctgaaatcgagagggcgatcgaaatgggtgagaagaggatcattgtcccgctcaccagactttgcgcaattttttatccggcggatgagttaaggaagtttgataaaaccttggtatcctttttcaacataaacacacctgaagacttggcaaaggccgaggaaatatgctcacggatccatttgggagaactataaaaaatctgaggatagctgttacgaacagatgcaatctgaactgcatttactgtcacagagagggtgagagcgacccgggaactgaaatgagtgccgaaagaattgcggaaattgcaaaagccttttacgagcttggtattaaaaagctgaaactcactggcggagagccattgctcaggaaggacatatgtgagatcatttccatgatgcccgattttgaggagatatctctgacgacgaacggaatccttctgagcgatctggcattcgatttgaaggaaagcgggctcgacagagttaacatcagcttagacacgctcgatgctgagaccttcaggttcatcacgggagggggagagttgagcagggttctagaaggcttaagaatggctgttgaggccaaactgacgcctatcaagctaaacatggttttaatgagtggtctgaacgaatctgaggtcagaaaaatgcttgaatttgcgaattcgttcaatgagaatgaaacaaacgttattctgcagctgatagaactcattccaaattcaagaacggggaaattctaccttgatccaacaattttcgagaaggattttgagagagttgccaaagcggtgaagatccgggacatgcacaggagaaagcaatttataactccttttggggttgtggaaatcgtaaagcctctggacaacaccgaattttgcatgcactgcaacaggatccgcataacctcggatggaaggataaagctttgcctaatgagcgacgagacggtggatatatccgaactgagcggggatgagctgaaaaaggcaatttttgaggctgttaagagaagaaagccctttttcatttagactttacggctctcactattttcctgactctttcagcctcaatcctgcccctattctttatataactccccacgatcactccgtcgcagtgttctgaaatcctcctagcattctcggcattcacaccgcttcctgcaagtattggagccatcaggcaatctcttgcaagcttcagtttttccaactccacttcctcccctgtcgaccttcctgtgattataaaagcgtctgctaagctcctttcagcatttaaaaggtattcctcaaagctggcaaaatgggctgcatgcttcacattcaggtctgcgaaaatcatcgctttgcatcctattgcccttctgtatctcatgatctctccagcttttgggtggagaaatccctccggtgcggctgaggggaacaggagctgattcactctgatgaagtccgctttgactgcctttgcaattgctaatgctgctatggcatcgttcctgagcacgtttatccccagccctaagctcgttgccttttttatctcccacgcaacgatggccatacttgcaactgtctcctttccaacttcaattagaaatggcatgtcgccgtaattttcaatgattagagcatccgctcctccatcttcaattgcttttgcatccctcaaagctctttcgagcacgtcatcgaattcctcgtgcagcggtgcgcctaaaagcggtttgagatgaatcacgcatatcactgtcttctccatatgctctctcctccgtattttatttcaacaattctgtgctgcggaatggctgctccgcttttaagataaacgaatttgtgcccgatctcctcgatatcctctcctttgaattccagaaatccctggaagcggtccagataaaccacaataacgcgggaaaaatcgtattcgggatgccatctcagcttgttcaggatctctcttgcgctatatctcctcatccctggcaagctcgattatctgtcccttaaggttgaacttgagagacctcttcacgatcgccctcacaaaaaggggatcgtagttcagagtcttcgttatgtgcccgacagaattccttccgagctcaacttctctccttatcttctccaagtgctcccgcagctcgtcttccgacatgaaaacgatctcaagaattctgttgaattccttcagagagacgtagaagtttgccgagatgtgcgtgtagctgacgtggtactccttttcgggcctccctccgggatctgaaggcattctccatctcacttcaagcattccggcatgcttcaaaatcctcagagtttcctcaaaaccctctccgaagtttttccttagctcctcgagcgtcagccagccgtcaaggagggccgagtataccttcctgtggtattccgacgaaagcagctggaatatcggtattagctcaactatgtcgttgacgagcttcgctcttttcaccatctataccattttaaatctcaagacaattatacttttccacgaagcgcacaacttccccgaccaccatgagggctggactcgagaagcttctctcggatctggaaagtttccctaaggtggaaacgaagactctttggtctttcagagaacccttctggatgatcgcaaccttcgtctcatctttaaaaccgtttcttattagatctctcgagatctcggaagctgagattccgctcatcagcacaacaagggttccgttcttcagggcttctgagtaatccttgacctctctccccccgatcacgatgatcgcttttccgaattcaggatgcgtcagcggcaggtttgcacaggctggcactccattcacggagctcactcccggaacgatctcaaaaggaattccattttccctgagaaaaaccacctcctcccagagtcttccaaagacagtcggatcaccgccttttagccttagaaccttttttccctcttccgcaaacttcttcataagagcatttatctcctcctgcctctcctttccttttccatcctttccgacataaatagccttctttccaaggttttcgatcaatcttgcaatctcctctccgaccagtctgtcgtaaagtatcacgtcgacctctttgataagtctaagggccttcagggtcaatagctccggatcccccggcccagcgccaacgatgtataccttgccgttcacaattcggatttttttctcgtgcttaaagctttttttaaaatcaatcagaggacaatgttctgcctgtacccgcagctcggacacctgctatcgctcagattcaccctttcaatgtaaaaacccctcctttcaattagaatctccccgcaattgggacagaaggtgttctcaaacctgtgcccccagacgtttcctatgtaaacgtactccaaaccctcgcctctcgcaatctccactgcctgctccagtgttgacacaggggtctcgattttgtccgtcatcttgaaatcggggtgaaacctcgaaaaatgaacgggaattctcttgtcaagcgagacaagccaggaggcgaaatcctttatctcttccctgctgtcgttctcccctgggatcacaaggtatgtgatctcaacgaaaattccccttctgtgcatgaattcaacggtttttagcacattttcaagcctcgccttgcagatcttgctgtaaaatcgctggctgaatgccttaacgtccacgtttgccgcatcaagccatttgaactgctcgattgcctctaaagtcatgtagccgttcgtaacgtaaacgacaaagtatccttcctcctttgcaagctttgaggagtcaagagcaaactcatgccaaatagccggctcgttgtaagtccaggcaacgccatctgcccttctatctctgcacaatcttacgacgtcctctggcgtgagctcccgaagatacgggtagctgaggtcggcaaatgagatctcgaaattttggcagtgatcgcacctgaaattacagctaacgctgccgaaggacagaactttcgatccgggcttgtagttgtgcatgggcttcttttcgatgggatcgagggcaattgaggatgccagtccgtagttgtagaccatgagttttccgttctcattctttctgaccctgcatatcccccattgctggtctttgagcttgcagcgatgccagcacgtcttgcagaccacaaatccctcgtctctatcgtaaaggtaggattctatcaccataaactcttcagctccttggcgtagtcttcgggggagaagaacgtgacgctctcatcgtgaaagagcttcgagaaaaagacgtcgcaccagtagtattttgaaaacggtgttctcgctacaatgtgaaaattcgtcctgaagtgttttccgattctgtccccgcagaagaatgtgaagttaaaggcgttgaacctctttgtttcgtagaacctcaggatctttacaataccttccgagaaaccttcgagctgttcgtcgctcatagaagcgaactcagtttcttctggaacgccaatgaagtggtagaatcccttgggagcaaatgctgcaagccagtctgttttttgcgttctgccaacgtatctctctccccctttttctttctcgaccagtgaagaccagtagtcccttccgttcacttcgaagaactcgaaggcactccactcgaggcgtgcgaagaagtctgttgcgttttcagaaaccagaagctgaacatggggatgcatgacgctgctgccagctgatttcaggtagttcatgcccacagatatgtatattttcccttcgggcagcttggtgatataatcttggatcagcttgaatgcatcaaagaagtgagattttttaaattcggagagggagaggtaatggctttcggcaatacgcaggattaacgaatacttcgagtaaggcatgatgttcgaaaaagcgaggcattctccgcgcctccatatttctcctttcatgatttccggatcccttgcagcgagatcctccaccctttcgttgcagaagggacagaattgtctcgaaagctcgatttcctcttcaaagccaccagaaaccggtatcggaatgcttttcttaactatcctgctcgtctgaagggtcaggggatcgtatctgatttctacagcactttcctcaaccttgtcgccaattaaaattcgggaggtctccaactctctccgaaactccataaatccagtggttccagacagatttaagttttgcgagaacctttttaatccacttcttacttcatgtgtgaagatcgaaaggccaaggggcacgagggactttctcccagatgagatggaaaaaagaagggctatcgagatgaaactcagagagatcgtggagtccttcggctacagagaagttttaactccaacattcgagcactcagaactcttcaagataaaatctggggagggaatagtagaagaaatgtatgtcttcaaagacaaatccgaaagagagctcgccctgcgtccagagctgactgccccgatcatgaggctttacgtgaacgaatgcagcaacattccaaagcccatacgcttctactacttcgaaaactgcttcaggtatgaacgcccgcagaaagggcgctacagagagttctggcagtttggggttgagctgataggatctgattcctatcttgccgatgc

>Bin11_11

gcctgaagaagagcgcttggctcacatgaagaagcaggggttgtttgtctttggagttgaagcaaagctcgtggatccagccactggcaatgaactcccctgggatggaaaaaatgttggagaactttggctgagaggtccttggatcataaaagaatactataacgatccaagaacaaaggaaagtgtaacagaagacaaatggtggaagagtggagacgcgggaacaatagacgaattaggatacttccacatagttgacagattgaaagacattataaaaagtggtggagaatggataccgagcgtggaccttgaaaaaacgctcatgagccatccatacgtttatgaagctgttgtcgttggggtgccccatccgagatggggagaaagacctctggcccttgttgttctgaagaacccatacagaaccaagccaaaggaacagattgagactgaactcagggagcacatgctgaaaaggttcgcaaggtggcagctgccagatcggatccttttcgtcgatgagattccgaagacaagcgttggaaagatcaacaagagaatcctgagggaaaagtacagggacatcttcctcaagtagcaaattttaatcatttattttttatttttgctaaaggattatgcaattgtcctccatggcctctttgcagctcaaatggaatggatatttagctctctcttgaacacaggcaggcataccaaaaaaggaaaccaaaaatcttaaatacaactcaaaagccaagaaaatcatgaaattgaaaaaagatgtcaaaaaatggtgggggataaaagagaatcggtatatcgcacctttgacaactctcgggcacagcgattaccttctggggtcagaggaaaggggaggttttgaacaaaaaatgatgagtggtggttgacgtgctcgcggagctatctttgatagttgcctacacctttatcttcggttcgatgtatctcggaatggccctcggatttacaataaccacaggagtgctcagagtcttcaacctcgcgtatggagctatcttcttggtagcggtatacggaacatggatgttctggaacgatttcggtctaggacttccagaatcgatcgttttagcaatcgccctcgtggcgctcttcacctacgccgtttaccagctggtcattgtgagatttgccgagctcgaagactacatgcttgcagctctcgtagcagtgttcgtcatagttgaggagctcatagggtgggcatatccggaaattgtcggcgtatacatacccacaacagtatacgacgagctcgttcccttcggtgaggcaaatgttccgggacagtacgtggttactgctttcgtctccttggctcttttagcgatctatttgctcttctttaccaagacgaagaacggattcataatgagggcgataagccaggacatgttcgcttcgaagctcgttggtgtgaacttcaatagaacctttgcccttgcaatggtcatagcatcgattccaccggcgatcgtaatgctcctcatctccccgatatgggcgctaaatccatacatcggatggagtgtgttcacgtatgcgattctggtggcagttctcggtggcttgggcaacctaaagggcagcataatggccgcgtacatcattggcttcatacaggcctcagttggcttcctgctcgacccgaggctgatgcttctcgttgcactggtcgtggtcatgttcgtgctgatcgtgcgtcctaaagggttggcgagagcggaaacaatatggtgatggcctatggtaaagtttgaggcgagaagaggcagaataataatacactttttcggaagaaaatatgaatggacgatagagccgagatactggagaaatccaatgataggcctggtcctctggctttctttgccccttgtcctttacacggtcgcagttgtggcatttgactaccagccgatcgccttgatgtcaaccctgatctttgctaatctcctgatcatgatggccgtgccatacaatctgcagacgatcggaaccggtcgccttagcttcggtccccacttcttcttagccgttggaggttataccgctgccctgctgagcagggacttcggcttaagtccagctttgactcttccagcggcatttgctgtcggggcactcgtagctttggccctgagtcctataacgataatctcgagaggagtgtattacgtgctcataacactgctgctgccgttcatactgggcgaaataacatactggcgctctgacatattcggagctgaaacggggattccgaacgtggaactcttattccctaccaccggcaacgtgacggttgacgtaatgatcttcgtttacgtctcgctcgcgatagcgcttgttcttctgtttttcgtcgacagaactctcaggtcgagatacggcttcatgatgggtgtgataaacgaggacgaggacgttgcacagtcttacggaatcaacgtgaggcagataaagatcataaccttcacgctgacgagcggagcgatggcaatctgtggatggtttttggcacactaccagggatcgttctctggaccggtgtggcttatgccccccttcttgatcttggtgcttcttactgcaaccctgggagggaagggtgccatttacggcgtagtgataagcgcctatttcgttgcgacgatgagagagataacgagaataacctttggagagctttcagtggtcgtcttattcctgatactactccttctgctctacttcctgagagagggattctggggactttacaggaagaggagatacagagaatatgagccatcgatcaaggttaggaaaaaggtctgaggtgatcggatgctaaaggtgtcaaatctaacgaagaggtttggaggtatcatagcggtaaacgatgtgagctttgagatcgaaaagggagaaatagttggcctcatcggacccaacggagctggaaagagcacactgataaacctgatctctggcttttacttcccagacagggggaaaatagagttcaacggacaggacataacgaagaagagaatgaatgagagggccaagctcggaattgcaaggacattccagaatccgagagtcatctcgaacatgactgcgcacatgaacgttctatacgcaattctcggaagcaatcatggcaaaaaactctcgatgacggaggcaggtgctgaagctatatactacctcgacctgttcggcctcctgaaaaagagagatgtcctagcttcggacttggcaatatacgagctgagacttctcgagcttgcgagggcactggcattaaaaccgaagcttctgctcatagacgaggcaatggccggattaaaccccgcagaagctgaaagtgtttcaaagctgataaataggataagaaacgaattcgatctgacgatagtgtggatagagcacgtgcttaaggttttgatgaaatccgcggagagagtccttgtgatgcattacgggaagctaatagcagacggaaagccggaagaggtcgtaagaaagcctgaagttgttgaagcatacataggagaggaagaagtatgatgagcgtcgaggatttggaagtatggtacggaaaaatactggcggtggacggggtatcgattaaggtagataagggagaaatagtcggcctcatcggacccaacggagctggcaagagctcgataatgaattcgatcataggtgcggtgagaccaaagaagggaaagatcacgttcaatggagaaaacttgatagatcttccgacctacgagataataaagaagggtgttacgatagcacctgaaggaagaaagctgtttccattcctcacggtagaggaaaacctcctaatgggtgcaatcaacggcgatacctggaaaaggagaaagagttctctcgagttcatttattcgaagtttccaagactcaaggaaaggagaatgcagatggcgggaactttgagcggtggagagcagcaaatgctaaccatagcaagagcccttatggcctctccaaagctgcttttaatcgatgaaccaagccttggattggctccaaaaatttccctcgaggtctataggctcattaaatccctaagagacgagcacaagatcacgatactgctctcggaccagaatgcgaggagggttctccagataagcgatcgagcatatatcatagagaacgggaggataaggatggagggtgtgagtgaggagctggctaagaatgaagatgtgaggagggtgtatttgggcttatgacggactatttcagctttgtggaatctttatttaaggaaggagagaaaagagagccgccattgaagggtttgaaagtggtcgagctaacacacttcgtctttggtccaaacgtggggagaattctgtcccagttcggtgcagaggtcgtcaaaatagagactcctggcgagggagagcgatacagacttgctgcaatatttggaaggtactacaacagaacaaaccttgtctatgggattcagaatgcgaacaagtacttcatcgcggcggatgcgagaaatgcgaaggcgagggaaataattttcgagctcgcaaagagagctgatgtctttgttgaaaacttcagagcggggctcgctgatctgatgggagtaggatacgctcagataagcaaggtaaatccaaggataatctacgttagctgttccggcttcgggcagtacggacctttgagcaaagctccaagctttgacgttgcagcacagggtgtagcttcgctggcggtaaaaactggctgggaagacgtggaagagttctacaaacttccagactactttggagattatctgccatcgatgatggtcgttttcgcaattctgaatgccctttattacagggagaaaactgggaaggggcagtacatcgatgtctcccagacggagagtttacttagattcctgtatgatataacctatttcagcctgactggggaagagatcgggaagacgggaaacatagatccctgcgcaagcccctctggtatattcagaaccgctgacggcaaattcgtcgccattgcagtaatgaccgatcagcaatttaattctcttgcaagcgttgtggaagggttgaagaacggcgaaagattcaggaaggataagatcaaggcactcaaccagatcttggaggattgggtttctaagcacaagctcgacgagatcatggagtatgccaaaaaactcggatttccagcttcgcccgttcttgaagacgttgaagttttcaatgacccgtggagatgggagagaggcagcatagtaaagctgaatgaccgcctttacggtgaagtgccagttcccggaccattcgtggctatgagcgcaacccccgggcagataaagtggctcgcaagacccgtgggatatcacaacaggctgatactcaagaagtggctggggatgagcgatgaggagatagagaggcttgagaaagaagatgttataggttactgggacgagcagccaggctgcgctccaccacccgtctgggaagaggaaaaggatcccgtgttcaagggtgagaaagatgagtagagacgaggcattgatgaagctatttgcggaggacaacaagccttttgcactcgaggacgtcagggttgtggaaatctcaggggaaaacttcgcaggagcgatcgcgggatcacttcttgcagagtttggggcgaaagtgattcgtgttgacttcgacgacgaggcaaggaaaatttcaccgttcggggcaaatctggagggcattggaattccatacctcgttgagagcagaaataaggagatagtgaaattcgacgagagtgtgagagagcagatcaagtcctgcgacatattaatagacggaatgaagccaggatacctcgattccattggcatcggatacaggcagctgtctgaggtaaatcctggcctgatatacgtcgcagtttcgccttacggtcacttcacgcagaaggcaagggagttcgcaaacgttccggactccgacctgacggcgcaggcccataatggatatccttcgctcattgggaacccatacctcacaggaaagcattcctatcccttaagagcagggatgtgggcagcatgggctatggctggagttaatgctgcatgtggagccatgattgccctgattgaaagaagaaagagcggtaagggtcagttcgtggacatcgcaactcacgatgctttggctgtcatccattccttcccaactatagttggtttcctcttcgggaaatcgaggacgagatacggaacactggactacattctctacccctttggctattataaggccaaggacggctacatagctctggccacgccaacggacccggacttcagggctctgctcaagatcatcaagaggtgggatctcgagccggattacaagtacagcctcgacaggatttcggacgatccggagaggcttagaaccctcgatgaagccctgaacaaagagcttcagaagtatacggtgaaagagctcgtcagcaaggcgaggaaaaagagtaagggaaagctcataggcagattcctcggcgctccggttatagtcaagctgaacgggctgcaggagcttttgaacgatccgcactggaggatcagaaaaagcttcatcaccttcagcccggacggaaaagaggttttaatcccgaataccgcggtgaggatgtcggagactcctccgaggatccttaaggtcttgggcaaagaagtcaaaaaatcctaattttttcaaatttgtttgacatcttcaccaaaaaattttagtttcgaggaaaacctttgatatgaaggtctttgcctcgtggagcggtggaaaagacagctgtctggctctctacaaggctttaaggagcggattggacgtaaaaatgctcctcaacacgattacggaggatggaatgtattcccgatcccatgggattcgtgcggaggttttaaaaaagcaggcggaggcgatcgggatcgagatccttcagataaggacatcttgggaaaactacgaggagaacttcttgcagaccctttcgagattgagggagcttggcttctcctatggaatttttggggacatagatcttgaagaacacagacagtggatcgagaaggtttgcgagaaagccaaaataaagccttttttaccgttgtggaagatggggagggaagaggccgtgagggaattcataaactcgggcttcagggccgtagtttgctccgtaaaggacgggattctgggcagagagtggcttgggaaagagatcgacgctaacttcattgaagaaatgaaaagagtgtccgttgacgtctgcggagaaaatggagaatttcatacattcgtttacgatggccctatattcagaaaaaggctgaaattaaatttcggagagatgagatcacggaatcgctacagcttcttggagattgacgtctgtctggaagaaaaagatttaattacatgagcctacggcgagcatggacttcgatgccagtgcatatatagagtcaagtcttagggctttgagggagacactgaaacctgaagatagcgacaaagtgctcgctgcatgttctggcggagttgacagcacagtggcggcggttctggtagcaaaggcacttggaaagccgataaaggccgtattcatcgacgacggacttcgcagaaaaaatgaacctgaaagcgtcgtgaaactccttcgcgatcttggtctcgatgcctttatttacgatgcgaaaaaggaggttcttggtgctttgaagggtaagttcgatgccgaggagaagagaaaggcgttcagagatgctttttacactgtgctcgggaaaatcgtaagggaaatgaacgcgagatatctcgtccagggcacaattgctgcagacatcgttgaaaccgttggaggagttaagacgcagcacaatgttctcgagcagctgggaatcgatacatcgaggtacgggtttaaggttatcgagccaataaaggatttgtacaaacctcaggtgagggaggtcgcccggaagctcgggattccaaaggaaatctctgaaaggagggcatttccagggccaggatttgcgataagaatcgttggtgaagtaacctgggaaaaacttgaaattctcagggaggcgacgcagatagttgaggaggaaacaaaggacatagaggcgttccagaattttgccgtccttctggatgtgagggcgacaggagtaaagcaaggaaagagggtttacggttacgttcttgtcgtcagggtagtttcatctgaggatgcgatgactggaaggtttgttgaaattccctacgaaaggctgaggagactgtcgcaacggatcacggccgaggtgcccgagataacgagggtagtctacgacataactgacaaaccacctgcgacgatcgagtacgaataagagtatgtatttcgagctctggatagacagttcaagaagggaagacgtgataaggaaactgagatcgctctgcgaggaagtctgggaggtctcagggcattacgacctcatagtccgcgctgacagcgaggagaaggtaaaggtcgacggagttctgagatggaggaggcactacacctgttagctcactctaagacctttgcgaccatgtctccctttttaacgcctagatcctcagctactgcttcgcatttcgccatcagcaggaaggctatcggcctaaattctgcctcagaaaggcattcgtagtttgccattcctttcgctatgataagatctgcctctttcagccttttgagcgtaatttccggtagctcttcaactataacccctatagccccctttccatttgttaaaacctcgtcggcaacatcttctattcccgcgagtcttgcatcctcgatcgtggcatcgttgagtatgggttttcctctgaccacgaccgtgagctttttgcagaatttttttagttccttcatcagaattctgtcaaaataaatttcgcccgcgttgtctgtcaggtaaactacgttttttgcaagctttggtattcgatccacgtggtctattgcaagcctctggcttagcttctttctgaaattctgctcgaaatcatcatcaatcctgtgccctaagacgccaaagtcaaattcattcccaactattgcgcactttacagccgtcctcaatggatctgaagactctttaacgatactctctgccagccctacgacctttgaagagctctcgttcgctctcctcttcatctcgagataggggtcctcgcaccttaggatctcgtataccctcctgtggattttggttgccagatgggcgtttacaggctttctgcgatattcttcgcttaaaattttcagcgcctgctccatgcacttcgagatgagctctctgtcatcggtcacaagccttgcctcgtagtaaaccctgttcagcaagcaggaagggcatatcggatgaatcttcatctcacaacctcttatgaatttccttcagcttccttgtcccctcctcgaatttctctacagcatctttcagcatcgaaaggatctcaggatggtctttcagcttctccaaccactccttgtatttttccacgtgctcctcgttgtgctcgatccagtgttcgagcaactttttgaatctctccatgtattattgcagggatatcgggttttaaaagattcctagaccacttccaattcgacctcttcacctctgtcattgtagcacttccagtccttcgggccaaaaggatagcatacgatgatgtggtatttaccgtatcgggtgaacagaaagaaatcctcctcgctcggctcacagcaaggcgagggatggctatgcactgttccgtgaaccctcattccgagggggagcatatcgagatgtatgatcgcagaactctcgcctgccatgaagggaagaaaaataagctcctcaatcaaatctttttttcccgacagcaacgctatgaactcattcggataggaatcccttgcgattgcaaggatctccttcagaagctccttagtgatcttcatatgaactctctcggatccgcgatctcgccctttatcgcgctcgcaacagcggttgcgggagaggagagatagataaaagcttcaggattccccatccttcccttaaagtttctgttctgggttgaaatacagatctctccatcggcgggaattccctgatgaattccgacacaggctccacagccaggtggaaggattatcgctcccgcttcgactaaggctgaaattattcccatttctattgccttcaggagtatccttctgctggcaggagcaatgatgaggcgaaccccctcctttactctcctacctttgatcagcctgtaggcgatttcaaggtctgaaaggcgaccgtttgtgcatgtgccgaggaaaacctggtttactggagtacctgcgacctcttccacgctaacgactgcgtcaaccgagtggggctttgcgacaacagggggcacctcttcggcgtttatatatatctcgctctcgtaattcgcttcactgtctgccctaaggactctaaagtcattctcccgacccatttcagccagaaacagtcttgttttttcgtcgctctcgaaaagcccggcctttgcgccgcactcgatagccatgtttgcgatcgtcaatctctcctcgatgctcatgttttccacgcaatccccgtggaattcgagggctctgtagtcggctccatctgctccgattttccctatgaggtaaagcatcagatccttggcgtaaacgcctctcggcattctcccgctgtaaacgaccattatgctctctggaatcctgaaccaattcttgccaagggccattgctactgcaatgtcagttgaacccatacctgttgcgaaagcccctaaggcaccatatgtacaggtatggctgtcggctccaacggcaagcattcccggcttaacgaacttctcgaccattagctggtggattataccctctcctggaggattaaaatcggctccaaactgctttgcaaactcgtagatgaatttctggtcattgctgagctctcttctcggtgagggagcagcatgatcgatgaagaaatgtgctctctctacagccctgtatattccgatctccttcagctgtcttattgccaaaggtgctgtaccatcctgaagggctatctggtcaatttgggctaccacgatgtctcccgcatacgcatctttaccagatttttcactcaggatcttttcggctatcgtcttcatctctacacctctcagcgaaaggcacatcttcggttaaaataggttgctaaaatcgattttttcgccttaagagcgcgacgtagtggaatggaagctccgagatcttcaagacctcgtaccccttgcagatttccagtatctcttcgagcgaaatcctcttctctgttggtggaccgaaatcgctctgaacttttttccactccgtaatgagcacaaattccgccattcttgcccagcggaggtattcaattggatcctccatctcgtggagaacgttggaaaaaacgacgaggtcgattttaaaatcgatcttcggaggctcggaagtgactatgattccaacgttcctcacaccctcatctttaagcctttgctgaagttttttggccatctcgtagcttttctccaccgcatatacctttttaaaaatttttgaaaggagaatggtcaggtagcctgttccagcgcctatgtcaaacgcgatatttttcttcatgccttcaataccctcaattatccctataactttgtctggtggaaataatttcttcctctggcttgattcgagcttttctatcttctcatgccacttcatgatgcttacacctgcctgcgcattttaacttagctaccaaaccccgtagatctctctgccgcatttcctgcattttcccctgcggatccgattctcgataaccctgcatccatatctctttaccagcaattccccgcagtcagggcatagggtattttcgtgccttcctggcacgttgcccacgtatacgaagcttattccttcttctttcgcgatcctcacggcgagttcgagcctctcgacttcggttggtctccctctgagaaggtaggcggggaagtatctggtgaagtgtagtggcacttcggggttcgcgtaccgtaaatgattttcaacgacttctcttatactctcctccgaatcgctcacattggtgacgagcagattcacgatctcgacgtgaattccaagcttcagaacctctttaacaactctccagacgtatctcacatcggcgttcaaaaatttcctgtaaacctcctctccgcccttgacgtcgacctttatcgcatcaagcccgacttctctgagcttcttcagggctaaaacgctcatgtaaccgttcgagacgagcgttgtgagcagcccattttcctttgagattctgaaaacgtcgacaagaaattcaaaaagcagtgttggctcgttcaggctgccgcatacgctgatatctcctctctccagggctcttttgactacgtaatccggacggacctcttcaccagtgggcagccctctcgagatcctccagttttgacaccacgggcaatcgaaattgcacgagtatgtggcaaatgtcatgctcgtactgccgggcttgaagtgaaagaagggttttatctctatgggcctactctcaagagcgctaagcctgccgtaggtcaaggtgtaaagtctaccctctttattcacccttacgccgcagaaacctttttcaccgtctccgatcttgcaaaatctgtggcagagctggcagaggacgtaatttccgagatttctgtagagccccgcttctctcataaacgggtttcaccatgtaagagtcatctctggttttaaaataccgcccccgccgggaatcgaacccgggtctgaggctccgaaggcctccaggatgtccgctaccccacgggggctgctcttctttcagtggaatcgcaata

>Bin11_12

caccgcaactggaagcgaagctgtgtgacagtgattcagacttgaaagcacagctaatgctgtagtctttccaccaagacccattggtccgattccgagttcattcaccgctttgagaatctccagttcgaaatcgttcatgctgtcaagacttctcagcaaggccttctttgcaagctttgcggagacgtcaaaacttccgccaatacccaagccgatgatgatcggcggacagggcatgcccatggctcttgcaacggtttctacgacaaaactctttatcttcattgtttccattggcaggagcattcgaagctgggacatgttctcgcttccagcaccctttggcatgtaaacgatgcggagcctgtctccttcaacgagatcgaggttcacgacaggaattccaatgcccgtgttatcgcccgagttcttccttgtcagcgggtggactgcgttgggccggagaacggatggcgtaagccttctcacagcatcctctacggccttcctgatgtcgaaatttagcctgagctctcttcccatttcgatgaagaagaagggaattccagtatcctggcagatcggaagcctggatcttttcgcgatttctatgttctggagaatcacttcgaggttcttcctcgctatttcgctctcttcattctcgtaagcctttttaagggcattaacaacgtctgaagggagctccgtttgagccctcactattagatcggaaacagcatcaaccacgtcttcataattcatctctctccctcctcacgaaacatccaagttcttcaaccttcgttatatcctttctcacgtatcggaatatcctcttcctcagctccacattcgggacatttccgtagcttgaaagtgtcctcagtaaacctttttcgatctcgaagccttcagcatcgtagtccgtgagaattatcactctttttccgtttaatttttcagctgtgctcgaaaagcctgaaaaaatgacgatctccccctcaacaccgatttcgcgcagagctctcagatccttgaccccttcaacaactaccacccagccatttctcgaaagctccttaagctcttctattgcattaaagaactcctttagctcttccatatcttattcctggttctatctgaagcgtgatatctgcgataacacccctcttcatcccacctttaaccacggtgaaagagctgaagtcgagaacttttatctccccaacgtcttcctccccgaagttcttagctatctcaacgactttccttttagccgcttcaatgaggtcctcatccttcggaatgctccc

>Bin11_13

agttcctgaatcgtgacatctcttgtgccgaaccttgagcaccctacaccaacaacaccaacattcattgcactaacttgctcaataaattaaaaagcttttcgcattttacgaaaataaaagtttaaaggatgatcgtcctcctgtgatccttgtgcgttttagaggcgatgaaggtgtggagatggtttatgcgaattccgtgcttcttgaaccatcccatgaactttcttctctcttctctgaactcctcaacgtccttgtatcgaacaatgaggcaaaggtcgtactgggagccaacggtctcgaagacgttctcgacgaaatccagttttttgaagtgctctatgatctcgtcgagctttccctttgactctatgtcgaagtctatcaggatcactgcgagcatgttaagaccaagcttctggtagttagcaacccagtaaccttctttcagctttatcagatactcctgctcttctaaggactttatcctgttctgaacagttctgagtgttacgccgactttccgcgcaacctcgctctgggtattcccctgaagtatgtactggatgatctttttatccacatcgtccagaacctttcgcataatgtgaaaatcaatggcgaatatataattcttttcacggattgacgaagagccatcccttgcccactctaaacccccttttgacactctcgagcacaaaaatcctcgccttccgaactgcgaactcaaggtcaccagtagccagatagcaggctaacgctgagctgtaaacgcatcccgtgccatgaaagcttcctgtaagcagctcagcctcaacgctgtagatttttcccttatcgcaaacaatgtctctccccttcagctctccacccgttatcacgactgagcaaccgtattttttgaataactcaagtgcgagctcttctggattttccttcgagccaaataccttggcttcagagtagttcggcgttatcacgctcacctcaggcataagaagctcgtatgcagatttttctccaagcttccctcccacgcttgcgaaaagcacgggatcgagcaccttcgggcactcaagcttttttatgcaatccagaacggctgtcgcgacttcggaattggggattacgccgatcttcacggccgaaaattcgaaatcttccagcagagcgaaaaactgttctttgattatctcaggcggagtggggtggacagaaaaaagatttctgctgttctgaactgtcagagcagttattatcgaacagggatgaaagcttagcgcttttgcagtctttatgtccgcattagctcctgccccgccgctgggatcgagggcagaaattgttaaaacagttttcataaaaaatttagaatagatccttgaggtttatcaggtgctttccgagctttcctccgtaatttccagcggtgatcttcacgaccccgggaatctttgtcgctgcaagtatcccgaccttcattgccttcttaaccgcatcgatgttgagcccgtttatgactatctcgtaaactgcattcacgccagccggaatctgggatccctctaccttttccctgagcgtgggggcaaacttttcgtttgtcgaagccttcaggaatttgtatttgtttgctccgacctttgagccagaagctacgattccgcctgggaatggagtgataactccctcgacttcagcgattgcatctacagctgctttggctgcggctaatgcagaaggctgattttcgcccattatgaagaagtttccgcccgcgattccgcttacatagccaaagtggctctcaactatgaactccccctgcatcataggtatggcatagcattttcttccaccgacttccgtctccctttcatagccatctccaaagaactttagcttgaatcctgtgtcaaacttatcctgggcatccggcaatccgttgaaggcagctgcagttggagcggtaagcacacattgcccaagtctcgcgagcagctgctcctcgagccctttcttgctcatgtggcagatctgtatgtagtatcccgccctcccatcgggggtttcagatggcaaggccttcttttctatcccggcctcagcggggcacatgataacgctcgtcccgaaacctgtggcttcatttgctgcaacccaggcccagtaatagtcgtacgctgttatcaaaaccctcgaaatctttatatcgaacgcctcggcaaaagtttcctcaacctcaacaccattcaccttcatgcgatcaccagtctcaattcaaaaactaagatttaaatcttttcaatttccccgcctcccgattttggaaaacaccaaaagttttaatattcaaaaatgtttaaagatttaatatggcggagaaggtcagggatagggaacaccatgaaaggcttttcaaggcatcaatgagccctgcaaggcgcgaaatagttcgcgcaataggtattcatggaaaaagcatggatgagctgaaaaaagagcttaaaatgtccgaattccagctcaaattcaatctcgactggctaatcagggagggtttcgtaaaggaggaagatggcaaatacagactgactgaagagggtatagaactgcttgatgttagctgaaagtgagcaaaaagataaaaaattgggtttttattcgtatccgaattttttgagcatctccgcaagctttccttctttcagcgtctttgctggttcgaatatcttctttccgtagaatttcgccagctcttcaagtctctgcgcaatttcgacatcgctgaagttctttgcaagttcgaagggcccgaatggtctgttgtatccgagcttcaatgccgtgtcaatgtcctgcggtgttgcgacacccatctcgacaagcttcacagcttcgtttatttcgacatacatgaagtccatcgggtttaccttgtcggaagccttgttcaagtcgatttctggtctccccttgctccagtcgtaccatccttttccagtcttggccccgagcttcttttcgttcaccatcttctccaagaattcgggcggtttataatcggggctaacagtcttttcgtagtacttcatcacatgatactgcgtgtcaacaccaacgtagtccatgagctcgaagggccccattggctggcctatgctcttcatcttcgcatccacttcttctggcgttgcaacacccttttctatgatgcccataagcaacacgcttcccggtgcgacaaccctgttcgcaatgaatccgggaacgtccttttcaaccctcacaggcacctttccaatgctcttcacgaattctaccagcagattcattacttcatcgcttgtcttctcccctcttatgacctcaacaagcttcatcagaactggaggattgaagaagtgcagaccggcaaacttatctggcctatttgtagcttttgagatatcggatatgctcatcgtagatgtgttgctggtgaatatgcattcaggcttcgccaatttgtcgcattctttgaatgtc

>Bin11_14

ggcctcctgggacttcattccagatttactcgaggatcctgccgtatatccgccagaagaggttcaggagaacatgtggctcatgccctcgcttagcgacgaagagagggaaaagatcgagagattaatgattgaagtcaaggcacagtgactttacaaaatttttcaagcaagtctcagaagttttggagcggattcagagggaaagaaaaaataagtaagatcaatttatttcacctttaaaaagaacgttcagcctttcgagaacctttttcgctttttcgttgtcatcaacccggaaaattatcaaggctttgtgcttttccgaagtgaacgcgtaaacgtactcgatgttgatcccctcatcgcctaaggccttagaaattctgtgcagttctccgggcttgtcctcaacctcgacagcgagaacctcgttaagcgtgactgtaaacccagcagacttcaaggcgttgaaggcttcttcagtcttgtccacaaccatcctgattattccaaaatcgccagcatctgcaatcatgaatgctctgagattcactcccctatcgtaaagaaccttcgtcaccgccgcaagccttccaggcttgttttcaacaaaaacagagagctgttttaccataaaaccacctcaaatcctcctcttatctataactctcttcgcctttccctcaaatctctggagggttccgggattcacgagctcaacctttgccgtgatccccagaacgcttcttagtctctcctcgacttttctcgtcaaattcagtatatccgctggcctatcaatcgcaaacttatcgctcagctccacctgaactgtcatctcgtccagttcttcctttctctcgagtatgatcatgtagtgctctccgacctcggggatctgcataagcacgtgctctatctggcttgggaacacgtttacaccgcgcacgatgagcatgtcatcgcttcttccgagtatccgcattattctcggatgagttcgcccgcagttgcacttttccgtttcaagaatcgtgatgtcgccagttctccatcttaataaaggcatggcttccttgctgagagttgtaactaccagctctcccctctcaccctccccgacgttctctcctgtcttaggatcgattacctcgatgaggaagtgatcggcccatatgtggagcccatttctctcaacgcactccgtgaaaagcggaccgctgagctctgaagtgccgtatacatcgtaggccgtgattccagttttatcttcaattcttttcctcgtttcctcgctccagggctcagcgccaaaaattcccattctcaaattcgtttctctcaaacttgtacccattttttcagcatattctgccatgtaaaggaaataagatggtgtgcaggcgatcacagttacgcccaaatccttcatcagggttatctgcctctcggtgtttcctgcagatacaggtaatgcagtggcaccgattttttcagctgcgtaatgaaatccaagaccacctgtgaaaaggccgtagccataggcgatctgcattatgtcttcgttgctcactccacagctcacgagccccctgcaaaggctctcaacccaaacccttatgtcgtcttcagtgtagccgacaagcgttggtttgcccgtggtgccgcttgaagcgtggaatctgactatctgagaaattggaactgcaaacattccgaacgggtagttatcccttaaatcctgctttttcgtaaacggaagctttttgaggtcttcaaggccacggatgtcttccggatgtattccgagctccttaaacttcctcttgtagaacggcgaatattcatatgcattcctaaccaaggctttcagtttcttttcctgtatccgcttcaaatcctctacgggcattctctcaatcgctggattccagaacatccttccaccccacctatatcaccatttcctgcgtttcaagctcgatctcctcacatcatagtcaactgtccagattataaaaacatcgttggtatatacgaagacccacagcggagccaagcttaaggaatcctctcggagtaatttcccgtacctcgctttcaagaccttctgagaggcaaagagttcgctgatttcatccttctgcgtatccatcccaatcttccaacgggaacttcagaaaacttattaatagaatctcacgcggatgagccataatcggcaaaaaatagaaatatactccaaagcacggataaaaacatgaagtccattacagttcgtcttaacgagagtatggctaaaaagttagacgaggtcgcgagaaagactctttcctcgaaatcggatgcagtcagaagcgccttagcagtatacttttcgctacttgacaacataggattttatttcaagccatcaattcccataaagaacatagacatggaatttgaaagaaacgccgcataccttgacctgggcaacttcatctctgttgcagttttaaacgtcacctacggcggtgtcggagaaaaggagatggatgaaaaagtggagttggccttagtttcggaagtgatggccaatcaactgtttgtcgagtccgcatgtagattcatagacgccaaagccatccttatctccaccggcaatgaatacgagtactccaaaaaatttcttttattgttttctaaatcaataaagcgaaggtttaaggcggagacttttctgatcgatcatgaagaggtcttcaagacgcatcagacttttctatctgccacacttctgggaataagggatatgagcctgaagaacgtaccgaaacgcggtgatgcgatttatttatggggaaaggtcgtaaaaaaagaaattaaatttgaagacctgccagatccgttagaactgctgaaaattgtggaattggtcagatccaaagttgcaaatgccatttttcctgtaaaaatggatggggttgaaagtgttgctaactacgctgcatcgatcgcaggcggtaagctcatgctttatcaaaatcccggaggatgtcctgcgaccgctgttttgatctgttcaagcaacgacctcgaaaaattcggatgcagaaaaattggagagataaactaaggttcggatatctcgtaaacctttcttagcctctccaatctcaaaacattaaccaaatctccccttcttataactttaaatgcgtgctcgattacaatcctgtaaa

>Bin11_15

aaccaagccagggcaaaaaaggcttaatttggctttttcgctcaaaaaattggccttggaaagaggaatgaaagcgttgattgtctatatcaacgaaataaccccggagaaactgatgaatcttccctttgatttctacgtgaacaccgcatgtccgaggataagctacgacgactacaaaaaattcgagaggcccataataacgccgcaggagtttgagtatctgctaaaccgtagagaagagatcgggcttgacgaaatagagtgaatcaacggatatgcgagggtttattacaccacacgactaaggatttcgatgaatgtgatcgaactgatcgacgttcataaaatttacagaaccgaattctatgaagttcacgcaattaacgggatttcaatggagatcgaaagaggagaattcgtggccataatgggaccctcgggcagcggaaagagtacactgttgcatctcatcggttgcctcgacaaaccaacgaaaggggaagtttttatcaacggaacagaaatttctaaacttggcgatggtgagcttacaaggctgagaagggacagcatagggttcatatttcagcagtacaacctgattccaacgctcacagcgcttgaaaacgtggagcttccgatgatcttcaagggaatcccaaaggaggagagaagaaagagggcgatggatctgctcaggcaggttgggctcgaggagatcgctgagagaaagccaaacgagatgagcggtggacagcagcagagggtggcgattgcaagggcattggcaaattctccgaccattcttctatgcgacgagcccacaggcaacttggacagcaaaaccgggaggcaggttatgggcttaatcagaaaaatgaatgaggagcgcggtgtcaccgttgttctcgttacccatgacttctcccttgccgattatgctgacagaataatcaagctcagagatggaaagattgaggaaatacaggatagaaaaagtgcaaatgaggagaaaatttaaaaaatttttacttctttggaggccagttcttctccatccaacctttaatccttccggagtcaattggacccacaagaactttccagccggtttcatcttcaattgaaccctgaagccttgcagcaagccctgggataacgagatatctgtgattcaccttctccgcaacctttgtttgctcgataatctccttgaccttcgctgctgtgagctttcctccagcgacgcttgcctcaacgccaatgccctcagtgttcacgacgagcaaccatcccttgatcttcgcactgttcagatcgttctccactgtatagtacgtcaaagcaaagttcgttgtcaggaatactggatcctcgggacctgggctgtttatctccttcagacctgcagcaacctgcactggcgtcctcggatctgtgtatatgttgaacctaagcgtcagcgttggcatgacgacgtgctggtcgatgctgtggaatatcatggcatctccgtacttcacgatgaacattcctgcgatcacggcttcccagtaagatttcgttacttcatctccctcgatcagccatgcggagatcggggttatcagaatcgggtatgcaatgtctttgtccccatcgagtatcgctttcctacgaagctgaatcaccctctcaaaagttccctgaagcccctcgccaagtggttcggtgacgggatcgaggacgatttcctttattcccgcagacttgaaggttactgcgatgctcttgagaacgttcaggtcctttgcccttatcgcaactggaaccttgtagtcaagggcgagcttcatgaactccttccagtttccttcggtcgctgcgtagatcagaggtctctggttagcgagttcctcaagcgcagccttcatgcattccggattgagtgaaatgagaaccattggctttccgtagctcgcaaccttcttggcaacttctctgaacctcttgggatcgtttgatgtgcatctcaccgcaaagccgtcgaggttcaaatacttgccaacgtagaacttcttgtagtccacaacccgtttgcatctctcttcgatcttcttatcgtcaagggtatcccagacgtcgaagaagaaggctgttgggttgaagaacgtcagctcgtgcctgtggagcacctcctcgccaccaatcttaatggccttgtcgccaacgcccacgatgacttcggcgatctctggagaggttagctcaacaagcttttccagtttttcctttaccttcggatccttttctgcatccctcacaaggggtttgcagtcgtaaacctttgccgagcggtcaaggatgtgggaggcaaagctcatgcaggtatcgtagccgcactccttgcagtttgttcttggcaggaagttgtaaacttcaagcggactctttaccttcattctcacacctccgcggtgatccaccttcctgcgtctgcaattccacccttcgtctttcctccgagagtgttgaagatttccttaagcacagcaactgccaatggatgcatcatcatgaaaacatccacgcctgcgagtgagagcgtgagtccagttatgatctcccagatcgggcctctcagctctctcggaccccaaggtgtatcgccctccaacggggaatcagtcatccaggcttctcttgcaccccatgcgttggtcgttccgcttgagatcgggaagttgagatctgtgtcccctttcaggccgcttattcttattctctccatgttcgtgaatgcatagtccaagccatatcccaaggccgcggtagttggatccattacgatgctgttcctcggcatctgggctcttttcagcagatatctgttcagggtcttctggttgtttatatccatctgtgtccagctcaaaacgacatgtccatatttcttcgctgcattgcaaattctttcccaatccatatcgagcgttgcgcttgcaagcatcactctctcaccctcgcaaacctctgcagctttttcgagaacctcgggatctttgtccttgttgccgctgccgcctatgatgatcggacacttgaccgcctgaagaacgtcttcaaccacctttacagcttcgcttgctggcgtgttctcgagcaaggggtctgtgctcacaaggtggattgtaaccatgtcagctccgaacttcttcacgcacttcttagcccactccgatgggcttccgacaacgtcttcgtagtgtaccttcacagcttttgcaagcgatggcgggcgatcgaagacatctattgctattgcaggaaggttgggttgcggtttatcgaagatgtagaatgcaaggcttttctcgccgccaagttttactacatagtctcttgtacctccatctgcccttgtagcacccagaacgacctcctcgatgtagccaggatattcaacctttgtaggctcaaacttcgcctcgatcagcttctcaggaagcttcagctcaggaagagctggcactacgggctgcggtgctgctggaactgctgctggcgccatgggggcgacttggaatggtaccgggactccaagcattgtagaaattctctgcaaatatcccaaggccatgtttgtgtgatagaagaagtttctaaactcgtttaacagagtgtaaagggctgtgagggacgaaagatccactccgctgccgccttcgatctctatttcgagatctccctctatcttgacgtcctccagcctttcaacgttgtagctcttcaacaactccaaaagatcctcaagcttgtattttgccaatttctcacccctttaaaaacaactctgcaaccttactcattctcctataaacttcagatttctcatcaagattcacgatcggaatgcccttcaagtcgagttcaaccactttttcatcgtatggaagaatttcgagcagttttatcccctcctcatctgcaaattttttgatcctttgctctgcaacttcgcctgcaatgcggttcgcgacgagcattatgtctttaaagtttagtttcaactcctttgtaagctcttttatcctcttagcagtcgctaagcctttcttagacatgtccgtcacgatcagcacgtaatcagcgctatcgatcgtctttctgctgaagtgctccaagccagcttcgctgtcgatcacgacgaattcgtagtgccttgtcagcttttttagcaccccgcgaagcagggaattggcaaagcagtaacagccctcaccttcaggacggcccattacgaggagatcgtagcccctgcactcgcatatgacttcataaattttgccctcaagccactgttccttgttcatcgtccccatttcatccctgcttgtctggaatagctctctgatctcgccaagcgttttctcgacttcaacgccaagggcatctgccaaattgctgtctggatctgcgtctaccgcaagaaccttactgttctttttggaaataaaatggatgaggagggcggaaacgagggtttttcccgttcccccttttcccgtaactgctattgtagtcattcctcctcctcaattatgacctctggtatcttgatttcagctccaacgaggatcacctttatctttttgacctctccttccatcagctttcccccttgcccttcttcaccacgatctttccgatcgtgatcttcgcgtcctttattacgagtttgattcccgctggcgcagcctgtggagcaggcatttgcaatgcaggtagctggaatgctggcatctgcattggctgcatcgccatggccatcgtaggagctggctgagctgcagccggggctgttggtgctgctggtgcctcctttttctcctctaccttctcttcaaccttcttctcctcagcccatccctcggtaatcttctttccgtctatcttccttacgacgcccttgactacgggatgatcaaccttcttcaggaactccttgagctcatcgagggtctttgcattctcttctgtcgcgatcttgtccctaacatcgtcgggtatgtacttcgcaactctctcctttagctccttgggcatccagacgactctgtaccagccaccgtcagcctgaatgaacttcttgctcctgaagtagttgattccaatcccaaggaatcctgcaacctgctttccgccccctgtctgtccagccatcgtcgagaacgtgagcccatttggcgccgggcttgcgtaacccctgtgcacccatcctatgccatcaacttcgggcatgtagaagcctatcacttcaaagcatccacagctcgtgtgcgggaactcgaagaagctgtgcagctttattctatcgtactctccaccgctctcctgctttgcgaattcgttaactccgctgtattcaccgcctattggatccagcacttctccctttggaattgccctgttgggaccctcgggatcgacctttgcagcagctctgccatcaaaccagctgatagctccgcagagcgatggtctgtctggtgagacaacgcagacatttgttggagcaaagctctggcagagagtgcatgagtagaactcctgaacgtcctcatcgtgaagcatttcgactcttttgtccctctcttcgtatatcggcatcgcaacttccttcaacagcttctcaacaagtgctttgtccgtgatgtaaagggcctcgattttttctatgaacggcagctcattcttgtaaagcatcattgtggcctttgcaatctgtattaagctcttaagcccctttttaaccgagttcttactaattctgacccagacgtcgtatctctggttgagatgcatgtaaccctcgatgtagttctggaaatcatggtttctgcgctcaataactggctcaaggtctgtctcgactttgcttccggcgatgtagtagatcatcgcgtacggatactcctttccctcctgcatctcgtcaagatctgggccaacaagcgttaccttcatgtcctctacctgttccgctggcaaagctaagacgagttcgaacttcacctgttttggacctccgagttcgacgaacatgtcctcctttcgtattctttcgccctcgtacatcggagatatgtcgaatgggaatttctcctccgctcctgcactgaccttaacctttgcccctgcagggatttcgattttctttctttcgaccatctaccaccacctcaagtgttcaccttttacacctctctttgcgatcctttatttgtatttatcggtttttatcggtgccaaatctaacataatttctcattgaagagagacagaaaaagcccgctaagccttatctgcaggctctcgattttaatttaaacctaatattattttgaataattttatcattttctgtagcagtaaatattactaatttgattaaaattaataccaaaagcttaatatttgaagagaattaaaaaggccgtggaagagtgtctgaaagttatatacacgaggagaagcataaggagctacagggaaagggaaatatccgaggaggacataaaaaagattttaaaggccgcatttcaggctccttcagctggaaacgaacagccgtggcattttatagttgttaaaagcagggaaaagctcagggagctgagcgaagcacatccgtatggaaaaatgcttctgagtgcgggagccgcaatagctgtttgctgcgacccaaagctctccaagtatccgaatccgatgtggattcaggattgtagcgctgctacacagaacattctgctcgctgcaagggctttgggaatcggctcctgttggctcggagtttatccggttgaaagcagaatgaaatcccttgccaaggttttaggagttcctgacgaaatcgtcattttcagcctcgtcgctctgggttatccgaaaagcgaagacgaattctttgaggctcccgacaggttcaaactggaaaggatacaccacgaaaaatggtaagtgttaaaacacgaacgcgaacttgttgtttatgcacaagtggatcctagtggtcgaagatgactcggcagtcttggaagtcttggagaccatgctatccggaaggtatcgggtgatcaaggcaacaaatgggagggaagcagttaacagctacaggatgttcaagccagacctcgttctgatggacataatgatgccggtaatggatggaatagaggcaacgaaggagataaagagcaggtatccgaatgcaaagatcctcggcatcacggcatttgcctcgcaaaaaggcaaggagatgctcgaggctggagccttagagataatcgaaaaaccgttcacgaagaaaaaactgatcgagacaatcgaaaagtatctgatagattgatttacagctaaaatttaattagtattatattattatcgcgaagataatcagtcctatggtaaaatttttaatcttccaaaatgactatggatatggaaatcctgaagctctggatacatcaatctgactgccctttcataatcagttcggatgattttgaagtcagatactaccacaatctgatagaggtgaacgacaaaacgaccgtaaagagaggttacgtcacagcgaaggattctgaggaattgcaaaactgtcttgagtggctttttggaagcaaatcattgctaaatcaccagatttacgagaaatgtcctggctttgttttgatgcggatggacttgagtcacacccacgcaatggatttgctcttaaaaaacaagggatatgttgtgggacccttcattgcagaaaagggtaaagaagtctggcaggtgggtttcgacgatagggaatcgatggagaaattccttgatagcctatatcctgacgattacacgatactcaggcactgcacgctcccagcgatgagtgaaatgagtgaaataatcggaaacatagaggatattgtgctctttgtaaatacaataaaggagctcacaccaacggagaggattacactgaagagggcgatagagagtggatactacgaccagccgaaaaaagcgaacataaccgagctctcagacgcttttaacatttcgaaggttgcggtgtataaaaatctgaaaagggctgagtataagataatgaaggcgttactgagacttttgatgaacatggagctaaacgaagggggacttgatgaaaacaggaaaaaaatcctcaggaggttgagggactgaaatttctggccaatattttgaataaattggcctctatttcgaatgttctgtcgcaattcgctttaaaaacgtctgtttccatcaggtcaaacccgagaaatctaattccattgctctgtgccaatttgactatctcgaggactgtgcggtagacttcatcctcggaaagaccagtataatccagaaacctggcgccagtaagcgcgttttttgagccgatgtcgacgtctatggagatgtaaatgtacttcgcttcaagccccttaatcgcccttttcagaattttctgaattttcagtctttccttggggatgatttcgactccctttttttcaaagcttaggtagtgctgtaggtatttctttatcctcgggtcatcgatctcaaaagcactttgcggtggataatctgaaactccagcaacaaccagattttgaggttcaacgagctcctcttctaccaggaaatgcaagaaagagtccgcgttatagctttcaggcctccacattatgtatggatctctcggatcaaaaggcgtatccggattgttctccagatcgtactgtatcacgccgcatcttactgatggagttatggcatcgaaatggctgtccaagacgaccaaagccaagtcttcggggccaaatttctcgcttagaaacttcacgacccccccgctaatcgagtgatcaacgcctatcagggaaacaaattcggcatccttaccccttagcgtttcacagaccatgtttgcgtattcgaggcacccattggagtctataaaggccacaacgttttcgaccgttagcagatctaagtagctcaaatcgggtgccggtagcaaccacggttcaacttcgaccttgagagcatttaatttgattccttcctgtgccagtctaaacgcgattgcatcgtaaggatccctaaattttaggtttggataacccttcaaagcattctcaattttaaaatttatgacgtgctccctctcatctgggtcgagagggcatgaaattgcctggaagccatcaagcataaaaaatttaaattggagcaataacttgccatttttctctgatttctttccagctcattttctttgctgcttcagcgtatttgggggcctgataggccttcttccctacaataccaagcgggaacgtttttacatactcttctgttattggtccaccgccgcatatgaagggtatctctatatttttctccacgagcttttgagcgatcttcgggaaagcagacatcgtagtcgtcatcagtgcagttccggtaaccattacgggcttgtaattctcgacagccttcacgacttcgtcaacaggaacatctctgccaaggtctattacctcccagccatccgcgatcaagaaggcctttgcaaggttcttgccaaggtcgtgaaggtctccttctgccacatgcatcacaattctgcccttcttctcgacagcccctgccttgccagatatctcttcaagtatctttataccctcctgcattgcattcgctgcgagaacgacctggggaaggaagaagacgccgttttcgtatagttttgtaactgcgttcattccaaccgtcagacccttgtcgacgacttccttggggttgaacttcgcgagggcttcctttacgagttcggaaactttctgcgcttctcccctgaacacggcataggcgatcgacttgaatggctcttcagtcggcatcatgccctttatcagttcctcgggatccagcgccttcctcttctcttcctccttaagaatctccgctgcgtaagaagttgctcttctcaccttcttgcccttcttttcggcaaattctcttgcagcttcaacaagagccacgatgttctgaattggcgttatcggcggtattccacatcctggggcaagtatgtccaccccggcttcgagggccttcagggcttcagccttcacgtcctcaggctttccgaagggtaaagtctttttcgtggatacgtttccgacaagggcgatgtcgtcacctacaatctttctcgcctcaaccaagtcaacgacttcttctacactcaacccattgtatccgcactcagccatgtacggcaggatcttctttacatctccgcagatgtgcagtatcgatggggctccgacattctctgtgatgtctaagtgctttggaacaacgaaatgcttgaacatcgacggatcaagaacgtcgggagctgcagttggatctgggataacatagtaatctgccccggctttgatcatgtccctaactacaatgattaacgcttcggttgctatgtcgaggatttcctcaacctttcttggctgtgttatgcacatttccaaaagtctctcagttccaacgaggtgtccggccaacgtaaaaggcccctcaaaaccaacaagtatcggcagaaaatctcccacatttcttctcagtatccttatggcctccctaaccactggtattctgcctctctcgagaagatcttcgggcagagttacctgatctggcgagaggtgctttgaaaccgagggctgtctgacctccatcccccagtttatcccgcaacccagggcttccgcaaccacggtaaggcagaatgggacccttgcgacttcaagctttgcaagttcccataaagaagcgccaagctttgccattttctctggatccttgtgagcggcgggataaaaagaattaaaggctgccattgcatctctgatccccaaggttgtggctgacgatgctggcgtgtagtccacggaacccctcatcaatgccgcaaggaccctccttttcgggctcataagccttccccttttatttcttgccaagtacttcgttgagtatctctataacatccatcattttcatatttgaaccttttgcctccaatgcatctttcagatttctgtagcagaacgggcaggcacttgtcagaatttctgctccagtcccttcagcttcctcaactctcttcgcagctgtctcaagcgagtattctgggaatgcagctcttactcctcctccagcaccgcagcagaatgagaactttctggtcctctccatttcaacaagcttaacgcctggaattgccttcaagacctctcttggttcctcgtacacctcgcaatgccttccgaggtggcatggatcgtggtatgttacgaccttctcgaacttgccggtgaaattaacctctcccttctttaacatctccgcaaccacctgcgttgaatgaagcaccttcgcatggaaatcgattccaagcttgtccttgtatccctcgggcagcccttcctggtagtctttcttcaaagccttgtagcagcctgcgcatgaagttatgaaaccctcagcaccgtatctgtcaagcagtgcagcgttgtgctccgcaaattccttagccaagtcccactgtccagttctgaacattggagagccgcagcaccattcatcctcagctattgtgaagtccactcccagggctttaaatacatttacagttgccagagctatttgtttctgcctgtaagacgccgtgcagccgacgaagtagacatattctgccttctttggcagtgaattccttactgagctcggcaaccagttaagcctgtcggagtgcttttccatgtacggatttctctccacctttacgtgagcaccccatctcttaatggcgtctggcacttttccaaatctccaggcttccgctctcagcgcctctatcgttctcataatggctggcttgaagtccgtctcgcacatctgctggcaggctccgcatgttgtgcatgtgaagactgcatgcattactgagtccgtgtactgaagcttgttcttcagcagaccccttgcaatcgaggactttgacttgcttccaaagtaagactcgagcatgaatttatctccctgcgggcagccaattgcggcgaagttttccttttgtccatactcatagagtgtgcggcagtttgtgcaatgagtgcacatcgagatgtagttgtaaacttccgcaagcttctcatagtccttccttatcaatggatgctcggcccccattcatctcacccccagctttccaggattcattatgtgattcggatcgaggcagtcctttatcttggtcaaaagcattatccagcccggatcggcgtacttgtttattatgttccagtactcaacaggaggcttgtagggaatcatgccaag

>Bin11_16

aaggatgggaaggattacgattaacagtgtcaggaaggtttacagagacggaactcacagggcaagagagcccgaggaaacactccgctggatcgagcccaaaacagctgtagcgggaataacgaggctggcaaacattacaggactcgatagaatcggaattcctatcttctcggctgtgagaccaacagcagcggagggggcgatttcagtatattcaggtaaagggttaactgagacgctggcaaagatttcagccataatggaagcctttgagagatattcggccgagttcagaaatgaaaaaacggttaagggaaactacaaccagatctcgaaggaattcaacgctttagatcctgaaagcctcattctcccgagagacttgccctacaacgatgacgtagttctaagatgggtctggggttttgacattctaaacggggaggaaattctcgttcccgtaagtgcagtttatcatccctacactccactgagcgacctgcacatcttcagaacaaatacaaacggtcttgcgtcagggaacacgattgaggaggcaattctgcatggtctgatggaagtcatagaaagggacgcctggagcctggcggagctctcgaagaggggtggaagggtgatcagaaccgatagcccggttatcatggagcttatggagaagttcagaagggctggagttgagatactgctcagagacataaccagcgatcttaaagtgccagtggtagcagctgtttccgatgatgtcgtgcttaaagatccagctctgctcacaatcggcttcggagcccatccggagccagaggttgcatgcataagggcaattcttgaggtcgcccaaagcagactcgtgcagattcagggagcaagggaggacaccataaaggccgaaataatgcgaagagcaggatacgagagaatgaagaggatcaacagacactggttcgaaaatgatgaagaaattgaattgaaagagcttcctggattctcgaatgaggacataaaggaggacatagagtttactttgagatcgctcaggaaaaggggatttgagagggtaatagtcgttgacctgactagaaaggaactcgaagttcctacggtgagggtcatagttccgggactcgaggttttcgggatggatccaagcaggattggggaaagagccctaagtttcatgaagagatgaaggtggtgatatttacaggcccgagcttgagccatgaggaggctaaggagatcttcgatgaagcagaatacagggcaccaataaggcgtggagatgcgataaaagccttgaaagaaggaataagagttctaggaatcatcgacggagttttccaccaggatgtggccatatcgcccagagagatccttgaagtgatgagagagggggctatagtggttggaggaagcagcattggcgcgctcagagcagctgagctttgcgatctcggaatgatcggtgttggggagatattcagaatgttcagaagcggggaacttgactccgatgatgaggttgcggtcttaatcaatccggagacacttgaagcaatttcagaaccgctggtgaacatcagggcgacaatttcggcgctggtcaaaaaaggggtaattgagacagaactcggcaacgagctgattcggctcgcgaagagaatgcactactcgaagcgaagctacgaaaatctgctggaagaggctgttaaaagtcacctgttgagcttaggcgatgctgagaagcttctgaagatgatgagagagaacgcggtgaatttaaaaaagctagatgccataaaggtggtcgagactgtaaaggaaatatgctcagctgagaagttctaagatcgcggaacagcccgtgcatcgcgggcaaccagccctgaacttcagctttatgtcatattcccccttaagctctttgagggcctcataaacccttagcacgtgattcaaatccgttttctctgcagaattcggaggtgggcggtatggagcgatgtaggggtaaatgcccctctctgccatagccttgaatccgctgaccacgctctcaatgctctccccaagaccgacgattatccaggaggagacgttccattccccaaagatgtcgacagcgttctcccaggattccagataggcgtcaatttcgggcttacccgggacgaccgctttcctgatcctctcgtcgaaagtctcgacgtgaatgccgagcgtgtcaattcccgaggagtagaggagttcaaggtactccctgccaaccggctctatctgtgcgtgtatcgggatgtctatctcggctcttattttctctgcaacctccgcgtatctcaatgcgcccttgtcgaccagattcggagtcccggttgtgagggttaggtgcctcgacctgtcctcctcgtaggcaaccttcacagcctcagcaatgtcctctggagccttatccaggatcaaggtctcgggaacgttctcctcgatcacgcagaaaacgcacttctttccagattccacatggatgcatctcctcgtaacagcggttacgagggcgtttacaccgtcaagggcagcaaatctccctgcagagatgccctttatcctgtaatcgtaataccttgcaactggaagcttcactcttccgatcttctcctctcccctgtaaagccagaatccgccctctccctcgactattgcatatggagattcctttgcgaattcctgaactgtcggtgcggaaacaatcgagtcctttaccgcaaaacctattcctccaactggccctcctccgccccttcgcaacggagtcattgaagccctgactccaagaaccaggagttcgagcttcgttctgtaatcaagcatatatctttaccgcttcctcaggtgaatttgagaaggcaagcgctgcaacagcaccaatgagccccctttctccagttattacgagggtctcgattcccagttcctcagctattctcttagcctcttcaagctccacaagctgcgatttcaccttttcgccgtacttcctcagcttctcgggtatcaggatttttctgtaaaccaccatggccgtgttatcgctgagcgtcgtattttcgagctccttcctgaacgcattcacgagctcctcaggcctttctgtcgcaaaggttaccgagatcgaaacgcagttcgtcgtctttgcagggtttttcgtgtagagctgcgttatcgtgtgcagcaagtagtgcccgtatcctgccttttccagcttgaatgcgatttcgtttaccagggcccatgtagctcccttttccttgttgtccgtgtcgtcaacgcctatcactacctttgaatacttcttcagcctgatcttagcctttcccagctttgcaccaccgccgagttccagtatctcgattccgtctacatcctctgcaagtcccctgcaaactgtggccgcaattccaccgccggccagacctgcgtaggttatctcgattcgatccgtcagctttacctcctcgattcccgcgccagcaattcccggaatgagtttcagttcagttttaccgggctttaaaacgaagatgttcctcgccccctctcttctcgcacttatcaccaaatcgcttaccctcgggtagtgcagaacttcccaggaagcgcctccgtagcacctttcccttgcatggaactcgtgcagctccacgaattcaccgtccgtcactgcaacgactctcgaatacggcgaaacccacggcagacttccatatctcctgatgagctcatccttgctaaggtaactcatgcccgttcagctatcgctaagatttaaaaccttttggtttcgcttctctccaaaagctcgaaaggcttatcaggttgaagtaaaagagtcagtggatgaagcacgaagatttcagaaccagagtaaggaaaaaaaccgaagggctcagagaggtggagggagcctgtggaatttgccacggaacccttgaagcaattacagaagaaaaaggagttgtttctgcttacgaaagatcagaaggaatacttgcagtggtcaaagacgatagcggggacgtaataggcgagggtttcgacatagtatggtcctcagcgatactcgcggcagaattagatgccaaactcgttccggaaagatttgaggagaagctaagggaagccctgagcgaagaagatgagatcagggccattgcagacgtttacggttatggaagagtcgtgacaccctcggtcattgcgctgcaatacgtgaaggatctgggcgggaaaacggtgattaggagggaaaagattggagtcgttgcgaggctttacgacggaagcggaaacctgatcgcccagtctccagtctcctactgcccgacatgtgccatagtaaaggccatcgttaaaaacgatgaacttaaggatttcgtgaaggatagactgaaaaatgccagaaacaccggaaagatcaagtttgaagaaggggtagagaaccgctacatagccaagggaggggctgtaaaggcgagcataataaaaggagaaaaatggttggcgaagaatgttttggggtgctgcatagcgtattctacgacaaaggccgaaatcgcagcgggcttagttcctgaggagagcgcgaagagattcaaggcatactgcaacctctgcccgatgaagcactgctggatggagaagtccatgggagccatggggaacatagtgctgcaccgcctgagcgagatcggaatggagatagaggtcacttcggaggggttcatagttgcgaagatccccggagaaggttttgttggcagaggaactctctgctcactcagcgcgcttactaatatgctcctgacctcggacggaagcaagcttctgaagccatctcctgcgaagaggtttccaaacgccgaggagtagtggctgaaaagtttttatataggtatcgaaatctctgtgggaagtggatggagagaacagactcggagaaagctagccctgaacttattgaagggcttgagagagtattcagaaacaatgctggcttagccaaatcgttagtcagatacatcgggaagttttacgaaaagctggagaaagatttcggaagcttcaagatcatggacttctgcggaacacacgagtggactataactcattacgggctgagaagcctgatgcctaaaggtttagagctcgtcgcaggcccaggatgtcctgtttgcgtcaccccttcgtactacatcgagaatgcgctgaaactgagctttgatggagtggtcgtttatacctacggagacgttttcagacttcttagtctgaagcgcgtaaaaggggcaagctcactggcagaagccaaggccctcggggcagatgtgagaatcgtaacgggaatagcggatgcaatagcagatgctaaggcacacggaaaggaatcgattttcctcggcataggattcgaaactgtagcctcaggatacgcgcaggtgctgataagcggtcttttgcccgaaaacctgaagcttatgagcttagtcaagctcacacctccagcgatgctcgcgactgtggaaattcttgcgaaggagaagagtgctgtaaggggaataattgctccgggtcacgtttctacgatcattggagccaaagcctggaagcctgttgccgataagctcaaaattcccgtcgtagttacaggctttgagccgatagacgtgctcatagcgatcgcagaagtcttgaagcagctcgtcagaggtgaggcaaaggtttcgatagagtacgcgagagccgtgagctgggacggagacatcaaggcccaggagatgatcgatagggccttcagagtcgtaaaggatgcatggagggggattggatttctgccaaatagtggattgaggatctccgaatcttacagttctcatgacgctttcagggaatacggcattgagga

>Bin11_17

cgccactgcgcgaagctgagcgggatgggtgaggagaagatcttcgagttcgccgagaagctcaggatgaaggaacacctaagcagatctgtaaacttgggcttttctggcggagaggtgaagagatcagagatcctccagttgctcctgatgaatccagactttgttcttctcgacgaacccgacagcggtgtcgacctcgagaacatcgcaatagtaggctccgcaataagtgagcttctacagagagaaagggaggatcgggataagggcgggctgataataacgcatcaggggcacatcttagactacgtctctgcagactacggagtcatcctttacaagggaagggttgcatgcgttggagatccgaaggacataattgcgcagataaggaaaaacggctatgaggggtgtgttgcaagatgtctgaaggacatggagtagatatcgaaaagctgaaggaagttggcatcgaactagatccgaagaagcgctctggaacgtttctccagaaggatcaggaacccctgaaatacgcatcattctacgaaggcgtcgaagtgatgagcataaaggaagcgatgaagaaatacgaatgggtcagggactacttctggaaggtcttgaagagagatcaggatgaatacaccaaggaagcagacagcgaagaagcgaatggctacttcataagggcactgcctaacgcaaaggtagagatccctgttgaagcttgcctctaccttcacagagttggaaagcagaaggttcacaacataataatcgccgaggaaaattctgagataaacataatcaccggctgcacttctgacagggccgcagtgggaatgcacatcggaatttccgaattctttgttaagagaaatgcaaagctgaccttcacgatgatccacagctggcacaacgccatcgaagttaggccgagaagtgcagcgatcgtcgaagagggtgcgacattcattagcaactacatccttctgaacccagtaaagcttgtgcagatgtatcccaccgcttacgttggtagaaatgcacgggccgttttcagcagcgttatggtggcacttgaaggttcaagcatcgattccggctcgagagcagtgcttaaggaaagcggagccagtgcagagataatttcaagaacgataagcaagggaggaaaggtgatcgcccgtgggcaaataataggcgaggcagaaggggtcaaggggcacctcgagtgcaggggcctgatactgtcggagaagggagagataaacgccattcctgagctaatagcaaagcatccaaatgtcgagctaagccacgaagctgcaatcgggaagattgcagaagaggaagtcttctatttgatgtcgaggggtttgagcagagatgaggctgtatccgcaatcgttcggggcttcatggagatcgagatcaagggactcccagaagtgctgaaggccgcgatagatagggcgatagaggaaatgaaggacgctttatgaaggcctacatcgaagtggagtgcgacagggatttactcagcgttcttaagcccgaagagacgaaagcaattagcgaagcaaagatttatcaatctgaaggaaagctcaaactcgagatcgttgccgagtcaatttcagacctcagggccgcgatcaactcgtggttgaggctgataaacatgtgtttagagatagaggtgttggtatgagcgaacttcctccgcaggttcagaatctggttgcccaactgcagcagatacagcagcagcttcagcttgtagtcacccagaaggcacaggttgaggcgatgctcgaggaaacgaagcaggcaattgaagaactgcagaaaactggggacgaagcaacggtttacaaggcggtcgggagcatcctcgtaaaggaggagaagggcaaggttcttcaggagctgacagaaaggaaggagagctacgagatccgaataaagaccctcgaaaggcaggaggagaggctgagagagaggtttgcggaactgcagaaaaagcttcagggattcttaggcccctccggaggctgaattacttaatcagatactccagaattttttcaagtttctccctactaatttccctgcttattctcagaaattttccgctgaaggcgacaaaacccttatcgaaacggtatgcaattttgaatggatatatcctgcagaactttccaccgatttcgctcaggtctgcattgggtagatcgaagtcgagtttcagagtaaaggagaggtcatctcccacagccaccactccatcaaattccctcctcaaagcgctctgaagctcccatggcttccagtcccttgagtttgcaacctcgtagagctttaaaacgacctctttcgtagctctttcaccacctcgacgatcctcggcaaaatttctccagcctttcccttcagaatgtaatcggaaacacgctcacgggtgagaggggtttcttcgaagttcacttccacaacccttgcccctcttctcttcgcaaccctcggaagctcagctgccggataaaccactgctgaagtcccgcagacgagcattaaatcgcatttcatggcttcagaaaaactaagctcgtatacatcgcttggaattggctcggtgaagtaaacgacatccaccttaagcgggcttctgcattttctgcaaagaggtacctttttctctctgatctcctcctcaaattccgatctcggaaatctgcacccgcaatatatgcacctgagcttgtatatgctcccatgaaactcgagcacatttttgcttccagccttctggtgtaggccgtcgatgttctgggttataatggcctttaagatacccatctcctcaagctccgccagcgcgtaatgcgctaaatttggcttcgctctttcgatcatctcgtaaaagggagatttaagggcttcctcccagtattcccttggggtttctaaaaattttccgaaaatttcgtatgaccttctttccgcttcaggatccctcgtccagattccagatggacctctaaaatcaggaatcccactctcggtcgaaataccagcgcccgtgaaggcaaccgcgtgttttgcatttaaaagatccgctgcgcatcgccttatgagcccgggatccataaaatgagaatgagatcaagtttattattttagcggcaaaacaaatgcgatgctatgcgaaagatgtgcggaaagagtgaaaatcgatgataaaggcaaactcgggacctgcccgatctgctgtggtatctacgggaaactcgacgaaatagcgctaaaaattttagaaaatctggatttcgaatttgaaagcttccaggttgggataaaaagcgaggggagtttcaaagccctgcaggagttcttcgagctcagaggaatagactacgacttaaaaatagaaataaaaaacgaactgcagagtagattgaagagtctcacaggaaagaagctctcagtcgagcccgatgtgctgatccttctgaatccggaagacctgagcttcgagatctcgatcagacccatatacatttacgggagatacataaagaggataaggaacatatcgcagacgaggtggctctgcggaaaatgcgatggaagaggttgcgaggtctgcaattacacgggtaaaagatatctttattccgttgaagaactcatatcgattcccgcaatcgagctctttgaggctgaaaaagccgttcttcacggttctggaagggaggatgtcgatgcaaggatgctcggctctggaagaccattcgttttggaggtcgtcaaaccgaggaagagaaaagtcgatctagaggagcttcagaaaaggatcaacgaattttgcggtggaaaggttgctgtgagaggtttgagctacgcaaattctggcgacattgagagagtgaaggagggaagattcagaaaggtttacagggccaaggttgtatttgacagggaggtgagcagagaggaacttgagagagcactggcagaattgattggagagataaggcaaaagacccccgaaagggtcttgcacagaagggctgacaaactaaggatcaggaggctttacggggctgaaattttagcacactttggcagaattgctgttttgaggtttgaagctgatgccgggctttatataaaggaactcgttagcggagactccggaaggacggaaccaaatctttcaaagaatttcagagcccacgttgagaagctcgacgtaattcaggttcttgacagggcatagggagttcaacgacaaccaaaactttgataaatctaaggcaatacaggcagtgaagccccgtggggtagcggtcaatcctgcgggactctggattccgcgacgccggttcgaatccggccggggctacttttttaaggagaacgtttaataattcccctcagacttcaactgtgctcgacgatctcttctgcccaaagtgcaagagacttaagaggagatgcacttgcggaagcagcgtgagggagagaaatatagacctgctaaaaaggaactacaaagagttcctcctccctctattcgattgtgacgatgaaatcgtcttctacgaggttttcaatccatttaacgagtttccaaccgaaaaaatcgattttctctcccctaatttgcagaaggccctgaaaagcagaggaattgataggctttatccattccagaagagggcaatggaggtattgctcgagggaaaaaatgcagtaataactgctcctacgggcttcgggaaaactgaggcattcacgattccaatgcttgaaaaagctcgcaaaggaaaggccatcgtattttacccgacaaaggcacttgcaaaggaccaggagctgaagatcaagcaatatgccgaaactgttggccttaaggccgtgagattcgatggagattcgagttatgacgaaaaaagggcggcaatcagcggttctgcagacatactgcttacgaaccccgacatggtcgattaccacctcagaaatacccctgcattccgctcagtagcgagggaggcagtttatgttgcgattgatgagcttcacgtgtacacgggcttccttggctcgaacatgcactacctgatgaagagactttcacaatttgcagactttcagatcgcatgctcctcggcgactctggccaatgcaaaggagtttgcggaagagctctttgaaagggaattcgttcatgtgcacggagagcacagaaagacgccgctgaacctcgtcatgagggagtgtaagagcatctattccgcaattctcgatatcgtcagggcttatccgaataggaagatcctgatctttggaaacagctacagaaccgtcgagacgataaactggatgttaagaagggcaggaataagttctgcagtgcacaagggcggtttgacaaaggatgtgagggaaagtgttgaaagagatttcagggagggaaggttgagggttgttgttgcaacgcccacactcgaactgggcatagatgtcggagacgttgacatggtcctttcagagctcgttccctatgcccagttcttgcagagaataggaagagctggaagaagggggcaggagagcataggggttcttctgctccgcgtagacgatccgatctcgaccttctacaagagcaatccgagagaataccttctgaacgagccaaatggatacgttgagaagagaaacgaagaagtgatgcgctaccagactctatcgatgatccatgaaaagccaaccaagccagaagagcttgatcccgaaattttgagagctctaatttctgagggctacgtgattccgggaaaatttctgtcaataactgaaaagggcctgaatctaatcaggaacttttcgatgcgcggtatcggcgaaagagtgaagatgctctgcgagggcagggagataggggaaagggtattgcctgttgcgataagggagctttatcccaatgcgctgattattcacgatggaaggaagtacagggtaagagagatagaccttgaggaactttacgcagaacttgagccgttcgaaagtgaagaaataacggatccactttacacctccattccgaaaataataagggttgaggacagaatagaggagcccgtcgaggccttctacacaacgctcgaggtgaagatctcggtctacggctacgtaaagcgggatctcttaagagaaaggaaaagcattcattacttggacgaggaagttgtttacacctacagaaccaagggattcgtcttctcgtgcccatttcctgagttcagagagcatgaggacttctttgccgggagctttcatgctgtcgagcacgttttgattgaatctacaaacggacttactggaggaggtagcagggaaatgggtggaatttcagttcctgagggcgacatattcgtttacgatgccacgattggaggcaacggactcagcaagctcctattcaagagactcagaagggggtttgaaatagctctccagattctcgagagctgtgactgcagaagagttgacggctgtccaaagtgcacctacagctaccagtgtggaaacaacaacaaaccgcttaacaaagagggtgcgaaggaagttataaggctaattctcgggggcaagaatgtgaagaccaactggaaaaaatatgtggaaagtgcagatttccgctactttccctgagcttctaggactgccaatcctctaacagccttgaagaccctttcaagttcttcttccctgccgatcgccttcatgatgccgtagcagagcttcattattgcattcctgtgctcaaccttctgcctgaagatctgcacggggagaattttcatggaatcgtattcgtagaaatactcgttttcgatgccagcggattcaaaaaacttttttatctgaaacaaaagcagatggaggtgcacgatctcctcctttccgaactggccagacatgtcatggagcttgtagcttaatatataaaactgtcgttagttgagattttaaaaagctttttaagccgaacggggcattatgaacaattaattttttattttaggctgttcaagggaagaaatcaggaaattgccacttaagcggtcttttgtattaaaaagtagcaacttagatttttatcttgttaaaattaattatatcgacgaatgctatgaacagtagtaatattcaccactataaattttccaaatatttataaactgagcagtcgcctgctgcaacatgaaaacgatcgaagagataaacgagaagatcagacaaggagacgtctgcgtcgtcacggccgaagaagtgaaggaaatgatctccgagctcggcccagaaagagttctaaaggaagtcgacgttgtaacgaccggcacgtttggagctatgtgctcaacgggagctgtattcaatttcggtcattccgatcctcccataaaattcaaaaaagtctggctaaacgacgtggaagcttatgctggtcttgcagcagtcgatgcgtttctgggcgcgacgaagccatcggaaactaaggaactatatggcggatctaatgtcatagaggagctcgttgctgggaaagaggtcgagctgaaagccataggttacggaacggactgctatccgaggaaggagatagagacgtccgtttcccttgaagacataaaccaggctgaaatgataaacccgaggaacgcctaccagcgatacagagcagccacgaattcctctgataggcttctgaggacctacatgggaactttgttgccaaattacggcaacttaacctttgcaggcacgggtgagatatctccgctcaacaacgatccagaataccgcacaattggcgttggtacgaggatttttctctgcggggccaagggatacgtaataggagagggaacccagcattcaccgccctttggaacgctgatggtcaagggaaatcttaaggagatgagtccaaagttcataaaggcctgctacttcccgggctatgcaccttcgatttatgttggaataggtattccgattcccgtgctcgatgaggacattgtaaaggccttagctgttagagattccgatataaagacgacgatagtcgatttcggggtagcgagaagggtaaggccagttgtaagggaagtgagctatgcagaactgaagagcggtaaggtggagatcaacggcgaggaggtcagggtttccccgctttcgagcttctacatggctgaaaagatcatgagagagctgaagaagatgatagaaaggggagaattccttctttcgactccagttgagaggttgaagcgggaggaggtattcaggccgatgaagcaaaaggagatcaaggtcgtgaaggacgtgatggtaaaggcagtgacgattggagaggactggacagttgaagatgccgcgaggcttctgatagagaaaaacgtgaaccatttacccgtagtcgacgaagaaggctttttgaagggcatagtgacctcatgggacattgcaaaggctgttttcaggaaatcgaataaggtaagggacataatgacgagagacgtcatcacagtttaccccgaggatccgatagcgatagccgtgagcaagcttgagaaatacgacatctcagctttgcctgtgattgatagcaggagaaaggtcctcggaatcgtcaccagcgaagccctgagcaagcttttggggaggtgataaagatgctttttctactgagattcgattcgaagactgttcgcgagccgataatctctcttgccgcaataaaaacaggagcactgataaacattctgagagcagatgttggtgcaagaaagggcgagctgatagtcgaagttcctgaagaaaagtctgaggaagttttggaagtatttaaacggatgggagttgaggttcaggagatcacgaagtcaattgcgagaaacgaaaactgcgtccactgcggagcatgcatcagcatctgcccgacggaagtcttctattttaacggagagaaaaaaattgagctcagagctgagaaatgcatccactgcggagcctgtattaaagtctgcccgacgatggctctttattttccaatatagacctttcgaggagctccagatacgagaggcgaatgctctcatctcttgagtagccaagcattccagcgatcctgaatatctcctcctttttttcaagtcctccctcgacctcgatttcgatgaaatctccgagcccttcgacagagtcaacgcagattatcgccccttcaagcctatagatctttctgcgctttttaactgttctgaacttcttgaagcccagaaactcgaaaagccttaatgtctcattaaagctgctgacctcgacattcacttcttccctgctcttcgtctcggcatcaatctttgggcccttgtacgtgacctttatccttttctcctgccttatgcgcagagcctcatccgtttttgcgaaatccctgcagggatggcttaggtagacatcaatttcttccttgtcttcatacagtcttgctatcccctcgatcttctccaaaactccttccttcagcctgaactttgcctccacctccatagaattgcttcccatttggccttataaccctaccgtatataaacttcagaacaaaaagcggttgattagctcaaaaccaagaatgcaggaaacggtcagaattgaagcccataaaattgctcctacaatgatccatctcatcaaaaatgagacccaaccccatcgatccgtagctgtcccatactttctgaatgcttttccttggtctaaaaaagctgtacttttttataagcaattcaagcaaatttttgctcaaaagcagaacaacgaatccaccgaaaatggctccggttgccgatgaagtggctattagcaatggatggagctcgagagtaaaaacagcaggtatcgctacccatagttccaaggaagcgagaaaaaaatgattagcaggttgacagcagtttccatacttgatccctcaaccacaaattttgatgtgatggattaaaaatttttgcagaatgagattcgcagcagacaagagcggttgtactgataaaatatggtgccatggtttgcagcttttggttaataaagaccaaagagccctaataaacggctttaaatgcaattaaaatgcaaaatgatacactgctattttcgaaataggcatcaaagctgagctcgaatttttcaaagaacatctaagctcctgaggaggcaaacttgaatatttttctgagccgtccgagaaaaaccttataatcccacaaagataagatcccttaagctggggttgccgagtggtaaggcgacagcctgctaagctgttgggctttgcccgcgtgggttcgaatcccacccccagcgcttcgttaacggcttaacccaatccaaactcttcaatgaattctctaatcttctggtatttttccaagaatctgaaacccttgtcggtcagcgagtagtactttctgcccctcctgtcatagatctcacgaacgaagcccttggcaaccagttcgtttatgtactcgttaaaactctgcgtggaaaggtttgaaaatcttagcagccttgtaggtagaatcgaattgttgtgatcgagaatcgttttaagaatgtcgtgtattatttccagtcgatctctcttcctcatagctcatccaacttttttaagtattctgtaggctatgtaggccaatattaaactcgatgcgaggtaaaggcaaactcttacttctaaggcagttctaaacacaagtactaaaacgttcaaaatccagaaaacaaagagcaggagatacagagggtaaatccctccccagaaggactttctaacctccgaagccctgtaggcagagtatattgcaatcgcgagcaggaaaaacagtgcaatttgcgagatcacgagtaactcgaaagatctgaaaaaagtgatcacgagcgcgaccgttattgcgagtagatgcatcaaggcttcgctcagatcaaattcatcccacaccagactgaaggagagatacagtattgaaagagagccgaaataaacgagcagaaaaccactcagcaccgcaattgttctaaaatccactaaaagggcttgtggtggaatttcgaataacactccccaggagatgagtaagtaaagcaagcttctcgaaaaatatgcgagaccgagaaataaaaatgaatttctgaaatatctcacgctcttctcagccgtgagctcgtatatttccttggttttgagatgtatgagggaacaaagaatgaacacgaagatgaagtaggctgcctccgtggccaagagctcgagcataaagcggggcataccgaaagcttgcataacaactttataaataatatggaatgaagatccatccgtggaccttcaccgcctgcctatccttctgaccaggacgtagagaccaatgagtgccgatgcgatgatcgccatgccaaggaatgttgcgtatcccttctcttgcaccacgaccgtgattctgtctgtagttgaagtctgatcggcgtggagacttatgctgaccctgtactcgctcggtgaggcatctgaagggatcgggattttgattctgacctgctcgctctctccagccctgagcgagggaattagctctggattcacttccagaacttcccaatcgcccggagtgcttgcagaaaccttgatgttcgtcaaaattgttccgatcccagaattctttacattgcccctaatcaaaacggtgtctccctgggaggcagtaacggtgtattttccaccctcgggttcgaaagtcagctgaaatgtgccaatgattgaaagatttagacttgcctcggctgtagacattccctcagcaatgaccctcaggcctatttcagatggttctgcggtagatgggacgaatatatccaaagtgagctccttagcgctgccgctctctataaatactcccgaaactgccaagtttccctccttaaaggttgctgtgtagccgtatggcagaccctcaacgcttaaagcatagctatcgtctgcgtaccccaagttcttgatcttcagaggaatgctcgtaacggtaccgattgttgccgaaactctggagctcattgttatttcagcgtaataagctttcttttccagaagaactattccgagattgttagttctcccacccttaaccttcacatctcttattgtcttttcatggtaaccgcctttctcgatcctcaggtcgtaggttcccggcattatttctacgattgctgtcccatctcccgaagtgtaaaaggtttcgttaccaaccgttatcctagcagaagaaacacctttcccgtctttgtcaataaccgtgagtctgatctcccccttctcgttaacatggcttttgttgacaagtatctcaatattttcagctttttcgttgaacttcagcactacgctgtaattccccacatcagaagagctgcttgtctctatcgctgccctgatcgttctgctctcgccagctgagagtgataaccggaatacttcaacgccagaatcgtaaaagtggcactcccagccttcaggagcgttgcagctcagtgagacctcgtaaatcccgcttagcttgttcctgagcgttagatctatgattgcagtgtcccctgcctccaaaattagacctcttattttgggcaaaacttcaagggcataatgtgagtaaccgacttttacctttaccgagacgctgctgtccgcggaaagtgtgagctggtagtctcccgtttccttgggagctgtgaagacgaagctgaagcctacggattcgttggatttcaattcaagcgaggaaacctttttgccatcatagcagaagtatccttctatcttcgccggataagagctgtaagagaggtagtaggtttcttccttttctcctgagtttttcagtaaaagcggaatgctgacttcttcacccggaacggcggttatctccgatatcttggtctccaatgctgaaactggaaccagtagagccagaatcaggaatattgcagctttcacagctcctccctcgtgaagcggaaataggcgattccgaagagcactgctgggatgatcaaaaggctcagaatattcttcgttatgtcaacggttcggaagaccgttgattgcgttaggctgcctacgattgtagtatagcttgaaacaggcgaaaatactgagatggtcgaggttatgctctgcactttctccctgtaacttctcatttcctccatgtagctaacccatctcggatcgcttgcatttctgaagttttgatttctcatcagatcgaatggctcttccggtggctggccagccagagattgagcgatgatactgctgaacatcgggatcataagggcaagtactatgaagatgccgatggtaatcgcgagagaagttgaggagttttttaccattgccgacacgagaaaggcaatcgagaaaaacgtgaatagataggcgttcgttgcgagagtaagctttgcaaggctgagaatttcttcgaagctgggcgtgtatatcatcgtaattgcccccaccatgaaaagcatagtaacaaaagaagccaaacatatggccaagaaggcccctatcgcctttccgattatgatttcatcccggaaaagcggatgggtaagcagaattcgcagagttccgctttctttctctcttgtgatcatgtcaaatccaagcgctataccgaaaattccgccaacaaatgcgatccctgagctgccgaagaagccgatagccgttcccatcgagatcctgctggcggagagcgtctgtagaacgctcatcaggaagaagactaccatcgcaaacagcaaagccatgaagcgcttgcttgttattatgtccacaaattcctttttagccactattaacgccctcattttccctcaccaggcggaagtaaaattcctcgagattcggtactttcagcctgagttccttgacgacgtaaccattctcaaacaagtgtttcgacagctcaaccctgcagtccttttcgacttcaacgatccatgcgtctctcttttcatcatagctaacccttccgaatcttttgagcagaccttcatcagctctgggctcggtctggactaccaccgcaagtgaaccgcaaagctcgctgatcttcccaaccctgagcattcgccccctgtaaatgattcccactgtgtcgcagacctctttgacttcagagaggatgtgcgaggagaaaaagatcgtctttcccttcttctgcaaatccttcatgattttttgaaagtcagcgattccctggggatccagcccataagtcggttcgtcgagaaaaacaaccttgggatcgttgagaatggcttgcgcaagaccaagtctctgcttcattcctctgctgaactcggacacctttttctcagccgcgtggctcaatccgacgagagccaaaagctcttcaatggcatcccttgaaatcccgccatagaattccgagaaatatttcaggttgtctatagcggtcatatttccatagaagccgtaattatcgggtaaatacccgcagaccctcctgacttctaagggattttcttcgacatcgattccgcagacccttatttttccgctgtcgggctttattaggccgagaatgcagtttatcgtcgtcgattttcccgacccattcgggccgaggaatccaaaaatgtctttttcttccacgtaaaggttcaagttgtctaaagccctgaactgcccgaaagatttcgttaaaccctctatttcaatttctgccatactcttatatctattgtgagtataaaaacagagtgaagaatcgatgtgcatttgaagtccattcccagaacttcccttatcagaaaggttatggactctgtgttgtattcagcgattatgataaaattaaataaaatc

>Bin11_18

agattttgacaaagtatgcaagatcatgagaagatacaaactcctgccgaacgatgccttgatagccgcaacttgcagacactacggcataagaaagattgcaaccttcgacgaagattttaagcgcgtggattatctcgaagttgtcgagatttgattgccattggctaaaatctatttgacttcgccaaatcgaccgctttaaatctaaacctggctatttttgcaccatttcaaaactactgttgcatgaaggcttggaggaatgcaaccgcgagctaaccttaaaactcatgcgaaaaccttttactcaagcttcctgactcttctgacaagatcctccacgatttcctgtctcgactttagcgcagttcattagttctatcaagctttgaggaaatctcccgaactgctgtaactgtctcgtcatttttcttgagcacagaatccattttcgataaacctctccaagcagagaaatgctcacatcatgttttttcaaaatctcatcctgctttttcagcattgagtccttttttcaaccatgagcaatccgacctgaactatctttgaaagttgggctgaatcgaagctggtcctgaatatatctatccctctgactctcccgtcatattcctcgacttttatttcctcaacgacggcttttggaggtacgtcctcctttacgaacttcaggaagttttcgagctgctctttgttgccatcgacaagaactattaaagcttctttcccgcctatttttacgtttcttgcctcgaagttaggtattagcaggttttcagcttcttcgagcaggaaagctctgtatccgacattgtgaaaaccccttggatcgcgatcttgagtgaaattacttcttttactccttttcagcattaaaaagattttctgggcatcaggcaatgtccctagaggtgtcgatctgaactccttcatctactaagaaccggaagtattcgtttatcggccttcttccccttatctttggcagtgcgaatttcgagacgatcctttcgccaatcctctcagattcaacgttgatcacgacatcagaaagatcgagtaccttataaaccacgcttatcggatgcaggttttttgtcagataaacgtaaacaggcgattcagtttccttcgatctcgcgtagagcttgttcagaagccagtccttcaggccccactccacttccaggttgaggaagaaagagattgagtcgacgattatgctcgctttctcatttcctatctggttcagcatgtctgcgacgaagtcgaagatctccttgtccctgtacctgtcctcaacgacgaactggccgaattcgttcaggtagtactgggtgaaaacgtcaacaaaattcacgttcttcgtttcaagccttagcgactgcatgtcccttaaaatgaactttgcaggtctgtttgtgttaaagtaatagctctttgagacgcatgcaaactggtagagaaaagcttcaggcattgcaatcggatcggcgtaaacacagacgaagctgccagctggcagcccgccatcaagccttttatcgagtaaggcgatgcccgttggcactgcatttcgcagcagcagcttctgctccatcattgttaagaattaaacctttggatatttaacgctatcgacttccaaagagctcaaaaacccttttaagcattcctgcgatgcggaatgcatgaaaatagcggtcattggtgcaggacttactggcttaagagctgcaatgcagttgaaggagtttgcaaatgtcgtgatattcgaaaaaaacgaggttggcggacttctctcaagcttatgcaatggatacttcattgaaaagttctatcaccacctcttcagaaccgatttggagttgctcgatgtcataaaggaattaaagctggcttctaaacttgtctggaggactgtgaaggttggattcgctcatgaaggcaaaatatactctctcagttcccccatcgagattttgacctatccgcatctctcgcttctcgataagttaaggctcgctatcttcacactgcagagcagaaggctaaaatacgaggaattcgacgaagtagcagcaattgctgccttaagggaaagattcagcgagagactgctcgagaatttcttcatgcccatgctgaaggcgaagttcggagagaatgctgaaaaggtcagctatgcatggctccttgcgagggtcgcgataaggagcaacagaaaactcaggggagaggagatcggatatctaagacatggcttccatcagctcatcgaaaaattgtccgaaggactcgaaatcgttcattcgccgtgcatcatcaagcggggcagcaggtggaacgtaaatggaacagatttcgatgcagtagttttcacagcaccaattcctgagctcggagatttagcggcgaaattcggtctgaaggaaataaaataccagagctccgtttgcgccttgctttccctgaaggaaagcatttcggacgtttactggaccaacgtcagggacgcaagctttggtgcgataattgagcacacgaacttcatgcctttcgaagactacggagagaaccttgtttacctggcaagctactcaacaccggaaggctggctcttcaaaatgggagaagatgagatagggaagcttttcctttcagaccttgaaagattcggaatagaggacaaagaagtaaactggatccgcatcttcaaggccaaatacagctctccgatatacgagaagggttttttgaagaaaataacgccttacagagtttccaacggattctacatagcagggatgacatcaaagccgaattatcctgagaggagcatcaacggaagcttgagagctggaagggaggttgcggagcagataaagagggactttggatttgaaaattcataaaaaccgaacatgaagttggattatgaagctcggattcgtcttcgagagtgccgaatgcgttgctgagcttcgggaagaactcgctcctgaaacagttaaggccatactccgatctctgccgattgaggcggttgcgaaccgatggggtgacgagatctactttgaaacaaatgtagactgcagagtagcggagaacagcagggaaatcgttgaaatcggcgacgtcgcttactggattccagggaaggccatatgcctcttcttcggtaaaaccccgataagcgatgataaaatcaggccagcaagcgcggttaacgttgtgggaaagataatcggggaccctcgagtgctgaagaaggttaaggacggagaaagagtttttgtcagcctaataaaataaaaaaatttaattttatctgcccttgaatttgggcttctcctttctgaagaaggctgaaaatccttccttcacgtcttctgtcgagaagacaactccgcacgccgtgcactcccattccatgccaacgtccattggcacctcgccgcccttgtttatcagcctctttatgagcgccatcgaaatcggggcgcatctctcgacgatgttctttgcatacttcagcacctcttcctcgaacttttcatcgtcgaatgccttgtttacgattccccatctctcagcctcctcgcccgtgatcctctctcccgtcagcgcgagctgcatcgccctgcttattcccacgagctttgcaagcctctgcgtgccactccatcccggaataattccaaggccgacttctggcaaaccaatgactgcggacttctttgcaagccttatgtcgcagttcatcgctatctcgaatccaccaccgagggcgtatccgttaattgcagcgataacgggcttcggtatctcgcccagtcttctgaaaatcctctctccctttctgttgtgctccacgaagtcgaatggatgtgttatcatcaccgtcagatctgctccagccgagaatgccctgcctgcgccggttattataatgactctcgtttccttgtcgttccagagcatcgttatggctttgtcgagctcgtcgagcatttcaaccgaaattgtgttcagcctgtcaggtctgttgagaatgagcttcgttatcccgtctccgagcttctcgatctttatcgtctggaactcctcactcggcttctcctccttcttttcgagcagcttcttcagcttcttctgctttatcgtctctgcaggctcgaagatcttctttccgaacatctgggcaagttcgttcagcctgttcacgatctggtcctcttcgagcgtggccgcgatttcgaatggcccgaatggtctgttgaggcccagcttcacggccgtgtcgatgtcttggggatctgcaactcccatctcaacaagctttacggcttcgtttatctcgacgagggagaagtcaagcgggctgatcttgtctgtagccttttccatatctatctgcggtcttccagagctccagtcgtaccatccctttccagtcttctttccaagccttcccgattttaccatctcttcaactatcttcggaggctcgtagtcggggctgatcatctttgcgtagtacttgctcgcatggtagaatacatcaactcctgtaaagtccattagctcaaacggacccataggaaggcccattttcctcatcatcgcatctatctcctctggggtcgcgatgcccttgtcgattatggccatgagaagggctccggatggagcctgaatcctgttgacgatgaatcccggcacatccttgcggacgattacgggaacctttccaatctttttaacgtaatcaacgcagaccttcacgacctcatcactcgtcttttctcccttgatgatctcaacgagcttcatcacaaccgctggattgaagaagtggagtccaacgaacctatcttccctcttagttgccttcgctagatcggttattctcatcgttgaggtgttgcttgcaaatatgcactctggttttgcgagcttgtcgcattcggcgaaaacctgtgtctttatctcgaaaacttcaggaacggcctctatgatgagatctgcatcctttacggcttcagcgagatcaaccgttggatgaattctctctagaactttttcagcgctctcaattttccccttttcctgaagctttgccaagctttgctttatcctttccatccctctgtcaacgaattcctgctttatgtctctcatccatacttcgtagccagccattgcagaaacttcggcaatcccatgacccatgtctcccgcaccgagaacagctatctttttgatctccatactaccaccgattactactcgataatcaattaaaaaatctttctgtttttgatcgcctcactgcagctttgccagatagttcctgacgttttcttctacctttgaataatcgtagctacaaaccgcaacgtttccaacgggtgtcccctttagcttcagcggattgaatggctcccgtccgctgaaaaagatgaaggtatcgcacttgtactcaactccgtttattctgactccctcaagcttcccctctccaataacctcgatgtcttccgttgcctccaagaactcaaaatcgtagtccatctcttcgagctgtttctctgccagatccagaatcgggtcccgcttgccggcaattattatcttttctcccagccttttgttctcggaaagaattctgagcgactggtcgagcgtgtaaattcccgacatcttctttctcaaaacattcaattttactggcagcgagtctgcggcaccactgcagagaatcgctcttcgtgcatcaaaccgctttagtccattctttgagttaacgataacgggttttgactttatcaccgtgcccaaaccgacctctatgtccgccttcaattcattgacttctttgatatacttctcgtatttctcggcaaagactccgagcttggaaaaaacttcgagctctccacctgcgaatctcatgtcgaatatgaggatctcgaagcccttttcgcgaagcctgctcgcacagtgaagtcccgcatatcctgcacctatgacgacgtaatcgaccgataccattctgatcgatctgagatagataatctctgtataaaattattttgtccgatgacgaacagccaaaggcttatttgttttcggtcgatttgcgggcatgaagttcgtcacctccaacgagggaaagttcagagaggcgagggagatcgcgaagaaatacggaatcgagttagagtggctgaaaatgaagtatgaggaaccccagggggaagatctggaagaaatagccagaaggagcgcggagatcctttcagggcaaataaaggagccatttttcatcgaggacagcggactgttcatcgaagctctcaacggatttcccggtccgtattcgagctacgttttcaaaaccataggcaacgggggaataatcaagctaatggaaggagtttcaaacagaagagcgtacttc

>Bin11_19

gaaagatatatatatcgcacgacctcaaagctgaaaggtgattgaatggattctgctgaaaatgcaagagctttgctcaaagagaatggcattaaacaggttctttgtgcatttagcgatcttcgaggctatttgatgactttttcaattcctgccagagaattcattgagggcaatgccttcaagggcattggcttcgacggctcctcaataaggggctttaagagcatagagcaaagcgatatgatctggattccagatccgaaaacgattaaagtcattccttggatcagcgatccgctccagaagtcggccataatgttcggcgatatttacgaagcttgggcttcaggaattgctgaatgcgatccccgcggctacgttgcaaagagaattgaaaatgagctggagagccagggaatgagtgcagtatttggcccggagatagagttctttgtttttcagaatgtggatttcaaaaatctcacctgggacctatggacatctccaaacggcggtgcgggagacagctggggagctccaagggttctgccgcagagtccggaactgagcggagcctatgtggtgcgccctaaggagggatactttagagccccaccggaagacaccaccttagaattccgaaacgagcttgtccacaatctggagcagttcggcgttcttgtagaatttcatcaccatgaggttgcaaccccggggcaggttgaactcgatttcaagcccaagaggctaactcctgtctgcgatgccttttacctttacaagcttgcagcaaagaacattgcaaggaagtatggactaatcgcgacattcatgccgaagccgatatttttggacaatgcaagcggaatgcacacacatcaaagcctttggaaaggcgaaccattcaagggtgaggcactttttgcggatccgaatgatgagtacatgctcagccaggaggcaagatactacattggtggtctgctcgagcacgcaaaagccctaactgcaatttgctgcccaacgattaacagctacaaacgtcttgttccgggctttgaggcaccaataaacatatgctggagcccgagaaacagatcggccttggtgagagttccaatgtatgtaaaaaagccttcagcgataagagttgaatatcgcggagcagatccgagctgcaatccctatttggcaatttcagcgcagcttgcagcgggccttgatggtataaagaagaaaatagacccaggcgaccccgtgctggtcgatgtatacgagctatcgagcagggagaagagagaaatgggtattggagagctccctacaacgctgagggatgctctcgatcacttggcgaccgatgaagttctccagagagtcttgggcagtcatatattcgatgcgttcatggagctaaaaatcgacgagtggaatcagtactgcctctacatcacgccctgggagttcatgaagtattttgacatctgattatataaatttcgaaatttattctttaccaccaatcagccaaacctttttagggatcgaaagcgcaagattaacggtgattatatggaaagaagaagaaagagatggataaccgactggtggccagaaaggctcaatttgaaaattctaagacagaactgtccaaagagcaacccttatggtgagaaattcgattacgccaaagagttcgagagcctcgatatggaagaggttaagaaggacctaaaagagctgatgacgaaatctcaggactggtggcctgcagattttggccattatggccctcttttcattcgcttagcttggcacagtgcaggaagctacagaatttacgatggccgcggtggggcaagaaccggagatataaggtttcctcctcggatcaactggcctgacaacataaacctcgataaggcaattcgacttctctggccaataaagcagaagtatgggagaaagatctcctgggccgatctcatagtcttggctggaaatgttgctctggagtcgatgggcgttaaaaccataggatttgccgggggaagagaggatgtatgggaaccagatgagagtcctgattgggggcctgagatcgaaatgctaacggataaacgcttcgaaggggagaaactgagagaaccttttgcagcaactgaaatgggtctgatttacgtcaatcccgaggggcctagaggaaatcctgatcctgctgagtcggcaaagcagataaggctcgcattcagcaggatgggaatgaacgatgaggagaccgtcgctttaatagctggaggtcatgcatttggcaaatgccatggagcggctccaagcaatttcttaggacctgacccctcatcttctccgatcgaacagcagggtctgggctggaagtttaactacgggaagggaaaggggcctgatacttttacttctggttttgagctcacttggtctccaactccgacaaagttcgggatccagtaccttcatctcctttttaagtacgattgggaactgacaaagagtccagctggaaaataccagtgggtagcgaaaaatgctcctgcaataatcccagatgcccacgatccaagcaagaagcatccgccaatgatgctcacttccgatcttgccctcaaattcgatccgatctattcaaaaattgcaaaaaggtttttggagaatccaaaggaattcgaagaagcatttgcaaaggcatggttcaagctgatacaccgcgaccttgggccgagagagtgctatcttggcaaagaagtgccgaaggaggtttttatatggcaggatccgttgcctgcgagggattacgagctgatagatgaaaatgatgccgagaggcttaaagaagagatcctcggatcaggactcggaatacgggagctcgtttacaccaccttatccgcagctctgacctacagagactcagacaggaaagggggagtaaatggcgcgagaataaggcttcttccgcagtgcaggtgggaagtgaatcacccagaagatcttgcaaaagtcattgctgtatacgagaagataaagaaaagattcgatgcagaaaatggaggcaagagggtatccattgctgacttgatagttctcggaggaaatgcagcaattgaaaaagccgcgaaaatggctggatttgaaataaagattccattcatccctggaagggttgacgcgccacaagaattaatcgatgtagacttctggtcggctgtagagccaaatgcttgtggcttcaggaattacatcaaggatggaagcataaacctaaaggtgagagtggaagagctcctggtcgacaaagcacagctcctcaggctttcagttccggaactcgtggctttgtttggaggaattagggctcttggagcggtttataggtatagcagcctcggagtgctgacggatagacctgaaatgctgacaaatgacttcttcgtcaacttgctcgacatgtccactgaatggaagcccgttgatgacttaggatacctttacgagagttatgataggaagagcggagagctgaaatggagggctacgagattcgatcttatctttgggcaccatgaggagctgagggcgatctgtgaggtctacgctgctttggatggaaaggagaaattcgtcagggacttcgtttcagtttggacaaagctcatgcacatcgatcgctacgacatctggaggtccaacagagaaatctacaaaaaaattacagcaggaattttttaaaattcctgctggcagaaccgcatttcctgaaaagagctcagccgtaatcttagagctgaaaattactctatgcagacttttcttaatttaaaaaaatttagtgggtggagcaagacaggcacgggtcgtaagccctgaacaacatttccgctagtctcttaagttgctctttcggcttgttgatgttcgctgagagcagggcgtataggtcttcttcgatgtttgttgtattcattccggtcgggatgatcaagttcgcatccttcacgattccgtttttatccacagtgtattcatggatcaaggttcctctgggagcctcaactactcccactcccgttccggctttcgtctgcttcggtctttcctcgtccttcagcttcaccgcaagctcttcggcaatctttatcgcctcttcggcgtagtgaaccatctcgagagcctgggcaaggttgttgtggaacgggttatcgctcgggaactttacagcacattccttcagcaactctttcgctgtgggatttaacctgtccttgaagagattaaccctcgcaattgctccaacatggaatccatgcccattcatgacgcagaacttcgtgttcgaatatggcttgaaaacctctttgatgtggttcctgtactcctttgcctccacattgaggcctctgtttgtcacaagcctccctgcgtaggcagggaagtgctttccgtcatctattgccagatactctgtcggtctctcaaaatccgggcactttaggctagcaaaaagcctgaaggtttcgatgcagtcattcagtatcgctttgaagtcctttgcaatcttttccatgtcctcccttgtcgggagcttcgcaaaacctcctggaattgagttgatcgggtgaacggcccttcctccaactaattcgatgcccctgtttccaacagttcttatcctgacgcacctcttcacggcatcggggaacttcgcgtaaacctcggatattccggttacgcccagataatctgctgctgcgagcacatagagatgcagggcatgatcctgaaccatctgtgaggatatcatgaaccttctcagcatttttgtttgttccgaagcttcaatgcccattgcgttctccaaggcctgaattgcggccatgttgtgcggcacagggcaaaccccgcaaattctcgaggacatctggggaacctcgtcagctcttctgcctctcaaaaagctctcaaagagcctcgagccttcatggatttcaaggcgcacctcaacgaccttaccgtctttgatgtcaaaccagattcttccgtggccctcgatctgggttacgaactcgtcatagatcatagcttttcacctccaacatccttaaacagcggagccatgggtgcgtactttctgaagagcctgactatgtcgtccttggatgctatcttctttaaaagttcgagttcagcactcatctgcggttcctcaattgggcccctgcaaccgtcgcacggcattcctgcagacggacaaggagcgccgcagcctccatagctcacgggaccggcgcatgggacatttttgagcagctggcatggattccccctgctcttgcagtccatgcacactggtcttgtgtgctctctcggaattctgcctgcaaagagatcgctcacaactctttcaaagtcttcaggcacgatcgggcatccgcgaatcatgaaatccaccttaatgatctcaggcagcggttttatgtacagcgtttcgatcttgtcggcattctcgccgtagacgtatctcttcgcctcgtttatgtccatgaagttgcttatcgcggcaattcctccgaagcaggcgcagctacccaaagcgacgacgatcttgctgtgctccctgatcttcttcacaagctcaagctcgtggctcgtgataacagcaccttcaatgaaggcaacatcgaactcctcccacctgtttaccctctgcccaaggcggaagtggacgaattctacaccaccgagaaccctgtctatgaacttcgagtttacaatctgaacttcacaaccttcgcaatctgttaaatcgaaaattgcaaccttcggcttcattcagatcacctcatatcgattcaggcaactgcttcacttctgcatagttgaacactgggccatcgagacagcagtatctgttgtttatctggcagtgctggcacttgccaactccgcacttcatgtgcctttcgagagaaacgtaaatcgacgattcaggcattccggccttcttgaactcctgaatgacgaacttgtacatcaccggaggaccgcagactattccgactgcatcctttatcgggtaatttagtttcgcaaacagggttgtcacaactccgatgttacccttccaaccacttggctttccatcgggaagcttgtcggagtccacagttactaagaagttgcactccttagcccacctgtcgaactcttctttgaagagcagatcagctggagttctggcaccgtaaaggaccgtgatctttccgaattccttcttgttatgcagcacgtagttcagggagctcctgagaggagccattcctattccgccagcaacatatacgatgtctctgccctttatcgtctcaaaggggaatcccacgccgtatgggccacggatccagacgtagtcacctgctttcatcctgtgaagagcgttggtgacgtttccgacccttctgacgcagaggtctatgtatccctcctcgtgtgggcttgaagttatcgaaattggagcttcaccgatgccggggatcgagagctctacgaattgacccggcatgtacttgaagtctttaacctcgagcctgaaaaccttggtgtcgtaggtttgctctgtaaccttaaggatcttgctcttaaagggcttgtatatattctccatccttatgcacctcccaacttgctgagattttccctcaggtctatatcagccgggcagaaggttacgcaccttccgcatcccacgcagctcggaacgccaaattcgtcaacgaaggtcttgaacttgtgagtgtacacattcctgtatcttcccttgaggtcccttctgaagttgtgcccacccgcgacccttgcaaagctaaggagaaggcaggagtcccagcatctaatcctctgcccctcgttgggaaccacgaaggtttcgtcgataacatcgtagcagtagcaaagcgggcaaacgagagtgcagttggtacatgcaaggcacttcttcgcagtttcctcccagatcggattgttgtagtcttccaagatccttctcccgatcttgtaaatgtcgagcggtatcttttcctcgtattctatcttcacttccctctcttctacggtctcggggccgaagtagagctgcgatagcaatttccttcccttttcagtcatcgaaaatcccacatagctgtctccttcagccttcagatacagatcgcaggcgttcgtcagaacgttcgctcctgtggacttgcagaaacagctctccgatggcttgcaggatatgccgacgatgagcattttttctcgccttgccttatagtaggggtcgttccagaacaccagatcgagccttccgatcgcatttacgtcgcagaggtgaattccaaagagggctattttctcatcgggaatcgtctccttgaactctccgccgtcaaatgtgaacatggtgtccttgtagggaagcaagaattttttcggtggtaaaactgtggttggatagtccagcgcgacttcgctgaagtctttgacttccattagaacgtagtcgtcctttctcttcactggagcgatcactttgtattctttgcttatgtcatcgaacagcttttttaaattctccttcctaatcctttttcccatcaaagctatcaccctctaaagctctgctttagcgaaaagcatggtaatataaatagattgcgaaagatgcgaccccaaaaggctttctgaattgttactccaagcagaatttttgctactcatgcataattaattgtttagtctgttttcagcgacgagccctttaaatactatccgggatttcaaaacatatgcatgagtgggccattgcgatggcagtcgtgagcagcgttgagaggtggtccatggaaaagaacttggatgtaaggagggtctttctctcaatcccctctctctcaatgctcgaagtcgaattgctccttgaggctttcaacacgatcaaaaaggagtcaaaggtctgcaatgcagaactcgaagtccgtgtgagagaccagaacctcaaatgcagaagctgtggaaacctttttaccctgaatgacataaggggccagcttgatccagtattgagggactatggcgaggagaacccgcttcacattatcccatcgctcataacagcgtttgcaaaatgcccgaaatgtggttcccacgacctcgaggtcgattcctcgatcagggtcgaggggatagaggtatgaatccgctcctcgagcttgcaagggagcgttttaaggataagaaggttcttgctgtgatgagcgcgaaggggggagttggaaagagcaccatcgcatccttaatagccctcaatttaagtgaaaggcaaaaaacagctctaattgacctcgatatctacggaaattccaccttaaagctttttggaattaattgtctccatgaggtgagcaaggaaggaattgagccattcatagttggaaacctgtccattttcagcgtcggtgggcttgtaggagatcgctacatagttttacctggaaaggaggaaggtaatgtacttgaatcccttctcggacttgcaaatgtcaaagaggacactgtagtaatcgacacaccacctggaatgggagaagaactccttacgctgagaagggttgccaatccagaagtcatcctcgtaacactgccatcgaagctatcagtgatggtggtaaagcagatgctcgactacttgctcgagtccaaaattgaggtaaaaaggctcgtggcaaacatgtgctacctgaacagcccaaatggccagatttatccctttggaaagcctgaaatcgttgagaagattggaaaagagtacggaataactgtggactacctgcccgttgatccagagctcgaaaagttctccgacaggattcagaactattctggagtccttagagaggatgtcagaaaaatccttgaaagttaatgtgctaagaaaagtttatataatttcaattttaaaaccgttgcgatgaccgacttgaacgagggaatggagaatctgcggaagggctctctggtgtatttgcttgctgttgctgtttcctgggcttcgattatacccttaatggctacaggcttcacggcgggcgatttaatgaacccggcattaattgccagtgtcggggcccttgcaattttaatcgtggcagcgatcgtgctcggcattgcgagcttcgtttacctgttcatggccacaggagcactaaggaaggtgaatgagaggtacggaataggcagaaaagggttgctcctgatgctcgtgggactgattttgttctctttctccttaatactcgcactttttgctgctgcaacaggagcatctgatgacattgcattggtgatcgttggaatcatcgggttgctctggataattgccgcagttttattgctgatcggcgtaattttattcggaataatgctgctgagactcgaagatgtggacacacgtttcaaaacggcaggaataatttatcttatcggaattatagtaccatttctaattatagtcgccatgattcttgtctactccgcagcgaacagagcgattaaggcggcaagggctgcagtttgaaaaatgataagtattaaatacgttatcccattattttttctatggcgaaggtgaaggtaggagttctgaagatgggtgccattggcacggcagtcattctcgaatacctgcttgacgagagggccgatagggaggacatcgaagtccgtgttgtgacaagcggtgcaaaaatgcagcctgaggaagctgtgattgcagaaaaactgaaggaattcgatcccgatctgataatagtcgcttctcctaacgctgcgctgcaggggccgaaggtggcgagagaagcttttgcaggaaagcccgttatagtgatcagcgatgctcctgcgaagaaggcaaaggatgagctgaaggagaagggtttcggctacatcttcctaaatgctgactcgatgatcggtgcccgcagggagtttctcgatcccacggaaatggccctattcaacgctgacgtgctcaaagttctcgccgccactggagccctcagggttgttcaagaagcgatcgatagggttatagaggatataaaggccggaaagaagcctgaaatgccccagatcatagtcacggctgagaaagctgtagaagccggaaggttcagcaatccgtacgcgaaggcaaaggctatggctgcatatttcatcgcagaaaaggttgcggacatcaacgtccgtggatgcttcgttgagaaggattacaacgtctacgtgcccttagttgcctctgcacacgaaatgatgcgcattgcagcaattcttgcagatgaagcgagagagatagagaaatacggggacaagttgttcagagatccccattcgagagaaggaaaaatcctctcaaagactgaactgataagcaagccacagtaatttttaatttttattacccttcaatgaaaggatttattttgacgtttttccgacttagcgggcagatgaagcatgcgcaacagcccagcgggcttcaagaaaattattcagtcccagaattgctccccgaaaagtttattaatttaaagaataactcagttaggggcgtcgatacaacattggcatctatcccgccgccgatgcagctgtgttatttttggcgccccctacgttttctctatcaaaactgcgtttataactccatcttggcttgggcggtttgtaactcttgcttttccgatttcggtctgaatcactgcgcttttcgtaattaccccatgtctcacgtagtgcatatgcgcagggttctcgagaacgctcttgatttccacccttaaaacctttttctgcgttggatcgtaaacgtttgcgtatttatccgagaagagcttgagcttataacttccccccatcatcctctccttctttgcctttcttccgccaattgttgtcagcacctgctctccgccgagctcgtacttcctctttttcctgtgcggcctgtatagccctcctgttggtttccttctactcctaccctggaagatcatccgctcacctttcccttgctatcactctcgccttaaagattttatgccttctggataagagctaatggggagcggtggtggacggcttaccgagaggaggaaagtccccccacccgaaaagatgggctccgcaaggagcatccccgagaggggatcgggcgggaacagaaaacccccggcagggggaaatgcgatgaggccgaagggcagagatcactcctgctggttgaaattccggcccgccatcgggtgcaaggctcaatgccgctcagactgatgccgtcggaacagaagggggcttactaccaccactcccctcttcacccgaacctcactcaacaaatttttaaggcccccgattaaagggggagacatggatccagttgaagatttcgtgaaactcgttggaagtgcgccatggtacaggctgatcggaatggaggtaaagaaggggcaaagcgagatcacggttgagatagaggtggaggaaaaacaccttcaggcccttggaatggtccatggcggagcaatagcgagcatactcgactccgcaattggccttaacataaacaaagagctggtcgggaagggaaagctggcaataacttcgcagctcaacgttcactatctgaaacctgtatatgggggcagaatcatcgcaaaggcaaagccattgttcataggctcaaaggtcgcagttggttatggagaacttaaggacgataggggagaaacggtggcggttgccacatccaccttctacatcaccacacgcagagaatgaatcaacaccatagaaattttatgggaaggattaaaaaggttggatggaattgattttgatgaacaccttcggccatgttttcagggttacaacctggggcgaaagtcacggaaaggctgttggctgcgtcattgatggatgcccggcggggttaagaatagatgagaattttattcagtctgaaatggacagaagaaagccgggaggggaattcagctcgaagagaaaggagcaggacaaggtagaaattctttccggagttctagagggcattactattggaacgccgatttcgatgctcatatggaatacagacgtcgactcgagaccatatgaggccctcaggacaattccgcgccccggacatgcggactggacttatttggcgaaattcggtataagagactggcgtggaggtggaagggcttctgcgagggaaaccgcagccagagttgctgctggtgccgtagcgaagctcattctaagaaaatacaatgtgagggtttttggatacgccaaggagatagccgggataaggttcaacgttgaagatcccgagagggcctttgagatcgctgaaaggagtcctataaggtgtcccgaccctcaaaaggaaagtgaagctgaggaggcgataaaaaaagccatggctgaaggagacagcgttggtggagtcgtggaacttgtagcaagaaatgttccagcaggacttggagagcctgtctttggaaaaattagcgcctattttgcctacgcattgatgggtatcccctcagttaagggattcgagataggaaggggatttgaggtttccaagctaaagggaagcgagaataacgatccaatcgtgctgagggacggaagaataagattcgctacgaacaactcgggcggaattttgggggggataacaagcggagaggacatagtgatgcggatcgcggttaaacccacgccttcaatatcgaaaaagcaaaaaagcgtggactgcgaaaggatggaagaggtcgagataagtgttaagggcaggcatgatccgtgcatagttccaagaatagtccccgttgccgaagcaatggtctctatggttctcgtggactgcatgctccttcagagtctgattccgagaagttttgtggggagaacatgatagaaaggaacgtcgaggtggaggaaaggataaggtgcattcagaggacaggggatgaggtcagagagctcatatacgattacaggaggtgtaatggctgcggtatctgttttttcgcgtgtccggtgaatgctattgaactggggcccgtgcacgagatcgcaaagggcatggaaatgccgcccgtgatcatagaccatctaaagtgcgcttactgcggaatctgttattctttctgccccttcaacgcattcgaattctatatagacggtaaattgattgaaaaatccacactgcccctctctcctgtgagatacacctacaagtacgagaactgcagggaatgcaccctttgctatagggtttgccccacgagggcgataagcagggagaagttagttgttagaagcggtataccagagaaaaatgagagattgaaaggctccatcaggattgacagaaataagtgcaatctctgcggaatctgcgcagaattctgtgaagttttcagaatggtcgagaaggaaattcttcctacagatcttatgccttacagcgatattctgatagacgatactaaatgcgattactgcaagctctgcgaggagatatgccctgagaaggcaataatggtagaagggaagagaatctcctacagcctgcccgagaaaattgcgaggataaccatagaccagaacgtttgctccaactgcggttactgcgaggaaatctgtccttacgatgctgcacgaactataaagccaattgagggaaaactgagcctttttgaagcaagaatggcgaggtgcgaccccgttggatgcggcgcctgtttgaagatctgcaggttcaacagggtttggtatgtctctgaagacaggaggagggtttacttcaacgagaaattctgcatttactgcggtgcatgcgagaatgcatgcccctacgatctgattctggtagaaagaaaaaattacttcacaaaagagacgatttacgatgccccgtggcgaaacgcgtgggaggatgctgttgataggattctgaaaaaggagagggttaaacagcctgagagaatttcggtggtggaagccgtgcaagctgcggttgaagaagtcgcccagataggtgaaaaagcaccaattaggggtgttgagaatctcgaaaaggttgaagttctgctcaggaaggttaggtttagaaaagccctcgaaacgggcgatatagatgtgttcatgagaggtgttagaagtgcacttggaaaggataaggggagcggagagtaaaaggcttgtggggaggaagatagttctcggggttacgggtagcatagcggctgtggagtgtgttaagcttgcaagagagcttgcgaggagaggtgcggaagttttcgcagttatgagcgaggctgcgcagaagataatccatccctacgcccttgaattcgccactggaaacagggttgttactgagatcactgggatgatcgagcacgtaaattacctcggagatggtggcttcgcggacctatacctgatagctcccgctactgcgaacacaatttcaaaaattgcaaacggaatagacgacaccacggtgacaactttcgccacaaccgctctcggagctggaaaaccagtgctgatagctccagcgatgcacgcagcgatgatagaaaacaaagccgtcagggagaacattgaaaaactcaagagaatgggagtcgagtttatagagccaaaattcgcggaggggaaggcaaaatttgcggacatcgagaagatctgcctgcatgttgagagaaagctttattcaaaggaatttttgggaaaaaaggttctcgtcaccgctgggcctacatacgagcagatagatcccgtgcgtttcataagcaacaagagttctgggcttatgggaatggagattgccttagaactttggcggaggggggcagaagttgttctcgtcagttctaagaacttcagaattgacttgccagacttcaaggaagttcacgtctggtccgtcagggacatgctgaatgctgtggttgaagagatcaaaggctgcgagctcttcgtctcttccgcagctcccgctgacttcgaggttgaaatgcgggataaaaagattaaaaccgcccctaaacttactctgaaacttaaagaggctccaaaaatcataaaggaggtcaggaaggtatacagtggagacatcatcggttttaaggcagaaacaggactgagtgatgaagagctggctaaaattgcgagagatagacttgaagccgacgagcttgcgatggtcgttgcaaacgatgttctcgaaaagggcatgggaaccgaggagacgagagttgttgttgttacagccaaaaggacacagtggattgagggcaggaaaagcctggttgctcagaaaatagttgagatctacgttcaggatcttctatgatcttctgcccgggtgcaataagctgcattttcagccccc

>Bin11_20

agaacctgctcagcctgaatccaaaggttagagaagcagcagttgtcggaattcccgacgagaagtggggagagaggcctctcgcaatagtcgttccgatgcctaaggtagagcttacggctggagagctcagacagcaccttctgaaatacgcggagcagggagtgataacgaagtgggcagtgcctgaaaaatacgtcttcgtcgatgagctgccgaagacgagcataggaaagattgacaagaaagttttgaggcagaagtttaggtaatgatccattttaaaatttttggccctaaaagcttagaagcacgattgcgatcagaatcggagtagcgatcgcattcacggctattccagctataacaagcttcgatgcgagcttcatcgggaaaatcgaggcgtaaaatggcagagaatgtcgcggatagtccattattgccgtgaatagaaatctgcctatgagcagagcaacaagacattcctgaacgcttatcaatccgtttctgagcagttcaccagctgtgagcactgaggacatcgggctaaatatgtgaatggaaatcagggccgcgaagttcgaggagaagccgaactgctttgtgtagatgtcgacctcctttgaaagcagctcgaaaaagccaaaaaagatgaggacggagattattgccgttattatcgtgtacctgataagaacttttttcgtcatttctgccgcagatttcaaagattctcttaccgcatcacctttatcctttctttcttcaggcagtttgagctccgcgagcggtttcctgaccttgattcttccataaagtattcccagagtcaatgagatgaacgcggttgcgaagctgagcgcgatgtagatggagccaacaaagaggccaagagctgcgagagagatcggaaggtaatagcgcaaaaggaagaagacggtggaaaagggccttgcaaccagtttataggccacaaccgctgaatcctcaattccattcttttccctgagcgatccgactatgctgagcccccctcttacatccacaatcgaaactatcgctgctaccgagaaaacagtcggaagatttgccaactccatcagtggagccacaggtctcgagatgagccttgccagtccgagctcaattagcaaatttgctatgaacactccaaggaagcatgcaatccccatcgtggctatcgagtaaaggatgctgtgcaattccatcggttaccctctataccttaattcagaatcctctgtgcacctctgcttttgtaatttccgaaatcatttctatgtagcttggcgatggatcaaaagctttcgctcttgtatcgtagatctcgagctttattcccttctttgcgatcagattgcagatctgggcaagatcttcgtcaacctttccgaatagaggaacgttggcctttccgtcggtgatcagtattcccctgatgtgtgcgctctttatgttgcctgttatcgagatcagcttgagaagcgctgaaggcagtggggtctttccgccagtgggaatagaatcgatagcatcgaatatctgctcatatctcttcgtcggtttcacaaccacttctgcctcgtttccgcgaaaaactatcagcgaaagccagtctctgcgctggtaagagttttccacgagtttctttgcaattcccttcgcaatgcttatccttttctgcaaccccatgcttccgctcgagtccagaattatcgcagtaattcttggtgctcgagttctgcggacgttgatcctgatatcctcatccctcacctcaatcggaaatccccgcatcgcggctgaattcagggttgccatgatgttgatatccctaaatccgtttaccgctggaacatatgaaacaggaattccctgcttttccccgataagcgtaatcctgcagtccttagatgctcttgacccgaaattctctgccagatcctttggaatcttcggttccattttcaaatcacaggaatcaaatctcatctcggaatttccgcagcaggtttcctgtctgttttcaggttttcctgaagcctctttagattcagtcttgttctttttatgattttcaggaagcttcaggtcgggtatctgcggcttctggaaaggtcttgattttatcctgtgaggaagcgttagctccattgcccttctcagatcctcgatgtcaactctctttcttccctcgaacgcagctatggcctttgcagccttaaccgttgcaatttcagctctgtgcgtttttattcccattccgattatggtttcggcaagaaggttcagcagttcttcatctatctcgacttctgcgagaattctccttgcgtttgaaattgaagtcctcagcctctcatcctgagctttgtacttctcgtaaaaagaaatcggatccttctgaaattcggttaccctttttacgatttcaatcctctcatcaacatttgtggatgcttcgactgaaacgaacattccaaatctatcaagcagctgcggccttagttctccctcttctggattcatggagccgacaagaatgaacctcgcaggatgcctcagagatattccttctctctcgacgacattccatcccagcgcagctgagtcgagcaagacatcggctatgtagtcctcgagcaggttcacttcgtcgatgtaaagtatgttcctgttagcttccgcaagaattcctggctgcaaagctcttattccttctttcagggctctgcttatatctattgttccaaccagtctgtcgagggttacgcttaacgggagatcaataaccctcattcgccttttctctgtcctgaattctcccctttcgaccttatccctgcaggcatcgcacatctcggcaacgctgcctggattgcagttgaagggacagccttcgatgacttcaatttctggcaatacctgcgaaaatgctctgaccatcgtcgacttccccgttcccttgtccccgctcagcagaactccacctatacttggatctattgcattgcagatcagggcgagctttgccttctcctgcccaacaattgctgtaaaagggaacagcttcatcttaacacctcctccagaatcccgtctatttccagatacttctcctcaacctttttcttcaaatcgccggctttccagtatcccctcttctcagcctcaagcagcctttccagcatctcgcgtgttgcatatacgttctgctccctgagcctgttaaagagctgttcatccagaacaaagcgctcagcaatcttatcccagagcctgctatcgacggcattggtcgttgctgcgaggcccaaaaggtactcaatcctgtccgcaattttttgcaccccgctgaatccgtgtttcagcatttcctctatccactttggattcagggttcttgttatcgttcccctctctatcgaatccctgacactttcgaccttcgcgatctcctttgttgaatccacaataaccatctcggccttttcgcccttaacaagctcaacggcttttgaaagacctccaaagaactcgtagtagtgatccagatcggttatttcataatccgtggagcttcttgtttgcgtcacgacatcgacagtctttaatagcccctcaaatagctcccttgcctcaatgccatgcgcatcttttccgtatgcgtagcacatcgagctaacgtatgcttttgccagatcgctttcttcttgccaaacactatcctcgacaagctgcagcatcctagtgccatactccgttgccacggggccgaagattcttatccggcttttcagctctctagcgtgctttttgacaaagttcagactctcgggctcatcgagctctgaaacgagccttacagctttatctataagctcgataagatttggaaacatctccctgaggaaaccgcagatcgtaacgacaacatctattctcggtcttgcgagctcttcgaggcttttaaccttcagctccttttcccaagctgttttgtgaatcagctcaactccaagtagctcgaagatctgtgcaactgtctcgccaaaagtttgagcggtttcgaatccccagagaactaaggcaacgctttcgggataccttccatgcctctccagatgtctttcgatcatttctcttgcagcccttcttcccctttcaaccgcagcttcagttggaatccttaagggatcgaactggtagaggtttcttcccgtgggtagagcttccggattccttatcacatctcctccaacggaaggctcaatgtattccccgttaagggctctgatgagattttcaagctccgctgcattgtttgaaaagttatctgcaatttctttgcagtaattcagggttttctcgaattcagctctggtctttcctgagaaaaactcttccactatcctctttgcttcttcatctatttctctcatttttcttggatctcctgcaatttcctcgaatttcagaccttttttctccgctatgattctgtgaagcgatttaatttcgcccctgtcgtatctgctcagaagaagggcaaaatccttcatctcgtttgcctcgtatcttttaccaagaacgtgaagccccttcgggattattgaacgcttgtactcgtaaattctggcctcaagcttttcaatgtccttttccattcccagtctttcagccagctcgaagatctttgccttcgctgcgttggcccttgcagggttcttttcagctctgaaatcctctatgagctcctcaagccttgcgtattcatcgtatagctctgcagtggtgtatggcggggaattgtagctcaaaagcgttgcatagctccttcgtttggcaatcgtggcttctgaaacatttgttacgtggtaaacgtatatgtgcggcaccgaggatatgagtacgtcgggccagcattttgagctcagaccgcactcctttcccttcatgaactcgagcgtcccgtgggtgccaagatgtattatagcatcagctccgaagatcttttcgagccagaagtagaatgcgaggtactggtggtgcggcggttttgttttgtcgtgcacagcagaaagtaaatcctcgctactttcaattattttcctcctcgagggctgaatcccgattgcgatgttcccgagcaaaacaaccgggatcagaattccatcttcgtcgaccattatatctcccggcggttctccaaagttctccacgacctctcttctcagatcctctggaagctcatagaaaaattttatgtaatcgcttttgctcaatctcggacattctattgctttttccgagaatagctttggattgaagagctttttctcgaggagaagttcttttacgttcatcctttcaaccctgtagccctcatcccttagtctttcaaggaccgccttcaggctttcgaatgtgtctaagtatgctgcattgcccagattctcctcgccgggcgggtagttgtagatgactattgcaaccctcttctccgaattgggcttttgccttaaactcacccatttctctatcctttcgcagaacctctcgatcctgtctgcaatgggaacaacttcgtcatcatggatcccagctattggcacaggctcaactgcgccgtccatctcgggcagtgtaacgcttgcaaataagttgacgatgctcagccctctctgctccctatcccagtcagatagcttctggttgaacatcaaagctggtgtgaaaatctttgccctctttcccttcagcagctcgattgttttccttggatctccccccatgggaccaccgttcagtctgaaccagagaaggctaactatggcatcgacatcgttcaggagctcaagggcattcaggttcagaattccatcgctgtagacaggagctacgctgatatttttgtcttcaaaccatttaacgatttcctccagagttttctggcactgctcgaaatgcataccgccgtaaaaaacgattccaatttttggcctttcagcgacaggtctgaaatcgaagccgtgggctggatggtaaaatccgaactctggaaactcttctggctctgcagcttttacatcaagccccttaactttacaaaggtgcaggaacatgtttctgtagttttcgaagcccccatttgcccagtatctcagcgttttaacatagtttcttgcatccttaagcacaccaaagggcaggatttttcctgctgtttcaattgccttctgtatgctttcaattctcttctttagagacgccggatcggaaacaaaagaagaagcagccttagccggcattctgaactttcccagcttcgccttggcagcaaggctgcttccgccgaccaggcatatcaggtccttttctccgaaatcaatttcgttcagaatcccgggatcgccgcgaacatcgattgcgatgatatccgcccagtcaacaaaactctgcacttccgcaatcttatatttcggcagatcgtatgagtatatcaggtttacgtcacagaattctgacacgctttccattgctttcttcaaacttttgttaacaattgttgatatcagcagtatttttattcgcttgctcataacttccactcatcttctgcgataaaaattttttgaattcacgtcagatagatttgcaacaatagttcgttttcatcgatcgctttttcgccgtttatccattcccagtactccgagactgtgagcaaaaagtatggctccaagaactgcattggcttgtagctttccttgtagacagcatttttcttcaccgcttcaacctccctgaaagcggggtccgattttatgtcctcttctgtatacggggcgtagtagcttattatccagatttccggattctccttcactatccactccttgctaacctgaacccaagttgagttgcactctggggaaacaacctttcctccggcggaatttatgaagatgacgtagggctgatccgaagcgttgcaggctatgtaagtgggagttgagaaggaatagagaacctttacgtctctatttttcttttttgcctcgaaatatttccatttctcctcgatgtatccgcaaaccttctcggctctatctcttgcatcgaagatttctccgagtagcttggcctgtctgcagatgtcttcaggatatttgtttatgtatccaatcctcactatcgggatgccgagtctttcgaacttcttcgcattctccttgtagccaggtggctcatcccagaccactatgagatcgggctttagcgcaagaatcgcttcttcgtttatcctgttcatgtcacccacagacgttatctttcccacctctggatcgattttgagtatgacaggatttttctttgcgagatcggaaaaggctacgaccctttcctttatcttgagcgcatagacccaggattcgcctgaaagaaatacgaccctttttggtgtttcattcagaactatcctgtttcctgccatgtcagcgatttctatcactcccttcgtatcctccaatttctgcgcgcagcagctaagggctacgatgaccagaaagattgcaaaatagacccatttcattttaccagcctccaggcctttaaaacgtcggaaattttttccccccactcatcaacatcttgaggggagagaaactctacggcgcttccttgaacatccccagctatttcatcctccagaatcccctcaatttccccgtagatctccctcaacctcctaagctgctcctctctcgcattccagattcccctctcattggcctcgatcagtcttctgacgatctcctcgagtgcgtaaacgttgttttcctcaaagaattttctcatctcctcatcaagaatatatttttcggcaatttcctcgaaaatccagtcatcaacaagccttgttgttgcagaccagccgtagagatgcagtatcttttttgagaactcgttagcgcccctgtagccgtgtttcttcatttcctccatccataagggatttaggatctttgcccttgctatcctctcgatctcttcccttatctcccttaccttagtctcggaagcgtcagttgtgtccgtgatgcagatttcaacctctcccctcagc

>Bin11_21

ctatcagatactttcctctctcaggaggatattgaaaagcgcaggagttacggcaatcgtgacgagcgaggcggacaagtttttgcccacggcttcaaaatacggaatactcgaatacatttgcgatggaatgatatgcctcaagatgataaggaagagcgagctcgatgagccgacgctcggattggaagtcgtgaagatgaggggtgtgaagcactcgagaaggccgaagccctacacgataaccgagaggggaatagtggtttacgaagctgcagaagtcttttagcgtttggcaaagtagagcataacagacaagtagacaaaattattttaatttcatcccgttctttcagccatgctcaccgagatcacgatctactgcagaggtggacagggcggtgtcacctctgcgaggctcattgcaacggcagcgatgctcaaaggactatacgcccaggcaatacctcagtttggccctgaaaggagaggagcgacagttaatgcttatctgagaatctcggaggcaccgataaggagaaggtcctcgataaggaaaccacagggaatagtcgtatttgacaggaaaatagatgtgaaagtagaggccgaatttgggatcataaattctcccgaaccgagaagaattgcgaatagaacatactacttggatgcaacggaaatcgcaaaaaaatacgagctcgtaaatgcgggctgggcgatactcagcgcgcctatgagcggagcgattgcaaatgcgctgaaaatcgagttccactacctgagagaggctttctggaaggagcttggagagaaggctgagaaaaatattgaagctgctaaagaggcttacgaggtggttcggtggacttagaaccaataatctcgcgggttaagctcggtacagcaggagagacaggactttggagaaattacaggcctgtcgttaacagggagttatgcactaaatgcaaaacctgcttcaactactgccccgagggggttataagcgaggagatcgagatcgagtacagattctgcaaggggtgtgggatttgcaaggagatgtgcaggcagaaggctatagagatggttccagaatgagagcgctgctcacgagcaatgaggcgatcgctgaagctgtcagaatcgcgaagcccgatgtgatagcagcttatcccataactccgcagtctcccatagtcgaaaggcttgcagagatgatagcgagaggcgagatgaaagcaagcttcgtaagagttgagtcagagcattcggccatggctgtgatccacggagctgcaaccgccggagcaagggtttttacagccacctcgagccatgggcttgtttacatgtacgagatgtgctggtggatcgcaggatcaagacttcctgccgtgatggcagttgctacacgctctatcggtgctccctggaacatacacggcgatcacacggacatagtccttttgaggga

>Bin11_22

gctaaccttgctcgtgctgggttactcgatatacaggttgaggggggaaaggctcacctctctgtttggagtgctatcggcagcgatatttgcagcccagatgcttaactggccgattccgggcggaacctctgcccatttcgcgggtggagcacttgctggaattctcctaggcccgtatgctggcagtcttgcaatggcgatcgtgctgacaattcagacgctcgtcttcaacgacggaggcatcactgcttggggtgcgaacgtttggaacatggcaatagtaaacgttttcttgggttattacatctacaggaccttttctaggtataacaaaaactttgcagcgttccttgctggatggctcgggatcacggcagcggcgatctttgcgggaattgaaataggactatctacaagctttgcatacggattgagcgtcaccgtgccagtaatgggggtatggcatgcattgcttggcgttattgaaggcataatcaccgcaggtgtcgttggctacatagcaactcgcagagccgaccttctagaaaagaaagaggttggaaagacctcttttgccctaatagccgcactaatagctctatcgccagttttcgcataccttgcggaggaagttggctacgctgaaccgcttgaaaaagctgcagagatgatcggacttgaagaagagccgatctacgaaggtttattccctgactacacgatctctggcctcgatccatatacgggaacgttgctgagcggcttgctcggagtcctaatcgtgctggccttggcttatgcggtgaagtatgcacgtggttctcgaaaagacacttaagaatgcctcctcatattttcagaattttttcatccacgagtacagctctaaaagcacactgcattcaattgacgcaagaatcaagcttctgggcacgtttttattcgttatcctcgccgtttcaactttcgagccaaaaaaattgcttttcctcctcctctccttaattttgatttccgcgatactcggtctgagccttaagaacatgatcaggcgagtttggcttttcccactcttctcattcgccgttgtttcccctttgctgctgcaagatataggatatcctctcctcttcacgttgcgagtccttatagccgttatagcagtccagatgctcgtgatgagcacgagcttcgcagatatctgctccgctttaaggtcgcttaaggttccagaagtattcgttcacggtctctggctggcttaccgatacatcttggtggtttttcaggacataatcaacattctgcttgcaagagagagcaggagagtcgcaagaggaagtcatctggagctctggaggaaaggtggtgaagctgtaggtcttttctttcttagaagcattgagagagccgaaagagttcagctcgcaattgcctcgagaggtgaaagaataatcgggagcaaattacaattcggaactcttgaggcaatctatatatcactttcagccttcgtggctttgtggtgcatagccctctgataagcgcgaggaacgtcagctacgcctatccagatggcagcatggctttaagagacgtcagcatagagatctacaggaatgaccgagttggaatcatcgggccgaatgggagcggaaagtcgacgctgatctttctgctctcaggccttttgaggccgactaaaggagaggtaaggatattcggaatgttacccgataaaaagaacgtcgaaagcataaggcgaaggataggagtcgtctttcagaatccggacgatttcctctttaatcccacagtgaaagatgagctcctctacgttccgaaacagctcgaaatggatgaggagcaaatgctcagacttctttcagaatacgcaaagaagtttgatctcgaagaaatactgcaaaaaccgcctttcaggctgagcgggggagaaaagaagaaggtcgagatagccagcgttttggtttacagcccggaggtgatattcctcgatgagcccacagccaacgtggacggaaagacgaaaagaatagttgcggagatgatgagaaattattcgggcacggtcgtaatagcttcgcacgagatggaattgatctgggagctatgcagtcgtgtggtgttgatgtcaaaggggaaaatagttgaagagaccacggttgaaagtctcagcgatgaaaagcttgaggagatcggcgtgatttaggccgggtagtagtagatcttatccgagtcctcctttttcaccaatttcctcttctcgagatgatcaagatgggcgatcgtttctcccaccgcaaaccacttctgcatcgtcggaagttcgttccagttctcgtaattaagatcccaggttattttctgcgctatttcccaggcagtactcgaaccatttttcacggcattcagagcttctttaagccttcttctatggtgctcttttagctcttcaatccttttcctgtgatctccatggaaatttctgtgcccgggtaacgtcttttcaacttcgaactcttgaatcttttcaaggttttgaaggtatttgccaagagcatcatcgagcgtttcccagtgcgttatgttcggggttatgtcaaagagtatgtgatctcctgagaaaaggagcctcttttccggctcgtagaggcagagatgtcccggagtgtgaacaacttcaagcctgtagtccccgaactccagaacatctccttcatccaagaaattgaactcaattgcttttgggctgtattttacagcaggatgaattctcgccaccttctccgcctcatccggaggaaagccattcttcttgtagaattccacgagctcaagccagtattcaggatgccttgcagaatgtatcgctatctccgcatcagttttgctcatgtaaacctctccacgcaatctcccagcaaggccaagatgatcggcgtgcaggtgtgttacgaagatctccgctctctcaagctcaactccgatctgcttaaggccattgagcatttgctcgtagcagata

>Bin11_23

ctaaagaaaagggaaatagacatctggaagcttgaggacagggagcttgcatctgcaatcaggaagaaagttgggataatgctccagagaacattcgccctcttcagcgaaaagtcggctcttgagaacgtgatggaagcgataatcagctcagaaaccgagagcaggatttccgttggaaccacaacgagaagggacttgctcgagagggcggagtactccaacaacgttgcgaacagggcaatggaactgcttaaggccgtgaatctgggacatcggattctaacaatagcaagagaccttagcggtggggaaaagcagagagttattcttgcaaggcaaatagcgatagatcccgttcttcttcttgcagacgagcccacgggcaccttggatccagaaaacaccagaataatactgaatgcgctgagaaagtatataaagagtgctggaaaaacgatgatacttacttctcacattcccgaagttgtcaaggagctttcagacagggttctatggttgcataatggtgaaattctggctgaaggcagtcctgaagagttaatacggaaattcttagctcagatcagtgaggaggagctcagggccgaattcagatttgagagggagattctcaagctgaaggatgttaagaaatactactattccgtgagcagaggcgtggttaaagcggtcgacggcgtatccttcgaagttaaggagggcgagatcttcggaatcctcgggccaagcggttctgggaagacaactctatctaggataattgccggaataaccgaagtcactggcggagaagtccatgtgcgcattggtgacgagtggatagacatgagaacccccggaattagtggcagaggcagggccatgccttacattggcattttacaccaggaatacagcctctatccacacagaaccgtgctcgacaaccttacggactgcataagcctcgatctgccgtcggaattcgcaaggattagggcagtggacatactcgcgggtataggcttcagcgaggctgaggcggaggcaatactgacgaaataccccgatgaactcagcgagggccagaggcacagggtggccttagcacaggtcatgatcagggagccgagaatactgatacttgacgagccttcgggcacaatggatcctttaacgaagatagaagttgcgagaacccttaaaagagtcagggaagagttcagaacaactatactgataatatcccacgacatgcagttcacaaagctcacgtgcgacagggttgctctgatgcttcaggggagactcgaaggcattggaacacccgaagagattatacagaggctccaggaactggaactgagaatagcctgctccgttgaggtcgcagccacgacggaggaagaggggatgatagagaaaattgataagggaggaagttgcggcgcttaagcccttgaaaatcccttttttgcggcgattcttatcgaatccagaaggtcattcttgggaattatcgtaacaattggaacgctcagtatcttttcaagcacggggctaagaattggggcacaaacaatagtccttgcgccatctctttctgccctgactgcagaaacgatcgcttcatcaaaggagttggcgggatactccctaaccttgaccttcttaccctctatctctacctcgtagctcgtaagctgattgagcgcagatcttgaggcaatgacagcaacgaattcatcggagctctttgaaagtcggactatcgtttcggcgatccttctaagagtactcaaatttggctctctttctccagaaagaagtttgtaaatagtgctttgcggaattcctgcttccttagcgaactccttaatgctcatcctcaaccttttccttacaatgttttccaactcctcaccaaaattcccattcaggatgagggaggagatcatgctgttcacatccacgcatacactgaggcaacaagctaataaatttttgcctttttaggaactaacaaacgaagtagacccaccattcccttcttggactgatctcaaaattacccaattcaaccctaaagtcgcatcccagcttgaattttccttttttgagtttcagagccgtttcaacttccttgggagaattttcgacgaagacgattttgtttacaacgtcaaagaatattctcccactataaaaatctatgacggtgtttctaattgcataagtattcaatatttcgcaaccctccctccaatatcctcctgcaaaaattttccttccgtttgtctccccaatctctataggcatcctcaatacctccctgtatatcttaggcggatttctgtcctctatcatcaccgcatccccggttaagacgttatttctctcagaattcgtaaccaagagttctccgtagtccctcctcgttcttgtaatcggcaacatgtcctcccttataacaggaatactgagtggcatcacgaacatgtcatcagagatgcagaagctacacgcaactggccctgcctcgcttaaaacatacagattgcattttttcttcacaaagctatttatcagcctccatctgttctccgtcaatgaagagaagctgaaaatgaaggttgcacttctgagtttttcccataaaagtctgtgaatttccttaacagccctctcaaacggctttcttaatatcctcctgaaatcttcgctgatttccctctcatcgaggaatttcaaatcctcctttatacccctcgaaagaaggtttatgtcagcccatcgcgaaatggttgaagcaggggcggaaacgacttctactttgtccatccccagtatttctgagagtacgtccagcctcctcgaatttggataagtcgaaatcagatagtccgcatcgaacatggaaaccacaagtctctgagaaaattccgatgagagaagaacaacgtttccattctcatctattccgtcttctctgttcttcctttcctgaacaaaaattaccactttacctctcctttcaagacctgcagagttgaatatggcgtacatgcctggcatccaattattagtcaacatttcctcgctcagcaagtaatacttgggctttcctgacgtgcccgaggtgtgcagtattatgctgtttctgaaattttctggagaaattgagaagcttctcggttccgttatggaattttcaatctcctcgaaactttttccagtatttctttccgcataatcttcaaattcctcagcggtaaattctaaccattttctccagtccggttttctgaactccttctttccaagcttatccaatttttcttgcacagctttgtcaaagtccctcatctaatgggtcattacaatcttagccggtattagacttttctcactccggacaaatattggggagaaccgctccccttttgaaggaaccattattataggcaggcgcttattaatccaaatgtctaaaaatattggtaataataggaaaatcaaaatttaaaattttcccatttagggtatctgtaaggcaaaaatggaaaaagttataacgtttacctcaaactgttcctttgggtgagagtatgagaaggcgaacctatttgctgctcgcggtttctgcgttgctggcagcgtttgccttctgcgtttcgcaggagaagccatcagaaccgcagctaaaggaaataaaggttagttaccagccaagctggcaccacacggcacttttcgtgataatagagaagggatgggatgagaaggttctcggagcgaaaatttctgcaaccccgttcccatcggggccaccgcagatggaggctttcgctgctaaagaacaccagatagcttatgttggcgcagctccgccgctttctttgctttcaaagggttttgatgcaaagatagttgctgttgcaaacactgagggttcttcattggttgcggctcccggcgttgaatacacgggtcccaagtctcttgagggtaagaaagtgatgacgttcccgcctggctcaatacagcacacagttttaatggattggcttaaggatagtggagtcgatatctcaaaagttgaaattaaatctggaggacctgaagagatcagggaagccttaagggctaaggctattgactttggcttccttcccgatccgggtccatacgttgccgagctcgaaggctacggcaagatcgttctgagctccccagagatctttccgaaccatccgtgctgcgtggtcttaatgcgtggagatttcatgagggaacatagggaactcgcagtgaaattccttgcactgcacataattgcgagtgagtacgcaaaggatccaaggaacaaggaagaaataaaggcaatccttataaagtggctgaaggtaggggaaaacgttgcaaacatgttcccgggaagtacgaatcttcagacggatccgagaagtgaaaagtggttgaaagggcttgacatgctctgcgaggcacaataccagctgaagattacaaaagatgcctcaggaaatcctgtaaggattggcccctctgaagttgtggattccggcctctaccaggatgcattgaaattggtgtccaagataaaagaagagctgggactgaaatgaggatcgaaagagtaaaggatttttttattttcattgcctcaatcgggatttttttggccatatgggaagcatactactggatactgattaaaaattccttcgtactggctccgccctcaaaggtcctggtaacgttcactcagctgcttatcggagatccaagattctccgaaacaggcttcaacatgcccttggatatgatttatagcatgtaccactatctctacggctttaccttcgcaataattgtcggtttctccttggggctaattgggggatggtctaaaaaggttgagaagtttctctatccgatcatagagctcatccgcccgattccaccaattgcatggatcccaatagcaatcctcatgctgaagttaactcacacggcagctgcattcattatattcataggagcggtattcccgatcctacttaacacgatgtacggcgtctcttcggtggaaaagaaatacgtggaggccgcgatgacgctgggtgcgacgaggacgtccacactgctgagaaaggtaataattcctgcttcgatgcccgcattcttcaacggcctgagagttggctcgggagttgcgtggatgtgcgtggttgctgcagaactttttggagtatctcaatatggtctgggatacaagatacagctagcaaggctttatcattccccgga

>Bin11_24

ccgaagacggatctggtttttataaccgaaagaagcaatccagttgagttccttggctattcggctacaatcttgcttttggcctacttatctttcctgccagagaaaaagctgatcttttactcgattccattttctctgcccttctactttttcagcccaattctgccagtccttattccgctactcgtctgctccgtattcgggatgattaaaaaagacacatctgccccactcatattcaccgcgctgtcctgcttcataatcccagagttcatcgcgatcgattcaagactgaatactgttttcaaattctatctttgcgcatggattttgctctctcttggagcagttttgaggctgaaaattattggaatagggaaagcaaaacctttaatcgcacttcttcttatcgtaagccttgcgtatcccatcgtcgcgacaccgctgcgataccatactgcggaatttactctagatggcatggcgttcacgaagatatacggagaatacgaagcgctccagtggctgaaggaaagggaaggcgtgattattgaagagggttgcacgcatggatacttctgtgcctatcagtacggggggagggtggcggtttttacgggaaatccagcagtggttgcatggacagggcatgagtttcagtggcgaagggattacgataagatagcggagagggccaaagatgtcaggaaattttacacttcaagggactgcgaagaaatggccgaaattctggagaaatacaacgtttcttatgtgttttttggctttgaggagagaagacttttcggttcggtcgaagaaaacatcgaaagatgcatcgccaaggttttcgagagtggaaacgcgaagatattcagcactaagcccgtgggctaattttattggcgaaaagtttatcatttagagtgcaaattttaatcatggacgagaaagatattaagctgatatcaatccttaccgaggacgcccgcaagaccctccacgagcttgcagaggagctcggtttgtcggtatcgagcgtacacaagaggataaggaagctcgaaaaagacgtaattgaaagatacactgtcatactgaaccccgagaagttcaaccagataactgccttcctgctcgtgagcgcagatcccaagagaacagctgaaaagctcagggacatacctcagataatagagctctatcagaccttcggtaacttcaatttcatcttgaaggtgagaggaggcagcattgaggagataagcgagatcacgaatatgatttcgagcctcgatggtgttgtgatggttgagtgcatagtggcaacaagaaggcttaaggaaacggcatggaagccggggtgaacttatgaacttcctgcaaacatttctttttattctgactccggcctttttacttctcggcggagccctgatccttcaggtttacaacgcatatttgagaacaaaacagaggtttctgatcctcctgagccttggtttttttgcgctggttgtaggcggggctttgcctgtgctggcctacgcgctggctatttctgaaattctctacattgcaggaatcctgctgcagatctgtggaatagccgcgatctattactccacagtgagagaatgaaggaggaaagaatagctcagaagtggaggctgatcaagctttcgaaaatcagaccgctgctcgcctaccgactcgtatcagttctcgctgaaaggctcggtgatgaggtttacaggatcgcatttgattatggaagggagaggggagaatacattgcggaaaacctgagattaagggatttcggagaagtggcaacttttttgtccatgatttcgggtgtaaagattgaaaagcgggaagaaggagttcttttcctgtcgtgtcccgtgaatgccttggaggctgtgaagtcggataaaatttgcaggggatttttggagggatttttcagagcttttggaatagaggtagaagccattccctcctgtggtgatcagtgcaggattttcgtcaggcaaattcgctcctaaagccccctatgacgaattccacccctattgcagcaaggaatattgcaagcacctttgcaactatatcagcgccgctttttccaagcaactttattatccagccgctgtagatatgggtcagtcttacgatagcgtaaacgaggaccgcgcttaaaacaactatcagcttggaaataagatccgatgcctgcgagtacatgacgattcccgcggtaatcgctccgggaccagtatacaagggaagagcaagcggaaatacggctaaggaatccacttctgcactctcttccgctctttttcggtattcctccctaccatgggctccaagaaggatgtcgacagagattatgaaaagaaggattccgccggcaattctcagactgtttatcgaaacgtggaagaaatcgagtattgctcccccggaaatggtggttattatcaatattgagagggctatcgtcgtcatccttttcgatatcttctccctcagctcttctttgaacctactggttagcccgatgaaaattggcagattcccaggcgggtcaacgatgacgaagaatgttagaaagcacttgaagaaatacatcaggtagtccatagctcttttgcccttttaacgttttcaagaagcttcagggcgcttctctgggatggaaatgcgaaaagaccgcagtctggcccaatgtagctcaggagatcagcgaattttttctttgcaattttcaatctttccaggattttctctgcaggctcaaattcctcaatagccagcatggcaaggtcttcactcttccatgcgttcacattatgcctctcgttgaattcagaaattatgcagtctatgtccgtcctcgcgactcctattctcacccttttttcggccgactccagcgcttcaacgtcgagggcatcaatcttcttcggatcacgggcagtctcgattcctattatccctatccccgtctcgaggaagcgatcgtaaaagaggggctcgtgcagatgcacttgggtgtcggcattgagcttgaaatcgtaagctctctcgatctgctctttgctgggctgcaggtcaggagcaacgcccaaactgggttcgtcgaagctcagacaaacaacgttggcgtaccttgtggcatttcttgcaaacctgtaaacggattccgcgaagttttgcagaacatcctcgtagataacccctccgaattccctgtaatagagctcgaatactccagttatgcagaccctgagcctttcaaccctcatcttctggacaaattccacttcgggaattgttgctcctttttcggatatcaggtaggcatcatcctgaagttctggattctttatgggttcgagaaacattctaaccatgtccctgaactggggatagtttggaacatccacgtactttgatttcatcagaaaggctctttgaaccatttcctcgtattctttgctcctgaaattcttttcagcccaatctctggttatcccgagtggtagcgggaaactgccgatatcgtcggtaagcataccttaagttgtagctcatgatttatttcattttgctcccaccgaaaaaactaaattttagcggtgtattttcgaatatgaggatagtttggctggggcactctgcttttctgcttgagggaagcaaaagggtgctgatagatccattcataaccgggaatccgatggcacccgtcagtgctgaggatataaaggccgacgtgatcctcgtgactcacgg

>Bin11_25

aggcttggagagatcgacttcaggctaacagaaggggcaaacgaaagaattcagctttcggcattcctagcctacgtttcgacgctttctaaaaagtgatgtggtcggaatacaggaaaaaaagattggagcgctacgagaaggcaaaaaaggaaggaaaggtggatgaggacataattcccctccttgagagaatcaacggcagagagaactacgtaacgctttcaagctgcagcgggaggatcgctgtcatcgaccttgaaagctttgggaataaaaaatcctcgcgatttctgggaaagtggcacaatttagccattttcgaagatgttctcgagtccattcgtggctgtaaacggcaggggtggctgattcaggatccgccaataatacacgttgcctgcagagacctggattccgctaagaatctgctgacggtcgctaacaactcgggattcagaaggagcggaataatctcgctgaagaattatgtcgttgaaatggcctctttcgagagactggagcttcctgtggtttttgaagggaaaaagatagtggacgacgactatttgcggatcgctgtcgatattgcgaacgagaagctaaaaaagggaaaaataaagctgaaaagacttgaggaaatgctcacttccttgtgaaaagcaagccaatcgcggagacaaaaagggcagcgaataccgctgcggcaaatctgtatattgagtctctgatctctggattcgcaacgtaaccagcggtcccccagatgaccaagccgatggaaatcgcgatgaatgcggaagtgaagtgttttccaaccctccttccctcactgtaagcatctatcgctcttgccatagctattgcgaccgcagaaagcgtgatccatgctatgctgtagaaaatgaaattcgcaaccgccaagtgcggaacagtgtatgcaatggaagcgttgaaaccagtggcaaatccaatgagcatcagaattcctgctccgagatagaagacgaaggatagccttccctcgctcacgctttttctcattccctccacaatgcttccgaagtatttgtccaaaccatatgccttggccacgaaataaatacccagaaaaagaattatccctccaacaccccactcagggtgctgtatcaggagaaagaacgagtagacgaggaaaattatacctacgggagcgagagtgatccttgcaacctttggttcgttgaacatcctcctgagcaaaaagtaagtgctttcaagtgtcttgctctgctttacaacgaccctgtggagcagatctactttaaatctcgagtaaacgaggggcattacgaattcgtcctcagaaccatcagttacgacgatcacactttccggatttagttttaataggatcatgtcgagctgttcggcgatcttcgtatccgaaaccttgccaacgtttttgtcacccgtaacgacgacgacttcgcagtcctctcctttctggatgagctcgtcgtagagcttgatcgcggaaaacatggcattggcatccgaatcctcgggatcggccagcatgagccttactgcagcctgaagaacgttttccctcccaataacaggagaagctatcccgactttctgtccgagatcgtcgtctctgtcaatcgctattataaccttctttgccacaaaatggatttgagcaaaaaatatataagattatctcgacttcccaaacatttctctcgctatcgtgttcagctggacttcctttgttccctcgtagatctccgtgatcttcgcatccctgagaaatctctcgacctcatactctgcgatgtagccgtagccgccatgcatctgcacggcctcatcacaaacctttacggcaagctttcctgcaagatatttggccattgaggtgagggcgggatctggcttcttggcatcgaaattccaggctgctttgtaaactagaagtctcactgcctcgatctctgctctaagatctgccatcctgtgctgtaaagcttgaaaagctcctaagggctgtccaaactgctttctctgcttcaggtagttcacggttctgtcaaatgcaccctgagctattcccagtgcctgcgccgcgatctcgattctgctctcgttgaagaattctagcacttggtagaaacctctgttcagcgttcctataacggcgctttcgtcaagcctgaggtctttgaaagcaacttctcccgttggagatgcccttatgccgagtttgccctttatcggagttaccctgatctcatcgcatttctttgccaagaaaagcgtgaatccgcggtagcggggctgtgtctcttcggttacagccagtatgatgaagtaatctgcaatcggagcattcgtgataaaggtcttggagccgttgatgacccattcgtctccatctttcacagccctcgttctaatggctgtgagatcgcttcccgcttctggttcggtgtagcaacccgcagttatcgccttcccttttgcaacctttggaagaacttcgctcttctgctcctcgcttccaaacatcatcaaaagctctgttgagaagtctgaaagtatcagagcacttcctatggtgctgtctgccctgcaaaattcttctgctaccagtatgttctccaagcaacccattccagctcctccatattgctcagggaagtgcaatccgataaagccaagttcacaggcctttttccatagctcctgcggaaactcctcttttttgtctaactcctctgctttttccttcaaaaactccttcgtcgcgaactccttcgcggctctccttatatcctcctgttcctgagtaaactcgaagtccatggaaatcgtgctgcgacttctttaactaattttcgattttgaacccagagcagcagctcgtgccacatgggtggctcagatcagcatgttgagcccctcgacaaatgcctcaacggatgccataacgatgtccgcccttgctcctcgagcggtgacgatcttgttcccgcgcttcagctgaaccacaacgtctacgagcgcatccgttccgccagttatggcatctacgtgatagcttacgagctttatgtcagagtagtcctttattgccttccttatcgcatttatggctgcatctacaggtccaacaccaatcgcagcctcgatcctctcctcaccgttgaccttcagcttcacgcttgccatcggcatagtgctctttccagtcagtattgcaaagtcttccagcttcacttttttttcgctctttatctggagaacagtctctatgatcgttctcacgtctgcatccgtaaccctcttcccgagatctccgagctcctttattcttgcaagtatctcgtccatttgctcctttgttgcagagtagccaagttccttgagtattgcctcaacacttgcccttcctgcgtgctttccgaggacaattacacgccttcttccgacagtctcaggagatatcggctcgtaggttctcacatcctttagcagtgcggaggtgtgaatcccgctctcatgcgtaaacgcgttctcgccaacgattggcttgttcggtggtacgacaactctcgtgaacttctcaacgagcttggagagcggataaagtctttccttgattatacctgtgtttattccgtagaggtattcgagaaccatgcagacctcttcgagcgatgcatttcctgccctctcaccaattccatttatcgtggcatgaaactcgttcgccccgctcttcacggcataaacagtgttcgcagttgcaagtccaaaatcgtcatggcagtgaatcgcgagcgggctcctcaattctttccttagcctgctcactatttctgaagccctctcaggtgtaaggacccccacagtgtcagcaaatgtcagcctgtcagcctttgcctcttctccagccttaagcagagatataacgaagtcgagatcggctctggatgcatcttcagcaccgaattcaacgatcagacctctctccttcgcatactcaacggcctcaacgctcttctcgatcacgaactccttaccctttccagggaatttcgcgttgatgtggtattctgatgagggggcaaccatgaagatggaatcagcatttgcttcagcagccgcatcaatgtcttccttccttatccttgcaaagctgcagatctccgccttaagccccctctcggctatcgccttaatcgctttgaaatcaccttcagaggcaacagcagttccagcttcgattatgtcaacaccaagagcatccaaagcttcggctatcatcaacttctgctctaccgtgagcgaaactcctggagtttgctctccatctctcagcgtggtgtcgagaacgagaactttcacgtacacccttctgagtcgtgattaaaaaggttatccaaattcacgagatgtttatttttgaacgacagattcaaaggcccttcttatcttctcgtagctttcctcaagcgcttcggggattatctgcacgtccgcaagcacaggcatgaatgaagtgtccccgctccatctcggaacgacgtggaagtgcatgtgctcggcaattcccgctccagcgacctttccaaggtttacgcccaggttaaagccatcaggattcatcgaaagccttatcgccttcattgagagatttacgagcttcatgatctcgagaagctcatcatcgcttagatcctcagtggatggcacgtgcctcttgggacacaccatcacatgtccgggattatatggatggcggttcatgatcacgaagctcctttctgccttgtaaaccacaagcatttccctttcgtctccccttactgcggaacagaaaacacaatcctttggtttcgttgccaagacgtatcttatcctccagggagcgaagatcctttccatgaaaggaatgagttatgaattttaaaaactttccaaaattttttcgagttttgcgaactccgagagcagatgactctttatcctcgcaactacaaccttcggatcctcggccccgtagacgtagccgatccaacctttttcgacaagccttctccttattatcccccttctgtgtagctccctcagcctttctctaacgaacctcgttgataggttgagctcctttgctatctccctcacgctgagctccttcctttcaagcagcagggtgtagattctgtggtcggaaggcttaaggtccagcccctccagtatttttattacctccctgagcattttctaccccctacctttgaactcgaaagatcataaggtatataaacattacgaaacggtaattaccatggacaaactcgagcttttcacgggcttcgtcgtcaatcccttaacaagaaaggcactgaagggcatttccagctactgcgatcattgcggaaagaacaggatcgaggttgctctcgaacttttcgtcggagcaaggaatgaagcctgttggaagtgcaggcttgcggaaaagattgtaaggccagtccttctcaggggggctgaagcctacaatgttggtgaagaggagctgaaggaaaagttcagggatccccactggagaaaagggctggcaagcgtacttaaaggaatagccgagtttggagtaagaaagcccttcgtacccggggcccccttccaggttgtgtgggatataacctacgcgtgcaatttgaaatgcaggcactgctatgctacggcaggaaaagccctcgaggacgagctgacgaccgaggaagccttagacacaatagataaactcgcaaagctcggggttaccataatagccttttcaggcggtgagcctttggttagaaaggacatattccagctaacaagctacgctgcagataaagggatctacgttgccattgcaacgaacggaacgcttataagtgaagaaatggcgaagaggatgaaggagaacggcgtgagatatctgcagatcagcttggacgggcttagagatacgcacgactctttcaggggtataaggggctgctttgacagaacggttgaaggtataaagaatgcggtgaagcatggattcttcgtcaacgtatcgatgacggtgacgaagctgaactacgatgaagttcccgcagtcatcgagctatgcgagaagctcggagttgactggttcatgcactacaacttcatcccaacgggcagaggaagggaaattgtcgacctagacattctaccagaccagagagagaacttgctcagaatgctgtacgagaggaatggaaattcgaagttgagcttgctatcaacagctccgcagttcgcgaggtttgccttgcagtgcgacggcgggatgataccgacgcatttctacaatttaaacgcaggcgagcggctcagacagcttgccgagttcatcgggggctgcggtgccggaaggttttacttcgcaatcaaggcgaacggagacattcagccctgcgtattcttcccgcttgtcgtaggcaacgtgagggtcgacgacctcgaggatctttggaagaaaaacgaggttttcgaagctctgagggaaaaggacagacttgagggttgcggagaatgtagctaccgctacgtttgcggcggttgcagggcaagggctttcaactacttcggtgactatctgaagccagatccgggctgtataaggaacaaggccctctggaaaactctcgtcatatgatcacactggtaaacccgaatgcaaatgtggaagtcgtaagcaggcttgaaatctcgactcctcctcttggtatcggctatcttgcatccgttttgcgagagaaggggtttaaggtaagaattatcgacgatctcgtggagaagctcggatttgaggaacttctacggaggatcagggattctgtaatcgttggaattacctcgacaactccaaccttcaactccgctctgaactatgcgagaaaaataaaaaatgctttgaaagacgtcttcgtcatcctcggcggtgttcacgtttcttttgagcccgaaaaagctcttatgaatgacttcgtggacgcagtttgcataggggaaggggaggagacgatagtagaggtcgctgagagggttgaagcaggaaagagccttgaaggcgttaagggggtttactacagggaaagcgggaagatcaggaagaacgatcgtaggggattcatcgacgaccttgactccattcccttcccagcttatgatatgatgcccttagagaagtacacggttctgggcgacaagctcaagcagttccccgtgataagctctcgtggctgccctttcgcgtgccgttactgcagctcatcgctgtttatgggtcacagatacagggcgaggagcgcagaaaatgttgtcgatgaaatcgagtggctctgcgaagaatttggcgcgaggcacatcgcctttagcgacgacactttcacgctcggtaaaaagagggttctggagatctgcaacgagatcaagaggaggggtctggaggtcacttggagctgctcctcccgcattgacacgatcacggcggaaatgctgaggattatgaagtctgcgggctgtgtagcgatctactacggcatcgagtctgcgagcaagaggatccttgaatactacaagaagaagatctcgcttgaaaaagccaaggaagtcgtgaaagcgactaaaagggcaggaatagctgcgatatgctccttcatcgttggagcacccatggagacaaagagggaaatgaacgagacactcaggtttgcactgagactcgatcccgattacgcgcagttctcaatactgacaccctatcccggtactgagatctacagggaggcaaaagagaggaatttgctgcttaccgaaaactttgaggaatacactgcaggaaagcccgtgcttaaaacgcttgtatcccccaaagagcttgccaaattcttgagaaaatgctacatgcggttttacatccgtccgggcttcatactcagggagctcaggaagggcaatgcccgcctgattctcagggttctgaagaagtctctcgttcaatcttaaaaaattttgaattgtttatagagccttcagaatacactctttcagcaaacctgctacgctgattttcgagaaaattcgctctaccgtgtctgttgccgtgatctccgaaattcctgaagagaacagcttgatggctgcaaaatctgcaagaaccgcatgaatgcaggccgtttcaaccctttttgcaccaagttcgtaaagcctcttggcagcctccacaaccgttccgcctgtggaaattatatcgtccactatcacgactgtttttcccagaacatcgagtttcttcggggcaatttcgacggtctgggcatctattcttctcttttctaaccagtcccattcgcagttcgcaaaccttgccgcggtcttcactctttcagctgatcccttgtcgggagaaaccattaccacttctctttccctgaaatgttccccgataaggggcatggcatccaagtccataagctttctgaagtattcctttgccttgttgctgtgtatgttcacggtaagaaccctttctgccctttcctcgatcatctttgcaacggcccttatgctcacggcttctcctttcaaaaattcacgatcctgcctcgcgtagcccatgtatggaattacggccgttatctctcccttcagtgcgtcgaaaagaaggttcagaagaactatgtcctctgaggagcacagacttccaaccagtatgtgggagttttcttcgcttagaactctgacatagagctccccgtcgggaaatttcctgtactggacctctgcaatctttactccgaggctttcagcaactctctttccaagcatcggagatgaagggcagagcacgagatccataaaaaattgaggggtcacttggttttataaattctcagaagctccacgggaatcgggagaactatcgtggtattcttttccgccgcaacatcggttagcgtctgcaggtatctgaggaataaagcactctcgctctgcgcgagaacgtctgccgcctctttcaacctcattgaagcctgatattctccttccgcccttatgatcttcgaccttctctccctctctgcctgggcctgcatcgccataactctgcgcatttcttcaggcagttccacatccttgatctcgaccgctgtaacctttatgccccatggattcgtcgcctcgtcgatgatctgctggagtcttacgttcagcttttccctctccgagagcacctcatcgagttcagcctggcctataatgctcctgagcgttgtctgagcaatctgggcggttgcatacatgtagttgaatacctctgttaccgcctttgccggatcaacaacacggtagtacacaactgc

>Bin11_26

agttacgcttgcagtctattgcaacaacgatgcactgagttccgaagatctgcgcagctctgcgaacgaattctggatccctaacagcggcggtgtttattgaaaccttatctgctcctgcagagagtatcgcatttatgtcctcgatacttccgatccctccgcccacggtgagcgggatgaagacattctcggcagttctttcaacaacctccaccatcgtcttccttccttcgggcgaggcggtgatgtcgaggaagactatctcatcagccccttcagaatcgtatctcttcgcgagctcaactggatcgcccgcatctctgagatttacgaattccactcctttaaccacggtggcctttccgtttctgtaggtaacatctaagcatgggattatcctctttgcaagcattacctcctcacctcaaacctttcgaattccttaatctcggcattcctgatctcgacatctctaaccacggcgagtggcggaccctggcgaacataggagatgaacttctccaaggaattctcctcgccaaccgcataaatgtaaacccttccgtcgggcaaatttttcacgaaaccttttattccgagctccttggctatcctcgtcgtgtaatacctgaacccaactccctgaacaacaccgctcacgtagatctcaagggccttcattatttcaaaatccgcagtgggtttaaagttttaattcctcaggtcttcgaattcaagctccctctttatttcctcgtagaaccttttcagaaattcgtgccttctctcagccaatttttttgctgtatctgtgtaaagcaaatctttcagctttagaagtttctcctcgaagtgcttaactgtttcttcaatgcttctgcccttctctcccgccacaataaaggctcttgcaacacccacagcccccatggcgtcaagtttgtcagcatcgctcagaacccttgcctcaagcgtcctcggttccattttcccggaaaaggaatgagcctcgattgcgtgtgcaacagattctatgaattcctctccgtagccttcttctctcagaacttttcttgcaaagtctgcagcctttaaagcgtgtttttcctcattcctgaaggaatcgtgaagcaacgctgcctttgagacaatctcgatatcagctccttccttcaaggctatgaaccttgcaatctctaaaaccctgagcacgtgatcatcgccgtgggccacgaaaattctaaacatggattttaaaatttttaagcaccaaactgcctgaatgcctttgctacgtgaagatgaaaattgagaatctattaaattacctcaaacccgagatcaaatcaaattcttctccacggcctttgaaggatctgcaaaaaccaagccatcgacgacttttgcttcgtatttttgcttaaaaacctcatcgagcccatcagcaaagcccttgcccgttctcgccctgcaaacgtatgcgcccaaataagctctgttcgctccagcaaaaagcattaagggtaaagactgtagcgagactgagccagctgcaaaaatatccttttccggaagaagaatccttgccagtgcaacgaattttgcaactgctatgggggaagctggactcgagctctctaaagg

>Bin11_27

acgttctgtggtctacggaaaccatgaggaactacggggtcatgcaggctgcagtgctttacacaatgggcgtcaccgtagactgggactctgcgggaagggttaaagatgtaaagcttatgaatctcagcgatctgaagattaagctttcagacgggacggaaatagcaaggcctaggatcgatgtcgtcgttgttagctccgggcttcacagggatacgttctcagcactgatgcagctgctcgataaagctgtaaaactttgcataatggctgatgaggatgaaagccagaattacgtcaggaagcactacttggaaatcaaagaggccctgatcgagaaaggttatagtgaaaccgaagcaagtctgcttgcgggcataaggattttctcagaaagccccggaaactacggaaccggattgtcagatgcgatttcagcgagcaatacttggcagagcgatgcaaagcttgcggaacttttcataaatagggttggctatgcctacggtttgagcctgtggggtcttgaagctcatgaggtctttgggatgaatcttaaagcaactgacattgccgtgcactccagatcgacaaacctctatgctgcccttgacaacgacgatgtcttccagtaccttggaggaatagctctggccgtaagatacctcacgggccaaaatcccgaaacatatatcttcgacattaaagaggcgagcaatccaaaggttagaacactgggcgagttcatagcaacagagattctttcgaggtacttcaatccgaagtggatagagggaatgatggagaacgactacgctggagcgagggagatggcaaaggttatcgagaacctgtggggatgggaggtcgtgcttccagaactgatctcggacagaacttggcagaacttctacgaaatctacgtgaaagataaatatggactcgggcttagagagttcttcgagaagagcaatccttacgcactgcagtcaatcgtcgcaaggatgctcgaagcgataaggaaagatcactgggatgctcctgaagaagtaaaaatcgagcttgcaaaagaactgcagaggcttcaaaaggaatacggattcacctgctgccaccacacttgcgggaaccttgatctgctcaactatgccgccggaattcttagctcgatcgaacagagagaaagcaaagagggcgaaggcggtttaccaagagaagatggcggtaaaaaggcaccgatagtcaagtcgggggagagtggcaaagtgattcagacacaaaaaacggcaggagaggaaacgaatcagagcgttggatttggatttgagcaaaccgctgtaccttctcatacgggagctgaagaagtttctggctacaggatggaaatttccgagcaacaacagtcaaagatagaggtctccacgactcctttggttgccctgctgatcgttatacttgctgttctggtcttctatgctgggatgaggatgcgaaaatgaactaatatttttattttttgggtcaatggaaagccttttacgtcatctcacaactgtccattatggatacggagttcataatccgaacattgttcaaagcacatgaaagcggaaactacgtgatcggcccgagttaccttgcagaggagctcgaaataccgaagtccacggcccaaaaaattctatttagattatcagagcagggctacgggttctacgtcgaaaaaaagggattcatattctctgagctcggtttaaaggagggtaaaaggatattgagaaggcacagacttttagaatgccttcttgaagatctggggatggataaatcggaaatctgcgaggaggcttcaaaaatagactttgcccttggagttggccttgaaaggattattgaggagagatacggaaaaagggagcgatgtccatgcggaaagcctataccgttattctgattctcttgatttcagccgcagacgcaaggatacttgtttcaataccggagctggagggcatagcagagagaatctcgggcgaagacgccgagagccttataggttctcaggttgatccgcactacgcttcaatcagctatcaggagctgaagaagattgaggatgcggaggttgttctgcttgcaaactccaagctaatcgagtttgaatcgaaaatcaggcaagtctgtggcgagaagtgtctggatttcgaggattacaacgccaccctgcaggaatttccgggaattggagagaatccgcacgcatactggcttttgcctaagaatgccctgaacatcgccttggcccttaagaacaggctcgccgaaatacaccctgaaagggccggagagttcgagaaaaactacaggaacttcaaaaaggcgatcgagcttgctgaaaaggatgccagaaagatcgtatctgatgttaaggattacagctttgttgccatggatccgcatgcagcgtacgcgatttctgcacttggccttaaggtctcgctcgccttccctgaggaagtcaccccgagcgccgaggagctcaagggaatagatcggttaaggaattgcgttctggtcatagcggagtaccaggaagggaccaaggttgaggagatagccaaacaaatcgccaaagagagcaaatgcggaattgcgaaggtgaaggtcgtttcaaacctcaattttgagactatgctgatcgcaaacgccgtaagtctcgcaaatcccctcttctcagaatctgagaacgaatggcttctttacttgctgtcccttgtggttttaggcgagtccgtggctttagtggttctatggcagtcaaagcgaagaatttgagcttttcctacggaaagcgggttgcactccaaaatcttagcttcgagatagtcgaagacgaattcgttgcgattctcggtcccaacggggcgggaaagacgacattgctgaaatgc

>Bin11_28

gcttccttggccttatcgatgaattcctgctttatatctctcacagcaacctcgtagccagccgttgcgcacacctgcgcgattccgtgccccataactcctgcttcgagaacggccaattttttcatagcaccaccaattggatcttcatagcggattaaataatctttattaaataatctttatgattttgctcataatcagctcctttggaatcacttgatttaaatataatagatgacccatgtccgaccatgagcagagtgttgtttgaagtcgattcggatcccagaatagcctggataaaactcaacgaaccgaaaatgaacctactgaatcttaaattgctaaaagaatttctggatgcaattataatggctgaaaaaggtgaaaattcagcaataattctctccagcacaggtgagaacttttgcgctggggcagacttgaaagaactgaaatccctttcttttgaagatggtataaggtggtttgagagctacatggatgttgtgagaatgcttagaaattcaagcaagccagttattgccgcggttcggggcttctgtgtcgctggtggtaatgaaattgcgatggcctgcgatattgtagtggcctctaaaaatgcgaaatttgggcagcctgaagcaaaggttggatcgaccgcaatgggcttgggagttcagcttttgcctgttctcgtgggagagaaaagagcaagagaactgcttcttacgggcagaattatagatgcggaagaagcttttcgcattgggctgataaacagagttgtggatgatgaaaagctcgaagagactgcaagagcacttgcgattgaaattgtagaaaacaagcgagttcagggaaagatgcgaaagtttccttcaaaaagaggagttcacgcgtagaaagttcagcgggattaaataggattggaaatttggagcatcgggaatatctttacttttttgttaaattgcttgggagataaaaagcaaaagctttttagagttaaaattcacggagtctatgaaagtcgaggacataaaggtggtcggcataatcggtgctggtgttatgggaaacggaattgctcaggtttttgccagaaacggctacgaggtcatccttgttgacataagcgaggaaatacttgagaaggcgctcgagaatattaaatctggaccatatggtctgaaaaaactcgttgagaagggaaaaatgagcgaggaagagatggagaactgcatgagaagaataaagacctcaacaggctatgaaagtctgaaaaacgtcgatttcataatagaagcagtgccggagaacttggagctgaagagaaagatctttgcagaactggatcgaatctgcaagaaagacaccattttcgcatcgaacacgtctggaatcatgatctccagcatagcgaccgtagttgaaagaaaggacaggttcatagggatgcattggtttaatcccgccccggtgatgaggctgatcgaggttgtgaagggcgccatgacttccgaggaaacattccagatcacgatggagctctcgaaacgcttgggcaaggttccgattcctgcaaacgatgctccgggtttcttcacaaccagattcatcaactcctggctgattgaagcgatcaggctttacgaagcaaacgtcgctggaataaaggagatcgacgagatgtgcaaactcgcttttggcttcccaatgggcccgttcgagctcatggatctcatcgggctggacacagttctgcacattgcggattacatgtatgaggagaccaaggagaagcactatgctccacctctgacgcttaaaaagctcgtgctgtcaggctatttgggagacaaaagactgaagttcggaagcaagggtggatggtacgacttcttccagattaagaggtgatctggcttgaggaaggtagcggtgcttggtgttggaatgacgaaatttggaaaacattctgataaatcccttgccgatctgttctccgaggcctttttcgaggcatttgaggagtcaaacatagagctaaaggacatccaggctgtatattacggaaatttcgttggagagattactgacggttctggaaatctttctggatttattgccgacgagatagggctgaaaaacgttcccgcaataagatacgaaggtgcctgcgcctcatcgagcgtcgctttcagagaagccgtgagggctgtttcagctggctactacgactgcgttgtcgttgggggaagcgaaaaactgctaaccgctggcactggcattggcacgagagcacttgcaacggctgtcgatgccatttatgagatttctgctggccttacctttcccggcgtttttgccttggctgcaaggctttatgcaaaggaatacggaattcccctcgagaagcttcgtgagatgatggcttacgtttcaataaaaaatcacagatatggcgcagtcaacccgaaatcccagttttacggtaagtatgggaatttgaaggtagaggacgtgctgaactcaagaatgatctgctctccgatcacgctactcgattcctgcccgatgacagacggtggcagtgcggcaataattgcagaggagaagcttgccaaggatgctgttgacacccccgtatatgttttgggcacgggacaagcttccggtggagcccttttcaggcagggaaaagatatagttaaggccataccgagaaaaagagcagctgaaatggcatttaaggaggctaaaattcaacccaaggacgtggattttgtggagcttcacgattgcttcacaattgccgagataatcgctcttgaggcgatgggattcttcaagtacggagaagcttgctatgcgacaaaggaaggcataacagccatcgatggcgagcttccagtaaaccccgatggtggtcttatcggcaaagggcatcccgttggagctacaggtgtttctcagatttattctgcggtcaaacagctcaggggggagttcaaatggaatcaggttgaggatgcaaggattgcaatgactgacacgcttggcggagacttcgggacgcttgtaaatgtgatcctcggggtggaatgatgtttgcagagtttttagaaaatctgaaggaaggaaggctgatcggtttgaaatgcagggattgcggagcaacgacatgtccaccaaagaacagctgcaacgaatgcggcagcaggaaactcgaaaggattttgctcagcggaaaaggggtaattagaagctacacggtcacatacatagcaccgataggctatgaaaaggaggcaccatacactgtagccctcgtagagctcgaagaggggccatggatggttggaaggcttgatctggatccagataaggctgagaaggaagatctaataggaaaaagggtctcagtttttgcaaaggaaatgccgagcgaaatgttctatcccgacaagagcaaaaggatcgtgccctatttcaggatcgaggattaggatgagcttcgagcttaatgaagaattgaaagacttgcagagagctgtcagggaatttgctgaaaaggaaataaagccatttggcagagaatttgatgagaaagaagagtatccctgggagatatacagaaaggctgcaaaacttggctacgtcggtgcaagcgttcccgaagaatacggcggagcgggaatggggtgtcttgccgaagccgtgataagcattgaactaacgagagctgacagcagcgttggttccgcagtggacatcgctgttctcggagttccaatggtcctaaagcacggcacggaagagcagaaagaaaagtatattcccggggtcgtgaagggaaaagctccatccgcgattgcgataaccgagcctgattgtggaaccgacgtttctgcgataagaacgaaggcggttaaggttgaagatggctgggttataaacggggcaaagactttcataaccaacggaagcatcgcgtcatacacgatcgtgcttgcaaaaacagcagaagtaaatccgccccatgcgggaataagcgctttcatagtcgaaacctcatccgatggttatgaggtgaggaggataaagaagatggggctcaactgccatgacacctgcgaagttcttctcaaaaacgtaagaatctccagagagaatttggtgggaaaagaaaacagaggattctaccagctgatggatttcttcaatgagagcaggataaagatagccgcattgcatctgggaatggccataggagcctatgagagggcccttgagtatgcaaagcagaggaaagcctttggaaaaccactgatagagcatcaggcgatagcattcaagcttgcggacatgttcacgatgatcgaagctgcaaagcttttagtttacagagcggcatcgctcgtggacagcggatctgcaaatcctgccttaagctcggctgcaaagctttttgcaagtgaagttgcagtcaaggtatgcggagaggctgtgcagatattcggaggctacgggtacagcaaggagtacgatgttgagcgctactacagggatgcgagagtcgggacaatatacgagggcacgagcgaggcgcagaggattgtgatctcgaggtttctggcagggaaactgaggtagccaaattttaattaggtgcttggatatgctcaacttcaaagttctcgaactcgcaagcttctatcctggcccctactgtgcaagaatacttcagcttctgggtgcggaagtgataaagattgagcctcccgcaggggatcctgcaagagccctttcagaggtctttggagcgctcaatacaggaaaaaaattcctgacacttgatttgaaggatgaaggagatagagaacgattttacgaattggccaaggatgcagatgtgatcgttgaaggctataggcccgggattgcgagaaagcttggaatagattatgaatcggtttccaaaattaatccgaggatagtatactgctctatttctgcttttggtcagaaatcaaggctatcgcagtatccagcgcacgatctcaacgttttaggacttttgggtattttagaaattagctgtaagggaaaaatgctggatccaaacctacaacttgcagatttctcttccgctgtatttgcagcaatagcgattctatctgcgctgatagaaagggaaaaaaccggaaatgggaagtttatcgatataagcatgctgaggtcggcatttttctcggttccaatccacacaacttcgattttcaacggacttggaattctccccgtttttgcgagaaatcctgcatacgggatttacaggacttcagacggttttataacccttggaattattgcggaggagcatttctggcagagattatgcaaggtgcttggttttgaatttgagatatccatgctcgaggtcttcgatcgatatgaggagcttagagatctaataggtgcgaagatcgagaaaatgagcacgaaagaggcgattgaacttcttcaatccgcagacgtgccggcttttgaggttttaagccttgaaaagccagagaagatcgaggaattgcttggagagagcataatcgaggaaatagagtttgaaggcgagaaaataaaggtggccaaaaatccattcggggtttgatcatgggtatagtggttggtggtggaaagtttgggctcaaagcagcagaattcctgctgaggggcgcagagatttcattcttctcgacagcgatcccgattgtgaggtggctaaaaaaacttggaatcaaaataaaagaagctaaggcagaaaagctcgttgaaatagccgaaaaattgaatcccgaatggatattcccaactgctcctgttcatttggttgcagaagccattaaagattactttgaaccttggaacgaggttatcaactgcattctttcgggaatcccacccaaacttgtggtctcgcaaccgcctgcagctgccatggtgtgatcacggctttacggaaaagggggctgaaggaagatctaaatagccagtcctaaataatcaaaagttagcgaaattttattatattctatcccgaaatcgaagccatggtaaagattgtcgatctcacacttagagatggacaccaatcgctgcttgcaacgagaatgcggacgagggacatgcttcccatactagaaaagttcgactctgcaggaatttactctttcgaagtctggggaggtgcaacttttgatgtatgccaccgatttctgaatgagaatccctgggagaggcttagggagattaggaaaagggtgaagaacacatttttacaaatgctgctccgagggcaaaacctcgtgggttatcgcaattatccagatgatttggttgaaaagttcgtgaaaaagaccatagagaatggtttggacatttttaggatctttgatgccttaaacgatgtaagaaaccttgtggctccaataaagtttgcaaagaagtatggagcgaatcacgttcagggtacgatatgctacacgatctcgcccgtgcacacgatcgagaattatgtggaaatagcaaggcagcttgcagagcttgaggtcgactcgatcgcgatcaaggacatggcaggaatgctttcacctaaggttacttacgagcttgtaagagctttgaagaaggagattgacctacctatcaacgttcattcccactacacaagcggaatggcttcgatgagccttcttaaagctgttgaagccggggcagacatcatcgacacctgcatgtccccgcttgcatgcggaacatctcatcctccaacggaaagcatcgtatttgcgctcgaggagctcggctacaaaactggagttaaattggacgtacttatggaggtcagggattacttcatgaagatcagggaaaagtattcgggctacatcgatcccctttcaacgatacccgacaccaatgtgctcgtctaccagattccaggtggcatgttctcgaacatgatctcccagctgaaggagcagaatgctctcgaaaaactgccagaagtgctgagagaagttccgagggtaagagaagaccttggatatccacctcttgtcaccccgacaagccagatcgttggagtccaagcggtgctgaacgtgcttatgggagaaaggtacaaagtagtgacgaaggagacaaaagatctcgtaaaggggatgtatggaagaacgcctgcaccagtaaagccagaaatagtgaagaaaatcctaggggacgagaagccgattgactgcagaccagcagatctgcttgagccagagttcgagaagagaaaaagagaactcgaggagaggggaatcgaggccacagatgaaaacgtgctcatctatgctttattcccccagacaggacttcagttcctgatggggcaaacgaaagaagagcctttcccctcaaccacgaagaaagttgaggggagcttcgaagttgttgtggatggagtaaagtattccgtgagcgtgaagccgaggtaattttttattttcttcaaacgattttcgggagaaacttgaaatattttgagttcaagcaaaatcatggaagaatttgcagaagcggaaatcatagcggagaagccaacgctcaggaatcccgttcttctgacaagctttcctggaatcggtcttgtcggcacgattgcgacgggttacttcataacagaactgcagcttgaagatttggggatcctggattcaagcgcgattccaccagtggcgacgctttacgagggagtggttcttcccccgataaggatttaccagtcgcaagaactaaactttcttctaatacattccgatgtgccaataattccgcagatagcctacgagatcagcagggtgatagttgattgggcaatggaggtcaatgtcagacgaattttctctcttgcaggtatagctacttttgaggacaaaaagagagtttttggagcagttacacacaaagaactgcttgcggaaatcaaggatcacgtggagatcctgaggacaggaacaatttcaggtgttgccggaagcatactgaacgaatgcatcgcggtgaagttccccggaatagctttgcttggcgaaacgtctggttttaatcctgatccgagggcttcagcaaatttgatcggagcgctgaacaggatactgggctggaatataaatgttcagaaattactacaagaagcggaattcattgaggcgcagatgcagaagcttgcggagcagactgtggaggaagtgaaaaaggaggaatttccgatgtacaggtgagagaatgaggtgctatgttctgagcggaatggcaaggggaatcatcgaggacatcatgagggggaacgtgagaacgatagagctcaggagcgctcacaacattgcaactgccctcagggtaaacttgggggattgcgttttcctgacgtattccaaaacgagcgacctcacaagaggggtgagcggtataattgcagaggtcgcgggaaaggaagtcgtgaggcagtccctcttctactcctcgccccactacatcgaggagagcgagatggctgtcgttaggcttaggctgaatccgagaggtgttggaaggataatacagataaagtctggcgaactgctcgaaccgattgaagctgaggtcgttgaggttactcacttcacagcttggtagcctgaagccttttctttaactcctgtccgaactggtgagcagcggagattggcagtggtagcttacccttcatgcaaatcctgactatctcaagagcagtttccggggaaacgaagctgccaggggaaatgtaaatctccctgcaatttttacatcctttcagggcatagcctattatcctttcaccgtccttgatcggcctgaactctccctcgccctctacttctccgaaaagccttttcttcgtgactcctatcgccggggtgttcgtcctgatcgcgatataagttgcaagaccgcatttcctcgggtgggcaattccgctgccgtccacgattatcacgctcttctccgatatcagctttgaaacggcattgaccgctggatccccctctctaaacatcagaaatcccggaatgtacggaaagtcaactttttcgatggaaaaattcctctcaacttccctaagttcgggaaattcgaatttaacagcgcatgaaattacactcttacctaagaatgtctggtcgaccccgataacgtatcttatttccgaaacactgtatctgtccctgaggtcaaccctctccgcaagctgtaattggagctctttaagtttctccaaattcatccgaggcacttccagaaccttgaatggacgctcttaacttcttctatgtttgccctcagcttcgccattgccccgctcttcctcctccatacgagttccgcgaggtaattctcgggctctcttatcgaggagatgtcgaatccagccattgcagagataattgccaggttcgtatacggcagcgccgcctccacgctgtatccgccttcaagagttgcgactattctgccgtcgcatatctcctcagcgattccaacgcaccatttaaacatctccgcatatccccttgcagtcagtgccagtcccgtgagctggtctgtgaaatgcgcatcaagccctgcggaaattgcaatgaactcgggctcgaactcgagcgtcagaggttctatcacttcttcgattgccatcatgtatccttcgtctccagtcccgggcggcatcgggatgttcacggtatatccaagcccctttcctgctccgcactcctgcggatagcctgtcccagggtagagcggaatctggtgggtggagatgaagagaactcggttgtcgttgtaaaaaacctcctgagtcccatctccgtggtgtgcgtcccagtcaaggatcaggattcttttcagccccctcctcagcaaccacttcaccattacggcgatgttgtttatgtagcaaaatccggccccgatgtgcggttttgcatgatgccctggaggtcgaaccattgcaaatgagttttcaacctcgccttcaatcacagcctctgcccctcttatcgctcctcctgctgcgagaagggctttttcgaagacccccacagggatgtttgtgtccatgtctattattcccccccttttgctctcctcccttagaaattcaatgtattctctcgtgtgcacttcgagaacgtcttctatgctcgcttctactggctgaaggagttttatctctggggagtcgaatattccttcttccttgagctggtctatcgtgtatgcaagcctctcttttctctcgggatgggttggactctgctcgtgctcgagatacgcaggatggaagacgatccctgttgccatcgaatgaagttcctgaaagaatattaaagttttactaaaatcataggagataaggcgggccaatttcgggatggaaagctttaaatttagctcgggcgatcatttcatctatggatgagtttgattttttggatgaagaggaagaggaagtcgtcgaaggagaggacggtgttaagcttgctgaaatttacaaactcacgatgaagctcctccggctgttggaggatttaaaaagcgttgaattaaaggaatccgcatctttaatgcttataagggagcttgttggtgaagacagggttcttttaggtcttgcatccaagatgctccaggacatcagcctcggctacgatggcgaggactacgtcatttaaaaaatttttttaaggcgccagcgttcagcgagagggctccgaagattatgtttaggtgatacgtggtgaatctccagatcgctaccaaggaacctaaaacactaaggtctacaaattttgagtagaggtaggccatgctagtctccgctattccgctgctacccggggttagcgggatcagggagacaacgacaatgatgagttgcgatgtgtatgagagcaacagatgaggatcctgcccgaatgagagaaggatgagcgaggggattagaaaactgctgctccacatcagcgcggttgttgtaaacagaaaaatgagctgcggtggagaagaaagaagcttcagggaacaatctctgaagttcttcatttcgttttcgagccctttggcaagcctttctctcttgaagattcttcttatgaaaccggtaaaggtctgaattctcccctcgtttttcaggatcgagtagaggaaatagagggagattaaaaaaaacacaagaaatatcaaagcaaccctgaagccgagatccgttgcgaagcccgtgaggatcaggaagattggaagggcgaggatgaagaatattgagtcgaggaccctttccgttataacgacaaatgtgctcttgcccaaagccagccccttgtcggagagcatctttattctcaaaggctctcctcccgcggaggagggagtgatcgcagccagaaacatgctgctgagagttatttcgagtgagcttttaaatggaagcctgagttgaaggctttttgatatctccttcagtctaaaggcccatagaagccagaaaaagacttgaaaaaggaccgcaagtgcaataaatgaaacttcgactcttttgagaagctcccaggttagactcgtttccgtgagtctgaagatcagaaccgtcgttatcaggcttatcgttaccccgaggacggcagaggcaaggcgtttcaacaaacccccctctttaaattcttccctgacttattaatcttatggcactctgcctgcaacgatcaccacgagcctgctccagaaactgtaagggccagtctcgacgaaatctatctccttcagccccgctttcctgagccattcaacgcattgctcgcttgaaggaaaaagcataattgattctgcgaattttcttataagcgcattatctggcttcctgggtgcaagaattacgaccttccccccactcttcgtcactctgaccatttcagatattcctttaagcggattgggccagtactcgatgcttcccgctgaaatcgctgagtcaaaggttttatccctaaaggggagattttccgcatcgccgagtataaagttggcgccaagcttctttttcgccttctttatctgctctaagctcagatcgacgccgaaaattctttcttcactaacctttttggctattcccagcgtagtaaaccctgttccgcagccaacctctaaaacaaggtcgccactctcgatttcggccatctcgacaactttctccctcatcgagctggagtagaagatcggattgatgtagtcgtaaaccttggaaaaatacctgtaaaatagctttgccctgttcttgttatcgagaagcgccatctactttattatctcctcgagcggaacctttccatgctttactaccttcatgttgatctgtagaatgtcctttgagataaccctccccctgacgatctttctctttcttagcccgggctcctttggcctgaaaccgactccacctgatagcaggattttctttctcgtggctccaggaatgtctggacgcattggaaagccgtccctgtctgagcctcccgtgatctgaagctcatagtcgggtggaagttctaagattgttccgctcacaacttcgcctatctgctttcctatcaatcgatttgcattcgctccgctaactgttttctgatacgctttgcccgtctttggatccgagacgacgaccttgaactccatgccattgcctttttcgaaatgtttataagcattgctactcggctgggcatatggtgtctcttgagaagatgctattcccgacggatttttctccatacagcatgattgtaactgagtttttggacgacctgaaaacggccggagtcgaagaggttggcattctttttgtcgtgaacactgcaaggctgagctcggtaagcggtggttttgaccttgcgagatacatagatcttgaagagaagagggcagaggaggagcttccaaatcttttggaaaagctggaaaaagcaggattgaaggcaaagttgataaagccgtttccttcggggaatccggttaccgagattctcaaggcctcaaagtcctacgacttcatagcaatgggagcaagggggaggggaatcttcaaggaaatcctgctgggtagtgtttccgagggggttgcgagaaggtcagaaattcccgtttacgtattcaagtcaaaaactgaagtcttggaagatggggtaaaatgctacaagccatgcgattatctattttcgagaattctcgttgcttttgacttttcgaagcactcgcagaaagcgcttgaatacgcgagcgaggttgcagcaaggactcgcggggagctgatactgctccacgtggaagaggggaagagtaggagagaggagcttgcagccttggcagaagacctgagggcgaggggattgaaagtggttgatattagcgccgcgggctctcctgtaaaggtgatactgagcaagcaggaggaattgaaggccacaacgatcttcttgggctccaggggaataagtcttgcgagaagcctcattggcggaagcgtctcggatccggttttgaggatgagcgaagtgccagttttcatctcaaaggggacctgaaaggttatgaggctcaaggggagggcttggaagtttggagacgacatctccacggatcacattacccccggaaggtattaccacctcaggagcaacatgccagagcttgcgaggcacataatggaggatgctgatccagatttcatgaagaagttcaggtccggggacttcatagttgcgggaaaaaactttggaatgggtagcagcagggagcacgctcccctagcgctgaagatcgccggcgtcagtgctgtgatagccaaatccttcgccagaattttctacagaaatgcgataaatgtcgggcttccgctcctcatcgctccgactgacggtatcagagacggggaggaaatagaagtcgacctctcaaagggaatcgtttacactgaaaagggcgagataagggctaaaagcctgccagagatcatgataagaattctcaacgaaggcgggctggtaaactacatcaaaaagcacggagacatcgaattgaagtgatccttactcctgagtccgcctatgcacagattgaggggccatcacctcatatgccttcattttttcaggggaagctacgactggggtttccgggaaaatctcagcaggttgctcgaggaggtctcggaagtggaagtggtggatggcattgacgatgtctgcagggtgtgtccatacaacaagggtttctgcagctacaccagcaacgctgaggaagaggtaagggagctcgatgagttcgcgctgaaaattcttggtctcgaagttggctcgagggttaagtgggaggagcttgaggagagggtcccagggataatcgaagaatggagggaatttgcatgtagaaactgcgactggcggggcgattgcaatgtttgagatcctcctcaaggtcatatggattctccttcccgcatacacgccaaacaacttcgcagtgcttttgggcggactaaagcccatggatttcggtaaaaacttcttcgacggcagaagaatcttgggagatgggaaaaccttttcaggatttttcggcggaattctcggtggaattctggttgcaaatgtccagagagttttagaagtgcattttggattcaggctactatcttcgctgccttactgcgaattcctttaccttgccctggccctttcgtttggttccatggttggcgacgctctcgggagttttttcaagcgcaggctcgggtttgaaaggggagcgagctttccatttgtggatcagctgacctttctcgtatttgcactcgcatttgcaagtctaacgaaagctttctgggtgctcttcgggatcttggaggtaattctcgccttcataatcacaccactgctgcatctcggtgttaattttctcgcgtacaagatgaagctgaaagatgtgccatggtgagctaacgcctcggaagtctgatctcgaaaactgctcccctcggaacgttgtcgtaaaccctaacacttccgcggaacatttccactatcttcttcacaaggaaaagtccgagtccaccgccttttctcgaaaatccagattcgaagatcctatccttcagctcatcagcgattccaacgccgttgtctgcaacccttacaatgcattcatctccaattgcggtaacgagcacctcgacctcgagagggcttacgcagctgtgctgtattgcgtttctaaccagattctctatagcagactttatcgcctcgtttgcctctacgtaaacgtcttcgagctttaaactgaatgaagcttcatctttgaacttttcaacgacctcctcagcgatttcagccacgttgaccagcttcagctctcccagtgcctcttcaagggcctttgtctcccttatcttctcgacagcgctgtcaagctttttcaaagccctctcgaggagcatttcatcctttgtttccagagcagccctcacaactgccaagtcgttcagtacgtcgtgccttaagatctttgagagcaaattgtagtattcgtttctcctcctcagctcctcgctcaacttcttgatttccgtgacatccaaggttgtagtgtaaactgcagactttcccttgtattctatcctcgtgctcctcccagcgacccatctaagttctccctttcgtacgatcctccaggtatacatctcggactccctgattccagcttccctctccatgtatctccttctcacaagctccctgtccgctgggtgaacgatctcgaatggatctgtcttcatgaactcctctacagaagaaaatccagctcccctcgccgcggccttgttggcaaagacgaattttccatcctgaactatgtaaattcccgcaaaagatctgtcga

>Bin11_29

catgttcctcggttactcgaggctcagactaaaggcagagaacatgcctgagcatgctgacgtcgttgaattcagcaggcttttgagcagaaggggttatgagatcgtggatgaatccgaacagagcagggtttgtttgctaagcctatgaggatcgaggattgcagagtgaggcttgaggaacttgaaagggctcgtgaggagcttttaaagctttcgagggagctgagaattcttgcttcgaaagccatcgtggcaattcacgaggacaaatttgagcttgctgacgagaacatcatgaaaggggcagaaataattgcaaggctcagggaattccagagatttccggagatcttctccataactctcgaaccaatgcaggagtttgcggaagcgatattttttgaaaaagccgtgaagggaaactttgatttttcattagacttcgagatctcgcactccgcatttatcactggccttgcagacgcaattggcgaactgagaaggtttgcgctttcgaaaatgatctcaaacgagctagaaaaagcagaggaaattctgggaataatggaaagaatttattctgagctcctttcatttgcttcattcccggacaagctcgtccccaacttaaggcaaaagctcgacgttgcgagagcgggaattgagagaacaaagtcggatttgctgactgcaaagctctatgcgagtctggatcggaatcgatgacactgacagcaggaatgggatgtgcacaacgtacctcgccctcattctgatcgaaaggcttaaagaggtcggccaactcttgggctttccaaggcttataaggcttaatccgacgattccattcaaaacgagagggaatggtgcggtcagtttccttgcagaagtagaggatattgacagggcagttgagatcgctgaggatgtcgtcgaaagctatgccgagctcgaagacccaaacactaatcctggagtagttttcgtcggcgaggaaaaagccaaattgctggaagaatttgcgaataaggcaataaaagatgttctcagaatagaggatgcactggaaattttagaaaggctgaaaatagagtatctgaagttcaaaaacggtagggggctgataggggctttggcctcggttggtgcacggcttgatgactttgtttacgagctgatcacatacagaatgcctgaaagaattggaaagccaagggaattcgaaaaagagagcttctacgacctcgatctgctcttctatcccgcgatcttcgataccgtggactggtgcaacgacgtcgttatggccgttccgggaacaccatgtccggttttgttcggactaaggggagaggacacagaagtgctgaaaaaggctctcgaattcgtgaagacggaaccatttgagaagtggcagatcttcctcacgaatcacgccaccgatatgcatatcgtcagcgatggagagatcaggaacttcaggtcctacagagtagtgggaaagctgaaagaaaggccttacgagatcaaaggtggccatgtgttcttcgaaattgagacaggatctaaggtgctgaagtgtgcagcatttgaaccaacgaagcagttcaggaacatagttagagcccttattcctgaggacacggttgaagtttacggttctgtgaaaaacgatacgcttaaccttgaaaaaatcagaatattggagctgaaaaagcagttcgcattcctcaacccaatctgcccgagctgcggaaagaggatggaatcgtctgggagaggacaggagtataggtgcaggaaatgcaagacaaaatttttcgagaaaataaaggttgaattgccgagaaaaattgagaaaggattttacgaagttcccccgtgtgccagaaggcacctgacaaagccattgataagaatgaatgtttcaggaaggcacattttcagatagaacgtcgagcatgctgtagattcctggtctgcttactcttgatatccactttaccgctataacggctccttttgcaaagcagtctctgctcgtagctctgtgcgttatctcgaccctttctccatctccaaagaataatacggtgtgctctccaacaacatcgccacctcgaagcccaaaaacgccgatctcttcccctctctttgcctccctgcagaacttcagctcaagctttttgcccttacttgccagaatctcccttactatctctgcggtctttatcgcagtcccactcggggcatctttcttcatcctgtggtgaatctcgaagatctcgacgtcgcagtcgtaaagaagcgaagaggcaaattcgacaattctgaagaatgcattaacgccaatgctgaagtttggtgaaataactgctggaaccctgttgcagatctcctcgagctccttcttctgagcttcattgaagccagtggtgcctattacgagcttcactcccttttgagccgctaccctcgcatttctgatgcacgcgctcggattcgtgaagtcaacaagaacgtcggcgtcgagcctttcaatctcattctcgatattaactcctatttttccaatcccggcaagttctccagcatcttttcctatttctacgatgtcaaaggcctgtgaaaccgtgaaaccttcgttaacggcatgctttaccacaagcctgcccattcttccagctgctccggcgattgcaagcctcatgacttcagtttttcactattttaaaaagttatcctgtggctacccaagtttctgtaacctttctctcgccccatcaatgaatgcgttcagataccttgcaacggccctcttcagatccattggatgcagagccccactcttgaagtcctctaaaagcgcttcaaaactttcgtactcgatatccccgccaaacttctgctccctctcgaccttcactttgccaaaggctggaaaaacgtagtgctttgcgaggtctattacgggatttccctcaacaaccttgggcgggcagtaggccttcatgatctttctctcaacctcctctggcgaatcgcttattgaaatgtaatttccctttgacttgctcatcttctcgccatcaagtcccgagattatcggcgtgtggatgcagattggagccttgtaaccgagtgcaggcagatactctcttgcgagcatgtgaatcttcctctggtccattccacctactgcgacatcgaccttaaggtaggctatgtctaaggactgcatcagggggtagatcatctgagagaccataggatcttcctttcttctgctgacttcgtccatgctcctcctcgccctgttcagcgttgtgatcctcgcaagcttcagcacatcaagcatgtaatccttgttcagctggtagctgctcccgaggacgaagcgagcattcctctcgtccaatccgaaggctatgaaagttttcatgttcttctccgcaagctttcttatctcctcgaaggttcccttttcgtttaaataagcgtgaatgtctgcgagaagaacaatgacttcgaatccaatgctttgcagatctttaagcttgttcacagttatcaggtgtcccagatgaatttcgccacttggctcgtagccaacgtaagccctcggcttatccttttcctgaagcaaaaccctgagctcttcagctgtcacgacttcctcagcatttctcgtcgcgatggcaatctttttttcaaggtccatggctaaaatttgcattctgtttttaaacttcttggagtcttttgaaaaaattttttctttttaaaaataaaaattctttgtatacgtattttggagcaaaaactttatatttatgcttgaagtatgtatcaaataagaaattagggtgaagcatatgaatgaagcttgtatccttcacagaaagtatggaggcaagattgagatactgccgaaatgcccagtgaggtcgctcagcgatttcagctactggtacactccaggcgttgctgagccgagcagggaaatagcaaaagaacccgagagagtctatgagtacacgaataagtggaacacagttgcgatcataaccgatggctcgagagtcctcggactcggcaacatcggcgctttagcatctcttccggtcatggaaggaaaatccatgatattcaagcttctcggcggtgtcgatgcttttccgcttccaataaacgaacaggatgcggataaattcatagaaattgtaaagaagctgacgccaggctttggaagcataaacctcgaggacatcgcagcacctaagtgtttctgcatcctcgatagattgcagaatgagcttgaaatcccggtctggcacgacgatcagctcggaaccgctactgcaactcttgccgctttgataaatgctctgaagcttgttggtaaatcgatgggtgaaatcgagatcgctgtaattggcgctggagctgcaaattttgccactgtcagattgctcgcaaaagctggagcagatctgaaaaaaatgttcgtcgttgacagcaggggaattttgaacatgcaaagggaggaccttgaaaagctgaagatatcgaatcccgagaagttctggctttgcagagaaacaaatgccgagcagaggagcgggggaattgcggaagctatgaggggcgtcgacgttgtgattgcagcaagcacaggtggagttatcaagaaggaatacgttaaacgaatggcagacgatccgatagtcttcgcactctcaaatccgatacctgaaatctatccacatgaagctaaggaagcgggagcaaggatcgttggaactggtaggagcgattttccgaatcagataaacaactc

>Bin11_30

actcttaagcaattccgccatcggaagtgcgatcagtctgaattcaagcttttcaagaatttgatttaagggaaaatcgccataagaaagctcaattctcttcttaagggctttgctcatcgaagggatctgcgaggctttcttttccagactgcttcgatcgcatatcagaattccattgttcttcaggttttgaaaatgcgtaaaaacgctctcagcatcaagggctatcaacgcatctaagggcatttgaagagcttttccgcctaccctgatgtgatagtagctgtgtgcgccgatgatgttgctgtgatactctctgcttcccagcacatcgtgccccttcatcgcaaaggccctgacggctatctgccctgcagattccactcctcctccttgaaagcctccgatcacaaagctcagatcaaccttcatagatctcttccctataatctctgtctacgaagacacctctgaaaaggcaatgcccgcgagaaagatgatcttcaagttcttcctgacttattaaaaccttgcagctttcacaccacagaaattcctcttcgaagcttaattccgaaaagaactcgcagtaatactcatggtggctgacatctcctttcctgcagcagtagcctatataggaatagtgaatgtggggtaaccagctgttgcacttggagcatcttttttccatgggatctctttcgcgttttccttaaaaagccttgtcatcactccgagaactgcgtgactgttcccttttttctcgctctttccgaggcccagattagaatcagaaacagaagaatcaggaataaaaagcttagagagtagccaagcaatgcgggattgctcgtgcttggagagaaaccgtagaagctcctgtaaaactccagaaaatgcgttagcggaattgcatatgctatttctcttacccctgagggtagaagggtaacagggtagtagattccgcagagaagcatgaagatcgcagtaagagtccaggcagcaacttctgctctataaccgaatataagaagtagcgagcaggtgagcgttccgatgatcatcgcactgagaaagatcccgaggagaaagagaagcagggaaggcggatcgattagcaggttcagatcaaacagatactttccagataacgcaaggatgagaaaggcaactaagcctctgaaggaaccgaaaagccaggcgccgaatatcatgtggaatattgaaactggagttacgaaggtgtaacgcaaactcttgctccagatgtcgaataaaaggacgtaggcaacgtcgagctgcgaaacctgaaggatgctcaaggttactgctccgatgagaataaaggccagatatctttcctcaaggctcaaataagctcccataagcccaacagatatcaggccgattatgggccagaagagggtctcaaagagtgtgaaaaagttcctcttcgttattatgtagtttctgtacgtgaatccgaggatcttgtagacctcacttcgcaagttcgttgaagacatcctcaagatcaacctcctttacggatatatttttgatccttccgaacctcgcaacctccttcgctatatcccatatcctggcttcagcagaatcaacgtagaaagagagctggtttccgtctctctcgaccgcaaaaacgccttcaatacttaaatttgggatatttgcatcgatttctatggtaatcttgtctctcatcttcagcattctcttgagctcttcttttgttccttctgccactatttttcccctgttgatgaaagcaattcgatcgcagagcatttccgcctctgacatgtagtgagtcgtaagaacaacggttattcccagttctttctgcaaggagcgaatcttttccctcagccttcttgcaactggcggatcaagacccttcgtcggctcatccaaaaaaacaagctctggatcgtttacaaaagcccttgcgaggatcaacttttgcttgtttcccgttgaaagcctgttgaactccacatcgcgaaattcctcgagctcgaactctcttatgagctcctccacagcttttttatcattgatcccgtaaaggagtgagtagtatcgcaagttctcgtagactgtaagactccagatcagattcggatttccgctcgcgatgtttattctttttcttatctccttgagctctctgagggtgtcatagccgagcacctcaatcctacctttcgtttgtctaagcagagtcgccatcagagagattagagttgtttttcctgcaccattaggaccgagaatgccgtaaattctgttactcggaattttcaagtaaattccgcttaaagccaccttttcggagaaaagggagcggtagaccatccagacgtcttcgcagactatggagtgcatagcaaaattttgaggaatggacatataaggattgggaactcgaggtcttccgtaggttctttcggtagtatgaaaaaattaatatattgagtcccaaaaatgtatgcatgggtatgagtggggaaatcactgcactttttgggatgcgggtttttacggatgaaggaagatacgttggcagagttgaggatgcgatcatcgacagcgacagcaacacgataaccgggctcgtgatcgtggactacaacaaggctctaatcgataagagttcgaggggagtcgtaattccatacaggctcgtaagggcagttggagatatcgttcttgtcagagacatcttcacgaagaagagaactccgagtcccgaattgagggagctcgtgctcggcgatgaagggtagggtattattaatcgttaactttttgaccctttaaagctgtttcttttcgaaaaaaatcacaacctttatattcatgaatttacaattttaaactcggtgatcctatgagcgaggcgaaggaagttattaaaaagatgtgtga

>Bin11_31

aacaagtcctctgagcttcatttctttaagcaccaatcccgccattatcgtctttccagctccgggatcatcggcaagcagaaatcttatccttggctgtttgagtatgtaaccgtaaaccgcctcgatctgaaatggcagagggtctattttagagacattcatcgcgagaagcggatcaaatagggatgcatatttgaagcgtagggcttcaatcgcgtaaaaaacgttttcagctttacccgaaagtttggcatgatctccaactacttcaagcctgttaatttccgcttccgttagcattctattgacaagagacttcgagctcagggtgtaaccaacgatgcgatagtagctgccctttctctccactttttcaatttttacaggctcaccgcagatccccctgacgatgtcaccttctcggagcataatcaaatgccttaaggttctataactaataaaatttttcaatttgtgcgagcggcgttttttctgtaaaataggctttcgattgatccttgctaaaatttcgcgttcattgccaaaattgcgcatttgaactcgatgcatctcttcgtatatgacgttattatttcaatatttattcgcaatttgcgaaaaagaaaaaatctttatttcccttcacggatccaaaagcatgaaactgcacgagtatcaggctaaacgcatattcgcggagcacgggataaagataccacgaggagctttagcggaaagacctgaagaggtcagaaagattgctgaggagcttggaggaaaagttgttctgaaagcccaggttctcgtgggtggaaggggaaaggcaggggggataaagagggctgatagcgttgattctgctgctgaaatcgctgagaagatgttcggatcatcgcttaaaggtgaaattgtcaaaaagatctacgtggaggagctgctcgaaataatggaggagtggtatctgagcctcgcaatagatagagtagaaaaatgcttctcgctcatattcagcaccactgggggaatggatattgaggaaatagcgcagaagttcccagaaaagatcgcaagggcaaaaatagatcccagatggggcctctgggattaccagataagagagattctgagcggggcaaagataaggacagagctctggaaagaaatgactgttatcgttaaggggctttacgagataatgatgaagtacgaggcggagcttgtggagataaatccccttgcattgacagaaaagggcctgatagcggcggatgcgaagatcatcattgacgataacgccttattcaggcacagagaactcgaagccctgcgagaatatgaaggcagcgacgaagtggagagaatggccataaatgcggggctgaactatgtgaggcttgacggaaacgttggggtaattgcaaacggcgctggaatggccatggccacaatggatctgatttaccttgagggtggaaaaccggcgaattttctcgacattgggggcggagctggggctgaaactgttaagagggcttttgaagtcctgtctgcagatcggaacgtgaaggtgatcttcatgaacatattcggcgggattacgaggtgcgacgaggttgcaaatggaatcttaaaggcctttcgggaaatcaacccaagaattccagttattttaaggctatctggaacaaatgaggaggagggtaggaaaataatagagaaagagctttcaggcaagattgaggtagtgaaaacaatggaagaaggggcaaagagggctgtggaggtggcaaaatggctgtaatagtggatcagaatacaagggccatagttcagggaatcacgggcagccagggcagatttcatgctgagaggatgctgaagtatggaacgaagatcgtcgccggcgttaccccgggtaggggaggagaggagatcttcggcgttccgattttcgacacggtaagggaagctaaaagaaaaacggatgcgaatgcaagcgtaatttttgtcccagcagcgtttgcggctgatgcgatcatggaagccatagacgcgggaataaagactgtggtatgcattaccgagggaattcccgttcacgacgagctcagggtttacctcagggttaaagaggaaaatgctatccttgtgggccccaactgcccgggcgtaatatccccgggaaaatcacatctcggaataatgcccgcgcacgtctttagagagggaaaagttggaattgtttcaagaagcggaacgctcacttatcagatctcgtacaatttcacatccctcggaatcggccagtccacagttatcggcataggaggggacagaatagtcggactcagcttttcagaagtcctggagatgtttgaaagggacagggagactgaagccatggtgctcatcggggaaatcggcggtagggatgaggagagagctgcggagtacataaggcagaatgtgagcaagccggtcgttgcgtacattgcaggaattactgcaccacccggaaagagaatgggccatgcgggcgcaataatcgagggcggagttggcacagcggaatcaaagatcagagccttaagggatgttggcgttgaggtggggaaaaccccgatggaggttaccgagatcgttgcaaggaagctgaaatgaggctcgatctgtggctcttgaaaaaagggttcttcaaaacgaggagcagggcaaagattgcgataaggatgggactggtaaaggtgaacggtcaaatcacgacaaagccttcggctgaaatcagcgaggatgcagtcatagaaattctcggtgacaagccagtgggctactggaagctgagggagctggacgagaagttccgtattttcagaggaggcgaaacggtgcttgacattgggagctctgctggaggatttctgctttacgcgagcgaaagggcaaaattcgtttatggaatagaggtcagcaatgagtttgaggatatcctgagggagatagagagggagagagggaatgtcagggttttcatcgaagacgcgttcagattcgatgtcgaaaggcttgaaagcgtcgatttgattctcaacgacctgacacttccattttcctcgtcgatgattgctctcaagagattcctgccaaagctcaagcctgacggaaggattctcttcgtccacaagctaagcagaaaggatgaaagggatgcgaattttgagggattcgagattcttggctttgagaaatcaagggataaaaaagaagcatactattttctcaggcctaagaccgtgcaggggtagcttgtgcctggaactgacctcggcgcgggcgattagtgaccgcgggctgaacggctcgccgaagcttgccgcttacaccccggtcctatcaacctgctcttttagcagagcccttaaggacggctcttttcgggggtggcttcaggcttagatgctttcagcctttagccactggcgcgtagctgcccggctatgccctgccggacaaccggtaaaccagtggcgccggaggctcgttcctct

>Bin11_32

agtgcggggggcgggattcgaacccgcggaccccttcgggacacggtcttaagccgtgcgccttttccactcggcaacccccgcacttggttacaaagacactcacgaggtaaagtagttaaattttgtgagtcctatgtctactctttcgtttcggctcaagactccagttgccctttcgatttcttttagtttatccattttttgtggaaaagcgaacaattttgtctcgctcaccgaaaatagtggattcagtttttggacggcctcaaaaagatatttatacaatcgagcaaaatttcatattggtgattggatgggcgttcacaggataaccagcgaatccgcaaggttctatgcaatgcgggaaaggatagtgggttccgcgatctcaatttttggagaagcgagtcttaaacttgaaagcctgagcagagagcagtgtgaaaagcttggagatttggcttcaaaactccttccctacgctccgggatatgcaggtaaagcgatgccgataattgcaagattgttctggaggctcgcaggggtgaaggagaaggagtttccactggttgaaatggagaaactcgaaaaggagatcgaggatctaagaaaagaacttgggatctaatcccagaattttcttgtcctcacgtaatttctttctgcttcaagaagggctttaaagaagtcttcttccgttggatagtaacctgaaagaaccctttttgcggtttcaaagccaacaccgtatgtgagcatggcataaactcctctcatgccgtagttcattacgaggttggctattttgaaaagctcgtccttctttggctcgtggctctttctgtcgcttaaaaccgctatcatactgctcccgcatttcaagcattttagactcagattgctcgcaatttcacggtagcttgaaccgcagttaacgcagtaaaccctgcagatttccacctttagcctctcagtaaaggctttcaaaattgctccgcttggttttgtgatcagtatgtcaggaatcttatcccttccttcaagacttatgggcgttaacctatcgtaaatggtgaatgagatctcccttccaaacttctcgaagaaaaacttagccctctccagatccatcttttctaagaatatctccctgagggcctccttgtaaacaggagtgccccttagcttcacaaccagcgttctcagatttattcttgccagctcttcatccttatcaaagagtccgaactttctcgcagctttcacaactttccactgcatcagccttgtatcaacaatagctctctcagcgaggctttcgatttcgcctgcgttaacgctcagaattgcatctcttacctctcgagggcttgcaggagatagctttatgcgataaggatcgatttcaactgatacattcgtccccctcttcagagaaaggagcagagcgagaatccttccgatagcctcgtttgccttgtggccgaagcagacattcaccaccgtatcactgccagaaccgtctatgactacgtgcacgtcggtgggaacgctaaactcctttttttgctcttctattttcctgataaccctaagggcgccatcttcatttacccactccttcaactgctccagaccaaccttgccttctgctatctcccctctcagctttccaaccagctgggcaacctcaaatggaactggtatctcctcaccgacccaggaaggaacttcaccttcggcaacaacaggctccactttaacgacgtcctcgacgctaaggatcctccataattcccccttcatagcaaaaacttccccgctgaaggtcgaaaggaagctttcgtccagagttccaattgttctcccggaagttatatcgacgactctgtagcttttctcgtccattatcatcgatatgttgtcgtaaaaatacttccttgtcctcctccgcgaggaaatctcgttgccatcgaagaaaaccctccagatatcgcttagatactggcatatatcacaaaattcctcaaaactcagattcctgtaaaagtaacatctcctgacgatttcgtaagcctttctggcttcgatccgcccatattcgagggcaatagcacaaatttggttcgcaaggacgtcgaggcttttttcgtgaagctccagatcttccaaaagacccagactggccctttttattatcgcgatggattctaggatgtcatcgaacgtgtttgcaactatgtaacccgtggatttcctgccgagtccgtgcccacttctaccaatcctctggatgagccttacagcttgtctcggactgctgtactgaatcactgaatcgacgtgccctatatcgatcccgagctccattgaagaagtgcagatcagagccttcagctttcctgaagcgaactcctcttcagccaagatccttgcttcccttgaaaggcttccgtggtgaacttccgcgcttatcagcttccttaggtgcacacccagaacttcggccgtttgccttgtgttcacgaagattagagtcgagcgatgtttctccacgagatccttgatgagagagagctcgtcttcggaacagagaattttgatctcgtactccttctcaaggtctcccgaaactatttcggctccaccgagtatttcagatacctttttcggatctccaacagtggcagaaagggctattgtctggaattttgcgatttcccgcaatctctcgattccaacgaagagttggacacccctctcgctgtccacgagttcgtgaatctcgtctattaccacgaactggacgtttctaagcgcattctttagtcttcttccgagaaagagcagctgaaaagtctcgggggtggtgatcagaatttcgggaggttttttgctctgcttaactctttcgctctccttcgtatctccgtgcctcacatcgatcgtcacgttcaggatctccgcgattttactcattctcctgaacatgtccctgttcagagccctgagaggagttatgtaaagaactctgatgccctgccttctttcctgaattagcttttgaaagattgggatcatcgccgcctctgtcttaccgctcccggtgggagctactatcagcgcattctttccagatatcactacctcaaaagctctttcctgaagatccgtaagcttctctattccaacggccttcaaagccctttcaagtctctcagccattgcgggcatcgctaccaaaaaggaaattttcgctaaggtttaaaaattctacaaccgcattataagcatggtaaaaattgacgaaattgatgtgaaaattcttaaagaactccaggatgacgcgaggaatagcctaaaggaaatcgcagaaagggttggcgttgctgagggcacggtttacaacaggataaacaaaatgaagagcatgggcatcataaagaagttcattccagtgctggattattctatgctcggctacgacatcacagctgttattggaatttcagctgagggtgggcatttagttgagatagagaaggagattgcaaaggaaaagaacgttactgccgtttacgacgtcacaggagaatacgacatcttagtcgtggccaaatttgagaacagagacaagctgaacgaattcgtgaagagactgcttgggatgaagagcgtcaaaaaaacttatacaatgctggtcctaaatgtggttaaagaggctcacatgatagatctttgatcaagtcgtgtattccgcgtttatttttacgtactcgtagccaagatcgcatccgatcgcgtaatcggtgcaatttcctctgtggaggtcgactataattctgaagtcccctcgcatgatctccctcgcctcctcctctctccccatgatctttccccgatccacgagaaacacggtcttttcatcgttctcaaaggcgatgctgattttctcatcaacttctgctccactgtagcccacagctgcaactattctcccccagtttggatcgcagccgaaaattgcagtttttacgagtagtgagttagcaacagcctttgcggcgagctctgccgacttttcactttccgcgcccctgactattacttcaaagacttttgttgccccctcaccgtccctagctatctgccttgcgatgctgtagataaccctctccagctccgactcgaaaacgcctctatctatcttatccctgcctgtagcaatcagaagcacagtgtcattcgtcgatgtatctccatccacagtaagcctgttcagcggctttacagcccttctaaggatttcataaagctccccgctttcgaatttcgcagaagtgaaaacgaagcaaagcatagttgccagattgggtgagatcatacctgcaccttttgcaacagccgaaatcttggcggattccgaaaaagccttctttatgaatctgtccgtggtccttattgcgttggcaaatttttccgcatgctctttgctgttgccaagaccactgtatacctccgccgccttccttcttatccagtcaacgtcgagtttccttccgataactcctgtcgatgcaaccgcaacttcgtcttccctacagccaaaaagctttgcagcgattctgcacatctctctcgcatccatcaaaccctgctctcccgtgaaagcatttgcatttccgctgttcacgatcaatccccttgcatagcccttgcgaatgttttcgctgcacacgattactggagcggccttaatcctgtttccggtaaaaacgccagcaatacttccgctgaactttacaagtcccaaaccaaactttccctccttaataccattacacagaatgccgtcgatatcggtgatctccatagcacaagttcgggcaggattaaaaaatccttttaaaacgaagcatcaacggactcctatgattgaacttcccacgctcgacgatatggatgaaaagcaaaaatccatacttctcaggctggacataaatgcgccgatcgtaaattcgaccattctcgacatcacgaggttcgaaagtcatcttccaacactccgcgagcttgaagacagggaaatcgtcattctggcgcatcagagcaggcctggaaggagggactttacgaccctggaggagcatgcaaaggttctaaggaaaatcgttggcagggatgttgaatacatcgacgaaatcttcagcaaaagagtcattgaaagaataaagaatatgaaatccggcgagatcgtgcttctggaaaacgtgaggttctactctgaagagcagcttgaaagaactgctgaggagcacgcaaactgtctgatggtgagaaaactgaaggatcacttcgatgccttcgttaacgatgcattttctgcatttcataggagccatgcatccctggttggctttattcccgttctgccaacctttattggccgagtggcggagaaggagataagtgcccttagcaggggattgaggaagggagataagatagcctttgtgcttggaggcgcaaagataaaggacccgataaaggtaatgaagaacgttctcgagaatggaattgctgagaaggtgttcctttcaggtgtcatcgcaaactacttcctgcttctgatcggcaaggaaatcggagaagaaaacaagaagatcgtggaggataacaaggagaacgttaaagacgaggacgccaagaagctcttggaaaagcacagggataagatcgttcttcccatcgatttcggtgtagacgtgggtggggtccgagaggacgtgtcgatcgacgagttcaatggaaagggcattataaaggacatcggggtcgagactatgaacctgttttcgaacatgatccctgagttcgacacggcagtcgtcaacggcacggctggagtttacgaggattcgaagttctctctcgggacttatgaagtgttaaaggctgtgtcgaagacaaagttctcgatcatcggcggaggacacagcgcttctgctgtgaacatgttcgggctcgctgacaaagtcagtcacgtttcaatcgcaggtggtgcctgcgttagatttctaagcggcgaaaaattgccagtgatcgaaaaaataagagaatactggggaaaggggaaaaaagggatagcgggaagctaaagctggtcaaaagtaatgacccttatggaaatcggaattttgaccacacttccaagcttagctccgattatgtagtttatttccttcttcgcggcaatgtctatgagcctctgggtgattattccgtcaaagactatcacagaagcccgcaggttgttcatcttcagggtcttcacaagatccctcaccgggacctccttgataacgttgaagttcctgtcaagaatccttgcaagcaattttccctcaaccatctccttgtgagttttcaggctttcgtgagaggataccttttcctccttcgctgactgctccctcttcagaacgtgaacctgctcgacaggcaccttgtttcttagagccttcaaaatttctttaagcgtcaggtcttccactcctttaccttccggcgctcttgctataaaatccacgtctgcaacctgtaaaagctccttaagtattagctctcctcccctgtcaccatccaagaaggcagttactgtcttctttttgctcagctccacgatcgtcttcggtatgttcgttccctcaacggcgatgacattcctgattccatgcttgagcagattcagaacgtctgctcttccttcaacgactatgatggcatcagattcgtccaccgctggaccggctggaagtttttcctctccgtactccaccacttcgtctatcctcagcgactctcttacgagatcggttatcttttcggattcgatttctggttcttcaaaaagttccctgagtatttccttagacctctccactattttttttctcttgcttgcccttacatcctcaatcctagaaaccttgatctttgccgagcaaggtccaaccctttcaatcgtttcaagcgcagcagcaattatcgcggtttcgactttgtccagactggatgggatctttatttctccgtagctccttcctcccttgctttcgatcttcacctcaatcctgcctattctgcccgtcttctgcaactctctgagatcaaggtctccaccaagaagtccctctgtctgaccgaagattgcccctacaacatcaggacgctccacaactccttcagctactatttcagcgtaaatgagatacttcgtggtatctgaagtcttcatcatataagcatcatcccctgtcatccaaatccctatttgatttccctttaaatagttttttagcccgacttcaaagactcttctacgaccttcacgacctcccttctcgtgaatggtttcttcagtatcatatcaacgccagcttccattgccttttcgcccttggagcttgcataggcagtaatagctatgatcttggcattgggatttattttctttatctccttaaccgcatctattccgctcatcactggcatcattatgtccataaggacgacgtcaggcctttctttcctgtagatttccaccgcctctctcccgtttgaggcctcgatgaccttataatccttcagcatgatcttgagaatctccctcatcgcggtctcatcatcaacgatcatcacgcttttcatcttaagctcttcctctcatgaatttaaaaatcttatcccagaaataaatcggacggagagcttatatagcagacctcacacaaagatcatggaagtcctgttgaacccggacaagtttttttcggagcgaaagaacatcggtttcaaatttcctgtacttatcgtcgctatttctgctataatagcagcattctcagcacatctgtccactgaaaccgtaatagaaatggcatcgaaggcactgagagaacaggggttatctcagtctcaaatagagatcttcaagtcgttcgtgtatgcttccatggtagtcggtgcctttatagctgttttcatcggctgggtcgttttaactgcgttgctttaccttttctccatgattttcagaagtaggggaaaatttacaaccctgatgaagttcgttgcattcagctacattccagcaatactcgtctgcccaattacgctatacctgtcctacgagtcgtttgtgatgcgaaacgtcagcgctctccctgcatcgactctttttggaatcgtgatatctctatggcaggctgtatactggacatttgcggtaaaaaatgcgagggagctaagtttaaggaattccgcgataacttcggcgatcgttctggcactctttatttcagccccgctctacagcttactgcaaccctctttacttgaaatgccataatggaaagcttaatatagtgccgagaagaaggggtaaaactttggaagggtggtttaatggtagatgaggtaattaaagaaaaggcagaggccttggctgaggcgttgatgaaccttcaggagtacagggatttcgttgagatggagaaaaacttgaaagcagatgtcgaagctcaggcaatgataatggaattccagaagaagcagcaggatttcgttacaaagcagatgtccggtgttttcgataacgatctcttgaatgagctcacagaactgcagtcgaagttgaacgcgagagagagcgttgttatgttcattgaatcttataacagacttctttcagcgattggcgaaatactggatttgataagcgaacgcctcgagctggatgttggagaagtctacagaaggtagacttcaattttttcatgtttcaggaattaattgcattttttctcggcttcctgtcgatcctttctccatgcgttttaccagtactaccaatagtctttgcaggctcaaggctcaggcttaaggactccttagcccttttttccggtttaatcctgtctatttctcttttcagccttacatccgtttttctggctggtttcagaattctgagcttcattctcatgttcttcttctctatttaccttataagcgatcgtcttgaactcgaattctcgaggaggatttcaaaattgagcggaattgcaaagatgaaactgccccccttcttcatcggcttccttctcccctttatctggcttccctgcattactcccttcttaggaattgctgtttcggacgcgatccttagcgagaatccgatcgtcgtgtcgatatgctacgttcttggctttgcaacggcagtcgctgtcgtactccttttcggcaacagcctgaaaatcagcttcgaaagggttcgaaaaatcctcggaatcaccgttctgatctcaaccctctatctggtctcgctttacctttgctaaaactttttaaactttcggggcaaacatggaacaaatgaggctctttgtggcagtagatatggacgaaaaaatcagggaaaatctttcacccttgctcgaccagctctcgaagctcaggggcttgaagacagttgagagagaaaatctgcacacaaccttgatgttcctcggagaagttcaggaagcaaagcttaaggatatccagatcgccctgtcgaaggttgaattcgaacccttcaaaataaccctgaagggaatgggcagatttccgaacaggggcgatcctagggttttgtgggttgcgattgaggagggaaaagatgagatgaccagacttgcggataacgtttactcaacgttgaagaagcttggtttcgatagggacaagtcattcgaggctcacgtgacagttgcgagggttaaaaacagaatcccggagctctatcgatttttggaggatttcaaaaatgccagttttggtgaaatgcttgtcaaagagttcaggctgaagcagagcatcttgaagccttcagggcccgtatacaaggatgtctatgttttcggtggcaaagatggatgaaattctgggggaagcactgaagcttgcagtgccagacgaagaagaggttcagaaagccagaattgctgaatttgaactgaggaaaaggctggagcctttcaatctcgattttattttcgtgggcagctacgcccgaaatacctggctcaagggaaatcttgaaatagatgtcttcgttctcttctcccccgagttgtcgaagaaagagctcgaggagagaataatccagctcggaaagcaggtctttgagaaatacgagctgaggtatgcggaacatccttacgttcacggagagctgctgggagtagaattcgacctcgttccatgctacaaggtcgaaagtgctggtaggataatatcagcagtagacaggaccccattccatcatgaatggcttagggagagggtgaggggaaaggaaaacgatatcagactgctcaagctttttctaaaagcaaacagtatttatggtgccgagtataaagtaagggggttttctggatatctctgtgagcttttgatagtcttctacggttctttcctcgaatgcctgaggaatgcgaggagttggacgagaaggactgttctggacccgaaaagaggagaagtgcggaaaggggagtctttcttcgtcgtcgacccggtggacgaaaaaaggaatgtggcggcaaacctgagcctggacaacctggcaaagttcgtgcatctgtcaaggcttttctttgaaagaccttcgctcgagtttttcacacggaaagagagaagagtctcggataacgcgataagggaggccatggatatcagggggaccgagatcttcgctttggagttcgaaaggcctgacatagtggaggacaacctttatccacagctcgagagggcgggaaggaagatatacgagatgctcaagagagaagggttcatgcccctccgcttcacttatttcgcttcagagcgatgctacattctctttgaatgccaggttaaagaactttcgaagataacgagaaagcttggcccagagttcgaggatcaagagaacactaagaacttcatttcaaagaataggccttttgagccgttcttggaggatggaaggtggtgggccttcgagttcaggaagcacacaaaaccagaagaggctgtagccgattttgcagcatcgaattatcaggctttggggaaaaacgtgggtttgaagatcagggaaggattcagaattttgaagggagaggaaatcctatctgcaccagttctggataagcttgcggactttttggggattagattatgagggtcacgtttctcggcacgggtgtttccgttccctgcggaaaaagggctcagagctcgatactgattgagagcgatttgaagatactgctggatgtcggccccggggcccttttgagactcgaagagattggagtcgatccggcggagatcaatgcagtttgcctcacgcacaaccacctggaccacaatggagacctcataaaccttctgaaggcaagatggctcatggaaggcggaagtctcgatatctatggtccaaaggggacgaggaatttcttagaaagtatgctgaacgcttatccgtatctgaggggaaagctgaaattcagagtaagcgaagaaaaaagcttcaggatcggggatctgggcgtgagcacgataacaacgattcattctatcgaaagtagagcctatgttttcgataacgccttggcaatcagcggagatacgagggccttccccgaactgatcgctacggagtgcgaggtgttgatccacgaactttcccttccctttggttacagggccgactttcacacgactcccgagaatttaaaggattttctcagctattgcagggctaaaagaatttatcttactcatctatatccaatgacgctggcccagaaggataaaattctggaatttctcgatttcgaggcaacagtagcggaagatctaatgagttttgagctatgaagctcagatcgaagggttgtaatgcttatcagattgaggatcgcgaaagaaaaattttaatcgacactggaacagatggaaagctattggcttcgcaaatagatgaattggatgcgattcttataacacatgctcatttcgaccatgttgcgggcgcttggaatctggagagagtctttggatgccctgtatacgtgcacccggaagacattccttacgtattgggtgaaaatgagttccactttggcgggattctgggagcgatggctaagttatttgaaaaatttgtcggctacagggcacccgagaatgtgaaaagcatcttcgaattcaagtcatccttaaaaatcgtgcaccttccagggcatactcctggcagtgtatgcatattcaaaggaaaggttgcatattgcggggatttgataagaggaggtggaaaaacatctttaaagagtttttgtagcgattacgaaacatacaagaaatcctttagtgaatttttggaaatggactgggaaaaagcatttcccgggcatggaaaggaaatcttaaaatctacctcaagcccctgaaacctttccctatgaagtatatctctgcgctgctctttctcgatgccttggggctgtgaaattttttgaagccgaagtatggcttaagttcgttgtagaagttctgtatttcctccccctgaaagacctttaccagaaaattacctccgagctttaaaacctgctgtgcaattctgaaactggctttggccagatcaatagagcgcaggtggtcaatagtccatattccgctgatctttggcgacgcatcgctcatgactacatcataatactcgctgatctccagcaatttgtcgattagcgactcatcggttatgtctgcctgaatgaattcaacaccctcgagcgcgtccatggggtttatgtcgaccgcgacgactctcgctcctagttccacagctacctggctccacccaccaggggatgcgccaaggtcgagcaccacgtcgcctttccggatcagcctgaaatcgcgattcatctgcttcagtttgaacgcggctctgctgcggtagccgagtttctttgcttcccagtagtagtgatccctcttgtccctcatagcgctctaaggagaacccttcttgttctcggtccatcgaattcgagaaacagaaccctttgccaggttccaagctgaagctttccgttttcgaagggaaccacgactgcattccccagcaaagaggcctttatgtgggctgtggcgttgttatcgatatcgttgtgtctgtattttgcgttggacgggacaattcgatcaagagcactcagaatatcctgaagcaacccactttcagcctcgttaaggattatcgaagccgttgtgtgtggggtgtagatgagagcgacaccgctcccttctagcactctctccacctcggcagttatgtccagaatttcctccttcttcttggtcctgagctctatctgattcatcaagcagcattgctggcagtttatttatctcttccgccctaatttaaaacctctttataaaacactcccttccatcttccagcctgatctctccgatctcgatcgtatcgaaatcggactgcgtgcactcaagcaggatgcggtcattaatactctttatcagacctatccccaagtatttcttccccctgtacaatcctgcgacgaagtttctggcttcgctctcagagaaaagaaagatgtcgtcaacttcgtagagctcctttaactctctgaggatctcatactcaggattgcagtcttccttagtgcagatcgtgagatgctcttcaccaagccttgcaaatacaactctcgtcccaagaacgctctctataaactgagaaacgtctttccccttgaaaaaatcgggtttcagcttgatcccatctctttccagctcgaaagtcttagctccatccagaaattttcggtaggattcaaacctcgccttggccctgtcgtttttatcccttctaatcacgtatcccttttcaacctcaaaagttttccagtcaaaaagcttgccctcgaatgaggcgataagatctggctcaataatctcgagctttgccagtttgtattccttcgcccttctcccccttacccatccagtcgtatctattatttttcttccatcaagcttccttagctccttccagagtcttgcaactccacgcagacatttcgcctctcttccttgaggcgtaatgctacccacgaagaagccgttgatcatcgaaaccttactcagactcacgacatccttggcaaatccgtagcccattgccccgggattggccacatctgcctgtccgatatcgagatcgagcacatatgctccgccaactttgttcgcaagataagttgccaaggtactcttcccgctgtcaacgcccccgtaaagaaacacggtttcccagtccatttttgaaagcttctcccagctttccggtatagttgaaccatctaggattttgaattcgccgtttacctcgatctcacagtcttcgaggcagaagactggaacgaacttttcgctttcgaaaaaattaagcctcgctccaagtacctcgcattctccccttatggaggcagtaccctttatgaggaccgtttttcctttcggaacgatcatccttccttaggagcctccacttcctttataatgactcccatcactctcgggatcgcaccgagaaccttcgtgaagtttttatcccccatcaccgcaatgctgaaatcatcggagttctcgaaatagtagtaatcgaaatccttctcggtctcgtttaattctgtgaagaaatagttctgtacaaagtgcactccccaaaccttctcgtactgctgagaagtgaagttgtatctgaatccctggaag

>Bin11_33

tcgatgcaggaggaagggggaatgcaatagcccatgcgttttcccgcagtgacagagttaagagggtttttgtagccccaggaaatgcgggaagcgagttctttgagaaatgcgaggttgcgaggctcgaagggagagcaataagctcaataagagacatcgagagcatagtcagatttgcaaagcaaaacgcagatctcgtcttcatcggcccagaagagccgctgagccttggaatagtcgacagacttgaagaagagggtttaatggtggttggcccgaggaaggagcagacgattttggaggcgagcaagtgctgggcgaaggatttcatgaagagcataggtgtgccgattccagattacaggaacttcgatgatcctgaagaggcaaaggaattcataagaaactcgagcgggaagatagtggttaaagcagacgggcttgcagcaggaaaaggtgtgtatgtttgcgattcagtcgaggaggcattaagggccgtggacgagataatggtcgagaagaagtttggatccgcaggagacagaattgtggtcgaggagaagcttgagggaatcgaggtcgcattcacagcactttgcgatgggaaaaaggcaattccattcggtcatgcgaaagattacaagagggcatttgacgatgatgacttcgaagcgctcagggacttctacatcggtttaaggaggaaattctacaagagagaggagatcgagagactttacaaaagcggcctgctcctgaatccgaacacaggaggaatgggcgcgataagcccgcatccagaagtcaccgtggaaatcgaggaaaggataattaaaaaggtcgttgagccgatagtgaagcgatcgggcttcaagggtgtcctttatcctgtgataatgctcgtagacggtgaaccgaaggttttagagataaacgttagagaatgcgatccgggagcacaggccaagcttccgaggctaaaaagcgatttgctggagctttcacttgcagtagttgagggcgaacttgattctgtcagcgtggaattcagcgatagcttctgctgtgcagtctgcgcggtcagcggtgcattgaaggggagggagggtctcaagcccggatatcctgcagaccattacacgagccagccgataaagggaattgagaatgcgaggagaattgcggaggtatatgcaaacggaataacgaaattagatggattcgtaaccacgggagggagggtgctgacagtagtcggcagcggaagtactttgttcgaggcgagagagagagcttactcagcccttaaagagatcagcttccccggaatgaggttccgaaggacaataggctcagagtatctttctaagtagctcgagatcgcccaaataaactacttcattttccaaagcgtagacttcgatccttttcgattttctcaaaactggagaaaggaactctccctgaagaacatcgctaccagaaacgagaggtgaaaaagctggaaggacgaacttcgtttcatccacaaccaaaaagcagggaaaagaataaatcgcccctcctcgcctgaactttatagctggatgttcgtggccgattattgccctctcaaagccaaaatccttgtgtccgtggaagacgtagtattctccgagcttctcgtattccttcagttcgatggagtactccgaaagaattgcggataaaaagttgtcgtgatttccgcgaacgaccgagatctcgacgtctattgagtccaaaaaagctctaacatcatcccactcgtagggcaaatttcctgagaactcgtgtttcaggtctccagcgattatgagtctctcaacctcataacagtccactatttgcttaatcctgcttattatatctgcaatctgcatcctcggaattgcaattccagcattttgcatcgcattctctatacccagatgcaaatccgcaatcaccgccgttttcttagcgatcagggccttctctggagtcagcctgaacactcagctcgtcgacagaaatatttaaatttctttcgtgaaaatattcactgaatggcatacattctgctttcaggttatcctggagccggaaagtgcaaaattttaaaggaaattgccgcagattttgagaatgtcgtctggataacaacgacctacagcatcgagaatgtaaggaatgggctgaaaagggatgcctgggtgattgatgcatttagctgggggatgaagggattaaaggctggggaaagagatgtggttgtttcaaatccaacaaacctgaacgaagtgagtctatctttttccaaagtccccgaaaaaattaaaggggattatctcctgatcctgaactcgatttcggggcttgcagtctaccagccccttccgaagatcctgaaccttttgagatccctcatcgtgaaggtcgaaaaagatggtgcgaaggccattttcacgatagtaaagggagctcaggagcgaggctttgagataagtctgatgatgttcttccccaacatagcagaaatcgaagagggaaagttaaaagtattgaagacaagctattctgagttcgaaaaaagagtttatggactcagtgaggcaaaacagatattaacaaaaatgctcttctgatttcatggacgttttttacgtggactcgagggcagttgtccttgagccagagaagtggtaccagccaaagctgagcctcgtaagcaagctgaggaggctaatagacgaaagtggaatccttgatggaattcagaagggagacatagttgccgtaaagacccatttcggggatcggggaactacaaagactctgcgaagtgtatacataaggtcggttgttgaaaaggttattgaggcaggtgggagacctttcgttaccgaaacgacgggtttgggaatgataagaccgagatccactgcaatagggcgtcttgagattgcggaagagaacggctatacccagcagaccttaaaagccccgatattgatcgccgacggtcttcttggcctggatttcgttgaagtttcgatagatgggagatacctgaagaggatatacgtcgca

>Bin11_34

gaagaagttcttcggaaatcttctcgaggaggttatcaaaccgggaatttgctctcactgcacggcctgcgccgccatctgtcctgtgaagggcattactgcgggagacaagcccatagactttccgaactggctcagggattgcgttgactgcggagcttgcgtgaaggtatgcccgaggtgggaatacaaaccgcttaacggcgttgggagatatatagaagctttctcagcaaggtcgaagagatttcgcggccaggatggggcaatggttactgaattcacggcaactgcactcgaggaggggatcgtggagaaggcgatctttgtcgcaagggatgaggagtggaggacgagagttgtaacgataagcaacgtcgagcagctgaagagcgagaaggtcgctgggacaaaatacagcttcgcggatgtgcttccagcagtgaaggaggcggtcctcgatgcaaatgccgttgcctttgtcggaacaccgtgcatgatctctgcacttagaaaaatgcagagaagcttcaggaagttcgaacgggttaagcttgcaattggcctcttctgcaccgagaacttctaccactcgcagctgagcgagtttttgagcaagaagggtgttgctatgaaggatgtgctcaagacggacataaagaagggaaagttcatcgttaagaaggccgacggcgaaatagtattccctgtaaaagaactcgacgagataattccttcgggctgcaaggtatgccaggacttttctgcagtcgaagcagatttaagcgtcggcagcgttggtagcgagtccggattctccaccgtgcttgtgagaagcgatgtcgcgaagcgcgtggctgatttgataagggccaaaggcaacgctgagttcagagatgcaagcatagaagctgtgaagaagctatgcgactacaaggtcaagattcacccctacagccgctgagcttcatccttttcatgtgcttttccaccattatcctttcgaaatttttgtaaatttccaagggttctggcagcttcctgtaaaaaatacccgctcccacggggtcaattccagattccattgcctttctgatccttctttccctttcaaagatcttcatcctgtattcagaaagcctggtttcaagctcttctctcgcaacaggcattccatgggcaggaatcgccacttcaacgtcctcagccattttgatgagccggtctatggaatccacgaagtcctgcacatcgcagtcgatacagccataccaggggccgaagggcgtgaggtcgatgtcggcgaggaagagaattcttccctctatcagaaagcagcagtgaccagcagaatgaccgggcgtgtggatcacttcgacctccttattacccaatttcagaatctgcccatcctctataaattcgtccactttcctgaacccgagtttcggataaagaccgaggaaaagcttaacgctttcaccgaggccgtaccttctctcgaattctccatagctttctatcgcctcagcatcgagctcgtgtgcgtagattttcttcgctaccgagttcatagatatgtggtcctcgtgccagtgagagttcaggatgatctcgggattcagctcccaggcattctctgctcctccgtctattaaggcaaaatcttcaatgtatatgcagtttgcccacggatacttcccccatttttcacccggaattatgaaaatgccatcatcaagtttctgccagccattcacagctcgaattcttctcgatttatttatgcctttcctccgggttaggttttaaaagcgcttcatgtaaatgcacagcgtgatcgcaatcctgaactacggtgttggaaatctcaggagtgttttcaacgcaattgaaaaagttggagggaggccgaagataacatcagatcaggacgaattaagagaagcttccgcgatagtttttcctggtgttggagcatttaggccagcgatggagaagataaagagcattggatatgtttttcccgaagttccgaagctcggaatttgcttaggaatgcagctttttgccacgagaagttttgaaaacggagttcacagcggtttgaactacatccctggagatgtcgtcagatttcccctgtctgtgaggaagatcccgcatatgggctggaacgaactaaaaataagaaaaaatatagaaattttggatggaattgaagacggtagcatggtatacttcgtccactcttactaccttaggaccgaggaaaggtttgtggtggctgaaacagagtatggaataacttttccttcggtagttgcctgcaaggactgcttcggatttcagtttcatcccgagaagagcgggaaagtagggctggaaattctgaagaactttgttcagatcgtgaggagatagctgaggaaattaaataaggccccaggaagtcagccaccctgtgaatccgccgtaaagaagctgggctgcgaatgcggcgattataatcgtcatgaatctgcttatcgccctgattccgtttatccccagcacccttatgaggagttcggaataacggaggagcagatatgctatcagggagactaggattatgcttatgagtagattgagcatggaaactgtttttgagaatacaatgagcgtcgtcatagtcccagggccaacgagaagcggtgtggcaattggaactatcgcgacttcttcctgactttcaacagctttcgttctcggaagcccgctcagcatgtctattgcaatgaccatcaaaagcactcctccaccgatttgaaggctgaaaatgctcactctgaagaactcgagaataaaactgcccccgatggataagatcactgcaaggatcagcacagtgataatcgactgtctgacgattttatgtcttttctctggatccattccctcagtcaacgaaagaaatgtaggaatcactccaacgggattcaggactgcaaagatctgccccgtgatggagaagatctgaattacctcgagcagcaccatcttgtcacctttctgaagcaaaggcgagcagatttaaagcctttccaacttcaaaagatttttaatttgcacaatgttagagtgaggcatgctttatggcgccgttatcggcacgattctcggtttctttgcaggaattactccggggattcacagcaataccttctcggttttaatcgtttcagcttcagcgttcctctttcttcaattttctcctgcagacgtttctgcaatgatcgtcacctcagcgatcgcctacacggttgccaacataattcc

>Bin11_35

aggttccgttacctcctttctgaaaactcccgcgccaccgagctcaacccagcccaagccctctacataaacttctggctccacgctgggctcggtataggggaaatatcccggcctgaagcgcacatcttcaaaccccatctttgagaagaacgtttttagcaaaccgagaaggtgtttgaagccaacattattgtcgagcacgactccctcgagctggtcgaattctggaagatgcgttgcgtcaatactttcccttctgtagactctatcaatgcaaaaggccttctgaggaggctcgggattcattgcaagatattttatcgtaatggcagtggtatgcgtcctcaagacgagctgctgtgcctttttcaaatcccatactcccttccatccagtcgatcccgttatccagccattttcatgggtttccttgacgtttataacgttctcgtaaccgatctcctcatacctatcaaggtagaaagtgtcctgcatgtccctagctggatgatcctgaggctgaaagagggcgtcaaagttccagaaggaaagctgaacgtaattaccctttatctccctgaatcccatttcgaggaagatcttcctgcactctcttattattctctcatacgggtggctctttccgccgtatatttctgctgagggaatttttacatcgtatctaatgaattgcttgcccttccagccacctgagatgatgagttcgggatttaaatccgaaattgcctccctgaattcgaagtcagcctttttgatgaatctaacaagaatggatttttcttcacgcttctccacgagcttcctcttctccagcagcctgagcagattctcatcgatctcctcttccatttcaattttctccaatgcctccctttcaacgatttcgggtttggatttgagctttactttcccctcttcaagctccaggatgttttttctcctcagccagcctactgcgatctgaaactcctcgccaagcttagatttcagctcttcaatcctatcagtctgggatatcagccttaggagcttttcctcgggaaaaccctccttcaggtatcttttaccctcctcagtgagataatatctggtctttttaatttcagaaatctcaacataccctttttcctgcaaaagataaacggctttcataactgcatccctgttcatccccgcaagcttggagagctcttctaagctgtaaactttcccaacttcagcgcatcgcagcacctctgcctcgattttggaaagcatatctgcagtttgcgaaaagttttaaagtatttctactccattattgaaggtgatgttatggatagaattccagagcttgttgaaaaacttaaaggtggcgaaataaaggcttctgagcttgttgaagactgctttgaaaaaataatggaattaaatccaaaaataaacgcattcgtcaccctaaatccgaaagcaattgaagaggcaaaagaggccaaggataaaccgcttgcaggactgccaattgcggtaaaggataacacggacacgaagggtataaggacgacctacggttccaaacttcttgaaaactatgttcccacagaggatgcggttttagtcgagagattgaagaaggcaggcgcagtagtcatcggtaagacgaacattccagaattcgggcttatagcatacactgacaacgtcattttcggccctacaaggaatccatgggatctgagcagaactgtaggcggttctagcggtggaagcgccgctgcggttgccgcaggaatggttcccgctgccacaggaaatgatggtggagggagcataagaatcccggcatctttctgctccctttatggactcaaaccaaccacgggaagaattccatggtatccgtcgatgcccatatttattggaatggttagcgagggttttctcacaaacttcgttgaggacaccgcatttcttctggacttcgtgaagggtcctgattttagggacatgaactcgcttcctgatgacggaaaaagctaccacgaaggtcttgaggatcccgtggacggcgtcagaattgccttctcccccgacttaggatatgcaaccgtagatcccgaggtagaagaggtcgtaagaaaagcagcttttaagctcgaaaaggtcggggaggtcgaggaggtaaaggtcagcgttccgtgtctcgaaacagagctgactctcaaagtggtcctcgagttcacgtcattcatttccgagaagcttgaggactggaaaaaggtggcctttcctccatacctgggttttctgaacatcgccgagtcctttacttacagggaatacataaaaatagaggaaagaaaaatggagctttggagagccttgcgcgagatcttcgaaaaatacgatttcctcataaccccaacaaccgcggtgaagccttttgagctcggaaaacttggccctgaggagattgcaggaaagcccgcaacgccaataggatggatgccgttcacctaccccttcaacttcacgggcttgccagcagcatcaattcccgctggcttcagcaaagaaggtttgccgataggaatgcagatcgtaggaagaaaattcgacgacctcggcgtgctgaggatttcaaaagcctatcaagacctgaacccatggcagaacgtaacaccaaagctttaatttatttgagctgaaggcgcatgtcgtactcgtagtaaggggcgacgatctcgaatccgagctttctgtaaacatggattgcccttgcgttcgtcctctcggttacaagagtgatcgcattgaatcctgccttcctgcagaaatctatgatcagcttcatcatctcctgccccaaaccaaggttctggtaatcctgatggatgaagatcgtcaggtcgacctcatttctctctccaggcactattacgaggtgcccgacaatttttccatctttttcagctatgattgcgaagccattttgacccaaataatcgatccagcctttaatggcctctttactaaggggagggagacccaggcagcggaacctgggatcgtagttaacgtacatctcgatgagcttgtcacggtccattgaatggtcatactttcgaatgaccacatcctctccatttcgcagttttaccatcacgctttcaaatgcgggtagcctgagcgtggattccacaatataatccaacctctcatccttataattttttctcgaaatattaattgaagtagaatgataaagttcgcccgggaaccgaaaaatttaactcccacccttaaaaatttggccaatgttcaagtgccatctatacaactggaatgacatctccaagctgtgtaaggagctcgcgaagaagataaaggcaagcggatacagagtggacgtgatcgttgctgttgctcgcggtggctgggttcctgcaaggattcttgcggatcttctggaaatcaaggaattatacagcgtcaagactgagcactggggaatggtggccacaataacaggagaggcgaagatcacgcaacccctgaacgtaagccttgatggtaagaacgtgctaatagttgacgatgttgccgacacaggagaaacgataaagatcgttagagaacacgttaagaatttgatggcaaaagatatcagaattgccgttatcgactacaaaaaaacctcaaagttcattcccgattactacgctgccgaaatggagggctggaagtggattgtttatcc

>Bin11_36

atggttccaagtttcgcggacctccagaagttctgggaagatgccctaaggctgcagagagagtttatcgaatctctctcttcaacaataaaacttgtttcgggattcagtgtgatgagcaaggatattgcagttttcagagcaaaaatacaggctggaggcagaatttcgattcctgaagccgagaaaatcgcgctgaaacttcaggatggggacgttgttaaggtaatcatcgtgaaagaaggaggtgagaagtatggagatgaaggaaatgatggaagccggtaggggtttctacaagatggggtttgggattgcgaagactaccttggacttgctgaaagtgagcatggacaactatgtgaacatgtacgagttctacatgcgccagttccttccaggtgagagcttcgaaagccttaagaaggcgatacagctttacaccgagtctcagaatagagtatttgaaaacttcaagaagcttttggaccagctcgagaagcagcaggacgaagtttacagcagaatgttagaaatggctccaaaggaagagaagaagaagcagtaaattttttatttttttattcaagatttttcgtggcatcttacgtgcttgttctgcacaacgaagtgcaaagaagaattttggttggcaagcttggagaaatcgaattcaaagatggtttttattactatgttggcagtgccgaaagacttagcaggttaaaaaggcattttggcaaaaagacgaaaaaatggcacatagactacatctccgaagtctttgaagttctcggagccatgctcgtcaatcttgaagagtgtgagctggctaagaggtttaatttaccctcaatcaagggcttcggatgctcggactgcgaatgctattcccatctattctactccagaaatttgacgaccgaatacctgttcgcgtgaacctctatcccccttttcccctccttggcgtatccgttgtagatctcgaccagcagaccgacttcggcttttctgtaaatctccttatcccagacaatcactcttatccttccgctctcgtccgagatgtatatttcgttcgcaatctctgggtctccaataccgcttatccttccccttacattcacccttccaggcgtgatttccgaaatctttgtaaactcctgattttcaggagagtagtccgcaagctctttcgctgtctcctcgtccaagagatctccaaatctctccctgatctcttcgagtctctgcctatccatagaaccaccatagccagagcgttgaaagaaatccaataaggagcaggatgagaccaattttttccctcataaaacccactccttcagaccttcaggagcggaggcgaaatatctcaggcactcgtattccatcacatgaatcctccttgcaagtatcacgagggtgtggagaggatctccgaaatcgaagtatctcagcttctcgagctcatcgcatttaacaacagcattctcacttccggcccttgctattccaacagcaaaatgctctgcaaagctgctttcaaggctcagaattctttccactgcttcttttatcgtcattggcggatttagatcgaggaaaagcagtgtgtgtgcatctatgctcagatttgccttgatcgtgtcgatgaaagctttcgagcgatgccagcttacagtcgctgatcttccgaagcgatagttctgaagtcccgttagcgagcagacagcgttgattatacttgcgttgtttattaccatggtctttacaccagatctttttgcttccagtaggagagagacgtgagtggtggcaatcgttggatctccgggaacgaggattgcgactttcttctccttcgccatttcgattatctttctgctcccctcctccagatcgcttctctttgcctctcttatctcttttccaaaaaattcctgaagatcttcgagcgagcaggagatcttggatgtgtaagtgtccaaatacacttcctccgcatccctcaccgcttccagtccacgcagggttatatctttgtagtcatggagcccaagtccaaccaaggtgagcacggataaccttaagctggaggtgttaaatcttacgccttgtgccatacagtcatggcaaagtttttataagaaggagaagaataactctatgcggtggttttttgcactggctatggcactgctgctctgctgctgtgttcaagaggagacgaaaattgagacaccgactccaacgcctcccgaaacaccgaccactacagtgcctacaactcctctgacaactccttctgaaaagaaagggctcaaagaggttgtgccagccaaggaaagagaagaaattccaaaggagttcctaattaaactcaagccatggcctctggtcaggggaactgaaaggagtgactgttacacgcccgagatcccgagaagaccggaaattgcgggtatagcaaatgtgagcgcgaatttcagaggagaaatctcaacgcctgttgtgaacgacatcgttttcctttttgacagccagagggtttatgcatacagggatgaacttctctggagctttaacgtcgaagaatctttcggaaaggacataaaagcatacgccctcggagattacctttacgtaggaactactgcaggcaagaaaggcttttctttgattgcatttgaaaaggagagcggaaaaatagcatggcataaagaggtagatatagctggaagcgtttccgcgttaacggtgagcgacctcgtctgtgtgggaaccgacaacctggatccttgggttatgtgcttcacgcaagacggcgagcttagatggaaggcaaaggttgcaggaacagtgaacggatttgcagtaggaaacgggaagcttttcgtttcatctaacaagctctacgcatttgaactgaaaacgggaaagctgctctgggagatcgacaagagttattccgctccgttatataaaaacggaattgtcttcgtaaccctttacggatacgtttacgcattctctgaggacggaaagcagctctggaagaaatacttcggtgcaggagaagatcagtatttcaatcccttgctctccgccagcgactacgcgctttacattccaaggaccctcggagaaaagaatctcgcccttcaagttgtcgatttcgatggaaatttgctcgggttgttcaatctgaccaaggatgaaattccaggctttccggttgcatcgaacagcgttgtaattcttcctgcaaagacagaaagctacgggaagatctacattttgtggcgaggtactgaaaagctctacgaattgaagcgtgctggaagtgaagtgttcatgccgaaagttgctgtatcgaatggaaaaatatacgtcgtattttcggacaacagaagctcccagaagctttacgtgctgagtgacaggagtgtgccaaggatagcttcagtagaagtttctgctgagaatgatgaacttgcgatctccgcagtagtgagcgatccagagagcgcgatttacagggtctctttggcgtattttgatggaggaaaatggaactacaaggacatggatctcggtagaaggtatgtaagggaacctgttggtggatacggactatctgaggaactttacactgcaacgctaagggttgaagaaagggtggagttctacgttgtggctgtggacaactcgaacaacgttgcctactcgaaggtctacgcctatgctttaacttagcctttcaacgacaaaactctctcccttcttttttatcgcaactctcatctcttcccttccatcgaaacccatttttctgagttctgaaaaatccgccttgctcaggaattcaactaatagctccagctccttctctttctcttcatcctcgcgctttaaaagatgggacaaaagctcctcatatctcctcttcgctgcagtttctatgcagtgctcagagatgtgaagcacgatcatatgctcttttaacattcctttcgacctcttcgtaaagatttcttccctcagcatcaattgcaacgacacatgggccaaagttttcaacttcaaagacccaaacagcttcggccatcccaaggtcttcccagaacactccctttatcttccttattttgctcgctgcaaatgccccagcaccccctggaaatgcgaggtaaactgctttccccttcattgcttccacaacttctccactcatccctcctttccctatcattaccatcagatcggcgccttcaaggatcttcggagcatacgggctcatcctttcggaagtagttggtccagcagagaccacgcgatcttcgcttattatcgggccgcagtgataaacaatggagcggttgaagctgaaaggaagcttctgcttagattgaagcatttcaaccgctctcgcatgtgccttgtcccttgccgtgaggatttcgccggtaacgtatactacgtcccctgctcttagattaaggattctttcatccaccttattcagaatcaaagtatcacctccgcccttcgatttgcccagcactgtatgttcaccgcaactggaagcgaagctgtgtgacagtgattcagcattgaaagcacagctaatgctgtagtctttccaccaagacccattggtccgattccgagttcattcaccgctttgagaatctcgatttcgaaatcgttcatgctgtcaagacttctcagcaaggccttctttgcaagctttgcggagacgtcaaaacttccaccaatacccaagccgatgatgatcggcggacagggcatgcccatggctcttgcaacggtttctacgacaaa

>Bin11_37

gctctatcgctccaactgggcactcatctacgcaattcccgcatccatcgcacttgagcttatcaacaaccgcaggcatagctttcataggctttcgctatttataagcttctttcacttctgtgagttaaatttaaatctaataattctacatctatcatggactacgcgagatttggaattccaaagcttgatgagtatcttggcggcggcttggataggaattctataagtttgataattgggagaagcggaataggtaagacgatcttagcttctcattgggctgcagagggagtgagaaatggcgagacggtaatctacatctcaactacgatgaacaagtacaactgcagggattacctgtctcgcttcagcttcatgaaggacgtttacgacaagatccactggcgcttcataagggttgagcccaagtatctgctcccgatgacaaaggagaaggttaaagaaggattggatgctacttacggcattccggcagagcaaatcgacagggtagtctttgattcatgcactgacctggacaaaacgctcgcggatccagtgctctacagaagggcaataaggtatatggcggatctcgcgtacaaataccaagttactgcgctctgggttgaggaggctccgatgagcggtgaatggagcgagacgaagaatttcgtcgagacagttttgttcatggatatcctgaggattccagaaggctatgcaagagcgatgaggatcctgaagaaataccgaactcctcacccgctcgaatggttcccttacgagataacgaatgacggaatcgttataaaggacgggtggttcgtgagaaaggactatgactacaagttcaaacctcacagagaagattaaggatgcgatagaccggggggcagagataatacttgtgaaggtggatgcaaagaactacgtcagggttagccttgagatcgttaaattcttctcgagctttgcagatggtgtctacgtaacgcttaacaagccatacttcagcctgaagcggatgctcgccaaggagaacatcgatttaagaaagatgtacttcatcgacagcataacgatgcaggttggcggtgaggtgatcgacgaggaaaggtgcttctatctgagctccccagaccccgttcagcttcaggtcacgatcgagagagccatggacctcataacatcggaaaaccgtttcatatacattgactctctttcgaccatatctctttacaaatcattcgaaacactgataaagttcctaaggcac

>Bin11_38

gaaaatttcctccacatttagttcagccagttccctgccaatctcagcaagtgttggtgcagggatctcgttttccccgatgaattccatcagcagaacgttcttcatgtaagcataaggcctaggcacgcgaactccagcttcgtatgccctttccaggtttctgaactccttctcggtccagaggtaaattttatccctcggcgatatcctgtagtcgaaccttctgtctccaaagatgtactcgtccatcttgtcgaatgagctcgtttcgattctgtagatcttcacggccatcggcaccacttttccctcgtaaattccgtctgcatagaaaacgtttgcctcctttcccgtgctgacaacacctcccattgcctttatatatttcgtcgataacttgtagagcgtcttgagagttctgagatccagaacttcagcgtatatcttcctgtcctcctctcctatctcctttatcctcagtttatccagaaacctatcgaggttctcgatagagtaattagggcggggtttttaaaaacttactcttcccatccccctcttctttcgaggaatacgaggaaaagcgaggctgagatcagcagaattgccgccagtgagatgaaggaaacggagtagtccacgaatctgaatttcacgtagtagcttccgttcccttcaagcagacctattcttactccatcaaactgctgctccctgtagctcagatttctgtattccagaacttctgcatcggggattatgaccactctctccccgctcagtttccgaatcatttcgccagtgggcaacgtgtaagcgattcttatgatccccgactcgttttccggaactagaagagacctgaaggtaatcgattcgttcgaaatctcgatgctcaggaagtcgaggctatttgttatcagccggaaattcgtgaaatcaggcataggaattgtcagatctccttcaaagttcaggtcacctctgttcgtgaccacgatcacctcgtcaacgctcccgttccgcaaaacgcttgcatgcaacatcacagaaagaaattctgtggagttcgtgaaccccagattgaattcaaccgtctggttttcctcaacccgtagttttttaatgaacgtcttgttcagagcacttagttcgaacgtgtaattcccactttctatgggaattctgtctcctgaaaatacaggaccgctgaaaacggcttcttttccattggaaatggtaagcatcgctccagaatcgtgatttttgagtataacagtaacctcgtatccctgaatgggaatcaaaagcagtaggagtaagctaagcccttttaatcgcattccactcaacctccttcctcctcaagtaatcgaaaaaggcagaaattgcagaagcctgaaggacaaagacgtagatcacaaggccaaggaataacttcagcttggagatctttcgatagtaagccagaattagcgggatcgagagtggaatgagtggtagcattattatcgggaagtaagttactgtaatcgccagataccaggaaattctcagacttctcgcagctctcttcatctcctttcgatacctccagagttgcagcccgccgaaataccaccttttccgctggctgtaaaagtctctccagctgtaaggagcttgctccaagacttttccatcgacaagaagtgctctgcgcccgcgtgcatgcattctcgtcgcgaaatctgcatcctcggccatcgcatcctcatttagcccctcacaaagaatatcgtatctcagcactcctatcattccgttaaactgtttaaatgcgctttttgaaagcaggaagtttataagcctgtattccgcttcaacggtctcagagacgaggtttttggcgttgtaaatgtaccgtcttgcagaagctatgtaagccctttggtcagctaaaagtgcggaaacacaggcatttaccgttctctcgtcaattcttgtgtctacgtccataataaggacgaattcgggcttgaattcgctcaaatactttacggcatcgtttatggctcctgcccttttcccccttcttcccaccctctcaactacctcaacgcccatttttctggccaattctaccctggcatcgttctctgcatttttatcaattacgtaaacgactttttctgctctcaggctcttgacgtgccttatgctttcctctaacacgctcgggtcttcgaagggtgaaacaggaacgatgactgcgagggtcattcgagcatcttggcaatgatctttgccgtctcttcagggtctgctcttccacgtgtctttttcatgacctgtccaacgaggaagttcaaggcctgtttctttcctccgaggtaatcctggactgccttgggattttcctgaatggcttctctgcagaatttctcgatttcggtctttggaattgcatacaatcccttttcggcaattatttgctctacgtctccccccttgtcgagttttgtccttataacttccacagcacctttttctgtaatcctctcctctgcaaacaacttcagcagccttgcaaattcctcaggcctaaatctttcaaaacactcgtaaaagctccagtccctgtagttcagttctcccctgagtatatcgacaacccatgttaccgccacctttggatcgataagctttgccaccctttcgaagaaatcggtcatcttgaggtcaaggatcagaaccttcgcatagtgctcggaaatcccgtattccctcttcagcctctctttcttttcctcgggcatctccggcagagttccctcgacctctctcaaaagctcctcggtgaacactattggcaggtcaggctctgggaagtatctgtagtcctgctcttcctccttgctgcgaagcgacaccgttatctcctgcgcctcgtcgaagtgtctcgtctccctcacaaccgctttcccacgctttaaaaggtttttttgcctcaggatctcgtagttcagcgccttttcaacccccttgaaagatgagatgttcttaacctcaaccctgctccctccagcgatcgagacatttgcgtccaccctcatcgccccttcaagttcgccgttgaaaacatcgaggtactcgaggatcattctgagcttgttcaaaaaaattcttgcttcttttggagagctgatcaccggttcggttacaatttcgagaagtggcattccacttctgttgtaatcgataagcgaatggctggcagtcgttattgagcctttgtagctgagctttccgggatcctcttccaagtgtattctcctgagctggatcctcttttcgctaccctcggattcgattgaaacgtatcctccaatggcaagcgggcggtcatactggctgatctggaaatttttgggcaggtcagggtaaaagtagttctttctgtcgaataccgtcatcggttgaactttagaattcagagccagtgcaacctttatcgcggcctttacagcttctctgtttatcacaggcatcgcaccaggcataccgaggcatacggggcagacgtgagtgttgggagggcttttgtggtagttcgtaggacatgagcagaagagcttgctcctgagcttgttcagctgcgcgtgaacttcaagcccaattacgacgtccatggttaaagggaggatgatggtttaaaaaattcacccagattctcccggacaattcaagaaaaagtttttagccgtagccaacaggcttcgctgaaaccctcatcttttccgtctttatcctttcctggaatctctcgtagaagctcagcatcgtttcgttcacgcttggctttatcttcttcatggcctcaaggaaatgcctcatctcgaccttttcagcattcggatcttctctcaaagcaagcatcacagcctccctgcaaatcgcctctatatcggctccaacgtagccttcggttatctccgcaagctcctcaagatccacgtcctctgccaagggcatgtttctcgtgtggattttgaagatggcaagtctgctctttctgtcaggcggcttaacgtaaacgagtctgtcaaaccttccaggcctaagaagtgctggatcgaggatatcagggcggtttgttgctccgattacaaccactccgtgaagctcctccagaccatccatctcgatcagcagctggttcaggaccctctcaaccgctcttgatccctcatctattcccctcatctgggctattgcatctatttcgtcaaagaagattatgcaaggcgccacctggcgggcttttctgaagatctttctgacagctttctcgctctccccgagccacttgctcagcagctcgccaccctttatgcttatgaagttcgcctccgtttcattggcaactgcctttgcaatcagcgtctttcccgtgccgggtggtccaaagaggagtatccccttgggaggctttattccgaatttcctgaacttttccgggaatttcagtggccactctaccgcttctatgatctctctcttaacgtcctcaagccctccaacgtcctcccagctaaccttagggatttctacgaggacttctctcatcgcagacggttctatctcctttaaagctgagacgaagtcctcccactttacccttattgactccagaagctcaactgggatctcctcgctctcaaagtcgatctttggaagatatctgcgaagggctttcatcgcagcctccttgcagaatgcctcaatgtccgctccaacgaatccgtgagtttggtctgcgagctttttcagcatctcccttacgatttcgttttcaacctcactcaggagctcggggctcagcagtgatttcgctgctctctcgatgtcttcctcgctttcgagctttttaacctcctcaattgcgaactcgaggtctttctgaactttcagatcttcggtgtttctcgcaaccctgctgagagcctcaagaacgaactcacggagatacttcggttccaagggcatgttcctcgtgtggatctgaaatatctcgaacctgccctctctgtctgggactccgatctcgatttccctgtcaaaccttcccggcctgcgcagcgcgggatccacggcatcaattctgttcgttgcacctatgactatcacctgccccctctcctcgagcccgtccattaacgtcaaaagctgtgcaacaactcttctttcaacctctccagtcacctcttccctctttggtgcaattgaatctatctcatcgatgaatataatgctcggagcattctgctttgcctcctcaaatatctctcgcaatctctgctcgctctctccgtaaaacttgctcatgatctcgggaccgttgatcgtgaagaagcttgcacctatctcgtttgccactgccttcgcaatgagcgtctttccagttccaggaggaccgtggagaagaacccccttgggcggctcgatgccaaggcgcttgaagagctcgggatacttcaaaggtagttctatgatctctcttaccttctgaagctcctccttaagccctccaatgtcctcgtaagtcacacctgcctttcccatcttctcgaatcccttcgcaggatgctctctgaaaacaaccttcgttgtctcatctattatcacgactccctttggctctgtcttgaccgcaacaaatacgaccgcctgattctgctggccatacttgccgaagcctgtgatagcaggagaaccgacgagtggaacaaaatcaccctcaactaagggtcttttcaggaactggtgctttaaatattccccgggatctattccgtaaaccctcatctcgattttcttggttggagcaagaattacgagtctcgccggctcgtagtcggctttcctgacctttacaatgtcacccacaccgacgccggcattttctcttgtgaagtgatcgatccggattatgctctttccccaatccctcttgggagacctccagacctttgcaacagttttcctcttcccttcgagctcgacaacgtctcccggagagatctggagcttcatcatcgcatcagggtcgagtcttgcgatacctctccccgagtcactcgggtatgcttggttaaccttgagcatgatctccatatgaccacctaaaaggatttttattttagagataaaaaaggcttgtggtgatttgatgaaggttgcgatcttcgatgcattcaatggtgcaagcggggacatgataatctcatcgctgctcggcctttcgctgacggaggaagacctcagaaggactgttgaggaactaaggctcagcttaagcttcagagttgaggaggtaaagaaaagaggtatttctgcaaaaagggtcgtggttcgagagctgatggctgaaagaagtttttctgaagtcatgaatctgttagatcgctcgaatcttgagagtggcgtcaaagaggatgcaaagatgatttttaaaagaattgcagttgcagaaggcagagttcacggaagggattacagaaatgcagtctttcacgaggttggaagcgatgatgcaatcttcgacgttgtttgcagcgttaagggaataagaaggctgatggacgaaggatacagattcttcgcaaatcccgtgaggctcggaagcggattcgttgatacagcccaaggaaaatatcccgtccctgctcccgcggttatggaaattctcgccaattcaaagctcgaagtggttttcggaggagaaggggagctcctcacgccaacagcagcagcaattcttgcccagtactgcgatggcgtcttcaaacttccgctcagagtcgagaagatctcctatggagcgggaagctatgaaaccgggattcccaatgttctgagacttgttctcggtttttctgagcttagcgactctgtaattgtcttagaaactgtggtcgacgatctgagcggtgaggaaatcggatatgcgatggagaagctcagagctgaaaacctcgatgccattgcaatacctgtcatggcaaagaagtcaaggcttgcggttaagatcgaggttttgaccagattcgaaagggctgaagaagctgccctgcagctgatgagggagactggaagtctcggggtcaggatgattcccgtttaccacaggacgattgcggaaagagaatttgaagatcgcgaggttgagattgaaggaaagaagttcagggtcaggtttaagcactcaaaatctctgggaagggtgaagccggagtttgaagacgtttcgagaattgcgagggagctggatatgccgatctacagagtttacagacttctggagggagcgcatgctgataccgagcggaagtaagtgcatagactctctactcggcggtggaatagagactgggacgattacgcagctttatggcccgagtggcacgggtaagacgactctctgcctgatgtttgcgaaaaatgctgctaaaagctacaaggtggcgtatgttgacacagaggggctctctggagaaagggtcaggcaggtttttcaggatgtctccctcttttcgaacgtctacgttcaggaagtattcaccttcagacagcaggcttccgccctgaaggagatagagaagctgacaaagagcgagaaaataaagctcgttatagttgactgcttcacttctctttacaggagcgagcttgaagatgagaacagacagataaaggttaaaagagagcttactgcgcagctgacatacttgctcggacttgcaaggaagttggatcttgcagtgcttatcacgaatcagatgttcacagacgtgagaacaggagtggacagacctctaggcggaacaagcatagaccacctctcgaagacgataatttcaatggagagagccggacatctcagaagggcaacgctcgtaaagcacagatacaagaaagagggagagagctgtgagtttcagcttacagacaggggcatcgagccttagcggagcagcggaaggcttccggtaaccttgtctatcttcttttcttcgtcgggacccaagaccacggctgtgatcgttcccggcggtatctccgttagtcctgcatcctcaacgatcgcggtcggtattttttccctttcggccttttccttaataaagaagagctcttcgaggtttttaaccctcagcactattttcttctgtccttcacgcagccacctctccctcttctctttatcgctcttcaaaaagccaagtatgctcgcgtgagcgacctgaacggcaagtttccccctagatagcccgagatcgtccctaacaactattacctgcttaatctccatctccaaaaccaaaattcctgagttcttcctcactcaccgccattgacttctccatccacctgatgccgagctcagtggccttctcctcatcgaattcggatagctctatcttctctccatcgaatctcacgcagctcatcggaacagcgatgaacctttcgcggactttaacgatcagatggttgttgtaaacgtctatgctctccccgtattccctgccatccttgaaaacaaaacggcaaatcagatccatgcatttcctcggccatgactttaaaaaatttttgtctctgccttctgctgaacctcggggctaggatcagtagaagccgaaatccagatcccgatcctgaatgtagttctttccaagctttaatgcgatctcagccttcatcagctctctgccgagataggcggcatgatcaagcctcgatacgagttcaagcgccattatcgtgtccaatatctcctttgcagacctcccagcgatgactgccttctcgtgcttgcagaagatcttcccatcagcgatgaagattatgaagtccccgaggggatctctgacgaactccttgctttccttcgcctcaacgaactcctcaggcttttcagcagcctcatatctgactttttcctttaagaccaagagggaaattcctgtatctttgggtggagacctccttatctttgcagcttttgccagataactcgcaattctaagctccttaacacttttcaccgtcttcggactcgcctccgttgtgaatacaagattgcaaccgatttcctctgctataaaggtgaggagtgcattgattccgatgctatcggcatcgctgagctccgtgacgtttccgacgccaaaaaggagtggagtctctgcatcgcgttttctgaactccacgtatctcccgatggactccgctaccctcaagggtggatcgaggattggatcggcgatcaccttttctgtcttccttttagcgagctctaaaagccttaaaagactgtcaatgtctctttcggctaccaccacggcttggtcttcgattaactcgattatctccgtattcgatttcgaaaggctcatcaccatgtcaacgccgtgcctgacagcgatctctatcgcggatggcgaaaagctgtcgacgctgactgggacccttgagtgatctacagcgatcttcacagctttttctacgatttctctgtcaaattcgagtggaattccaaggtcgacgatatcagcgccattttcaacatagtagtcgatcctatccctcagcgtcatgagatcgagtttgtggatttctgcgacaaccttaatcctgctatccccaccaattcccacacttcctattttgaattttgcagtcttttcaagctcttccacagctttaatcgcctcttcagccttctttttcgctataagcctgcatgcagggattttgtgtgatagttcgactttatccaatagcttaagcacatcgcccagatcgtaggcatgcaacggcccaaggcgagtctttactcccctccttctctcaaaatctgcccagttgcattctgctgtaagcccagggactaaaacgagatcgtattcttcaactgggac

>Bin11_39

agcatcaacgattatctccgcatcgcattctgagatcttctgaattctgtagccagcctttttcagaatctcgaagttcttctgtgctatctcggttttaggttctccggcgaggaatatttcaacttcgaatccttcgagatgtctcgcagcaacgaatccatctcctccgttgttcccgcttccggcgtagacggcaacctttcctccgctgaacctcctcattatttcctctgcaacggcctttccagcattttccatcagctgaagcctgctgagaccaaaatactcgcagttcagatcgagaatccgcatgtcctttgagtttatcgtttccatcagagtttaatttgcctaaagcttaaaatttttgcccgaatcgctaaaaatttatgtttaggccgagaaaaaaatacgtggtcttcagcgggaggcacaagacggtcataggggacagggagggccttagactgatagggatgattctatcgcatgagaaggtgagtagggttatttttggaaggatcgagaacaagggctcaaggacggggcatgggctgagatttaagatcacaagagttgacgaaaggggaaacctgagggcaatactctcctacggatcctcgaatcaggagatccacatcattaccaaagcttccagcagagaggagggtctgaagatcgcaaaagagctttcggaccttttcagggatcattgagtgctcgcattccgaataaagcggtcttgtggctcatggccgatcttttttgtcccacttcgagcaaaatcgatttataggtgctggttaacgataagatcgatgtatcgctacccggttgaaataatttcggatatatacgtgagcaggatcggtggctacagctacgagcttggagaagatgagctgggaataaattcgggagctatagcgatgaagacatcattaatcccagaagaaacttttgtgaccctcggtattctcgacaggaaaatgaggccgtactttttcaggaaaaggttcattgttgtcggtatcgaggaaaacatggagtcttacgagatgacgcagaacggagaggttgttctttccgatgaactagaggaaaaggttgaggtcgggaaagaggtgataataaattctgttgagttcttcatggttgacagagatctctcggcattcgtcagggcatttatccggtttagattgaatgataactgaaaaagattttgcctgaaagccctctcatggggtgtaaaagtttaaatatcacacgagaactgcgtttatgggaacggtaaagcctgcctacataaaggtgatcgcgaacgaactgctcaagaagtatccggaaatgttcacctcgaacttcgatgaaaacaagaagctcgtctcacagctgacgacgataacgagcaagaccatacgcaacagggtcgcaggttacatcacaaggaaggtaaatc

>Bin11_40

gtagatgttcatcacatcctcatgggttatgggctcgaagtacagcctgtcggaggtgtcgtagtccgttgacaccgtttcaggattgcagttgaccattatcgtctcgtatccttcttccttcagactcttcacggcgtgaacgcagcagtagtcgaattcgataccctgaccaattctgttcggaccgctgccgagtatcatgaccttctttctctttgtcgggatcgcatcgttctcgtcctcatatgtggagtagtagtatggagtttttgcctcaaattccgctgcgcatgtatcaaccatcttgtacacgggtatgacacccaaacgctttcttacagccctcacatccctttcagcgcatttgaagatgaaagcaagctgtttatcgctgaatcccagctttttagcttccagcaaaacctctgctggaacctcctcaatcttcctctctctggctatcctcttaagcttctcctcgaattcgactatgttcttcaccttatcaaggaaccaacggtctatctttgttagctggtaaacttcatccacggtaaatcccttctggaaagcgtatcgtatgtagaaaattctgtcatgacttgtaaatctgagcttccgaattatctcttctctgctcggatctttgtcttttccatcgcatccaagcccatagcgaccaatttcaaggctcctcaaggccttctgcaatgcctcctcaaaagttctgcctatagccataacctccccgacgctcttcatctgcgtacccaagatctggtttgcagtcgggaacttgtcaaaggcaaagcgtggaatttttacaaccacgtaatctatcgttggctcaaagctcgctggagtctcctttgttatatcgttcgggatctcgtcgagcgtatagcctactgcaagcttcgcagcgatctttgcaattggaaatccagttgctttcgaagccaaggctgaagacctgcttacccttggattcatttcaatagcaactattctgccgttatctggattcacggcaaactgaatgttgcttccacccgtttccacacctatttcacggatgatctttattgctgcatccctcaactcctgatattcaacatccgtaagcgtttgcgctggcgcaactgtgatgctgtctcccgtgtgtattcccatgggatcgaagttctcgatcgggcagattatgacgacgttgtccgcgagatccctcataacttcgagctcaaactccttccagcctataacgctctcctccacaagcacctgtcttatcatcgaaagtttcagccccttagataccaaattcttgagttcttccctgttgtaggctattccaccccccgtgccgccaagggtgaaagcagggcggattattaggggatagccaacttcatctgcaaaatccaaagcttcctccacatcattcacagatttgctcctcggcacttcaaggcctattttaagcatgcactgcttgaaaagctccctatcttcggcctttcttatcgcttccgccttcgcgcctattagctcgacaccgtatttttcaagaatgcccatctcgtgaagctgtatggccaagtttagccctgtttgcccaccaagtgtcggcaaaagcgcatctggagtttctttttctatgatctttgcaacaatctctgcttcgaggggttcgatgtatatcgaatctgccatctccggatccgtcattatcgtagctggattggagttcacgagcacgactttatagccctcctcacgcaaggccttgcatgcctggctgccagaataatcgaactctgcagcttgtccaatgactatcggaccgcttccaatgaccattatcttgctcagatcctctctcttcggcattttaacccctcaaaagatccaaaaagcgatcgaagaagaagtatgtgtcatgaggcccgggacccgcttcggggtggtactgaaccgttattatgggcagttctctatgcctcagcccttcaacggttctgtcgttcagatttatctgcgtaacctcgaatcctcttggcagagttttttcgtctacggaaaagttgtggttctggctcgatatgaaaacccttccggtttctaaatcctttgtaggctggttgctgccatggtggccgaatttgagcttgaaggtttttgccccaaaagctaaggccgtgagctgatgtcccaagcagattccagccataggaagcttcccaatcagcctccttatggtctcaatcgtctccttaacccttgcgggatctcctgggccattcgagatgaaaacaccatctggattcatttccaagatcttttcagccggataattgtagggaacgagcgtgagatttattccccttttcaggagctgtcttacgatgctcatcttcaccccgcagtcgatcagaacgacttcgaatttccccttggcatcaatcctttttggctcccttacacagaccttgtccaccaaatcgatgtcgccgatgaaaggctgatcccttgccatccttatcagcttctccttctcaattccaatgcctatcgccgccttcatggatccgtagattctgatcttcttcgtcagcattctcgtatcgaccccttcgatgccaacgactccgtattcctttagcagttcatcaacgctcatctcgcttcgccagttgctcggctttctgcaaagctcccttaccacgaagccctccacctttacaccatcgctctcaaagtcctccctgcagactccgtagttgccgatgagcgggtaagtcatcatcaggatctgacccttgtagctcggatccgtgagcgcttcaacgtatccagtcattgaagtgcagaatacaatctcccccaatgcggttttttcggctccaaaggcctctccttccagataagttccatcctccaaagccaatgcagccttcataagagttccctcagccttctaacgaccgaactaatcttgggggcgtcgaagaacttccttccggttatctcgttgcagagggccatgtatagctcggaaacggcctccttaacttcaggatcgagcttgggaggggggccaactatctcgcgccagttttccctccccttgtatgcctttattctctcataccactcggtctttctgtagtaaattctgaggaactccttgctgacttcaaatccctcgaaactaaatcggcactcatctggtgttccgacagcatccacgaccataaatctcctttcttcatcgaaagcgagctctatttttccatcttcattctccaagcctgtctttgaaacctcggctgtgatgaggtcatcaatcttgagcacaatctccttaagcccctcaaattcctcatcgctaagtgaagcgatctttttcgcttcctcatagcttaaatacctgtcaacatcttcgagtttcgtgctgaagtccactattggcctttcaagcttcatgtttggctttatttccttaagcccgaagtcctcgggctttacctcaccgcttgcaatcctcctgagaagagaagaaccctcaggaatcttgtttctgtagattatctcgagggggataagaaagtttgccctttccctttcaaatatcgagtaatcccttcctgaaggcctgagaactcggacaagcttgaccctcattttattcgtcgccgagtcgagttcttcgagccttttaaccttatcatcctcaatcaagcccaagtaatgtgtttttatccctgactcttcaatcttttcaaagaaataggccgagataaggcaaagagatgctccttttccctcgatcttgtctggcatttccccgtaatcaaaaacgctgtatcggttggagaactcgaagattccctctccaagtccgccgtctggattcttaatcacgatcaaatccttaacgcttcccataggtcacctctctgtcagcctttaatatttcctccttcttcttcctctggtactcctcaacagctaatctaacattttcgctcttgatggcaaagatctttgcaacggccaaagctgtattctcggcctcaagcacgagcataggcgcaacgccagaaggcatccgaatgctggaaaatatgtccgcccctgcaaatttttcgctgtagggaggagaagctatcacgggcttggtagtgttcgcatctacaaaaccgctcaatgcattgcttcttcccgctatcgtaacatagaccacatcttccttctcataacccctgataatttcgagcacatgctctggcgttttgtgagcagaggctactctaagctcgctttcgattccaaaaagcttcagcttttccactatctcctttccgtactccaaatcagcttttgagcccatcagaatgacggctttcatgagaactcaccttcaggaattgaaaaacccaacgctccagatctcagcgatggggatgattgaaaaatcccagtttcctttctccttagctcagccgataaagtccttctatcttgcagaaactcggccaaggacatcaataccgccaatatgtcaggttaataagattttccctacagatagcattaaaagtttggcacgagaaaattcttaaattcaaaccctcaaattccatgaggggctcgtggtctaggggttatgacaccgcccttacacggcggagatcgccggttcgaatccggccgagcccatcggattcacatccttagtagctcgtcgatcttatctctgttgaaacccaacacccacttctcacccttcacgattagcggcaccgaatagctcttcgagatgctgtaaattttctccactgctctcttcttctcctctccttcgagtttgtccacccatacgacctcaaagtctgcctttgccatctttaaatattcgatcgttctcttgcagtgcgggcaggttgtgagaccgtaaacggttatttcggccataattgtagagataacggaactttttaagcgttttgcgggtttgagaccggcagaaagttaattatatatagtttgaaaacgagaaaattctctgtgaaaaacctcgccctcctcgctctgctgctcctcgccgctcaggcatctgcagccgagctcaaagtgggcgtatacgacaatccacctctagtcggaaggtctgactcaggatacgaggggttctacatagatatcctcgagcacatagcggaaaaggagggctggaaaatcgtttacgattacgacagctttcccaatctgctgagaaagcttgaaagaggagaaatagaccttatgaccgcaatagcctattcagaagaaaggctaaggttcttcaacttcacgaatgagaccgttatcccgaactgggggctaatagttgcgaaggagagatacgattcaattctccagctcgagaatctgagaatcgctggggttactcatgacgtgtacaccgagagcttcaagaaactctcaaaggacttcgagctaaactgcgaaattctggaattagaaggggactataaggatgtgctcgaagctgtaagattggagctggccgacgcgggaattgtctcgaggatctatgcttctctctacgcagaggattacggtcttaaaattacgagcataatattctcgccaataagcctgaaattcgcttcgaagaacggagaactgctttccatcatcgacagacacctcgcggaaatgaaggcgaacagtaactccgtttactacagatccatggaaaagtggtttggcttaaggtacgaagtcgttccgggctggttgtatcccttaattgcgggtagtgccataataatctgctccctattgctggggaacattttacttggcagagaagtcaggaggagaactgaggagcgcgaagaagcgctaagacaatatgaatatctctgggagaacgcgaatgacattttattcatccacgacctgcagggaaatttcttagaaactaacagaaaggccattgaactttttggctatgaaagaagttggagagttaaagtctgggacgtgattgtcccagaataccacggagtcgttgaggaaaaactgaagtggattttggagacaaagaagcccacaaagccatatgaactgctgtgcagaacaagagatgggaaggaggtctggctcgagactgtttcccaccccttcataaaaaacggagagatcgttggcatccacggaatagcgagagacattacggaaagaaagaaactgcaggaacagcttgagaagaacataatgcttatcgcacatctcacggacaggataagaaatatgctgacggctgcaagggcttactgcgagcttcgtgaggaactcgaggatgaagcagctgagaaagcgatcgaatgcatagatgaagttataggcctccttgaagatttcgaaaaagcctgggccgagtcagaaaggctcaggaatttgctatttggccgtgagagatccttatgaacttcaggggagtaccgagttccctgaccagccccttcttcttgaggtaattcaggtatgccaaggtctcgagtactgcgaggtatctgtcgaatgggttcaggctgtcgtattttccctgactccactttatctccctcgctatgctctccacgctcattggctcctcgccaaggatttcgagaacctcttcagttcttcgcgtgtaatgttctattaaatcctcgattctctgcgtagcgtcgtttatcctcctctcgtgagctggatatatgacctctattcctaagcctttcagcctctgaagggccatgagatagtcctcaagtccataggggtagtccagataaagccccagattcggagttgtgtcactgagaattgcgtctcccgaaaaa

>Bin11_41

ttcaaagccctggctgtctggatggactgggaaaggccctacatgactgtaaaggcagaatacatgaactctgcgtggtttgcaattaaaaaggcccacgaaaagggtttgctcgaaaggaaaaagatggtggtaaactggtgtccccgttgcgagaccgcactcgcggatgctgaagtggagtattccgagagagaggatccatcgatctacgttaaattcaggctcagaaacggcgaatacgttctcgtgtggactacaacgccctggacaattccagcaaacatggctgtggctgtaaatccaagactgaaatatgcaaaagttaaggcttacaggaatggcgctgcagaggtattaatccttgcagaaagcctgatagacgttcttgcaaagggatacgaaaggtgggaagttcttgagactttccctggcgagaagcttgaaggtctcgaatacgagcatcctttagctgaagaaattcccgttcagagaaatttcaaccacaaaattgtacttgctgacttcgtttctgcagagaacaccggctgcgttcacattgccccagctcacggcgtggaggacttcgaacttggtctaaaatacggaatcgaggttttcaacccggttgacgatagaggcgtcttcacagaattggctggaaagtatgcaaatctgagtataagagaggcaaatgaggtcataatagaggacctgttgaggaaggaactgctgattgcggaagagaagatcttccaccgctacggccactgctggaggtgcaaaacgcctataattttcagggccacggagcagtggtttatccgcatcacggatctgaaggaaaaaatgctcgaagagatagagaaggtcttatggattccagagtgggcgggaagcgcccgattcagggactggataagcaacgccaaggattggtgcataagcagacagcgatattggggtattccgcttccgatatggatctgcgagaagtgcggtaaaataagggttgttgggagcataagcgaagttccatggcaatccgatctcgatctgcacaggccaagaatagatgctgtgaagttcaaatgcgactgcggtggagaaatgagcagggtgaaagacgttttcgatgtatggttcgatagcggtgtcgcgagctggggtagcataggatacccgctgagaactgagaaattcgatttgtggccagcagacttcataacagagggacacgaccagacgaggggctggttctactcccagcttggtgcctcgctgatatgctttgatagagccccttacaaaactgtgctgatgcatggctttaccttggacgagcagggaaggaagatgagcaagagtcttggaaacgttgttgagcctgaagaagtgatcggccagattggagtggactgcttcagactctatgttctgagctcggcagtctgggaagacctgaagttcagctgggaggaagccagaaatgttctcagaaacataaacattctctggaatacgataaggttcgcttacacctacatgagcatagacagattcagggcaggaaaagatgccgagcttagcttagaggaccgctggatcctctcaaggctcgagaggtttaacgaggaagtcgtttcagcaatggagaagtaccagcttcacagagtcgttaaagccttttttgagttcgtaatcgaagatttcagccgctggtatatcccgctgatcagggcaagggtatgggaggaggcggaatcgaaaagaaagctttccgcatacgaaacgatgtttcgagttatagacaagacgatccgaatcattgccccattcgctccagttatcgcagaatggttctaccagaacttcattaagagctttagagacggaaaggaaagcatcttcctggaagactatccgagagccgatagaagtcttatcgactccgaacttgaggagaagatgaatttggcgagagcgattgtagaggccagtaacagcgcacgaagcaaggcgagaattaagctgagatggcccctgaaggagctgatcgttgaaacaaatgccaaaggacttgaaagcattgccgagataatagcgaagcagagcaa

>Bin11_42

ctttacaggatggggaaaatgcagaacgagatcgatggagcgataatgaagcatgcggaatggagaaaggatgcgctcgtcgaactctacttcaccctcctcaggatcctgatggagatcgaggacaagattgtgcaaagcattcagtcagactccaagcttccttagaacgctttcctttttctctatcgcctttctgaaggcaatgtcgatctttcgcttcatttcctcaaaatccgctggcatctcctctccaacgagctttttaagcggagtgtacgccgactccctgaacctcggctttatcggcatcttctgcccgccatcgatgagatatgctccctcacctttttcaacccatccaacctcctctatcttgaccttggcatctttaacggcctttttaaccctttcagcaacgctttcggggcaaacgatcattagagaatccgttgacacgcctaagaaatcgatttgaagctcttccagcattttcagaacccttggattcacgcatttcctgagcttttcgtaatcgaaaactagcgcttttcccgagaccttcgagatctcctcggcatctcccctgatccccccattcgttacatcgctcatggaatggatctcgggcaggagtcttgtggaaaatactgccctcgctgcctttatgaagtcgaggtttatcgtctcctcgacaacttcgtgcatgccgtagtagatcgcggtcgtagctatcgttccacctccagcaccttcggtgagaagtattacgtccccctctttgatgtttttcctcggggtaaggtgagttgcgacaccaacacagccaacgcagcccgtcatgcgttctccgatgaccatgtcacccccaatgcgcagcgtgcttccgctcaccagcggaacctgcgttgcttcgcaaactgttgcgatgcccgcgatgtgatcgaagatcttgctcacgtccccgtcatctgcgacgtgaatgtcagagaaaacagcaacgggttccgcgcccttgacgagcacatctctcatcgcagcccttgttacgtggaatccggcgataaaaggaaattcgctgagccttgaatgcatcccgtcaacggctacggcgatgaaaatacccctgctctccacgactccggcgtcgtcgaggtcctttgaggagagaagtgcttttacattgcatatctgaccgatctttgagtgaacgtaaaaatctccgagccctcgtgatccaaccccaaaggttcccatggtgacgccgctgcgataataatccagtataggcaaactcttcgagtaagcgtttttaacctcggcgatgatcgcttttgcccacttccttgcccttccctcatcccatccctttatctccctgatcctgaaagtcagcttcctctcaacttcatcctccctttcaatctccagcagcttcctcgcgtaaccttcgaggtccatactacccctcgcttacaagtatgactccgaggactataaatgctattcctgcgatcgtctttggactcaagggctcggataggagcaggaaggagaataaagctgtgaacatcggatagattgcagttatcggaacggttctgcttgctcctacggattcgagagcgaggtagtagaagaccatcgcaagggctccgccaattgcgccggcgatgaggagcaaaactgctgccttcgcttcaaccgccagaccccctctgaggacagaaaggataaaaagcatcgcgatcagcgctccgaatgctctgatggcgagtgcagcgtaaattggggctcctgaaagaagcgcgtatctgtcgagtatcggtgcaaatccccagagtactgcagagatcagtgcgagaatctcgcctttcatggaaggaggtcatcgaagggtataaaaatttaagattgaagctccagctcggccaccttttcccagtttctctcgtcgaagaccacaatcttggagccaacgacgtagaggaagtcgtcgatgaacagagccctgaaacccgatcccgtggcctttataagctttaacccgtccgtgtatgagaagatatagccgtttctcgctggaatgaagaagatctgatgcttttcgtcagccaggaatgccctgtgattgccgatcacctcgctataggattcagcgagcaggtatctgtcgatttcaacgggattctctggatcgctcacgtcgaagagcgacagcttaaccttgctttcctccataccgacaccgaggatcatgttctccccgagtgggtgcaggtatgaggagaagccgggtatcttgagctctcccgcaattttcggattcttagggtcggagagatcgacaacgaagaaggggtcggtctgcctgaatgtgacaatatagcctttctctccaataaatcggaccgcgtagatcctttcagtctcgccaaagccctttactgagccaatggtcctcagctgtgagtcaagaacgtagaggtcgttcgaattcccgactgtggtggcaactctcagatttccttcgtattcgtccatcgagaactggttcaaaagccttccgggaacttggccggttgcgacgatcttcatatctggcgagaccttccaaatgatggttctctcgagttccctcgcatgcctttcggtatacttctcgaacctgttgttgaattcgttttcgaataagatcctttcttcgggccccattccgagctttagtcttctcaggaggtccagtatctcgacttgttttgctctctcactcagatcgtagcttgagagctttttgagcttctctaagaaccagtctgggaaaagatcggtgttttccgatgaaaacctcagcatgagctctgagtaatcagttggagtataatatgcaacgtaaatggcgtctctcgacatgtaaacgacggaagcgtaatttccgacaaatgaaatgctttcctcgatcctaccgctttccgcgtcgagttttagaaccgtgtatatgctctccactggtaggggctttaccggatggtagatcccactgcattcaacaacgtgagagaccccgtttacacttaaaggcactatcgggcaggtttcagaatactgggataaaatgaggtatattttccctccatacagccttgccgccaagatcgagccattcagatccgctgagtatttctccttcaggtcttctgtgctgaa

>Bin11_43

ccataataatgaggggaagcatatgttcgaaaatgtcatagccgatctcctcggagttagcggggtcaggggagtgtatatcgcggactccgaggggatgctgatcgagtcggaaagtataggactggcgaacgaggaaatgtgtgcagctcttgttgtggaaatgttcaacaaagcctccgaaatcatgcaaaagcttctggcagacgaacccgaagttctaacagtcgagggaaagaaggagagggttatcgtttcaagagccggtaacttcatcataggcatcgttgcggacattaaggcgaactacggcttgctgaagatcgagctcaggaaggccgtggagaagattatgttgatggtgtagctatgatcctcccgaaaacggctctcacaaaaatagagaaagggaagcccggaaggatactcgaaaatctgatgctatcgaaattcagcggatacataagggttggatttaagatggatgaactctgctctgcagaggttctactggaaaacggaaagatccttgcagcggagatccttaggattaaaagcaagaaaagcgtctatggagacgaagttctttcagaactggctaatttggagaactctgttgttgagatctacacgctgagtcctgagcagatcaaaaaagctctcgaattgaacaagagcgcgagggtgagcgaggcaggagtgatgaggctcgaaagggataagatcattgaaaggtacgggatcgagaagcctgacgaaaaggagatcgagcaggttataaccgaagctgtgggaaattccgagctggccgtagagctcgaaaaggagaaaataatgaaaaagtacggtatcaaaaagccgagtgaggaggagatagactatctgatctcgaatgccttgggagaagaagaaatcgaggaggttgtgagcgttgacttcgggcagctgaagaaggagctcgtggacatactcagctcgaagatcggaaagccgtcgaagaagactgtgggcataatagagtcatgcggaagctatgaagagctgataaagaaggggacggaaatagagaaagcgctgagaactctcgtgatgttcttaccaagagagaagatcgaggaagtcatctcggaaatcgagcaaaaaattggtaggaaactcacttgagcttttcaagctttttctctacctccagtatatagttcctcagctccttgttcatcttcaggacttcaagaacatcctgatagttgctattatttatttcctcaattcccaaggagctcatggccttgtccaagaatatcctcgctactgggccaacttcctttttcagctcctctaagaggacttgatacgtgatcatgttcaaaatccccccgtgtgtgaaccctcctaaacttcttaaaattttcattttaaaaacctatcgctttcaaacattggcattaaaaataaaaaaaggattactttaaagcctcttcgaggctctgcatcgccaccttgaggcttctcttcagcctttcaatactgttggcaagaattcctatttcgtcggccctttctctaaatggaacttccgtatcaacttctcctgcacttatcttgtccgcagtcctgctcagctcaacgatcggatttgcgacgcccgttatcgtgaagtaagcgatgatcagtatgaatacaactgcaatcgcaaaggcgataccgaggtttacgtatatctgttctccagccttttcaatgctctttgtaacttctccggcaatgttttccgctggatttgcaggattcttcgtcaagtactggaagtagccatcaatgtaagcccccgtgcccgcaattaggatgacgcttgtattaagcgttggatcgtaaaccctaaggcttagagaaactgggatgttgcacccgtatttctcaacctccttctttgtgtcaggctcgatccacttgtaataactgcatgtaacctgcgtaaagctacccatcgaagatagcgttagattgtagagctctggcatgcttttgtcccatcccaagtcgttctttatgtccttaccccactgagtcttcggaagggtcggatagacgaccgttacaaccctcctctcagcgccagccacactgccccctaagatccatgtatactcccccatcccccatctctttgcggacaactccaagaaatctggatccgtcatcaggtccaaggtagtgagattgggattctctctgagcttctgacgaaggtaaagctctaccttctttgcaaggtcttcggttttcattttaacagcttcctgtcctgccttttcaatgtaaacctggctgacatttataactcctgtccttgcactttcaccggactgggcaactccaaccaaggtgagctgagccagaactagcaggggcacgaagcttgccaagagaactatcaaaacgatctttggtgttagcttcatctcttaatcacctccacataatcgttcacctttgctcctatcttctccctaacccaggtaggtaagactataaaaccagatttcgtgtaagggaggactctaagattcacgctgccgtctttttgaacgacaatgtagtcaccttcggaagcaccaagggttttcatgagatcctcgccgaggaaaacgacgtcgcttccgctgaagtcatacctttgtgtgaagttcaggtccactccaactctcgccctgactccggataatttagcttcggctgttttgatatcctctacagtcctgcttatctcctccttctccctctgaactttttcaacgtccagtttgaccctctccacagttttctctctgagatacttcccgatctcaattagcatcatttctggtggaagatccatcagtttctgcagaagctcatcgggaacgatgccacgatatatttccctgatctcgtagtatctgagctttttctccagctcccttatttttgcctgtagctcctcgatctttttgtccttctcctcaatgatcttcctcacatctccatccataggtaaatcctcccccttacgacaattaccatatggattaaagatataaattttctcatctgctttaggctgcgtcaaaaattaaaaattaaaataattactcaatttcaaaaccgattagcacagtcccctttccgtccccctcccagctcatgagctttttaagatagaggtctgagggcgtagaggggctgcctacaactacccctttcgcatgaggaacgcttccagtccagggcagatctaagggaggtattacgagtgtagcctccccaatatttattccaagattttttattttttctacatatagaacaaaaatatcaccgattaccgtattaccctcattcttcaaccttctgagctcttcctcgtcccttatctcctcgcctatcacttctcccctgtaaataaacttcaccgtgcctctctttggccactcaaaatcgtcatttgtaattgccgcaaccgtaaacgttctcccaattgcctccctaacaccctgatttattcccggcattaggcccatcagcgagcttttctcagccttctcttcgattctcatgaattccctcttctccgattccgttaacaatcttacatcctcaatgcctttcagagatctcaaaaattcgagaatctcctgcattttagtcttcatcatcaaacctccattttcaccctcgccctgctgaagtcgaggatcttttcagtacaggccatgacgcatctcctgcaggcagtccccagacaaaggcttgagcttatggtaaccttttcttcaccaacggacaatatcgcattctccggacagtttttaacgcattttttgcactccttgcatcccttcacatctatctcgacaagcgttcctacgtctgagacgacctcgagtcccagttttcccagatccgctatgtctctcggcacaaccttcacgacgacaggggcgccagactgagccgttattttctgccttatcccgtatgcactcagaaacctgttgtagtcctccagcttcattccctcggtccagtaggcaagttcaagtgtgtatgcattctgaaagacctcagaagtcgcaaacatcacatgctgtgccctgagctcttcagcaaagttctgcagggtgtccaaatactctggagacctcacaatatctgcagctaacgcgagcgaggtgtttcccacctggtatatcttttcagctgagggaggaatctcccccacaattctcgacttgatcgggtcaacgtaaaaacctgaagcaccagccatgtacatcgttttaatatcagaaaagcttatcccagcttcctctgcaagcgtgagaaaacctgctctaaaagctccgagtgcctttcctgcttcctcaatgtctttctcagtcagataaacaccgtcctggaagtgtagccttctgtctgaggtcagtattctcggaggctttatcaggcccttcacaagacctaccgcaattcctgcaattactcctgtccccgttatgccctttgccctgccatgcattggccccttctccagaaccctgcccttttccacgtctacaatgtctccccgctttgcgtacatctcttcgctcagcacaaagtttctccagcccatgatctcgaaagagatatcagaaatagcccccggagcagcgagcatacctttttcaatgtgctgcccctctattgcgggccccgctgcgcatgaacctgttaagatcctgtctccaactttcaaagcgatttccgcatttgtgccgtagtccgcgaccatcgcaagctcttcctcaagaaatcccgacttcaacatcatcgcaattgcatcggctccaagttcatgctttatgcagggcggaacataaaggtctacc

>Bin11_44

agtctgaatattacgcctctctcgtattctttcactatttttatagaagacgctaaaaacagcagcaaaattatggccaaaccaatccagtaccagttcatatttcgattcaccatctcatttattaagctttttgcggaagtggcacatgttcttcagaacccttactgccctttcaggagttggataaaccgggaagttattttctattctccttttcacttcgacgagcatctccaggtcctccttcatcgtgaagactatcggctttgaatacctcttgaattccccgagtaagtcgagatcaagaccaagaaggctcggaatgaatattcccgagactattgcgtcgacattttcgtcctcacagagcagcttaaggacatcgaaaagaaccatgtccccggatttctctatcagaggatatagatcgatcggattctggacggtatgccactcgggagccagttcctggagctttctcttagttctctccgagaaatccgcaattttcattccactctcttccactgcatcggcagacatcacacactcggctcccgatggctggatgacccccactctttcccccttgggtataggatttagtgcaagggaccggagggtgtctatgaattcctcgtagtcctttaacttcagaacccctacctgcttacaaacgccctcgaaaatctcatcccctttcgatatagatgccgtgtggctgagtgcgctcctctttccagcttccgttcttccagatttcagaatcactataggcttctgagcctttttaagcacctcaaaaagctttctgccatttgtgaacccctcaaggtaaatacctatgaccctagtcctttcatcatttagcagaaattcgagagaatcgatttcgtttatgtcgcatttattgccaattgcgatcagcctgctgacccccacgtggtgcaacgcgaggagactgaagttcgcaacagcaccgctttgcgagataacgcctatgcttccgctttttatattcagctgagaaataaacatcgaaaagaaagaggtgaatcttgtctctgaatcgaacacacccatcgtatttggccctattatgctcatccccttgcttctcgcgatctttactatttcgtcctcaagttccttccctttctcccatccctctgcaaaacctccgcttattacaactatccccttcacgcccttttcagcacattcttcgacgacctgaagcgcattcttcgaaggcacagcaatgatggctatgtcaatactcgctggtattgccctgaggcttggatagcatttataaccgtagatctcgccaccagcaacgtttactgggtaaaccttaccgctgaatccaagacttttcaggttgaaaacgatggtgtttcccggcttcccaggagtcctcgaggctccaacgactgcaacgcttcttggattgaagaagaagcttatgtccctcgaagtgtaatcgaagcttttcctgttacccttcacgattctcgcatcggcgacaaagcagccattctcgttcgcaaatactgggttaagatccatctcaactactccttcattctca

>Bin11_45

aaatcctcctgaatctccgtatacctcacatcctcaagatcgtcctttttctccttcagagcattcattgcttcttcgacctcgtcgagagcctttacaagcgcgattacgtatttatcgtcactttttagctccttttcaaccttttcctccgcaaactctattgcagttttcctgagtaacctgtagtaatcttttccgaaaaccttcaggccaagctcgtagacgttgaagggcaaaggcccatcgttcctcgccaaggcaaaagagctcttcagatcttccgaaacaacaacaactccatcaaactttccaaaccagaggttatagctcaatctccgcacccctgtttctcgccttcgtaacgatgctctcgcagtttatccccttctccacgaaataaccctcaagctccttggccaaagccctcgcattcgtgtcggtcggagcaaagatcgcgggcccggtggagctcattcccgcgagaaagttatcgaggcactcccttataagagtcccatatctttcgacttctactttcttgaaaccaagatgctgaactctcctcaaggctgagaggaacgaatccagatcttcttcagcaactgcaggcagcattttcatcaggatgagatggcaaagttccctgacgtcttccaacggtacaggacaacttttcttgaacatgtcaacctctgcaattccatgtgccccgctcaatctggggattgcaattacgacgtcccaatcggggaattcaagccttgaaataaccttcggcggtttcgccctgctcgctgaagatggtaaaaagtcccttttctccttcttcgagtgtcccccatcgactatgaagcctccgaattcaaaggcggcaacaccaatgcctgacgtccctcctctgccaacaagctcagctatctctcttaagctcattttaggtgcataaaaggccgaaaatgccttaccaacagctaatgcaatctgcgttccgctccccagacctacatggccgaggtagtcagacttgacaacgatttcgatccccttcccaaaatgaagcgatagtttctgagccacttctctgaatctttccaaattttgcgccttaccgcttacagagatttcttcgctttcaaatgcttctatttccacgtggggatcgtttaaagcaaaaccaacaccaccatcaacccttccaatggctccgttcatgtcgatgagcgtgacgtggattcttgaaggggttctgatccgcatggataaatttttgggaaaaagattaaaagcttaacgcagactccgcccgatgattgaagtgatagaagctctctctgagacgctatctggaagaactgttgttagcagggaagaactggagagaaaagctattcgcatggctttgagggtattaggcttaagaatgagcagctttaccgagaaagatctggaggaagttgtgtcgagccttatcgagactccaatatcggtaagatctgcccacttctcagaaaagatctcgataagcggagtcaacttttatcacctgcacacaaaaaggcccgaaagtagagagcttgaactcgcctacgccgagtacctgaagtcgaagaaattccttgaaagactgcacgataccatggtctcagcagacagtttcttcgagggatatacgcggaaagggtattttctcaggttttatacatccgttaacagatatgccgtcttcttttctacgataacggatttaatcgaagattcaggcctgcacctccagctttcttccggattcgacggagagtacgtggtgatagtccaaaccgaggatcgaccagatgagttcgtaaaattcttcaaactgcactcagaagaattcaagaagagtaatgccaaagtttgggttgcaaatccagaaaagaaaacgatagacccattcataggttatccaaaggatttcaggcttttgaaaaggttcaaaaatccaaaaatcgctacccagattcaggcgctctggcgggaaaaggtggaagaactggactaacttctcaaaagctccagggcttccttcacctctgcgttttcgtgcactatcatcgatattgccttagtcatcttcgtgggatcctcggcttggaatacgttcctacctatggcgacacctctcgccccggcatccatcgccattctcaccacttgcagcagttcttcgtcgctgctcatcttggggccaccagcaatcaccacaggcacgggacagccttcaacaacttttctaaagctatcaacatctcccgtgaagttcgtcttaacgatgtcggcaccaagttccgccccaactctcgcagccaagctgactgctttgggatcgaactgattaaccttatctcctcttggatacatcatcgcgaggagcggcattccccattcggtgcagatcctcgatatttcaccaaggcatttcagctgctctgcttcggtcttgctcccgatgttgacgtgaatgctgaccgcatctgcgccaagctttatggcttcctcaaccgtgcaaacgagcactttttcattcggatcgggcgaaaggctcgttgaaccgctgaggtgaacgatgaggccaacgtcccttccataaccgcggtgaccaaaggccacaatccccttgtgaaggatcacggcattcgctccgccctcggccaccgcattgatcgttttcgacagattcacaagtccctctatcgggcccattgagacgccatgatccatcggcacgattaccgtgttcttcgtgttcctgtttacgatgcgttcgattctgattttcttcccaatcatgctattgagtttcaaagtggcaatataaatctttccattcaaaacaatattttatccctcactctcctccctactgatcgccagctgcgtgattattcctatcacacccagcacgaaccacagcaggaagagtgaaagggaaaagaacgtggctgaaaacacgttcctcggatccaagagaaattcgtaacccgttattccagagacaagtatttctactgcagccgtaaccgcggttgctccgagaaatgctgttatcaggattaacattatcctttccaggaacaaaaccagtatcccgcaaagaacggcgaaaacgtagcagtaaagctgtttttctggcagcaaggagctaaggaacatgtaaccagctaccacgcccgtgagaaatagtcctgccctgtaaaataggccgaaagcgattgccattacgactcctgctgcaaagtaagttagaaacgagggctcgctgaaaatgaagagcgagagataatagccaagaatgactccaactgcaaaccctatagctgcgctgtaaaatctgaaaagcttatagccagtaaaaccgacgaccatgccgccgaaaacgagaacggtgacgagaaagactggattatacaacagctcgtttagccaggtgaagctgttcatagtatatgtcagtgcatcctatttataaaattttccagttaacggctcttcgactctccgtgaaccactgcagatgccacgagatgtgcaattctcaacgcctctggaatttttcccctgaaggcgttagcctcgatcatcctccctgcctcctctatgcttacgccggaaagctgcaggtaaaggtttttaaatctgtaaatctctcctgagcgctcaacaatcgcaattctctgttcgaagttatccagattttttaaagcagtgcgaaattcgtccattttcggttctcttcccataacgaccacaaccggaatgcccgttcttgcgttgatctcgaagatgtcagcgacgttgaaaccgccaaaggttattccattcaaaaatgcgcacctcagttgaccgtagaattttgacctcctcagcatcgttattatcttctccgtggaatccagcccatctaccgcgatctcctcatacatgaatccctcaacacttcttccacacataacgcagcccacgatgcagcaaaaatccttcgaaaaactgtcgtctattcccacgaccctccagcttttcataaagtttattgccactggtgcttattaagcgtatggaagacatcgtgaaattcctgcacgaagtgggcagcctaaagttaacgccgaggtctggctggttcaagatcggaattgataatcccgagagcgtcgcagagcacagcttcagaactgcgatcatagcatttattcttgcagaaaaaagcggagaaagtctcgaaaattgtctacgagccgcatttcttggactaatccacgatctgcacgagagcagaaccacagaccttcacaagatagcaagaaggtacgtgaaagtcgatgaagagaagcttgaaaacgagacgaaaagatttcaagtcgagccttctggagttgagaaatacgtcgaagatgcggacaagctcgagctcgcttttcaggctgtggagtattctgtgagtaataaatttgcgattgagtttgcaaagaatgttgaattgaaaacagaagttgcaaaagatatatatcgggctttgatgttgagaaacgaccttagatggtggagatgaagttcatctgcgacagaatgctcggaaaacttgccgtatggcttcgaatatctggctacgacaccctgtacgttggagatttcgcagctgaggacgaggacgagtttttgctgagaaacttcggtgacaggattttactgacaaaagacaaaagactgtttgaaaaagccgtgaaagccagaagaggggccttttttataacctcaaacgatgtcgcagagcagatgagggaactcaaagcgcttggaataaaattccagatcgtcatggatcgctgcagcgtctgcaacagcttgctcagaaggcccaccattgaggaggctttgaatgttttgagggaacaggggcttccagaagacatgctggaaaggtatgaactctggttctgcgaaaaatgtagaaagctgtactggatggggggacactgggtaaacatggtaaggtttttaaggaaggttgaagattgatgctatggaggttgtcatagcaaccatcgtcgccatcttggcaatagccactccctacgcgcttatgtttcgaaagattcttcgaaaaaagggttcagagtgatccgcaaggcgatggagagattcatcaaccgaaaaattgcaccttaaattttgagtgagtgggagtccaaaattctccatccgatagagttaaattcacacaaaacttagcaagaggagatgaaaaacgagaacggagagagactcataataaagaggacgggctatccttcagtgggagagattgtggtgggtactgtcacaagagttctggattttggcgcttttgtatcgctggatgagtatgaaaataaggaagggatggtgcacataagcgaggttgcttctggctggatcaaggacataagagaccacgtcaagagggggcagaaggttgtctgcaaagttttaagcgtcaatccaaagagggggcacatcgatttgtccatcaaggacgttaatgagaggcagaagagggagaagctccagcagtggaaaaacgagatggctgccttcaagtggctggagatcgtaaacgaaaaagtaaagctaagcagagaagaactgataaaaatcggcagaaaactgctgaaggagtacgaatccatttattcagcctttgaagaggtggcttacgagggttacgaggttcttgtcggccttgtaggagaggaatttgcgaaggcgatggctgagatcgcacgcgaaaacataaaaccaaagaaggtgaaggttcggggaatcttcgaggtcaggttctttcagcccgatggggttgagaggatcagaaaggtcttctctggaataaagtctccagacgatgcgaaggtggagatatcatacatcggtgcacctaagtatcggatcgttgtcgaggctgaggactacaaagttgccgagagcgttctgaagagcgttgtggataatgttctaaaagcgactaaaaagctcggtggagaggcaaagtttgtcagggaggttgcatgaagacgctgatgagaaagtgtgcgaaatgtggcacttacactttgaatgagaggtgcccgaaatgtgggggagagacgaggatgccgattccaccacggttctccccagaagacccatacggaaagtataggaggatgttaaggaaggaaaaggggttctttttgagagggtgagcagaatgataaagagggtggatgtcaggtatctgaaagatcccgagcgctgcggcttgaaggacccggtattcatagagggtctgccaggaatcggacacgtgggaaagctcgtcgcagatcatctggtcaacgagctcggggcggagaaggtggtagaaatttactcgcaccactttccacctcaggtaatggttctcgacgatggaacggtaaggattccgagaaacgaggtctacgcttggaaatcgaacgacagctcgccggatctcctgatccttgtaggcgatttccagagcataagcaacgaagggcatttcgagctggtaaatgcctacatggaggttgccaagaggttcaatgtgaagagaatatacactctcggcggctacggtctgggcaggctcgtggaggagccttacgtcgtaggagcgacaaactcgagagaactggttgaggaaatgagcaaatttggcgttaaattcgagcccggagagcctggaggagggataattggcgctgctggccttttgttgggagtagcaggacttgagaaaattgaagcagtctgcctcatgggtgtcacttcaggatacatggtcgatccaaagagcgcaaaggctgtattggagattttatcgaagattcttgggctcaaggtgtcttttgaggctttggagaagagagcaaaggagatggaaaagataattgcgcagataaaggagatgcaggaactcgctgtacagcagtggaagagcgacgaagacctgagatacttcagatgaggttctgggagatcgacttcgcgagaggggcagcagtaattctgatgctcgtgttccacctattttttgacgcatactacttcaacaagatatccctcgaagggtttttctggtacgtctttccccgcttcataggagggatgttcattttcatatctggcctaaccttcagcatcgcttacaagaacgcaaaagctgtttcaaggagaattctaaggctcgcgctcgttgccacagcaataacccttgccaccctcatcttcgctccagagagggccgtgttttttggaatcatccactttttcgccctagcatccgtatttggcatcttcttcataggaaaaccatttttgtgcctttttacaggcatacttttgattttctcgaatttcaaggtatcagagctgcgaactgaggaaccatatctgctgtggctggggataatgccatacggtttcagaacgctcgactactatccaatgattccctggtttggcatatttttgattggaatgttcttcggacactactatccgagaaaaagctctttttacagagaaaatccgataagctttcttggaaggcactccttagcagtctacctgattcagcaccctataatcgttctgctcttggaactctactacggagacataatgaggtcgctaatgacttgaagtggaattttgaaagaaaggattatctttgtcgaatgaaacgatcaagtatgtgggatgaaatttcaaagcgctatcgtagctggagccttgaccatcggtggttctactacctcactggactcgatgttaagagaagagtctgccatgcagattacatcctcgacattggctcaggcccaggcatttttgcagaagaactcggaatactgtttccggattctgaaattatctgccttgatgcttccttagaaatgtgcaagctctcccggggggtcagatgtggggcttcatttctgcccttcaaggatgaaatcttcgatatcgtaaccttcattttttcccttcatgagctcgaagtcgatttagcattggaggaggcgaagagagtgctgaaaagaaacggaatcatctacgtcgttgacctgaacagagatgcgccaaaaaccatcaagagcgcttcaaaattgattttagaaaaaatcattggcaaagactacgccgaacatcttgaaaagacctggaatgcattcaaaagctgtgaagagattgcagaaaagctgaaggcgaaggggttggaggtggagtgcgggaagacttttcaggaaattcgaatcgtagcaaaaaaattatgagacctcgcgtagcttcagcgatggtgagaacatggcacgaatttacggcgatggggatgcaagtcttgagcccttgaagggtaaaaagatctgcatagtgggctatggaagtcaggggcatgcccatgcactaaatttaagggatagcgggctcaatgttgtcgtagctttgccggaatgggacaaggaaagctggaagagagcggaagtagacggaattaaagctgtcagcctagaggacattgacggtgacctgatcgctatgctcattccggacatggttcagcctgatgtttatcgcaggttcgtgcaggggaagctgagagagggttcggcgatcttgttcgctcacggattcaacatacactacaatcaaatcgttcctcctaaaaacgtggacgtgataatggtcgctccaaaaggcccgggaccgctcgtgaggagaatgttcttggagggtaaaggggtgcctgctcttgtagctgtgtaccagaacgcaagcggaaacgcgttgcagttggccttggcctacgccaaaggaatcggagcaacaagggttggagttatagagacgactttcagggaggaaacggaaaccgatctcttcggagaacaggtcgatctatgcggtggcgttgctgagatgataaaaagctcattcgaggtgctcgtggaagctggataccagccagaagtcgcttacttcgaggttctacacgaactgaagttgatcgtggacctgatctacgaggggggcatcttcaacatgtggaaagccgtgagcgagaccgcaaagtacggtgggatgacgagggggaaaaggatcttcagcagtgccgttaaagaggagatgcggaaaatcctgagggagatacagagcggtgagttcgctagggagtggatccttgaaaacatggctggaagacctgtttacagcaaattgctcgaaatggaggcgaagcacccgatagaggcggtcggaaaggagatgagaaagctcattccctggcttaagtgaaacatcattttttgatttccacctcaatcatgccaggaatctaatcatgaggggtctggctatcggtatggctttatttttaatcaaatctcttctctttgcttctgctcaaacatggtttttgctttaaacctccagaaattcagatttttcgaagacttacgccaatgaagcagacaagatatttcatgttccaaagaatacgagggggttcagtagatttcagcaccagtcagcatatccgaaagcaagttcttcaaaaatctgcagttctgggcctgcctgaggcattcgttggaaaatatgtccgcagagaaggcaacgatgttgttctttctaactccgaataccacgttcttccccgccctgaacacgatatcaccgagcatcgctcttgaatccttgtttccgtttaaaattatctcacaaacctcctttggctctatgtgataaacgtttaaagctaagatctcgtcaactcctctgaaatactccaattttcccttcattgcgcttgccttcaggagtaatctctcggtaacaggtagtagctcatttttgagctgtattggagagaaaccgttctttctgacgaagaagatgaagatcttggtctttaaaagccttgaaattgcagaaatttcgctcgtttcgggaatttttagataaaaaaagatatcgtacgagaactccagcctgctcagctctgaaggttctgagcttaacttttcggcgtattcgacatagtagtcgtttatgaagcactcctcgagctccttgtaattgtgccattcagtggattcagcaactatgagggttgaaagcataaaacttcaaccaaacatcacaacataagctttgccaagatcggtaaccgctatcttgaggtgttttccttttctctcagttaagacgagcccgtcttcctccagttcattcagatggtagctcatccttgccctctgggccatatatctcttctgctcttcaattttgccgtcaacgagttctatcagcctgtttatcgagtccacatagccaccttcgttcacgagcaccttcaatatctttattctgtccccattgaactttttcaaaggaggggtttgcagaaaactcgcgccattttctgagctaagatatatcttgctgtttgagacctgcgcagctatgagtaacgccatgcaaagataacgatcggcgtcggttacattaaaaaggatcgtgtttcccttttcaatttccttcctgacaacttcgaggagctcccaaacaatctgataaacctttttacctccaaaatcaagtattttagactcaacgagtgagcttaatgcacggttaacagtctcaacagcgcctacggactccttattcctgtacacgatgtaagccttgtgtatcggatatccgatggacttgatgctttcaagaacagaagaaccatcagaagctattgccacgacgtgcacaacttctcccatggtttaccacaaatttttctcgtttaaaaaattttctctattagcataaacttttatcctaaaaaaccctcccccttcatttctcatggaaggttgcaaagcccaaagagatacggagttgaagtgaagaaccggttttgagtgctggaatttcgaaatgaatgcaatgtctttaattttacgcgaagaaatttgttcaaagtgattagtttaatgtttatgcccttttccgcttctaatcgagtttttaggttttcctgaggattcctgcaggaaatctgggggaaaggtcgttaaaaagaggttcgattagagaatttccaaaacctcgatgatttcagaaaatcgccgacggaaagtgactgaaatcttaccatacccgaagtttaccacatccatagctgattggatgtgctctaggtagaactgaagttaggagttgcaagccttgcgttcagaacttcagggtgaagacctaagaaggttcacatttcactcacgggcatagccggcgtaacccacacgcatattgcttagccttgtaagcgtgaaggtataaaccataaagaataagagaaaagaagaggattaataatcgcaatgcgaagggtttatgaaaatgcgatcattcatagttcgcatgtcccaaaacccgaaataagcccggagactaccacagctgagagataaaacccttttagcccttagaagttgcgttccggagtgctgagatgattgggctcgatgactttaaccttggagcttacaaagcgatctcggaaagcaagctcaagggctattttcactgaacaggcgaatctctatgtcgccgtagatctccttctgcgagatatggaatttcctcctcaggacgaggtgcagtaaagatatgtaatagtccaccaattcttccctgctacccactatcgagaaaagggttacgaaatcccttcccctgagtattctcgaaatgttctcctcaacctgcctgattctgttctccatgctctcatcgtgaggtatcgaagttattacatccatgcccagagtctcttttctcttcctgctctttctcctcttctcaatctcttcagcctttctcaattcctcgatcaaatcctttaaagtatttatcttccttatcctcctcctctgagcccttaaaatgtcctcgagtttgatttcatctatctcctcgctttcgagttcttcccactctattatttcaacatcgctttcgtcttcactcggtggtggttgaggggctattgcgtctgctttcattctgacgagaattgccgcatacagcagaacccttcccgaaattctcaaatccagcttttttgcctcctcaagcctcttcaggaatttgtccgtaacatcgattacatccacattccaagggtctatttcccccttcttcgccatgtcgatcaggatctcgattggatctccgaattcagcttgattcccgttacgatcgatgagttatcacccccaagagttatgcccacaaccgcgtcagcagcttccaccatcggttttcttagggaaacgacgatgaactgtgccgattgtgagcgtttctttatcatccttgcgaccctctcaacgttaactccatcgagaaacatgtcgacttcgtcaaaggcataaaacggggccggtttgtacatctgaatggccaagatgagtgaaagggcaacgagacttttctctcccccactcaaagcttcgagtctttgcattggcttgttcttcaatctgacccgcatgtgcagtccgctgttgaaaacgtcatcgttatcaaggaataactcgccctcaccgtccgcaagttcggccaaaatctccccaaaattcctgtttatcgcattgaatacctcgaagaagacctccctctttcttttctcgtacttctctattctctctattatttcaaccctctctttttccagaattctcttcttctccacgagctcatctctccttgctttaacgttctcgtagtcctgtattgccttcaaattcacctccccaaactttgacagctcttcctgaatttccgacagctctttattcaatgtcgggaggtctcttagcactccaggcggatcaaatccttcgaactttttgagctcttccacgatcgcggccattttctcttccagagttgaaagtctatcttcgcacctcttcagctcaaaatcagccctatttttctcttcctccaatctcctaagttccgaaagcagttcgtctctcctctctcgaagctccttcaccttttctccgagagatttctcctcctcgcgcagaacttttaatctaaaggttatttcctcgcatctcctttccccttcctcgattttaagcttcagctcaccctttctcctgacaagtagctctatttctgaattcagggtatcgaggtttttgctcagctgaagcctttggaaatccagattttctattctcttctcaaccgagatcagaatctctctgttccttgaaaattcttccttagctctttcgagctccgagagtatctctggaatttcgctgccctttaatttcccctcaatccccctcaaatccgcatcgatctccctgattctcttttcaatctcgaaaatgctgctattaaccctcttcaactcctcgatgctttgctgcttttccctgttcttcgattctatcttcctgccgatctcgccgatctggaggtcgtatcccctgattctttcctcgagaaccttgagctcccctctgagttcctgaagctttaaatttgcctcatcgagtttgaccttgagttctcttctcttctcttcaactctgccgagctcggcgagcattctctccttctccctctgaaggcgatctatctcttcggacagctctttctccctctctaagagttccttcgagatcagcaaacctctcctttctctactccctccagacatcagaccgcttttctccacaagatctccatcgagagttacgattctgatatttttgtccatcagtctcctcgcattctcgatcgtgtccaccacaactgtgtccctgagcagaaatctgaaaacaggtaggaattttttgtcgcaattcaccaaattaactgcgtagtcgatcacacccttctctctcaaaatctctctgtctaattccagcctgaaatccctgattttcctcagcggtatgaagcttgctcttccagcctgaagcatttttaggtaccttattgcctccaccgcatcgtcttcggtttcaacgacgataaactgcagcgcaccgcccattgcagattcgatcgcacttgcaaacttttcgtctacttctccaagctgtgcaacagttccgaaaatccctgcgagttctctcctctcttttgcacttagtacagcctcaacgggcttagaatagctctgcaacgccgatatctttgcccttatctttgccagttccacttcagctctctttgactcctcctcgatatctgagagctttgaacgaagggagaaaagcctcttatcatgctccagaaactcggagtttaggttcatctgctcctgttcccttctaagcaactccaccgaaatagtttcaagccttttcttatcctcatcgattttaagcgttatcttacttct

>Bin11_46

aagcgtgagtctgccgagtgcggctcttttcacgagagaagctatttttatagctttatcgctttcgatcgtttcaagctgctcctccctcttcctcttgaaaaaggaaaatcctctcttttgttcggtctctgcgagactcgttgcatagcctatggaacttattccccctcccttgagcgtgttgataacctcgctgctgtcaacaaccaattcgccaacgctttccccttccaacggctctcctgcccttgcaagcagagctattctacgaacgatttcatcgttgattttggcaaaggactccctgaggctcataccctcgaatttccaggcaccgttgtcgaaaaggatgagattgtcaacgtatttcatcagcgagatcatgctcctggcagcgtttaacgagtaaagctttccctcttcaggagctgggagaattccgaatgcataaactggctcagagtacatttccgaaatatgttttgcgagaactggtgctcctccactgcctgttcctccgccaagacctgcaattatcagaaatgcatcgagatcgtgtgtccctctctcatcaatcgcgttgagtatggtctcaatctcgtcctgcgttatcttagcgccgagcttgttgtccgttccgacgccgtgacccttaacgatggtttgcccgatgagtattcgatcctgcatgggaacgtgcttcagcccgagcaaatctgctcttgcagagtttacagcaagccatctcattcgaatgttcgatcctctcatcttttcattctcgatgaacatgtccaagatttttcctcctgcctgaccgaagcctatgacgaagaacctcattctggcacccttaaaggataatgtcgatttttatttaaattttttgcatgtagaaaagactatatttagcaactcaaacttttgccatgaaaatcgggattgtgggcgcgagtggctacactggaggggaacttctcaggcttttagtgatgcatccagaagctgaggtcattaa

>Bin11_47

atcgaactggttcctctcaaggatctcgaggagagtcgatttaagctcgctctcgacgatcttgtaaaggctttctcctatcttcttcctccttccagaaagactgttcttaagcttttcccttatttctgagacgacttcaaaggccacatcgctttcgagaagcgctatttcgagttcatccaagatctcctccactttctcggcgctgatgaccacctctccgcccgcaaatgcggagactttctctccaacgctgtctaccttatcccttaactgcttaagcttttccttcagtgctctgaacatttcccttctcttttcccctcgaaagctctgccaactccctctgaatcttagaggcttcgagggccaggcttcttagcgtgttagttgtttcagcgctcgacttctcgacctcctcaagccttttctttatgatttcaagggcgttttcgacgtccttttcgacgacaactccagaaccaatgtccacgagaacttttttgctctcccttatgtccgaatacgcgtaaattccaccaccaaggcttatgagggcgttcacctcgccctgagtgcctttcaggaattcaagtgtagagatcgccctcctgtactcatttgcaacaagctcgagctcgaccagtctcctctgcagcagctcaaaatccctctgaatctgctgcagtatagcaaccttctcctcgatcattcgcccaccttttttatctcctcaaccttgatcaggcatctctttaccttatggttgctgcctatgatagacatgagtttttccaacgccattttctcgctatttgcatttatcagcttcctgaaccttttccagccatcaacatccttaaacgatcccctaacctcgaacatagaaccacctcacggaaagagggcttcatcgatgatccccaactccagacctgtggtgtctctaccaaccaaatatcctttcgaatttgccagaatgctcgaacctaccatatcattgccaaaattcgcggtccctttcaaagcctccacacccatgagcttcgatatttttctcagttcccaatccgttgcattgggatgcacgatgccgcctttattcgttattactgcagccattccaacggtcttgattcccccaatcgttccccgggatatttctattccgaggaattcgctgatctcttcaatcgtatctgaatccaaatcgggatgagaaattcctcctctgtcattaacacagaaattgttgcccagacatgtcatcggggtgttcacgactttcacttcaaccttactcctcagtttttccagctccttgcccgtgatcccgctgcagactacgactccacttgagttgaccaccaccattgccccaaccagttctgaaccagccacggtggtcgatacgacctcgacacccagagtttccctcaaggaggattcgagcttttcgtctgggactccaattactgcgaaatcctcagagacttttgcataaagtccaatgagcggatttccgcgaactgcaagaagcttcatgcaagttcaacttcgacgataccgtcgtcgaactttaccgccctgactttaatcctcgctggagcccatttagctcctctgctccagagaaactcgttcaccttctcgttaagcttaacgttttcaggagggactttcatgtgcctgctcagaaaatttctgagatactttgtagccttcttggctctgagccacctcggatatccgacaagcttctgcctgagcctgagagaatataccctctcaatctgaatgttcgccatatccccacctcagaccttcagcttcctccttctccagtacctcctcttgggactgccgaagactctccttccagttttgatcgtaacccaaacgggtgcacgaacgttctgtttcagggctttggcgagcctcaacttaacaccgagtgtcttcttccccataccatcacttcctgacgattttcgtttccctcttcttctgtaccctcttcaaaatctcgacgagcatttcgtcggagatcttgctctgaattctaccagcctgggcaagatagacgagctgggtttcaacagcctctgcaatatcgggatgagccagtttaagtctcgcaagtctctccctcgcctcaggctccaaaattgctctcattattgcctgcttctgggcttcatactgtcttctcaactcctcctgctttctaagctcttccaactctctctgtctctggatttcgagaagttttcttctccttatctcttccagctcgtccatctgtttcacctcaatactttttaagctcaggaatcgtttttgacagctccgctttcaactcgagagcgattttatccagataagccctgccggctggagttaaaacccttccctctgaagtcctcttaacgaagcccagctgctcgagctgttgcagcgcatttcttatcaccgctccacttccctttctgaagtgcgcaggcctcactccgcgcctttttcttccgccgtaatagctcttgagcctctcgattcctacgggtccatcgatgtagatccgcctgaaaatcgatgcaagcctcgtataccaccagtcactctgctctggagctctttccctgctcacccctgttttaacgaacttcgcccactctggcggggaaactgcgtccttcaatttttctgcaaccctgcttatcagcagatccgcggggacgtcatacacactcatccctttcacctctccaaggttatcacaaatcccctaaggtcgatcacccggcatttaagtctctcagctagctccttcacaaccaaatccttttctttcctcccatcgaaattcctgagcaattttaccttcacagttcccctcttttcaagttggaaatttatttcgtttattaaactttccgttattccattcttcccgacgttgatctttaacgtttccatttcccgatccctcactctcaactaaaaaattttttcatgggaagaggggaggaatcttcagtcctacccactcttccaagccaaacatcaggttcatgttctgtatcgcctgtccactcgcacctttaaccatgttgtcaattgcagaaattgccacaactctctctccgtccgagaaaagcgctatgtcgcagaaattgctccctctaacctgagccagtttcacctcgtcctggagcctgacgaaatagcagcctctgtaaaattcctcgtagactctgtaaagctcgtctctgctcaactctcctctcagaaacacgtgggcattcgtaagaatccctctcgagccgggaaaaacctggggtgtgaaggaaattctcaggcccttctgaaatctcgaaagctcctgcagaatttcaggaaagtgcctgtgatcggttaccttgtagggcaccacagattcatgaagattcgggtaatgcgtgaagtctgaaggactctcaccagcccctgtgatgccgctcttgcaatcgaaaacaactctctcaataagctcgagcttcgcgagcggtgcgacagcaagtatcactccggttggataacagccgggatttgcaacgagtttcgctctccttatctcatccctgtgtagctccgggattccgtaaacagcttccaagtagcccgtgtgctttttgccgtatgtcttttcgtaaacctc

>Bin11_48

acgtcaacttcgtcggcggaaatccagatcagcacgcgcacacgattgccgaaatattgctgaatgtcagatcgaacattcccgtggtctggaactcgaacatgtaccacagcgaagaacttgcggatgtcatagaggacttcgtcgacgtctggcttggcgacttcaagtacgggaacaacagctgtgcactgaggtattcaaaggccccgagatacatggaagtcgtcacgagaaacttcctcagggcaaaggagaatggagagcttttgattcggcatttggtgatgccgaaccacattcactgctgcacggaaaaaatagttgactgggttgcagaaaatctcggtgaagaagtcagattcaaccttatgttccagtatcacccaacgtttaaggcctacgaatatcccgagatttcgagaaggctgacaggtgaggagatgaaaatagcctacgaaatagcctcgagaagattttccaacctcgtatgaaccttatagagttgctggataggattgagaaggccgtagaaagaagagtgctcgaaaaggaggtattcgatgcggaacttcttgcacggaagggcgaggtctcgatcttactctcctctggcataatatccgctggaagcgctgtggcctatgttgacgatgatttagttccatttggctgggtgatcgacgttagaaggtggagaaatggttttttgctcgctataaaggagttcgagagatttgaaggtgaaaagatcgtcgaagcagaaaatttgctgagtctggcattgagaaaggaagttgcgggcaaaatggacgagatcctcgacttcacttactggaggggagtgaagaaggtcgaggctcccgaatggctggacaggtggcagaaggagtgcttttcagcttcctgctcgctcgaggagggggaaatcctacttgtcataggtcctcccggaacgggaaagacgactttcatcgccgaagctgctaagaggctcgctgaagaggagagagtatggataacatccaacacgaacatagcagttgataacgtgcttgaaaagcttgaaagagggctcagaataggtcatccatcgaagcttactgaaggggccaaaaggcacagcattgaagcaaaggtcatttcgcagataaccttcaacgactacgaagagcttgcaaggaagatctcttcggcctacagggatatagcaagggctcaggaggagatgatcgagagtggaaagatagcagtgggatcaacgattctgaagggtgcgatgagcatcattcgcaacttcgagtttgacacggtcttcatcgacgaggcaagcaacacctgcatttctactgccttg

>Bin11_49

agaggataaatgagaacctcgcaactgcggttgaggaaattggtattggaatgggagttggcagtcagagagcggcaatagaagatgaagcgcttgtcgaaactttttcagtcgttcgagagagagctccgagcgcattcatctatgcaaatatcggtgctccacagctcatcgaggatcctgaaatcgcagacagggtaattgaaatgctggaagctgatgcgatcgcagtccatctcaactatcttcaggaggccgttcaggatgagggggacaagaatgcaaggggctgcttggaggcgatcgctgagatctgcaaaagcgtcaaggttccagttatcgtgaaggaaactggagcggggataagcagagaggttgcgatgaagctgaaaaaagctggtgtttcggcaatagatgttggtggaaaaggggggacctctttcagcatcgttgaagcttatagaagtgaagacgaagtccttagagacgttggattcgattttgcagaatggggtatcccgactgctttctccgttgttgactgctctgacattctgccaacaattgcaaccggaggaattagaagcggtctggaagtagcgaaaagcatagcactcggagctgagattgcaagctcagcactgccttttctttccccggcacagagaagcgcagcagcagttaagaggcagatccttcgattcctgaggggatttaagactgctctctttctcacgggctgtagaactcccgaggaattaaaaaggaaacctttatttataagcggaaaacttgcagagtggctgaagttcaggggaatagatccgagggctttttccgaggggagaaaatgaaatacgtggaggagggagcgaggatcgttaatgaggcgataaagtgttatctgcccgagaaggaccttgacctctacaaggcagcaaggcacctgataaaggctggtggaaagaggttgcgtcctgtaatagcaattgccataactgaagccataggtgaggactaccgaaggatcatgcctgcgatcgtggccattgaagctatccacaacttcacgctgatccacgacgacataatggacagagacgatatgaggagaaatgtgaagaccgtgcacacggtattcggggaggcaacagccatccttgcaggggacacgctttttgccgaggcttttaaactcctttcaa

>Bin11_50

aactcagacagcaggcggaagccctgtagaccttgaaaagacttccatagcgataacaagtgcaacaggattcaagcaggttttctacaacacaacaaacgctacggattggtgggattatgactccatagttggcgacgaagataacgtggtcgagatgaacgagaagtacaagataacaatctacgtctccaatgatggctggaatactgatataggagatctaaagccaaacgatgtcataacaatcgaagtccgcccgccaattggcgctccgctgacgataacaaagcagctgccgccgagcttccaagatataacatacgtatgaggtgatgtgaatgaagctcttcaggaatgaaaagggttttacaggccttgaagcggcgatagtgctgatagcgttcgttacagtagctgcggtgttcagctacatactgcttggagcgggcttcttcgcaacgcagaagagccaggaagtcgtgcacactggagtaaagcaggcaacgagcagcatggaaatcgttggctccatagtggcgaaagggaataccacgaataataatgtaacgaatgtgactttcacgcttcagcttgccgcaggagggcagccgatagacctgaacaagaccctgataactgtgctcgttccagtagatgggactttcaaggagctaacctaccaagccaattcttcgattttagatgataaaacagagtatctcgtaaaatggatctacgcgctgcagacaccacctgacaactacctcgaagaattcgagaaggccgaggtgactgtgtaccttgaagctgctgggttcgatgttgatcccaacgataacttcatcatcgaagtcaagccgcccataggggcagcttatccgatagagctcaaggcgccgccgagcatcgatgaagtgatggttttgctccgctgaggcctcaaagatttcaattttttaattttttgaggtggtgccaatggcggaagtggatcaatcggttgtcgatgaaatccttgcgaaagcgagtgaggacacggaggaaggaagactggagaagcttgaaagagaagtggagacgctgaagggctccatcaagaggcttctcttggacataagggaaacgatgaacaggctcgagaatccctttgcaaatctccagagcctcgccgaagctaacatagtcccgcagagtgcgccgcagatacagatagtcccagctagcattccaaaggctgaagagaaggtagaagagaccagagaagagatggaagaggaagtagaggctgaagaggagagagaagatatgggagttcaggaggaagttttgaggcccgataaagcggttgaattgaagaaggaaagtaaggagagagaatcagcaaaaattgagacattgatggataaagaagttaagattgaacctgcagaaatgtttaagggcaaggccgggaaaatggatttcacgaccctatacaacctgatggaatgggttcacggaatgatttcgaagcacagctacgaatcattcaagctcatgctcgaagttttcgaaatgacgggttacgtgagcagcgataccaaggagctgatcatgaagcttgcagatcttgtgaaagtcaacggaacaaaggatgtgctcatcgagctttacatgctgcacaaggtcttcaaccccaacgaccgttcgatggactccgatctgctctcgctgctgctgaagaagaggtagctgatgaggtattgtaatggcaaaggaggtggtgacgacatcaattctgatgatcgcggcagttgtagctgttactatcgcaataactgtaatgatgcccgctgtacgggatttagcgcattcttatacctccgtttctggaaagcttagcgacagaattcagaccgacgccgagattattttcgttagaacctcagaagtagattcttcaaccagaatcgatgtctggattaagaacctcggaaaatcgattaacgtaggtctgctggagaaaggggacatcttcatcacctccgataactattacaggcatataacgaatttcagtgtttcaattgagaacggagactcggacagctactgggagcgcggagagactgcgaagatctcattcatcgtcgatgacacgctcaccggggactacacaattacctacgttttccacaacggagtcaagatcagcgacgtcttttcgaggtgagttcgatgggctttgattcgatcgcaacggcaattctgatgtccgccgtagtcctgacagccgcatacgtgctgatgacagggaacagctaccttgcagaggaaactctcgagagctatagggagtacacgcacgcctcattacagcggttgcagtcgaaagttcagatccttgcggtgaactacgaaggaacggttctcatagcatacctaaaaaatgtaggcagcacaaaattcgaggacttcagctccttcgacgttattgtctacggaaagactgaatcgggaacatacgtttcagactaccttaaggcgaactttgaaatcgttaaagagttgataaacccgggtatcttcgatcctcaggagactgcaagatgtagcgtgagcatttcactgccggaaggtaattactctctcttgatctgcactcctaatgcgatctgcagcagttttgagttttatgtaggggggtgattttaatggggaaggatattagcagtattagtgagattgaggaggagaggaggaaaagcattctatcgagtggaaacgatgagatcgacaagagactcggcggcggaattccgctcggttccttaacgctgattgagggtgagaatgatactggaaaaagcgttctctgccagcaattcacatacggcgggctcgtgcaggggcacagaatcgcctactacaccacggagaacacgataaggagccttctgaaccagctcgagtctttgagcctggacgtttccgacttctacgcatgggggtatctgaagatattcccagtgcacctcgagggcgttgaatggaggcccgagcagatgaagagtattctcaaccttgtcgcaaatcacataaagacgataaaggaaaacgtcgccataatcgactccctgacaatgttcacgacctactcgactgaagacgatgtcctcgacttcctgacgaggctcaagaacctgtgcgaccttgggaagacaatcctgatcacgcttcaccagcatgcattcaaggaagatactctcgtcagaataaggagtgcatgcgactgccacctcttcctcaggaaggaacaggttggagatagatacgtcagcgttctcgaggtttcaaagatcaggggagcgaagaagacgacaggaaacatcgtcagctttgaagtgcatcccggatttggtctgaagataatcccgatcgcagaagcgagggcgtaaggtttatgtgataattactacaattattattaggggtgaggggaaatggccgaactcgagtttagcctgaaaccgaaggggctgaagcagggagttgtggctaatttcccctttacccccaaggagaacgaggagcatgagcatagcaatctggcttcatgcgggatttacaggcttctcccggatgagatgaagaaggaggttgagaagaaccttcaccttctcgaatacctccacatcctgccgatcgacaagatcgggattcccaaatttataaaaaagcttgaaagaaagcatggcgagattgaaaatccgaacatcatctatcctgctgacgagaacatttacatccacatatatcccgataaaaacgatgttcgcaacttctacatcccgatcgaacccaccctgcttactggagtggaggaactgctcagagaggtggaaataaggcttgttgactacatcacaggcatcgaattcgaccctcaggacaacgaggaaaagataaagatcctgaaatcggcacttgcgaagatctgcgatgtaagttcaaagagtctcgttcaggatgtaagcggagaggcgaagctgattccaaagcttaaattcggatccagagagaataaaaagcttaaagtgacggaacagcagtacaaggccttagaatacgctctgataagggacaagatcggattgggcgtgctcgagccatacatccgggacaaatacatcgaagacatttcttgcaacggcctcggcccaatattcatcgagcacaagatattcaagggtttgaaaagcgtaatagagttcagggaagaagatacgctgaacaaatttgtaatcagacttgcagaacgcattggaaagcctgtgacctacaaggaccccatcgtcgacgcaacgcttccagacggaagcagaatcaacatcgtctacggaaacgacataagcaaaaacggcagcaatttcacgataagaaagttcaacgaaacgcccttcagcgttcttcagctcatcgaattcggaagcctcgattacatgatcgcaggctatctatggcttttgataagcgagggaatgagcggtttcatctgcggagagactgcgagcggtaagacgactcttctgaacgctatcacggcctttataaggcccgaggcgaagatagtttcgatagaggacaccccggagcttcaggtccctcacaggagctggacgagagaggtgacgaggggcacgacgagaggcgggaagattggagaggggagcggaagcgatgttacgatgttcgatctgctcaaagcagcgttgaggcagaggcctaactacattctggtcggtgaaatcagaggtgtcgagggcaacatcgcctttcaggccatgcagacaggtcaccccgtgatgtcgaccttccatgcagcaactgtcgagaagctcatccagaggctgacgacagaaccgatcagcgttcccaaaacgttcatagacaacctgaacttcgttgtaatccagagcgccgttcgccgcccggacgggaagctcgtcagaagggttctcagcgttgcggaaatagttggttacaacccgcaaaaaggcggtgttagcttcatagaggtcttccagtgggatcctgtgacagacaagcacgtattcactggatacggaagctcatacctcttggagcagaaaatagcgactcgactcggaattcctccaaacaagaagaagatgatatacgaggaagttgagaagagagccaagattctcaaaaaactgcatgaacagggaatagttgacttctacgaattcttcaaaacgatatcgaagatcacgaagagcggaatactttcaatgaaggtgtgactatgttcgaaaccgcaagggctccagcatttaaattcaggttcccattcattgaaaagctgagaaagctcagggaggagatgctgtccgacaacgacctgcttttcctcctcacctacatgagctctctgtccacagcccacctcagccgggacaagatattcacgatggctgctgagacgaagtatgcgccgagcaagtatttcagaatggtccgcgatcttgcacagaaatggcattacgactacgctacagcatgcgaactcgtggcagagaagataaaggctgaaagggtgaagaaacttttcaacaggcttgcaaatgcgatctccgcgggagaacctgacagagagtttcttgaaagggaatggaaggcattcaagaccgtaagaaaggacgaatacgacagaagccttgaaagtctgaggaagtggagcgacgcttacacgtcactgctcgtctccgcatcgctgatatcggtcgtcgttctactttcggtcatcatttacagcgtgggagatccgggtcaaacgttatacgccagtgcgtttggaaccttcctcatatgcctctttggagtgatcatgatcttcaaggcggttccaaaagacagaaaggttcacagccacaggataaagtccagagagcaggcattagcatctaagttttcgatgctgctgattcccacgggaataatgctattcatcttcttggccgtggttcctctgttcacaggtaattacgaaatcgcaggatacggtttcatagctctcgggctcaccttgcttccagtgggaatactcgcaaaagtggatgacaaaaagataagcaggagggatgaggccttcacgagcttcatcagaagcctcggagcgataaaaggcggagcaggagtatctattgcagaagcaatcaggagaatagaccagaagaacctcggagagctgaaagagcttgctctggaactccagagaaggctttctttggggcttgatccgaagctatgctgggagaagttcattggggaaagcgggagctatctgatagagaagctcactccaatattcaacgacgccgtcgatcttggaggtgatgctgatctcgtaggggagatagtcagctcctcaaacctggagatggttctcctaaggctgaagagagatctgatagcctcaggattcgtaaacctgataataccgctccatctggccatggtttccctaatgctcttcatctcccagatcctctcaatattcacagaataccttggctctctgttcgcctcgcagatcggctctggcggcggggatgtattcagaaatgttccgctacaggggctaaacctcggaatcttcgcgggaattcctctcgaacttctggctgagtacactttcttcataatactctcaataactgttgcgaacgttgtcgtctcgaacatggtgaagggaggggggaaatacctttacctctattacggctcaatattattcctcctctccggaattctgatgattgtagttccccaggtcgttagatgggcgttcacattgccctcgtttgtcgaagtcgttgcgggggtgatttcttatgtatgaaatcccgttagaactgatattggggttgatttttgttgctgtagtccttcttgtgccgtacatgcccaagaagaagataaccgtgagcttcaataagctgaatcttaatttgtcgagaaagaagagggaggagaagctcaaggaaatagacaaaaagcttgaggaagtcctttcagagggtggaaaggaaaagatcgttaagcttgatgaggcaacggaagaagctgccaagaaatttgatatcaaggatgacctactttcggaaatgcaaacctcaaacgctttcaccattaccgcaaatgactccacgcaaggcattttttcggagccaaaaatggatgaattatcccttccgaaaatcgagggcctggaagaaatgggagaaatcgaaaaggaagtgaaattggaacaaaacgagcagacaggagagacaaaagtcgaattcgatgactcagacaaacttcttgaggacttagccagggaagtcgagaagaaagaggaggagcagctcgatttgcttagagacctcaagggacaaaagttcgtgcttgaagaacttgaggcagagcttaaggagattcttgagagagctaaaaggctgaaatcatgatagagttgctctttacggcattcacaatcgcaagctacgtatggcttctggataacagggagaagatactgagatttctgtgcgacgagatcggagagctcgaaatctgcagggcgatcgtggtgatcgacaacgggagggaatattacagaatgaagaagggggtaagagttctggactgcgagtatctgcgctatcagccgaagagcttcaacttcgtggaaaaggaaatgcttataactgccccgatttctgcaaactcgtctgtttatattttcctaaatcagtacgacgaagaaatcgggcagatctttcaggacctcctcagggttgttggaagggctatcgaggacctcgaggtaagaaagaggaaggaagagctgctgaaaaccgtgagggaaaatcttaagcagtttcagtatctctcggacaagttgagaaatccgctcgccgtgatctttggtgcactcgaaatgaagtatgagctcgggcttgaaaaagcctgccttatgatatccgaaagtgcggaaaagataaagagagttctggacgaactttcggagtgcgaggtccagacgataaggctgacgaggactatcttct

>Bin11_51

cgttgacaggatgaacatcgaattcattcgcagattcatcgaatatctgtgcgacgagctaagcgatctggagactgaaactgccataatgattctgaggtcgctgaggggagacgtgaactgcaatcccccagttttcggggatcttgaggtggaaaggctcacgaagtacttcgaggggctgggcttttacgaatacatttccgatattgcaagacccctcgtgaaattttactattcaaacgcagaaaaaacggagttggatgagagggaggagcagattcttgtcgcaaagtgtctacagctccgctcttggagggagatcggaggagaggagaagtacaagaccctgctcagtgccctgaaaaaggtttggggatggtttttaagcaagcaaaaaattattatttcccactcgtaaggcgggttatggttccaaaacaaagggttctcgaagtggttgagaaagagataaagccgatgctctttatggaaggcggaagcatagaggtagtcgacgtcaacgagaatgaaggcatagtcaaggttcgcctactcggagcttgcgggacgtgtccgatgtcaatgatcacgctcacagccttcgttgagaggactctgaaaagcaagatccccgatgtcaaaaaagtagttccagtatgatccacctgatcgaaccgatcaagatggctgtaatagagaatgtagcttacagtgtcaaagatgtcagtgaaaaactttttggaggcttttgcgactgtggaggaagaatgctgcagaaattctggctcaacttggagggaatgagagttcttttctctgaatgcgaaaagtgctggaaaaatcacgcctacgtcttcaattccaacaattttctgaggaaggaagaagtcagggtcatcgggaaaggagagatcttggactatctcaaaaatttccttaccgaacttgaagttgaggcccttctaaataaagcgcagaacagagcttacaagccccagcacctggcaagagtgaagaaaaagctctccgatatgaatctaccattggaagaaattttatcactgctgaaatgaggttgaagaaggatataaggatttactttgacctcgccgaaatatcctccgtatcgagaaggtatttcataatcggcttttttgacggagtgataacgatcgctggaatgctcataggcgcgtatctatcgggacacaatactccagagctggtaattcccgtgggcttcgcaacagccttggcacttggaatttcaagctcctggggtgcattcgaggcagaaaggatagagcaaaaggttcttagattgagaagggagagatccctgctcacaaaactggatgagaccatcttcgatgaagcccatagctttgcaactgccttcaattccattatccacggaatttcgccgattctcggcgcattaattctaattctgccatacgcctttctacctcctttcgaggcgctaaagtgctctatggttatctgttcggcaagcctattcgttttaggatttgcaatgggaaaaatggccaatgaaagggctattttgaacggaataaggataatgatcctcggaattctggtgctgttcttagtcctcataatgaacccgggacatgtgatatgatttttaaatcctcggaaaacgggattcatgggcagagtgtggaagttcggtgacgacatagacaccgatgctataatcccggggaagtatctggtcttcaatgagccggaagagcttgcgaagcatgccttcgagaatgtgagaaaggactttcataacaaggttaaaagaggtgatttcatcgtcgccgggaagaattttggatgcggcagcagcagggagcatgcagttcttgccttgaagggcttaggaattagggccgtgatagctaaatcctttgcgaggatattcttcaggaatgcgatcaacacaggcctgctcgtcatagaatgccccgaagtggagaggatcgaggagggtgatgagatcgacttagatatccagggaggattcatagtaaatctgactaagggagaaaggtacagaataagggaactgcctgactttctgctcaagatcgttgaatgcggagggctaattgagtactgcaggacggtgatgagatgaagctcgcagtgattcccggtgacggcatagggaaggaagtgatggatgcagccatgcttacattgggagagcttaacctgaacatagaatacgaattttacgaagcaggagacgaagctttaaaaaaatacggaaaggcgttgccagacgagacgcttgaagcttgcaggaagagttctgcagtccttttcggggccgctggggagactgcggcagatgtgattgtgagattgaggcaggagcttgacacctacgcgaacgtaaggccggcaagagctttgaatggcataaagtgtctatatcccggcctcgatattgtcgtggttcgcgaaaataccgagtgcctttataggggcctcgaattcgagctaaacgattccgctgtagcgctcagagtgataacgaggagagcttccgagaggattgcaagattcgcattcgaacttgccagaagagaagggaggaagaaggttacagcactgcataaggccaacgtgatgaaaaaaacatgcggaattttcagaaatgtttgcaggagagttgcaaaggattttgaagggatagagttcaacgagtactacatcgacgccgcatgcatgtatctagtaatgaatccctaccgttttgacgtaatagtaaccacgaacatgttcggggacatagtcagcgatctctgcgctgggctcgttggcggtctcgggatcgctccctcggctaatataggagaaaagcatgcaatattcgaacccgtgcacggtgcagcattcgatatcgctggaaaggggattgcgaacccaacagcaatgatcctcacagcgtgcatgatgctgcagcacttgggc

>Bin11_52

caacggcctgagagttggctcgggagttgcgtggatgtgcgtggttgctgcagaactttttggagtatctcaatatggtctgggatacaagatacagctagcaaggctttatcattccccggacgtggttatctcttacatgcttgccataggtattattggacttatccttgacagactttacagggcaacagaagcccgtttactgagatggcggagggggttcgtggttgactgaaattctacagatcaaaaatgttcgaaagtggttcgacggcggtgagagaaggcttctggtaatcgatggtgtcagcctcaacgttatggagggggagttcgtaagcatcctcgggcccagtggttgcggaaaaacgactctgctcagaataattgcaggattagaaaggcaagacgagggagaagttcttctcagaggaaagcccataaaggggcctggacaggacagggcaatggttttccaggactacgcacttttcccttggaggaacgttctgggaaacatcctctttggacttgaaattcgaaacattccgaaaaaagaggctttggaaaaagctaagaggttcataaagcttgtaggacttgagggcttcgagaaatcctatccccatgagctttcaggtggtatgagacagagggttgcgcttgcaagggctcttgtctgcgaaccggagatactgctgatggatgaaccgctgagcgctctcgatgcacagacgagaaacgttatgcaggcagagctggttcgtatatggaacgagacgaagaggacaatcatctacgttactcacaacatagaagaggcggtctacatggcggacagaattgcagtgctatccaaaagacctgcaaagcttctcgaactcgttgaagtcaacatggacaggccgcgagacaggttctcaaaggagttcatagacctgagggcaaagatattcaggctgatgcagggctgatcagaagtcttttatcgcagatcttcaacttttccacatgcgtcctcttaaagactgcagggctctcgtagtcgacatgacccacggagggaaagttatctgcgaagagctgctcagaaggagctgcgaagttcacgcttttgacaatcacaagacactgaagaaagaagaagttgactctttgaccaaaaaaggaataaaagtattttctgacgaaaaaggtctgaaggtcgaggaatacgacgttatcatagtccagcatgcggatccgaggatgaatcttttcagttccgctttggagcttggaattccgataataagccatgcaagggctgttggcattatactttcggaaatgaagggatccttaaagatcgttgagataaccgggacaaatggaaagacgacaaccgcaaacatggtctccaagatcctatgcgactccaacggaactgttctgatccacgacagcctgagtacaagggttttaaaggattccaaagaatttttgcttgcagaagggttgagcataacgcctgccaacgtgctaagggctttcaggctttcagaaaattacgacctcgactacgctgtattcgagatctccttaggggggacaggagcgggagatgtaggagctgttacgggaatctacgaaaactacagggcttccttcttcagaaacgcgtttaacagcaa

>Bin11_53

atcttcgttatcatcatgcccccctttattcccgcagcttcggctgggaaagttctaccgttctcattgtagagacccgttatcttcacgcctttaacccccaagaattcaacggaagaccttcctgtcccgttctccagaactgcaaagttcagttcaggtctaatcagcgattcaattttcccagttacctcgaagccattaagctccaccacccgtcctatgaagtttccgctgtcgtcaaccacgctaacagcaggattcagcaaggagagacagctgaagaatgaaaagaatgcgataaaagccacgatgaagttgtttacggcagcaatcgaaaagacccttatcctcgaaatcctcctcgtcttcttatccagaagctcgccttcatctggctccgcaaatgcccctattggaaccaacgcaagaatgagtccgagtgacttaactcttattccttcgaccctgcaaagaattgcatgtccgaactcgtggaccacaatcgccacaataagcccgatgagtccccagacaagcggaacgaatgggttaattcccggaatcagcaaggccgcctgtgggctcgtgagctcagacggttcgggaggcgatctgagcatgatgacgtctgccaagattactagcgcgagcatgaagaacattccgatgagcaccgcagaaagaccgaggtcggccgttagtctccagaatctcttcgccttggagagcttttccacggcattaagccctcttttagtcctgatcagcaaaatcggcccgtaatttctcattccgtatttttcgagagtccccgctctgccaagtacctcgagaagagtccagtaaagcagaaaaaggactaagaaccagagcattgagacgtatcaacttgaaagaataaaaggtttttcaagtgtattccctgaggatttcaacgagaatttctccacgaccgcccgttggcttcgctatcgttctaccgcatatcagacacttcacaacagtagagggatgatcgaagataacctgttcgtgctcgcagtcggggcacttaacgcgaacgaatttgctcttcttcggcatgccctcacctctctatgagctcaaacctctttgccctccacgtgggcctcgtgtgtgccttcttgcagacggtgcagcgataccttatgttcactctcttcgtcggcttgtcaccgcctggaaccttgctgaactttccgaggtttcccaccttcccccttcttctcttctgcctgttgatccacttgagcttgctcggcttacccttgcccactttctccacctcgtggagcgtgtgcttcctgcaaaccctgcagtatgtcattaccttctttggatacttcattcatttcacctcgatcctttcagcaatgccgctttttacgagcacttcggcattttcagctggtagagtagcgacatcttcccttctcaatttatatctttttccatcaactccaaggatctcaacgtccttcttgattctgacgagcatcctctcaaatttttccttccttgcctcaaaaacctttttctggaattcccttatcttctcaacgagatcccggaaaaattctctctctaaggggctcatgttttcctcaatgaaaccaacttcctgcccgcaaacctctgcccatgcggcgtttattatcttccctgttctgagctcgaaaagtctccgctgcaggcgcctcagcgttttaatctcatcctcgattctctcgttttcaccttttttttcttcaagttcctcaatcttgctccttattttctcataaacgttctcctcgatctttgttagctgggttcggtttttctcgatgagcgtcaggagctcttcaatcataagcttcgatctcttttaaagctttaaacgttacttaaattcgatgacttgtatatttcgtacattaaaattgccgccgctgccgagagattgagagaacccacttttccagttcccggaattttcccgatttcatcacaaagcttctttaatcctttggatatccccttatcctcaccaccgataaccaataccaggggaggcttcatcgaaatctttctaacgtcatttccgtcaagatcggctccaaagacgaaaaatcccatttccttgagttttctcacagcattcagcagattttcttcactcgcaatcttcacgtggaaaacggctcctgctgaagctctaacgacggcttccgtaacgttcgcactcctcctcctctttatcacaaccccagcacacccgaagaactccgcacttcttatgcatgccccgaggttctgcggatcgcttatgttgtcgagaaccaatatgaagctgttgtcgcataacgctttttttgcaagctcttcgagacttgaaatgcttaccgggctgatttctgcacagatcttctcaggtaagcttgtcctgtagaccggaacgcccatcttttttgcaagctcgattacctctcttatcttcggattctcagagctatagtataccttcaaaacccttcctgacttcagagcttccaaaacgctgttggcgccacagatcctcataggatcacggattgtagctccaccaaccggcccgggatcatgcttctcacccgggtgtgagaggtggagcaatatcagttttcatggaaattaaaaaactttgtgctaaatttaaaaacagcggagcgtatccaaaaaggtggagatcgaagcgctgaagcttgaaactccgtttgaaaagcctgatttcgtttatgtctactttctaaattctgaagtcctggtcgacggtggattctgctccgcagaacatgcagagaaaatttccgaatttgagccaaaatttgcgcttatcacccaccaccacatagaccatgtgggctacctgttcttttccgacatcgaggcctaccttcacccaatcgaaagagaactaatttacctttacgaaaagccgcacttcttcctcgatagacagctcggaatctgcgatatgtacgatgtgccgaaggaatacgtgaagacgctcgaggtcatttcccttctaaggctgaagcttcaggcgagagttcatgaattgaaggagaagttcggaagaatcaacgctatccacgttccaggccacacggctggccatatgtgcttcctaatcgacaacgcgcttttttcgggagacgcaattctcagtgacacaactccgaatctggggctttatctggactacccctatggacttgaggactatctcatggcccttcagaggctgaaaggcttaggaatagagg

>Bin11_54

agagggagctatcgcagtaacggctcaaggatcacggctttgggaaccttttcctggaaaactttcggatctgccttggtgttgggtatcgctccgtagtgcatcggaatgtaaacttcggctctgatgtcctgggcagcctttatcgcttcactcaagtccatcgtgtaagtgcctccagcgggaagcaaagcgacgtcgacagcgatcttcttcatttcagaaattctgtccgtatcccccgcgtgatatactttgacgccgtcgatgttaacgatgtatcccaggcatcctctcttatgaaatggcttgtcgatgttgtacgcgggcacggcagttatctcgaccccctttacgttagcagtatcgttttcgaccatttcaaggctttcaaatccctttacggagcaagctttcggatgcacgaccacggtgtcgccttttgccagattccctatggatttcaggtcaaggtgatcgaagtggtcgtgggtgactaagatgatatcggctttttcctgccccgcaggaacctcataggggtcgatgtagataaccctcgagcccttcagcttaaaaccagcatgcttcagccacacgatctcaacatttcgaaacttcagcgtgttcatgcacaatgatgtggattaaacttaaaaattttttcgagcccaatcgagctttgggataagcgaaaatattaaatgtcttttcaaaaagcatttagccatggcaaggacgaagaaagtgaagcaagcagcgaagttcagggcgagcgctggattgagtgttcgaagaaagtggctcgaaatcgacatcccgcagaggcagaaatacgtttgcaagaagtgtggaaagaaagccgttaagagaacaggttctggaatctgggaatgcaggagctgtggttacaagttcgcaggtggatgctatctcccctcgacgatttcagcgaagataatcgagaaggaaaagactgagactgcggtggagcaatgagctacatctgcttcatctgcggtgcagaagtggacatagatcctgagaagaagagagttcagtgcaccatatgcgggaacagaattttgagaaagccgagaccgccagctaagaagaagagggtaaaggcaatatagccttgccgcagttctctcacgtgttcagatgcagagtaataaatcccggatacgctgaaggaattgccttagtttcttcaaagccggtctccctcttaggagacatagacgagaacgggattttcaccgtaggcgagctcaagggcgaatgcgtcgctgatcgaattctggtcttccccttcggcaggggctcaacggtcgggagctacactcttttgaggctcaagaaaaggaatttagctccgaaggctataataaacagggaaacggaggcgataatagctgtaggtgctgttatagccgacattcctctggtcgacagagtcgaagaggaattttttaagatcgtgaaaacaggagaccacgttgccgttgatgcagtggggggatatgtgaaaacaagatgatagaggactacttctacgtttacgcctccgaaaggcttgaggatggttttaggtactttgtcataccgaggcgaagctccgaggagatcgaggcctttctgatgcagctttctaatgactacgaggtttctctgaaaaggagatatggcgaacttgttctcgagataaggcgacaaagacaggactacaggctaaatctgattctattcctcgcaacccttgcgaccacaacgttcttcggttccatgttctacgaagagccaagcttactgggtggtttagcattttcgggagccattttcttcgtgctcggaagccatgaactggctcactactttgcagccagaaaatggggaatgaagacttcgcttccatacttcatcccctttccctcgatcatcggcaccctcggggctgttataaagtacaggggagcgattccaaacagaaaagctctcttcgacgttggcataagcggaccgcttgcaggctgtttcgcctctatcttagtcatcctcctcggacttcagcttccatttgaagcggagggagaggaaaagatatacatcggcattccccttcttttcgaatttctcgcaaaactaattgcctttgagggcgaattcatccatccagtagcatttgcgggctgggtcggactttttgtcactttcttcaatttgctccccgtaggtcagctcgacggcggtcacgtgctcagggccattgctggagaacgcgccgagattatttcgaaggcaacccctttccttctaatgaccctctcaattttcttcggagatctctggcttttctgggcattaattctctttttcttcgcactgcaaaagcatcctaaacctcttgtggacgagggactggatggaaagcggattgcggtcggaattttcggctacctgatcttcttcctctgtttcactccagaacccttcagactatgacttcaaattcctgatcttttccacaaagctgtcaacatctctcgccatcgcctctccctcgccagcagaatgccatacgtattccactctctccgaatcgatttttagtgcctcaagtgcactcctcagcagatcaatcctgtccttggccttgaaatttccgtatctgaagtgacactccccaagcctgcagcccgccacgaggacgccgtctatgcccttcttcaaggccctgaggatccacagggggtccactcttccgctgcacggtactcttataacccttatgttcggctcgtactgcaccttcagcgttccagcgagatctaaggcgccataggcacagtaccagcaggcaaatgccaggatcagcgggtcagcattcctttcctcagctaaggcgtcaatcatggccccaattccctcattgctgaagtatcccatgtctattgcagaaactggacaggcagaaacgcatattccgcacataacgcatgaatgaggattaactgaagccttcttttcaacgctcaccgccttgaaattgcaaacgctctcgcaaattctgcatccaatgcatttttcggcatcaacgaaagcgtagaacggatctatctcgatcttttctcctaggataagctttccagccttagcagcagctaagccagcggatgctatgctgtcgtgaatatcctttggaccgcttgcacagcctgcaacgaaaattccgcgaacgttcgtctccacagggcgcagctttggatgtgcaatctcgtagaagccgtcttcgccaatgcctatgccgaggatcgttgccagatcgttttttccttccattgccgttgcgagaaccactaagtcaaattcctcctcgcaaatttctccctgaagcgtgttttcgtagctcaaaatgagattcttgttctcgttttctattatctccccaacctttcccctgacaaaccttattcctgcactttgaacttttttataaaactcctcaaacattcttccaaacgcccttatgtcgatgtaaaatatcgtgacatcgatttccgggtatctctccttgatggcatatgcatttttcaggcttgccatgcagcaaactctgctacagtacttgttcgcattctcatccctgctccccacgcagagcacgaaggcgattctctttggttgtgttgagtcagaagggcgcaaaagctttcctcttgtcggaccacttgcagaaagaagcctttcgagttctataagcgtaataacgtttctgaactttccataaccgtattcgggctttctcctcggatcgaaaagcctgtagccagtcgcaactattattgctccagcctcgatctccagtcgattcggtgtctggctgaagtcgatggccttcggcttgcaagccttctcgcacaatttgcagccaatgcagtggtcccagtcaatggccgcgtatagcggggtcgactggggaaacggaatgtagatggcctttctaacgcccacgccaaagtcgaattcgcttgccacttccacggggcaaacgctgctgcagtcgtcgatgcatcccttgcacttctcgatgtccacgaatctcggatgctgtaaaaccttagatcggaagagcgtcgtgtagggaaagagcgta

>Bin11_55

aactcgagaaattaaggagtgaggtaaactcaatgctggaggggttgatatgatcgccacactccttctctccattctgatcttttcagtttctgcgggcgagaacgttacgatacctgttgccgaacccacactcttggaggcagaagatccgtgcatgttcttctgggagagcatgagcaacgtcgcattcgttcagccgggagagcatgaattgcagatcgggctaacatgctcgcagggaataaaagtcgtgactgcgaatggatctaggcttgcagcgatctcggtctctcccgccaagccagagaacttgctgaaatatgctgtgtatcttgaagaggagctcaaaaaggttggaaggaactatacccttctgagtgaagatgtgaagaagctctctgagagggtgagagaacttgagcaggagaaagagaagcttagaagtgaaaaatatcttcttgagaatgagcttagagcatatagggagagttacgaacagcttcaggcaaggtattccgccgtaagccaggatcttgagagcaagaagagcaaactttcgcaaatggagtccgagcttaagagtctttcggagcagagcacgatttacagagcagcaactttcttcttggtctcagtcttcttgggttccttcacagctacagcaataatgctgcgcaggtcctaagggttgaagatttcttcagaattcgttgtaaaattttttccaaaactcgcgtggaggatgaagcggtaactcagatcggcctttccacccttgaaagcatctccatagcttgcatcaacgatttcacccacaaaaaacgtgtgatccccgacctcaacctcttttaccactttgcactccaaattcgcctggcattcctttatggatggcaccctgacctttctcgaatttacaagcgtgagtttaagctttgaaatcttatcctcgtttgccccgctaacgcttcctgcgagccatacgtctcttaggagctctatcgtcggaacagcaacaacgaactccttgaattcctttattagcctgttcgtatacctcctgtgtccaacagagacccccaggagctttggctcgaaggagaggggaacgacccagtctgcagtcatcacattcggcttttcgatccccgagactatgagataagtcctgagaggatacagaagttcaagcatacttctccctcttttcgtttatcagcgagatgaaatcttctggggaatccaacgattccagctcctcttttaatacaaagactacagagcttaccctcttcttcagctccttctcggtaatataagctgcattcgcattcacgatttctgatacctttcctatcaggtgggctctcctctctatctctctaacctgcttcagacccattagaacttccctctcttcaaatttcgaaattgcatcgaatggcgtatgctttactgtgtaaatcgcaattcctatctctcttagctttccaacgacttcgtcttcctcctcttcgctctctatctcctcgcccttctcgctgaagaattcggaaaaatctatttttttaactacgtccccaaatacctcttcgatcttcaccgcaatgtccacactcgcatcggctccctcctcgtagttctttaccattctcttggaaacaccgagcattctcgctgcatcgccaattgttatccccctagcctccctgatctttctgagcttctctgcatccagcttcacgtagtatccaccgggggcggagtatatatatggaagctcccctctgagatagtcgtaaaatgtggcgaggtttatgacgggaaggttgtatcgggtgtatacgacccttctttcaaggaagtcgaacttgaatctctcaccgatcacgagcgccgtagcattcaggagctttgcaattttcctcatctcttctgcagtctccattttcagagagtcgacgttgtagagaacctttatgagcaaaatctgttctccttttttggctatgatatcaaagcatcttggcttagtttcgactaactcaagcagagagtagcccttctttctcaggagcttaacgacgttactcatcagcagagtctgcattttgtatactgtagtgggagatagtatataaaaattttcgaagcaaaaagtatttttgagcctctcaccactttgttcatgctcaaagaatacgcatcaaagatccttggatcatttgacgagctgagcaggatcctcaggaaagaagagggcaatctcgtagttgaggacgatcccctgatagtcgtgataaggagaaacagaatcgaattctacgtttcaggcgaattccacggatatgttagcgaaagcgaggaagagctaagcgaaacggttagcgaggaagcaaagctctggcttcaggcattggcaaatctgcacttcaagagatttacactgaggaggtagtatggacttcccaccgtttcggccgccaagcgaggcattcagctacctgatccgcgtggttaggggctgcaactggaacaggtgccttttctgttcgatgtacaagtcaattagatttgaacagagaccgaaggaagaaattctgaaagacatagatgagattccgaggtacttcccaaaatcaaggagcgcgtttctcggcgactcgaatccgctcatacacagggaaatagtggagatcgtccgttacttgaagaccaagagaccggaaattgagaggatcacggcgtatgcgaggatcaagacgatcgccaatatgccagaagagaggctggaggcccttaagtctgccgggcttaccagacttcacatgggtctcgaaagcggagatgatgaggtcctcaaaattgttcagaaggggataactgcagaggacgccattaaggcaggcaagaaggctggaaaatacttcgagcttacctattacgttatcacgggattgggaggggcagatagaagcgaaagacatgcagtaaatactgcaaaagtgataaacgaggtgaaaccaacttttgtgagggttagaaacctcacgatcacgaatcccgagatggagggtgtggtgaaacttcttacggctgaggaacagcttgaggagctcaaaaaactgatagagaatataaaagttgagacgtattttaccacggaccacgtttcaaattaccttttcacagagcgcgggacgctttttactggagttcatggcagaattcctgacgaaaaggatgaaatgctcagaaccatagaagacaccctcgagactgtaagagccctgaaaaaatcgggattaaaagttttcacgagtaacgacatgttcaggctcggtttaataacgctttagggattgcaacttctgatgtccacctccacgacttctcctctcggtctgacctccttgaatatctgcccctcgaatctgtaaacctcggccccgctgagccagcagtcaggcggcagcccagctttcatgcatgtctggcttaagaattcctccgcatcgaatccaaactcaactgcaacctgcggaagcagaagtccagaaaatagcccttttctcacgatcaaaccgtgtcttccgatttccacaaacttcggaatctcatagggcttcgcgtttatcttctctggctccgtaagaaccgtaacctcgaccgttatttcatccatttcacttcgcctcacaggctcaaatcgaggatcgtcaaccgcagcagatattgcggactctattatcgcctcatcaagcctttttatggggtaaggaaagccgatgcatccccttaaatcatcgttctttatcagggtcgtgaaaacacctcttttttcggcgaagacgccatcgtatctgtctctaatgactttattttcttccagatacttctctattgcttttctcgcaagcctcacagctttctttccgtcctcaaggctgagcttcatgagggaaaaagtatttttaaattaataacgttgttgctccatgattcttggcgttgatatcggtggaacgaatgcggacgttgttttgcttaaggacggcgattttgagattctcggcagctttaaaacgatagagttcgacctgagtaaaataagagttgattacgatgctgttggcatcggtattgcggtttggttcaagaatggaaagcccgtgggagctccaaacttgaaaaggattccgaatttggaaacggacaagccgaaggtaatcgacaacgatgcgaattgctttgcctatttctcttcatatgttacgaaaaagaagaacgttctttcgctcactgtggggacggggattgggacgggtatcgtgatcgacggcagagtttatcgaggtgatggtcttgcgggggagctcgggcacacctttgttggggggaggagaaagtgtaaatgcgggggctacggacatctcgaggcctactttggagggtgggcaataaagaacataaaggaaaggctcgaagacgggtcaatttacgggactaagggattcaaactcttctgcatcgcggtatcgaatgcaataatgcttctgaatccggaagtggtcacctttgggggtagaattggtgggagactgagtgaagatgttctggcagaggaaatatcgaagctgattccgggagtttttatgcccgagatcagggtaataaatgacgattacgcggtcgccaagggagcatctctgctggctagggattcactcaaaaaatagctcacgatattcggaagacttgaaaccctctctctgcatcagatcgagggaaactatgaactcatcttctgcctccctttttgtgcccaccaccaagtatttttcaccctctatcagctccagatctcctttcgcaaggaccctctctccccttagcgcctgtcctgcaaacgtgtgcgtgaaacacagaattgctttaacttcagggtgtttgaggggataataggcaggatagtcgaaggtgtaaaggtcgtcaacgacttttgcggtaatcgttgcctttccaagcttgattccagcgtcctcgggtactccgttgtctatttctccatatcccctcacgtagagaaggtcgaaataagtgcttccgatgtatgccctgtggaactttctcctttcgtgtgccacgaagagttcgtagggaatgggaaccctcctctttcgatatatgaagtcccatgtctcctcgtcaaggtccgaaagtgtgccagtttcaataccccttctcagcttctccctcgctaagaaccagtatttcccatacactatgaagtccacatcggaatcctcgctttgcaaccctatcagcctcgatcccgttacacccatttcgttcatcgggatcgtgctgaagaaattcacaaccttcgcgacagtcgagtccatcgcatccttgagcctttcttctggcttgaagatctcttcaacttcttctactggaactctgaaaatcccgtgctcgtagtatttctgagcaatcggatgcgctaaggcctcttcatgcgatagcttcttgaacctctttccataccttaacctgtcccccttttcgtgcggtgagtatcgaagaaagcacttcactttttcctcgtttctgtaaccaagaacagagaaaaagtgctctccaacccttaagaaatccctaaggcgaataggtagcatatactctttttatgtcctttgcaaaagccttttccccctctcttctgagcactctcccatcgatgagcttaatcctcgttatgacgaacttcctgccgtcaaattcgatctcctcaccgaccatgaattctgtctccccatcgaaaagaaccttgtaaggggttgtgaccgacctcttgtgcaggctgaatttgacgtagacttctcccgtttccttcagccagactgcctgagcatcctttgccctggcaaattctacagttctaccatcctttagctgaattgccgttattcttcccagcctgctttcagcaccgaaatcaacgacgagttcagaacctttaaccagttcttccccctccgaaagctttaccgttccgatgacagtttccctgtccctgctgaaaattgccttcaactccacctctttttctggaacctggtgaacatgagtcccgcaaatcagacacttgtaaagaccactcttgactatctcgtgctctgtaagctccctgcagttatcgcagtaaatttcaactctcacaggaaaaatagcacggccatgtttaaaaaaacttctttggggagagcaagatttaaaacccaccgccaatttttggcgtgcagaggaggaggctgagaaggaaggaatccaaagagatagcgagggaggttttcgagaactccggggtaaatgtagacggggagatggatctgcttgattttggggagataaaaataatcttggtcgacaacgaccctctactactcgaatacggcggaaggcactacctttcagtttacggtgcgataaggctgaagcccgagaagtacagagtgaccgtagaccccggggctatgaacttcattttgaatggcgctgacgtgatgaagccgggaattgtctttgcggacagccggatcagggagggagatttcgtatacgtcacggttgagggaagggaaagtccgatagcagttggacttgctctctgcagcgctgaagaaatgaagggcaagggaaaggctgtgaaaaacctgcaccacttaagggacaggatctgggattacctgataagcagcaggctgataaaaacttaggcgctttttcgagcaaaagccttctgctcgtgtgttggagagataacctttaaatcttttaaccgttaagtgacttcatggcactcggatttgtcctcataaagatcgctccaggtaaagaaaagagagtttacgacagaattgcgaacataaaggaagtcgaagagctctatcctctctttggggaatacgatcttattgccaagatcgtcgtgaaggactttgaagagctgagcgacatagtggttaacaagataagggccattgagggtgttttggaaacgaaaaccctgacgggggcaaagttctgatgctccgcgggctcagaattgttgccgagaacaagataggggttctgagggacctgacgaagatcatcgcagacgaaggaggaaacgttcagtatgcgcagagctttccacttcgttttggcgagcacaggggaaaggcactgatatacttcgagatcgagggtggcgaattcgaaagaatgctcgagaaaatcaaaaagcttgattacatagtcgagatcgaggaagaaaagcctttcgagcaggtttacggcaaaagagtgataattctcggcggcggtgcactcgtttcgcaggtggcgatcggtgcgataagcgaggcagatcggcataatctacgcggtgaaaggatcagcgttgatacgatgcccatagttggcgaagaggaaattgcggaagccgtaaaagctgtttcaaggcttcacagggcagaagtcctcgttctcgctggaggactcatgggtggcaggatttcggaagaggttaagaagttgaggaagagcggaatcagggtcatatcgctgagcatgttcggaagtgtgccggaagtgagtgacctcgttgtgagcgaccccgtcatggccggaacgcttgccgtgatgcacataagcgagagggcgcagtttgatatcgacagggttaggggaagaagggtttaaacgaacatgtgctttggaggctgctttgaggaaatttttagcttttccatcacgttcagaaagtctgaagtgtctataaattcccttccttctgctataaccttgtgaagcgatgccttgatcatcttctccacgagatcccttccgctcaacccttcggaaagctctgcaatcacctcaaggttcgccctgacctctaagggtagctcctttacattgttcctgaggatctgaagtctgtcttcgaagctcggcagcctgaactcgatttcctcttcaaatctgctccttatggagtaatcaagaagctctattctattcgttgctgcaatcgtgcagataccttcgttgctctgcattccatcgagctccgtaagaagggcattgactatttcagatacgtcgcctcggagatcctgatagctcctgtccagggctattgcgtcgaactcgtcgagaaagactatgcacggcatcagttctctcgccttatcgtataactcgtggatcttcctcgccccgtcaccaacgtgctcacctatgagcttcgttgacttcacggaaatgaagggcgtatttgcctcatttgccaaagcctttgcaagcatcgtctttcccgtgcccggaggtccgtagaaaagaacgttctttggagcccactttccaaactttttgggattcttgagatactcgagaattaccctcgctttcctcttcgcttcctcctgcccaacgacatcggaaagccgaatattcttcacgagctccgtttctatgacgcgcgtttcacattcaacgatgatcttggtcgaatcggaaatatagcctgaaggaggaacagcctcgacaacttcaaaggcgaaatcggggaacatcttcatatcaaacatcagctttcccttgtatacgatttcacccctccactggtcctttgcgtagacgttaaagaccctcggattgtctaccttcgggtactcgacgaagctcgccttgagcggatatccgaggggacgcaggataacgtatttcagctcaacctccttttctgccatttgatcttagtattcccaaacgctttaataagtttttctcgccagtaagcttgaaatacctcatgaaaaatagattcgattcacggaaccaagattgcggaagaaagtttcgaacagttattttaccttttctcctggcttgacatctttctctggcgttaatagcacaggccttccgtcgacatcggcagcaagcaccatccccctgctttcgactcccatcagttttgcgggcttcaggtttgcaagaacgacgacaagctttccatttaactcttcaggggtgtagctttcagcaactcctgcgacgatctgccttttttcatccccgatatcgacgatgagcttcatgagcttcttgctgccctctatcctctgggcggagattatcctgccgaccctcagatccagcttcttgaaatcttccagagatatttcggcctctttcttgcccgaaaatctttctgaaagcagcttcttcaggggctctatgtcttcgctttctatcttcttaaacggcacttcgggctttttaagctctatcttttcgtctactctcagcacatcatccagagagcaatttttcagctcaagagccatcgccgcagcaaccttttccatgcttctcggcatgatcggataccccaggatgactaaggccttagcgatctgaaggcaattcgcaacaaccttcatagccttttctttatcggacttcacgagctcccaggccttcgaattctggaagtaaacgtttccaaagcttgcgagttccatcaccgcatcgcttgcagtcttgaactcccatttctttatcgaatttgcgatcttttccttcgtttccctgatcctctcgaggatttctccatcaacctccattttcaggcttccaaagttcttccaggcaaaggaaagaattctgaaaaggaagttgcccagcgtcgctatgatttcgttgttcaccttctccctgaaaatatcccatgagaagttcatatccctgtcgtggctcgtgtagttgacaatgtagtatctcaaatagtccggattgaatcccgctttcagatactcatcctctacccagatgacgtatcctctgcttttcgagaatgcctttccctcgatctttaccattccactggcaacaactgctgacggcagggagtaatttgcccctttaagcattgcaggccagaatatgcagtgatgatacgctatgtccagacctatgaaatgcacgatctccgcttccccttcaagccagatcttcttccattcatccgtcgctctctctgtaaagcttatgtaccctattggtgcatcgacccaaacgtagagaacaagagtttcgtcgagaggaaatctgacgccccactccagatttctcgttatgcaccagtctttgagatttccgatccactcccttgcgtaattcagtgcattttcggttcccgaaagattcgaaaggtattctttgagaaaatcctggaatgccgttagcttgaagaagtaatgcgtttgtgtcctgaaaactgctctattcccgcagatcttgcacttcggctctaagatctcacccggctcgagatgcctgccgcagccctgatcgcattcgtcccctctcgctggagccttgcagtaggggcaaaccccttccacgtatctgtcgggcaggaacatctcgcagttctcgcagtaagcaagctctatttcctttctgtatatgtaaccgttttctacaagactttttacgaattctctcgttctttcatggtgatacctttccgtggtctttccgaagaaatcgaagtttatgtccagcgccctgaaaacccgctggaagtgctcgtgatacttattcacaagctcctctggcttcagaccctctttctcagcattaacgactattggtgttccatggcagtcgcttccgcagataaagatgaccttctcacccatcaat

>Bin11_56

ccgagtccgattccctgaactcgataccgcgccaaaggtccgaaagcagggaaagcttgtccacaatcacagcagatctgttcttgaacctaaatccattggcgaagatgtttttaggtcttacaagaacgtattcgtcaagatctacgtttatcttcgaatacaattcctttgcctcgtcgtaaatgaatccccatgcacatctacagtccggtgcccttctcctatcgtagatgttgacctttagccctttttttgacaaaagaaaatatagaaaggaacccgcaacccctgccccatagatctggatcataaagagtgcaggttctccacaacagcctctgcaaattcattggttcccacaagctttccaccgatctgcctgtgaaggtcgtaggtcacgatcccttgctggatcgtcagctccaccgctctctttatcatctcgctcgccttgttccagccaatgtactcgaacatcagggcaccggataagatctccgctgtgggatttgccttgttctgcctcgcatacttcggagcagagccgtgaatcggctcgaataccgccataccgtcaccaatatcgcttcccggggctattccgagtccgccaacgagtgcggctgcagcatcactgagatagtccccatttaggtttggaagagctataacatcgtattcatcgcttctcagaataatttgctggaacatgttgtcagcgatacgatccttcacaacgatttttccctctggctgtcttccgccgtatttgctgatgagttcatcctcggtaatgcatttttccccgaattcgtctctcgcaacctcgtagccccagtctttgaaggcgccctcggtgaacttcatgatgtttcctttgtgcacgagggtaacgcttctccttttgttttcgattgcataccttatcgccattcttacaagcctcttcgttgcaaactcgctgatcggcttaacccctattccagagtctctcctgactgtgacgccaaattccttctcgaggatctctatgaacttaagcgcttctttgctccctttcggccattctatgccagcatacacgtcttcggtgctctccctgaatatgacgaaattgaccttttcaggctccttgatcggggaagggattcccttcagatagaaaacagggcgaacgttggcgtaaagatccaaaacctgccttatcgtcacgttcaggcttctgtatcctcctcctactggagttgtaagcggaccctttaacgcgaccctgaattcccttatcgcattcagcgtatcctcgggcaagggattcccgtagatcttggatgcctcttcacccgcatagacctcaaaccaaacgactttctttccaaagagctctgcagccctgtccaaaacttttatggctgctggaaccacgtcgaatccgattccgtctcctttgaaaaacggtattatcggatcatcaggcactatcagttttccatccctgtattcgattttatttccgtcttcgggcggtttgagcttctgatacatgaaatccactcgaagctaagaataaaagcttttcagaattagacgaactcagagatggataaaactatttaaatccagccaattctcaattatgcttgaagtgttaaaattgaatgcagaatggctacaagagctgctcaaactcgaagaacttaagactttcgccgtgcttcacggctgcctgaagctcggactcttcgagttcctcgaaaaaccgagatcagccgaggagatctccaaaaaggtaaaactgaactacagagctgcagaagctgtgtgcagatttctgagctcaaagggacttctaatcgaagaaggaggaaaatacaggatttctgagatttcgaaagtattcctgagcaggaggtctccattctcgatcgcagaagttttcgaggagaagcgagaagagatcgagttctggctcaa

>Bin11_57

agagcagcagatagcagagttcggaatagaagattgcgagcagaaggattgtgcctgcaatggcgagatattttcctccaattctcggatacctgatgtcggaaaccatgaggattgaaaggatgagcgatgctgaggcaagaacttcgatgggcatttttagcctcaaaagagaggtaatggcaattgcgctcacactcgttggaattccgatgaattcagggcagttcagcacgttgaaccttgcgagtcttaaaatggaaaaggctaagagcagagatgaggaggcaaatagcgaggcatctcggatgaacagagctatcgcaggaaagagtccgaaagatatcgcatctgcaagcgagtcgagctctcgtcctattttgctcttctcggtctttgatgctacccagccatcaaggctatctgtaattaaagcaatataaaaaaacgtgaagcttttttcgaaatcctgaatagcatagactactgcagagaatccaaataaggcattaaggacactcagggagtctgcaagactaacttcccggaatatcttcatcctttaccgccaccgtttctccagcctttactttttctccgagctttctgacgaatctgaacccctctggaacttctagcacaactcgcgaaccgaatcggatgatgccaattctgtcgcctttattcaacttctccccttcttttacgtagcacacaatccttctggcaaatattcccgctatctggataaccctgaaaattccatcctccgtctctatcactatctcgttcatcctggggtctcttatccttttgaaggccggcaaaactctcccctctctgaattcggtcttcaatactctgccgctcactggagatctgttaacgtggcaatcaaacggagacatgaagatttccatcctgcctttttcgatgtggtcgatctttccgtcggcgggagagacaactcctcctccgatcctcctttcagggtctctgaaaaaatatgctgtgaaagctgcaaagaagagcagtattggggacagcggaatgaataggattagcgagacgaggaaaaggaccaagattaggcagagtatcttcagaccgctccgttcaatcacctttaacaccccttattatttcctcctttagggaataaattagttccgtgagctctggaagaattcggaatacagcataggcaatggctaaaagggcgatgaaggaaaagaactttgcaacgacgtggtgcgctatataaaagctgttctctccgaataaagagtatgaatggcttacgataacgaaaacgttcctgaaaaggttgagaaagtagatcgttggaacggaaattgcgaacgcctttagctttatcctcgtctcagcatttatcccgagggttgcaccggcgaatagggccatgctctctatggctgtacaggccagaattatctccactgagcttccattaagctccaaaattcttccatgagctgtaatggagtatccaagcgtttttccaagtgccgccgagagaaatgctgtggtctcgatgatcgcatcgttaagcggggatatgaatacgaatgggaagtagaagatgcaggcaagtgcggaaaaggctgtaacctcttggaacgttggggagttcctcgagataattgccttggccattaaaatgaaaaatagtgttccaagacagaaaatgcaaaagttcacgtaatcattggcttgaaggaattcaaatgcctttaaaaggcaggcaaaagaaaaaaggagccacgcaaaagccccgatttctgctctttttcttataacgtagagcagcatcggaacaacgctcagagaggctatggattccggtaacattaccgcgcctttctcggtcaggattttaagctttgccaaaaattttaaaaatcctgatttcggataagataggatggaaagtgccaagttggtggagcttttgctaactgcagagatcttcaacagatatgagaacctcagcgagagcgacataccaagggagataagaaaggccctttacaccaaggggttgctgaggagaccaataatcgtgaaggaggaaaacgtggtgagatattctggcaaggtcgtcaactccttgccattcgtcgacttcaatccgatgacgaagcagttcaggataacctctttcgagcttgcggttaaatggctcgcttcgaagggcattgagctgataaggagaaatcccgtgcttgcatactactacgagaactttgactcattggatgtttcatacgaggaggcgaaaaaggctaatccaccggcccacggcgacaaggagtggcttactagcgtgattgcggagctcagcaaggctgaggatacgaaggaaatgctcgatctcgtgagaatattttctccagaggaaataaacgtcgatttcgactcgatagccctcagcgaggagcagatcgaggaggtgcggaagatagagatcgccctaagcgagaaagagtatctgagaaagattggccttgtcgacataggcaagatcctctttatcggacctcccggaaccggaaaaacgaccacagcgagggctttgagcaggaagctctaccttccactgcttgaagtgaagctttcgatgattacaagccagtatctcggagaaacatccaagaacatcgaaaaggtttttgaaatcgccaagaaactgagtccctgcatcctcttcattgacgaattcgactacgttgcgaagatgaggacgagtgatgagcatgctgcgataaagagagctgtaaatacactgctgaaagccatcgacgacataaacctcgtaaacgatggagttcttctcatcgcagccaccaaccacccatcaattcttgatctggcggtttggagaagatttgacaaggttcttgagtttccagaacccgaagagaggttgaggaggaagatattcgagatcttcctagaaaaagtcgagggcgaattcaacatagacgagctcgtcaaagaaaccgatggtttcacgggggcagacataaagctcgtggtaagggaagctgtgctcaaggccttgcttgagggacgtaagaggataacacaggaagatctgattctcgcgatagaggaaataaagagcaggttcaggataagagttcgtgaagcatgatggtcaggttgcttggcacaggagattctcctggcactcctgtgttgaactgccactgcaagacatgcgaggatgcgagaagaaatgggtgggaaaggaagaggttttcgattctcgttaaaaacgcaggtaagataattctgatagacacatccccagatctgagaaggcagctgctcgacaacgacatagaaagggttgatgcggtggtctggactcatccgcatttcgaccacttcggtggctttagcgagttttacagggtgcagggaaacgttgaggtcttcaccaccccgcagatccacgaaagcatcggaaaatacctgcacttcctcagctacaggaggagggaagtcgacgtttacgagaagtttcagatttccgggatttccttcacccttttccccgtcaatcatcccccggtcgacgcggttggtgtaaagcttgagtggaatggctacagggttgtgattacaggagatacgaacattgaaattcctgaaaggagcgttgaggagatgaataaaccagatctcttcatagccgacgcaattgccccgagcggaaaattcaagaagcacatgaacgccaaagaagctctatctctggcagaacgggtcggggcaagaagggtagtccttacgcacctgggccatttcttcccacctccaaagattgcagttcgccattatcctgttggcgaggattaccagactttctctttcagagaagggactctcgatgaattccttggggattgacataggtggtgcaaacctgaaggtttccgatggtacggagcacaggataatttattttccgatgtggaagagggcccgggagcttaaaaacaagctaagagagcttgcaatggaatttggggcagatagagcaggagtcgtaataactgccgagcttgcagatgtcttcaaaagcaagaaggagggtatacagtacatcgcggggatttgcagcgaggtatttgcagaggtcctctttctcgatgttgagggttgcctgaagaaggaaatagacgatcccttgaaattctgtgcgagcaactggatggcctcagtctcgtttcttgtgaatgatggctttaaagacttcctttttgtcgacattggctccactacaaccgatttgattcctgtgaaggataggcccaccgcaggaaaaacggattttgagaggctgaaaaggggcgagctaatctacatgggagtcctcaggacacctgtattctacgtgctgaagcgctttgaaggcgctgatctctgtccagaatattttgcaatcactgcagacgttttcagagttacgggcgacataggagaagaggattacaactgcgaaacgccagattcagcaggaaaaagtgttgaggagtgcatgcgcagg

>Bin11_58

gagctcgagtccacctggaacgaaatcgcacgcgttcatcttaattaggatcggaattctgctcttctccctcgccctttcgattatttcgaggacaactctgacaccgtccccgtactcgtcgttccttctatttgtatgcggggacagaaattcgctcagtagatatccgtgggcgcagtgaagctgaaccgcatcgaaacctgctttttctgcccttataacagcgtttttaaagtcctcgattattttctcgatctcctctttgctcagttctcttcggcatcacgtttattgagggatcgaaaaccgcagaaggggctacaggcgggcatacggttgtttgccttcccgcatgtgcgatctgggcaacgaagaagacatccctatcaacactcttcacatcagaaacgagctttttaagtccctcaacgaactcatccctgcttattcccgtcatctttggcattgcccttccatcatccctgacatacatgaatccagtgatgatcatccccgctccaccatctgcgagccttttgtagagctctattaactcatctgttacgaaaccattttcagccatcccctcagctgttgctgatctgacaatcctattcttgagctttaaatcgccgttttcgaagggggtgaaaatcctcacagaaatctgcctcgaataaaaattttaaaaaattcagctcctaaacttctgaagcttcttcaaaagctcaagatccccctgcgcatctccggcgcaggttgcgattattccgtcaacgattccctcaaaattggccgcgaccttttcaacttcatccatcttcgccgtcgtgggccatccaatcgccttcagaatctccgcattcttcggagttcccacgaggaagtaggcatatatgggctttttcagctttctgcactccttagcgacctcttcgagcattggcatcactttttcttctgtcctcatgaagaatatgaagtcccatggttcctggactctctttataagttcttctgtggactttctggc

>Bin11_59

tgcagtaggggcagactttcctgtcaacgtatttaacgaagattcttgccagcttgttccccttccttgcgcgggtgcagacaggacatttttccctgcaaaaatttgcttttttgatcaactcattttcaggcccttccatatgccttttgaccagagaaaacagaaatagttatcgcttcgagcctgaaaccgattcagtaaagggaaagttccagaccgaatttgtccgcaaaatcgtaaccatcataccacttctgcccggaatccagacggatgattgcgaaatttgcctgcccataggggtttgcgcaggaaccaactgttattaccattccgttctgttcaaccctgagaaccttgtgaggctcgtgggccctcaccacaagccttaccccaagcttttcgaatagatttctggtcgcctttttgccgaagaagaacatcactcccctcttaaaattctccccgcactcctctctctcccagggatcgttccagaggatctccaatgccagatcatcagaaggattgcttatacccctaagctcactcctgcacggaacgcccccgtgggcaagccagatttcgccgtttaagatcgcagaatagggcattctatcccagagatcgaccaaggagcggtagatctcctcaccgtcttcataggagctcagctgccatggtagttcgtggggataaacatcttcggtttcatgatttcccctcaaaagaactgccttatcctctaggaacatcttcagaatcctttcgtaaacctccaacggctcttcgcccctgtcagcgtaatctccaagaaatatggtctttccgtaaattttctcctctatgagctcgaatgcctcaatgtctgcgtgaacgtcgccgacgatcgtgcatttatcctcgttcagctccagcattttccccttcatcagcttcttcgcctcatcgatcatctcaaggatgtccatgtggatagtatgcgtatcaatccttaaataccttttagggaactccgtagagatgaagcatctgatctcgatcgatgatctaagcagggaagaaatcgttaaaattctggataaagccgaggagtttgaggatgttgcgcttggcctgagaagctgcaaaatcctcgagggaaagattctggctaacctatttttcgagccttcaacaaggacgagaatcagttttgaaactgcaatgaagagactgggaggagaagtagtgaacgtttccgcgcaggaagcgagcagcatggccaagggagagacgttagccgatacgataagggtcatctccaactactgcgacgctatagtaatccgtcacttcttagagggtgctgcgaggttcgcggcggagaattcgtcggttccagttattaatgcgggtgatggtgctgggcagcatccgacgcaaacccttctggacctttacaccataaggaaggaatgcagaagacttgaagggctgacaatagccttagttggcgatctgaagtattccagaacgatccattccctcataaaagcactaaagctattcaaaaacaaaatctatctcgtaagtccagaggttttaagcttgccagaggagtttcttgaagaaatcgatggaaatgccgaaaaggcaaaactcgaggatatcatagaatcagtggatgtcctttacgtaacgagaatacagaaggagaggtttctggacgaggaagagtacagaagggttgctggcagctacaggataactcctgagatggtcagcagaatgaaagatagcgcgataatcctccatccacttccgagagttgacgaaatcgatgtaagggtagactcaacgaggcatgcaaggtacttcaagcaggctttctacggggttcctgtcaggatggcgatcctctcagaggtgatgctgtgaaggaactcgtaataagcaagatcagggagggaacagtgatagaccacattcccgccgggaaggcaattttagtcctgaaaattcttggaataaatgaaaggacaaaggaaacggtttccatagctctgaacgttccgagcaaaaagatggggaaaaaggacatcgtgaaggttgagaatctcttcataaaggaagaggagctgaacaaaatcgcccttatcgctccaaatgcgaccataaacctggtaaaggactacgaaattgagaagaagttcaaggtaagacttcctgaggtcgtgaagggaatactgcgatgcccgaacagaatgtgcatttcgaacagcagggagccaatctcttcgatcttctacgtcagggctgaagggaacgaggtttttgcgaaatgccattactgtggaaggaaaattaaagagcttgcagactatctcatatagcatgaaactcgtcatctgctccagaaaggatcccgcatctgtaaacatagcgtctcacctcctgacaatgggctctttcgaaaaaagaaggcttggagactatgtattccaagttgaaaaggatttcgcaattgcagaggttgatgaaaggctgatatatgcagataagatcgacgaaaggctttcaaaattcttcaagttcgacgagatcctgttcgcatcgaggcacagcagcaaggacggaaggaagatcttcacaacgcacgtctccggaaacgttgcggagaacacttacggcggcctgccaaaaagcctggcaaaaccatctccgatcacgatgaaaaactttgctctggcattgaaaaagagaatgagcagaattccagacttcgagttcacccttgaagcaacacatcatggccctacggagatctcgaagccctcggctttttacgagatcggctcaagcgaagatgaatgggaaaacggagaagcgggagaaatagttgcggaggctatatttgaggcaataagagatacgaggagggaatggaagatcgcagtaggtgtgggaggcacgcattacgttcccaggcagacagagataattcttgagacccccttcacattcggacacaacttcgcaaagtacaccttcgagaatctcgatgtcgaaatgctcaaaaaagccgtcgagctgagtgaagcggaattcctgatatacgacgacaagtcaacaaatgcgaaggtcaaa

>Bin11_60

taccatgcagttcttggaaaggcatgaaagagatgaggatattttcgcctattttgaaagacacggatatgcttgggcttttccccttggcgggaaatggcacataggcgctggcagcgcgttcgaagaaaaagtgccaggattaataaagaagcttagagctctcttcgattttcctgaaagagagccagaatgcagctgcaaagctgaagtcagaatgcttcccccatccagatgcagaccctttatatgcgggaaagttgtcggcgtaggcgaggcaataggttgcgtcagcggatttggagagggaaacgttccggcacttaagtccgctgaaattctatctgaatgcctggataaactggaaatttatgaaagaaggatattgagggagttcggatggttagaaagggagcagagatttgtgggcagctttctcaggggtaagcctgccaaatatcttttgccgtctataatagcctttgaaaggaaaagaatagccaaaatatcgctcctcgactttttgagggcaatgctatgagcttcaaaaggttcaggttttcatacaaggagacagcagttacgatcctcgcagaggatgagagtttttttgaaacagccgtaaaagcaatacttagggcaagaaatgagattgaagtttacgtgaagcttaatccatactttctaataagttacgagccgattgggtgcagaaattgcagaataggaggaattgtagaggaaatgtgcaaggcagcccgtttggctaacgttggacccatggcatccgttgctggggcaatagcacagtttgccgtagataaaatggttgaatccggggctaaaattgcagtcatcgacaacggaggagacattgcgatccattcggacagggaactcagaatcggtatctacccctcgaaaattgccttgttaatccccccttcagatagaatcgcagtctgcacctcaagcggaaaaatagggccctccgttagctttggtttggcagattcggcgacggttatcgcagaaaatgccgcaatcgctgacgccttcgcaactgccctcgggaaccaaataagagatttcggaaaagaggagcttgaaaattgcgttgcggagttttactcaaaaaacaggaattatataaaagcgttgctcgtcgtaaaggacgatctaatcgcatttgcgggcaaggttcctgaaattctgcctgccgagtttgatgagggtctcattactcgaatctgaaccagtattttgtagcgtaaaatttaaatttgcatcgtgccataagcttattctatggcggtgaaaatcatattctgtccttcttgtggggacgaagtcgaaatcagggatctttacgaaggtgtggagattaaatgcgagtcctgtggctgcgtaatgatttaccaagagggaaagcttctccttctcgacacgaacgaggagttcaacctcgaggatctcgagagttatgaggaagatgatgaattagaagaggaggaagaagaggaagaggaggaagagctgtactacgacgaagaatactgatggatttttttaatgtattttgccggtatttcctttgccaaagcgattgactcgtttgcctttattattcttataccatcttctctcgccttttttgcatcgatctcgaaaattatcggcttctttgtcctcagagttgctacttcgagtcctctctccatggtcgtcgacagatggacgtaggcctgatttacaggctttatgccaatttcgagcattctgtttgcctcttcttcgcttgttccatagtataaagtgtcttcctcggcttcaggcatatcattgagttttacctcaatgctatgtccgtatcttgccctgatcttctcacctttcatctcatacctcttcttctcatcgctgtaaaccagtgcctttagaacccatctgttggcccatctgtacttcttagcaacaaccttcgccaaggcttcgaagctaacccacccgttctcgtcgatctcgagtccaaatttttggggaaaatgtctaagcagaccagaaacgaattttccaagtttttccaccctctccttgttaagcacgatctctcccttaataccgcagcagcagttctcccccctgtagaatccgtgatctgggcagaaccttatgtcctccatctcactacctctttctgggctttattttcttccccgaaaccctaaggagcccgaacttctccaaggacttcacaatctgatcgaagatttcttcgcaatttattaatatttcctcatccccctcctccacgatccccttgctgaactctctcagcttgtcgatgtcgtcgattttctcaacgttgaagagtatgttcgaaattatgagttccagatattttagctcttccggctctacttctttgccgaggagactcattgtgtcaactaaaagctcaaatacaggaaagtagtcgattaaaacgatttttttgcccccttcgattggtgtatcgctgaagattgagatctcgggatcctcgggcagctcacctcttatgatctcaccgatctcgaatcggttttggtgcaggaagtactccagctcgtctagaaggagcttaaccttcagcatgtcttcagtctgatccgctaaatcttcgaattcgccgtttaaaagctttttaaatgattcgtagcgccttgagtcctccttgctcaggaatttttcaatgaactcttcataactcagctctcctttggagagtatctccctcaggatttcaatcctcctctcccattcaagcgctctctcggaaacttccctccctttatgcttcttgagctcgctcagctttccgctgatgacgaatctttcctccttctcccattccacgacctctctaagctcaaccttggcaccgagttgcctgagctgcgaggcaatcccgatcagtttctctctgtcctccgaagcgaccagtttgagccacatccctcagttcggttgaaaactttaaattttttgccctctaaggaaaagaagatatagcgtctcaaatcaattgcagctatgttcgagctgtcagaaagaatgaagaagatccccccttatctcttcgcagaaatcgatgcgatgaagaggaaaaagatagcagagggcgttaaggttatagatctcgggatcggtgacccagacctgcccactccaaggcatgtggtttctgcaatgcagagagcggttgagagggttgaaaggcagaaatatccgagctacgagggaatgcttgaattcagggaaagtgcagcggatttctacaggagaagaaaaggtgtgaaattggatcccaagaaggaggttatagccttaatcggttcgaaggagggaattgctcatttaccccttgccttcgttaacgaaaacgattatgtgctcgttccagacccgggatatcccgtttactacgcctcaactgtaatggcaggagggagagtttactatttgccgttgagagaggaaaacgattttctccctgaccttgatgcagtcccgggggatatagttagaaaggcgaagatccttttcttaaactacccgaacaatccaacttcagcggtcgcggagaaggaattcataaggtctgcgatagacttctgcacagacaatcgaatcatcttagctcacgattacgcctacggtgaaataggactcgacggttatcgaccaaagagctttttagagtttgaaaatgcttttgaagtcaccatagagttcaattctctctcaaagacttacaacatgactggctggcgtgttggatttgcctgcggtaatgaagaaatactcaagggattgcttaaggtcaagacgaacgttgacagcggggtcttcgaggcagtgcaggaggcagcaattgccgccttgagaggtgatgacagtataatcgaagagaactgtaggatatacgctgagagaagagatgttttcgtcgaaggactgcgaaaattgggtttcaatgttaggaagccaagggccacattctacgtctgggttcgtgttggggagagcagcatacaattcgtaaagagaattcttgacggagcaggaatagttgcaacccctggaataggcttcggcccctcgggcgaaggctatgtgagattcgctgtgacgagggataaggaagtaatcgcagaggca

>Bin11_61

ccttactttcttcgctttgaaatcatcctttacttggttcatgtaatccaaaagctggacaagggcttccaagctggaacgatcttttttgagttcgattacaacgtaacaatttttatccttctcttttgccaggatgtctatgaaagaacccttctttgttctgtaatgttttcctccatcaacgatttctaattcgggaaacagtttttcgaagttttcacgaatcagctcttccaagtgtgcttctttgacgacttcaacgtttttgtattcctctgcaatctcttccttttttatttcatccaaaactctttttaaaatcctttgcggaaaatctatcaatggtttggttccaacaattatctgcctgagggcatcgtcgattacccgagatactaattcgattgactttttccttcctattttctttcttactctactaacgaattccatgtaaaaatctaagactttttcatcgtacttatgttcatatgtcaaatactctaccccgtatctcttttttaacactttgaattcctctttcgggagtttaaaaagctcttctaaacatgttaaaagtatttctgggtgctttgttcttttcagcttatccagaaccgttattttagcaagaaaaagctccttttcttgactttttagaaattcttctcctctttttgtcagcttgaattttttagcatttcttgaaacgaaaccatagtaccttaaagcgcatagtctttcccaaactgcatagtagaagtcctcagatctcatagcgatttattttatcgagtatttcaccttccgagcctttttcgataacttcgatgatcttttttgcaattctcatccgatctttttcattagtttcaatctcaaagcaacctaaattttcgagttctgtaagaatgtcgtagaggacttcaagtcttcccttcccttcttttgttttcacaatcgcaatttgaatattccaaaaataataagcttacgatgctcttctgtgtttatacaaaataataagaaaagcgataaaaatcctaaggcggaatcgggaacttgtcaacaatcttcctatattcttccaatgctgtattaaacaaatccaaataatgcctttccgctactgtctggggtttatgcccgaccatgtattcgacggcctcgaacttaaggttatgctggccccttgcgaaattctggaaccatgaccttagccttacgacagaggcaggattatcctttggaggcttccaccttgaaggttgcaatctggatttatatgttccttcagcaagcaatttatcgaatctcttaagctttttagcaaattccgccggcatgaatgcgtaaaagctaagcttatagccctttgccacgtccccggtcggataaacggcaacgtctccatggaattcaagctttttggggttgaaagtcttgagtatcctataagcatgctcgaatctaatgccggagaacactataagcttaaataaaagctccgtggcttcatctttccatttttcctttaccagcttataggcttcggctacttgctcatttgttaagaaaatcttttcctcctcttcctcctcttcagcttccttcttttcctctttctctttctgcggctttgtcggcatgtgcttcttccaaatgctaaaatgtaaaccgttaagctctgtgatataatgttgctcttcaaggaagttaaggaaattccttaatccattgggcaggtatttggtctttttaacgggttcatccaaatccttcggctctttaaccccttcgggcagataagaatcgattgccttgatgtatttctctgcagtctctggatctatgcgttttttaagccaggcttcgaattctgccttatacttttcataaaacttctttaaatcgaatttttccttaacggatcccttttccttaattggctctacttccttaacgggttctttttccttaattggttctatttccataacaggcctttcaaattccttcttttccgattctgtggctttttggactcttaatcccgcagtcgtgggttcaaatccctcccggcccgtgatttttgtaaatgcggatgtcttgcctcataagctgcgaaatccaactttttggccttaaacctcctgacccgaaatcttagcacagattcacatcgcttcgcgacagcaaaatgcgaggcagagcttaagagctttatactgcctgcaatttttaaaattcaatctttgagtttcctccagtcttctttgagaaattctctgtggtcgaaaggttttttaatcgactcttcaaccttcttttcagaaatcttggggacttgaaggggttcctaactcggtttgggagccccaaatggttccctacggggcggaagagaaaatttgggggtagtatgaaggtgaacttgcaggatcacgtgcttgtaccgaagcacgaaattttaaaggagagcgaggtccaggaactcctcaagttactcggtgtaagtaaggagcagttgcctaagataaaatctacggacccgattgtgaaggagatcggtgccaagccgggggacatcgtgaagataaccagaaaaagccacacggctggtattagcattttttacagactggtgatcgagtaaggtggttctatgctcaacacccgtgctcttgcgagggcttatttcactcccgaaagacttgtaaagcatcagatcgactcttttaaccgtttcatcgacgagggacttcagagggtaatagatgagcagaagataatcgagctcgaggcgaaggacacctacatcgagcttggcaggatcagggttgggagtccgatagttaaggaggccgagggtcttcaggcacctttgctgccaagccatgcgaggctcaggaatttgaattatgctgcaccgatatatctcgaggctaccgtttacgacgagggcaaacccctcgagacggagttcgtggaaatcgggatgctgccagtgatggtaaaatcgaagatatgcaaccttagcccggaaaggatcgacaaggtcctcgaattcgttcttaagaaaaatgtggaatctttgagctacgaggagaagctttatttagcgggagaagatccgctcgaccctggcggttacttcataataaacgggactgagagggctatagtcacactcgaggaccttgctccaaacaaagttctcctcgagagagaggagaagtatggtgagatggtcgaagtcgcaaagtgcttcagccagagagctggctatagggctctgatcgtcgtagagaggggaaagaataacatcctcgaagtaacttttccgcaactgccaaagcctgtgaaattcgttatcttaatgcgcgctctcgggattgagagtgatcaggaaatcgtagagatggtgagcacgaatccagacataatgcggtacatgctccagaatctcgaggacgatgaggtaaaagaggtggagacgcaggaggatgcgatagaatacttgggaaaaagggtcgctccgggccagagcaaagagtataggatgcagagagcagagcaggtgctcgaccagtacttcctgcctcatttgggaatagacaagatagcaaggagggcgaaagcatttttcctcgcaagaatggccgagcagatatttgagctccatctcgggctgaggagagaggacgacaaggatcactacgggaacaagaggctcaggctcgctggagatctgatggaagagatcttcagagtcgcattcctcaggctcataaaggacgtaaaataccagctcgacagggcaaaacagcgtggtaggagcatgaagatgtccacggcagtgagaagcgatgtgcttacagacaggcttatgcatccgatggccacaggcaactgggttggggggagaacggggatttcacagctaatcgatcgtcacaacttcatctcgctcatctcacatctgcgaagggttgtttccccgctctcaagatcgcagccacatttcgaagctcgagatcttcacgcaacgcactggggtagaatatgcccctcggagactccagaggggccaaactgcggtctcgtgaaaaatttcgcccagtatgccgaggtcagcgtcggtgtagacgaaaaggaggtcctgaacattcttccgagattaggggttgagatgctgaggggttgaaatgaggaagtgtagggtttacgttaacggtgcccttgttggattttgtgaaaacggagaaaagcttgctgaagagataagggagcttagaagaagggggaagatatcgaatcaggttaatgttgcttatttctcggactcaaatgaggttagaataaacacggatgcaggaagagcgagaagaccgctcatcgttgtgaagaacggaaagccgctgataacggacaagcacataaagaaacttgaaaacggagagataacttttgaagatctcgtgaggcagggactcatagaatatctcgattctgaggaggaagaaaatgctctcgttgccgtttacgagaaggacttgaccttagagcacacacacctggagctcgatccgagtttgatcgtaggtatttgcgttggcagcatcccatacgctgagcacaacgcatctccgaggaacacgatgggagccgcgatgataaaa

>Bin11_62

gaggtgtaggggatgttagtggaggcgaggtggaccaagattacggcatttccataaggcagctcttcttttgtctgctcaagtccgtagttcttccagtttatactctccactgccctcgttagggcacagccaccctgctggtagagaagcggtatcttgtttgcaaacctgaggaggacgatcttctctgtctttatttctccgccgtatgcaattgcagcctccacgagaaatggatgtccggaataaacctttggtcttctcgtaaccgcgtagacgaattccggagagtattcggagatcaaactgcgcatgagaagctcctcaccaatgggtgagagacagtcaactggcggcggaagtacttcaacactcttgaaagcttcaataagctttgcagcctcttcccttccaagcttctttggatcctcgtttcccgaaaatcctgcttttgcaaggatttcgtcggcaattttgtccccgatcctcacgaactcctcctttagaaatttcctaagcgttggcgaatctgtgaacttaagcatgcccatgagcttaccaagctctatgccgtgtggatgcggctttatcgcttttgcgggttttggcatttcgtttgacgacctcttaaactcgtatatgtttccttcgggatccacaaaagtgatctttgcgtgcggattcacgactgaggtctctttgaggtattcgaatacactctgcttcctgtccttgacgtagctgccagagatttcgaattcgacccttgttccgtgtggcatgtaccagtcttcttccccttcactttcaatgatgggctcattccttttcgtatctatgtaaagcaaaactctgtaagctttttctgctccgatctgcttggtcgtgacaacggttggttttccagtggtcagctgtgcatacaagactgcagctgagattcctatgccctgctggcccctgctctgccttatttcatgaaatcttgaaccgtagagtagcttgccgaatgctctcgcaacgagttcctttttaattcctggcccattatcttccacggtgatcctgtagttattattttcgacgttggaaattctcacaaagatgtccggaagaattccggcttcttcgcaggcatcaagggagttgtctacggcctccttcacaaccgttatcaacgccctcgcgggattcgtgtaaccaaggatgtgcttgttcttttcaaaaaattcggctatgcttatctcctttaactcgctcatatttcggcgccgacgaccgtttgcgtctttgtaaccccttcaattcttctgatctcgtccacagtcttgttcagctcggagaggctgtctgcgtggattatgaccacaaaatcgaactcgccaaacacgtggtagacgtcctttaccttcttcatgttcttaatcttcacgtatacttccttctctttcccagcgacaacgttcacaagcgttacaccgactaccatgtttctaagtttctttggagtttttaatatttttccccctctcaggataactttaaatctttccgcgccaactcagctcgtgatagagggctggctgctggatgcagaatacatcacgatcaacgaaaaagcggttcttaggctctggtgtaaggatcgtgaaacctttgtggcatatgacggaagcttcaaaccttatttctatgtccttgacatcgatgaggagataattcagaaggcgatggtgagcacgaggagggaagttatatccccggactcgtatgaaaaaacgcaccttagcttcttcggaaagaagattccggcttttaagatatatgcaaagcatccccagcacgttccgaagctcagggaatacttctcccagtttggggacgttagagaagcagatataccttttgcctaccgctatttgatagacaaagaccttgcgtgcctcgacggcgtaacaatcgaggggaaagagattgaaggaaagctcagaagctatgaaatagagagcattgagagggtgagcaaagaaatcccggaattgaagatctttgccttcgactgcgaaatgctttccactttcgggatgcccgaccccgagagggatccagtgatagttatagcgatgaaatggggggaaagcgaaaatgaggaggaaatactccagggcggagaaagggagatcctgcaaaggttcgttgaaagactcaggcatgtggatcccgacgtgatcgtcggctacaatcaggatgcctttgactggccttatctgagaaagagagctgaaaagctcaaggtctcgcttaacgtcggaagagatggcagtgcggtaacattcaagggtggaaggccaaagattgcaggcaggctcaacgttgacctctacgacattgctctgcgaatttcggatgtgaaggtcaagaaactcgaaaacatagcggaattcttaggagccaagatcgacgtcgaggacatagaggccaaggacataaatcgatactggcaaacggacagagagagggttctcgctcacgcaaggagagacgtcatccatacctacatgatcgccaaggaactgctcccaatgcattacgagcttgcaaggctgatcaggctcccgcttgacgatgtttcgagaatggggaggggaaggcaggtggactggcttctgctgagtgaggcgagaaaactcggagaagtggctccaaatgcgagagagacgccaatggaaagctacgagggagcctttgtaatagagccggagcggggactgcatgaaaacgttgcggaacttgactttgcgagcatgtatccgtcgatcatgatagcctacaacgtcagccctgacacgatcacgagtggtaacgactgttatgtcgccccggaggttggctacaggtttagaaagcatccagacggatttttcaggagaattctgcggatgctgatagaaaagaggagggagataaagagagagatgagagggctcagcgaaggcgacccgaagtataagctcctcgacataaaacagcaaaccttgaaagtgcttacaaattccttctacggctacatggggtggactggtgcgagatggtactgcagggagtgcgctgaagccacaactgcctggggaaggcacttcataaagacttccgcaaagatcgcccaggaactcggatttaaagttctttacggagatactgacagcatttttgtttcaaagccaggaaaaagcctgaaagaacttcaagaggaagtttccaacctaatctctgagatctcaatcaagcttccagttcagatcgagcttgacgagctcttcgagacgatcttcttcgttgaaaagaagagatacgcaggacttacggctgacgggaaaatcctaattcggggactcgaagtcaggcgtggagactggtgcgagctggctaagagggctcagagggccgtaatcgagataattctcagggagaagaatcccgaaaaagctctggattatgtcagagagatcgtgaagtcgataaaggagggaaaggtcagacttgaggacatcattatctacaaaggacttacgaaaaagccctcgaagtatgaagctgtacaggcgcacgttaaggcagcaaagaaggctgaagaagttggcatattctatcctgtgggctcaaaaattggctttgtggtcgttaagggcgctggaaacattggggacagagcttacccgatcgatatggtcgagcacttcgacggcgagaatctgaagatcaggctgaagggaggggaggagatgaagaagctggataaggattactacatagagcaccagataattccagcggtgctaaggattctcgagagattcggattttcagaagctaagatcaggggagcctcgcaaacaagccttgaggccttcttttagattttaagcttttttagtgattttaagcgcgtgagttggaaaggcttatatactgccctgcaaagggctgagaaaaactgaaaggagggttagaattggcggaaattacggaagttagaatatacaaatcgaagggagaaggaaacgttaaggcatacgcctccgtaagcctcgacaacgaattcgtcgttaagggtctgaaggttatcgagggcgaaaaggggctctgggtgagtatgccgagcaagaaggggaaggatgggagctatcaggatgtcttccatccaatgagcaaggaggcaagagataaaatagttgacgcggttctgaaggcctaccagaaccaggaataaaaaattaatggctaatttgaaatttttcgtttattggagcttttttagcatatcgggacgaatttggttccgtagtagattattcccgtttctccgtcctctccataccttctgaaggccttctttaccctcatcccgatctcgatcctgtcggcgcacacctctgcgagggtccttgcaccgtttttcagctcgatcatcgcaatgatccttccgtttacgattgtatagcttatgacctttccttcatcggggaaattctcgtctctaagctttcctttccttctacactttgggcagagttctctttttggatagtagtggcttccgcagttctcgcagtagctgccaatgacgtcatatctgtatcttatctttctccagaatctcggaaccataaaatcacctcgacaaaatcgtaaccacagcagtcgcaccggttccaccaacgttgagggccagagccttttcagcatcaacctgcctttttccagcctctccgcggagctgaagaacgagttcaacgatctgccttatccctgtagctccaaccgcgtgcccacaggctttcaaacctccagatgggttcaccgggatcctaccacctatttctgtctctccctcccttatcagtttcgctccctctccctttttcgcaaatccgaggtcttcgtaggccatgatctctgcaattgtgaaggagtcatgcacctcagcaacgtctatctctcttggctcagtcctcgcaattctgtaagcctctttcgaggctttgagaactgatctcattgtcaccagatcctttctgcactgcaaggccaagtaatcgctcgcctgggtgcaggcctcgacgaagatgggtgtatcgcagagttttcttgcaatctcttcttttgcgaggatcaacgcagcagcgccgtcgcttaagggggcgcagtcgagcactttaagcggatcagctatgacttctgaggagagaacatcctcgacacttatttcctttctgaactgagcttttggattgtgaacggcatttttgtggtttttaacggctatttttgcgagatcctcttcggtcgtgccgaattcgtgcatgtgaagccttgccattattgcgtagagcgccggaagattcgctcccacgaacctctcccactcctgatcgacggaggttgaaagaactcccatcgggtcgacggaatctgtaaccttctcaactcccgctgcaatgactatatcgtgcattccagaagctacggccataaaagcctgtcgaaacgcagatccgccgcttgcatctgcattttcgactctaacggcaggaattccgaactcagcgaggccagcatggtccgcaatcaaggcagcaatgtgctcctggtctatgaacttccctccgctcatgttaccaacatatatcgcccctatctcctttccctcaattccgcagtcccttagcgcctcaatcccggcttcaaggaatatgctcctgaagctcttttcccagagctcaccgaattccgtgcagccgactccaatgatcgcaaccctcattttatcatcctcctgtgcttcagataaagcgcgtagtcgatgtaaacgcagttcttcaccttgtcccagacccttagactccgggggtagctgaggatcttgtccgtaaccctgatcgcaaaggcgtcgcttccagctccagagccgaaagaaacgagcagaatcctctgttcaggctttgccacgtctaaaatcccgcacaagcccagcagagatgccgcagaataagtgttcccgatcgccctgacaagcatcccgtcggctattttcttctcatcgaatccgagcattctggcaaccctctgaggaaacttagcgttcggctggtggaacacggcgtagtcgaagtccttctcggtgtatccgcacgtctccatcagtttctttgctgcagaggtaacgtgcctgaaataggcgggagttccagtgaatcttcctccgtggctcgggtagggctgaagatcgcgtctccagaagtcaggtgtgtcgcttgtgtaggaaaccgttgcctcgatttctgcaatcggatcccttccgattaatatcgcagaagctcctgcagcggctgtgtattcgagcgcatccccgggccttgactgggccgtgtcagagcctatggacattccgtattcgatcatccccgccttgaccattgcgtagcagagctgcattcctgctgtcgcggccttgcaggcaaactcgagatcggcgcagtagtagtaattcccgattccgagagcctcgcccacgatcgttgcggtcggcttgacagcgtagggatggctttccgagccgacgaatatagcccctattttctctggtggaattcctgccctctgcaaagcttctcttccagcttccacagccattgtcgccgtgtcctcatccaaatcaggaacggacttctccgcaactcctaagctgtttttgatggcctcggggtcttcaccccatatccttgctatctcctcaactctgatcctgaacttcggaatgtaggttccgtatgaaacgatcccgatcacacaaaatccctcctcagcctatctatcatttcgccaatctccagctcggtcctaatcagcggaagcttttcagcttctgcaatttttatcgccatttcgtcaacctttgagacgccgtgaagaacaacagcagagggcttcaagctcgaaaccctgacagcaaccagcggcgaccttccgctcgtgac

>Bin11_63

ggattcgggctctgccctattgcaacaactacagtgtcgacgtccatcacgaactccgatccctttataggaacgggtcttctccttccagaagcgtctggctctccaagctcgaatttgatgcactccatctgcttaacccaccccttctcgtccccgatgtacctgacgggggctgttaaaagcatgaagtttacaccctcctcctttgctcgctcaatttcttcctttctcgcgggcatttcagcctcagtccgacgatagactatgtaaacctcctttccgagccttctggcccatctcgcgcaatccattgcaacgtttccacctccaatgacagcgaccctctttccaacctttatcggagtatcgtaattcggaaaatcgtaagccctcatcaggttgagccttgtaaggaattcgtttgcggagtaaattccattcagattctcgcctggtatattcatgaagttcggcagtcctgcgcccgtgccgatgaatatggcatcatactcttggagcagctcgtcaactgtgatcgtctttccaacgatcacgtccgtctggatcttcacaccaagcgacttgacgtattccacctctgctctcactatgtccttcggaagcctgaactcaggaattccgtaaactaaaactccaccagcttcgtgcagcgcttcaaaaactgtgacatcataacccttctttgccagatctgctgctgctgtaagccccgcgggaccagaacctataattgcaacctttttgccgtttggctgaggtttaggtggaactgtaaagccctttgccctctcgtagtcggcagcaaacctttcaagccttcctatgccaaccggctcatatccgggcctctttccgagcgtgcatgagccctcgcactggttctcgtatggacagactctgcccgttatcgccggaaggctgttcttctccttgattatccttattgcgtcgtcaaatctgccctcccttattgcgagaatgaactgcggaatcgggacgttgacagggcaaccttcaacgcagggaggaaattccttgcctttcctcggcttgcactgcagacacctcttcgcctcctccatcgccatctcgggtgtgtagcccaatggaacttccttgaaattctttatcctctcctctgggggctgttcaggcattggaacctttttttcactaatcttcgccatccgcatcaccccttgcaaccgcaaacccttttatactcctcgagcgcaatcctttcttcctctatgaacctcctgtttcttgccatcagaagatcgaagtcaacgagatgtgcatcgaattcagggccgtcgacgcaggcgaatttggtctctccccccacctcaaccctgcagcacccgcacattcccgtcccgtctatcattatcggattcaggctgactatcgtctttatcccgtaaggcctggtggtttcggcaacagccttcatcatcgggactgggcccacagccacgacgaggtcgaagctttttccctcctcgatcagcttcttcagaagcagatgcacaaagccgtgaaatccttcagaaccgtcatcggtggcaacatagatttcatcgctgaactccgctatttcgttcttcaggatcagagtttccctgcatctggcaccgagaatcgagaccacgtaatttccagcattcttgaaagcccttacctcaggatatatgcacgctatcccgactccgccaccaacgcatgcgactttgccgtagtatgcaatctccgcgggctttccaagtggcccagcgacgttggcgaggaaatccccctccttcattgagttcagcttctttgtggtctttccagcctccaaggcaactatcgtgatcgttcctctctcaggggatgcatcgacgagagtcagcggtactctctctcccttttcatctattctgatcataacaaactgccctggttttgccttttttgcgatcaaaggtgcctcgatctcgaacttcttaatcgttggagctaagtcgacttttttaactacacgatacatataaacggtgaagacgtattatgtttaaatatttttttatttccaaaaattgcttcgattaaacgtttagaatttccgatcccaatcttcccctctctgagccccctcagcgaaagttatgggcggcaaacatcgattcgcattcaggttgatcggatcaactttaagatcaatttttttaactctatgactgaaaggtgaaggatgggaaggattacgattaacagtgtcaggaaggtttacagagacggaactcacagggcaagagagcccgaggaaaccctcagctggatcgagcccaaaacagctgtagcggggataacgaggctggcaaacattacaggactcgatagaatcggaattcctatcttctcggctgtgagaccaacagcagcgga

>Bin11_64

agaggtatcttccacaaaattccacggaaatatcacccagacgttttctgcaacgtattctccgagatagtcgggcacgaattcggagttctggaacatcaagagtgcagaggtctttacttcatcggcgctgatcctgctcagtgccttcctcatagttctgcccgaatttacgaaatcatcgacgatcaaaatcctttccgctgaaatttcctcgctccgcctgaggtcgaggctgaccgcttccacaccaaggtaatcgctcagaatcatcgaagcataccagccaccctttgcgggggcgactattttttctggtctgaatgagtcttcaagaacctgcatcgcaaccttcctgcaaagcatttcggcataattccagttaacaaccacatacctttccattcaaccaccgctgaagtccagccttcccaccgagttgccaaagtaaacggcaaccttgcaaggcatcgggttcagattgaccttcttctggaactccgtctgcgactgccatgttgaggagttcacgaggaatattcccctgtaaaagccaactccgaacgtgtgcacgtgaccactgtggatcacgtcgggaatttcatcgattacaaggtggtcctccctgtcgggagcgatcgggctccttccgccgtataacgggctgagatgacgcctcttcaaaagctcctccattcccttgtgcggggcatcgtagctcagcctcgggatcttcgtcacgatgtcgtcaaggcttcttccgtggtatacgagtaccttagtcccctcgaggtcaatataggcaggatttcccacgcatttcgtgttccttggaaacaggctctcgaactcctttggcagtggtggctgaggttccgcctgccgaactgcatcgtggtttccgggtgcgactatgatgtctatctccttccttatcccgtccaagttctcggctgcaaactcgtattgctcgtatatgtcaattatcgcaagctccttctcctgttctgggtaaacgcccacgccatctacgacgtctcccgcaaggatcaggtactttaccttctccgcaatctcgttgagcctctcatcatcactctcgcagttcagccactgaacaaacttcttccagttttctgcaagaaagtccttgcttccgaagtgtgtgtctgaaatgaagacgattccgaaatctatctccttcctctctccgttgacagggacatcgggaaaaaggatcctttcagcaagaatcgagttacccctcagcgttcctgtgacgccgatcacttcgtccccgagcaattctctcgcaatttccgcgttctttccgtttgctatgcagttaactgtgccagtttggtcttcgagctctatcacgtagtggtcgctacgctcgaaaacgtttgaaactatacccacgacgctcacggtttcgcccctgaaccttccgagatttttgatctgggttggctgaatcctgttccgcagaattctggatatcttctcgagccgggagttaaaatatgcaacgaagtcctcaacctttccctcaaccatggagcttcgaacttccttcaaaactctgatcctatgcctgttttcctcaggctcatgtctttttacttcaattcgacgcgaactgacggaattgatagcgttcagaacatcctcctcgccgattatgaaattccccttcgacttcctgcaaacctcctcaatcaccgcttcaggatcctgagaggagcagatgagctgggcagcggccgggcttatgttgaaacccctcacgagaaattttttggcaactgtcacggggtcgatattcttaactaccctctgccccccttcattcatggaaagggctaaggttattgagttcataaaggatttggtctcaacattggccatcgtcggggcgatagtgatcctcggaattctcataacaggttgctggcccttcatggtagccgtggagtctggaagcatggagccaaatctaatgccaggggatgtcgtgatcctcatgcatccgagcagagtggggctcacgacctgggaggaggggaagcagatagaatacaggagctttgggaactatggggacgttatcgtgtactaccccaacggacacggaaaggcgatcatacacagggtaattgcatacgtcaacgcctcagaaagcattcccgagctcagagggggaaggctatatgccacagaaaagatagcggagatcagcggttacgtaacgcagggagatgcaaacaggtattcggatcagcatgtgggcacgatttacacaaagaggggaattgaggagattaagcctgtacaggaagagtggatcgtgggcgttgcaaagctgagaatccctctaatcggctacctgaggttgctgatccccatatagcgcgcgcacctttacgaacatcgggccacgaacgtctatttttccacccttccagactatgtaattcctgacgttctcctgaatgctcaggtttatgcttgaaagcccttcgatgtttctgacccttatcaaagcctcattctttcctctaagtgcatattcctcgattttgctggccgcaagaagggagaaagagtctatgtatctgattccgcatctaaccccttcttcaaacaattttctgaactcctccgtttccggaacgccgtaaacacttccaccgtgcaccacgatttcgttcgcaaaagcaggaccgcagagctttgagttcgcttcattctcttctacgaagaccttgacccttctgccatagatctcaccttcgaaggcgagaaaactgcaaggactgtcagcatttgcattcttttctgcaattttaacgatttttcgcgcaatttcgaagcctgttgcacttcttggcacttcctttaatcttatggctcgagctatgtccagatcgctgagctttatctcgccgaagaactgaggataaacgagctttctaacatcctcgtagccgaagaggatcatcgcaagcctttcgacgccaaggccaagattcatcacgggatagtctatatcatactccgcaagagctgttggagagtaaacgccgaaggtcgctatttcaacccaaccgtcgctatacttagtctttgagccgactagcttaggatagaacgcgtaaacttccgtttgcgttcccggaatgtagtacttgctccttttctcatcaggcctgaacctgaatttttcgaagccgaactgcctcagcaaagcttccgcgaccgctttaccatcgtcgacgccaacgttttcgtctgcgattacgcagcttgccgagaagtaactgtaaagtctcgttgcatcctctccctgctcctttctgaagcatctgtcaacgctgaatagctttattggcagtggaagcttgtctgccagctcgctcagagttatgaaccagcccgttgtcatgtggctcctcagcgtgagcctgcttggctctggcttcagctccttaaactcgggaaaaacctcgtctatgacccttattgccgttaagtcgtccaccttgagcacccttgcaacctcgtagctgagatcgtctccgtcgagctttcccttcttgtaggtatggaaaaccctcctcagctcctcctcatcgaaatccctgcccgtgatactctttattttctctatcctatccaaggaaatcccgacgttcggcttgggaagtgaagcaagataaaaacagcgatcgagtacggccaaagcttcctttccaaattgcttcctgatgtggacatcttcgacgatgatcgggttgaccacctccctgaatcccatcgcgatgtaagctttcctgagcatctcgatcgttttaaagagcgggtgctcctcgccataggaataacccagacggggataatttctgttgggatccctctcttcgagtatctcccttccgctgagccatgcattttcaaaatccttctctgcaagctccttgaacttccttgcgttgaatttcatagaaggttttggcagtaaagttatttatctttgtcgtaatttatgccgtgctgaagagggagaaaaagagggagcgcgaattagcgatggagaggatcgtctacctgatcgagagggctgaaaagttcaaggaagtcgattacgagctttcgagaagacacgttgaactcgcctggaagatctccacgagataccgtgtcaggatcccgaaagaactgaagatgcggttctgcaaaaactgcctttatccctacaaggcaggaaattttcgcgtcaggatcaacaagtccgcagtgatcgtgacgtgtctgaactgcatgaacgtgaaaagatatcagctgagggataaaaatgttggaagggcttgaaaaaatatgcgaaatttgcaaggctgagggatacgcatacgatgagactccaaaaccactgaggccaaaagcggcggaattcatcgtcgctaagccgaaaaactcttctgaggtttcagaaatcctgaaatttgcaaacgaacgaaaaattcctgtttttattcgcggaggtggaaccgggctaagcggagggtcaatcccgacaagaaagggaattctcatctcaacggagaaaatgcgcgagatcagaatagacagggaaaacagggttgccgtgtgccaggcaggagttacgcttgaagagctggctcgaagtgcagagagatctggcctcagttttccgccaaagcctggggcggagaacgcgaccgttgggggcatgatagccacgaatgcgggaggtgtaagggcactgaaattcggggtgatgaggaattacgttcttggcatcgaagccgtgcttccggacggaagaattttgaaactcggggggaaaacgttgaagaacacctctggttattctctgctccacctcataataggaagcgaagggactcttgctgtgatcacggaagcaaccataaagcttctgccaccgctaagagacatgacgatgctcgcaataccgttcagaaggactgaggacgcgatcaatttcgcccttgagacattcctgcacatgactccgatggcaatggagttcatggatgaaagggccgtgaggatcggagaaaaggtcagcagaaagaggtgggtcagcaaggggggcgaggcacacatactcgcgatattcgagcaccaagaagaagctgaggaatcttccgagatcgcattcaggcatggagcgatagatgtgttccttccaagtccgagggaacagagagacctcttagagctgcgcggaaagatctacactggactaaaggaaaggataatcgagatccttgacacttgcctccctcctgcgacgattcctctgtatttgaagacgagcga

>Bin11_65

gaaatcccatttctctccctttctgtagtaaaccacacccgagtcaagaattctgatgtctttgccgtagtaaaagcagtctctgagctccttagaactcatacagatctcttcaccgttgttcgaaagttcgaaattatttggaaactcaataagctttcctaagggctcaaatttttcgagaaatgcagaactatttttcgtaattgtgtaaaagccctcttcgagcatcaaactagcttctccatcgctcaagatgcacgagtctgggcagtagactttcagatactccgcatcatcgcttccgtatggattcgggcagatctcaaggagtaaggctaagctaattattaaaagaaagttcattccttatctcgccgattttggctattctttcggctacaacgttgtgctggtgaatgctctcctcgttttcctgccttaaaaatattaaaaactcattcggagcttcggggtaggctttaaggaattcgtaggccatttttcgcacgctatcctctacgaacattggccttttatgcgcccgctcgatcacctcgagttcgtcctccctcttgagtatctcaaaggtttcctcgctcattcccgatttggctatttcgatcagctttgatatcgaaaccctgaagctatcgttaacctgaacctttatcatcgcccttccgcgctggttgtgggtggcaatcggcaccacttccaagatcttctcgatttcctcatgcctgaaaccttcccttaacaacctctcggcagctcttgcctttaccagctcctgagcgcatggacaggctgtgattccgctgacctctgccccaacgaatacgagtctctcgccagctctggaaatcgttgcgtctccgaaaattctgactatttcttgagtcctttgcgaactcgccggggctttcttgcgccttatgaattctgaagacatcgaaacttccgctctagtcgcatattcgtgtctttccagcaagttttctgcgatctttatcacgaggtcctctatttttccgataggttcagcagtcaatctttccagcacctcgtctatgacctcgaaattcctgcttagactgacaccctttaaatgatgaggtaagttgacgaaaacgtcgaaagttgagatgagaattatcggcctcttttcctccctttttacctgaactaactttcttattcctttggccccaactctgctcagctcgatgggaaactgcggtcttaaaagctgaatgtctggaagcatgaaatcgctacgattgaaagattttaaagtttcgttatgagaaagcttaaaatcttcctagcagaactgagtatatggagttcaagtgcgaagaacacatccgatgcacgctcaagtgtgcctttgacctgagctgcctcgacctcgagatctacgtcacgctgctcaagaaaaatccggccacaatcgaggagatctctgagcttgtcggcaaagacaaaagcactgtttacaagtcacttcaaaagctacttgaaagaggtctgatagaaagggactacagaattttaaggagcgggggatacaggtatctttacaagccaattcccttcaacgaattcaaggagcaaatgcttaggactgtggagaggtggagcagggctctgatggaatcaatttcggagctcgagaaaatggataaaagagggattcaaagggccttggaatcagtcctctaaggttggcaacctgatttcgaaaactgctcctgaaggtatgttatctcttacctctacttttcctccatatgcctccaccagccattttgcgatgaaaagccccattccagttctctttgcggtggaaaatcccttttcaaagatcctcggttttatgtcgtcagggattccttttccattgtctgaaattctgcaaactgcccactttccgttcttgaatgcttcaacgacgacttttacgttttctcctccatgaaggaatgcattgctgactatgttttcgagaacactgctcaaaccctcgtttgcatacacgaaaatttcctccacttttgcctcaatcctgtaacgcttggaaatgtctctaactatttcggcgatgtttataggcttcagaacgtcaatcgctgactccaaatttcggatgtcagcaatcagcctcattatatagtcgattctctcctgcgcttttgcttttaactttggatcatcgagctcaagtgcagcaattactgtggtcagtgcattgcctatgtcgtgcctcaaaatgctgttcaaaagcgatagatattctgctctccttttcagctcagcattcatttcatggattgcagttgtgtccactgcagttgcagcaattgctggctcaccgttgtaggttatcctcgccgctttgattgtgagccacctgactttaccgtcttttctgatgatcctgaagctgaaggtatcgcgatctttcctcgcgtagtctccctcgacgcacactttttccacaagttctctgtcctctggatgagctatgaacaattcttttccgaccatttcatccctacggtatccagtgaactcctcaaatgccctgttcacgtaagaaatttttcccttttgcgttatgcagattggactcaaactttcctcgatcagagccctgtaaaattcctcacggtttctgagctcattttccagtctttttgcccttgttacgtcgatccagctacctaagcagtacttttttccttcaatctcgatcggggaggcgaatccccacatccacctcacttccccactctttcgcatatatctggcctcgtaaaaaatggtctcgccattgatggttcttctaaggtactcctttgccttttctaattcctcaggaggcgttattttaaaaatatctccactgtacagctcctccttcgaataacgtgtggctctctcaactatgtcgttcacgtagacaaatctaccctcactgtcgagtatatagacccccgcgagcgttttgtccaatatctccttccagttcattctcattggctacgggaaatagtacattattctttatttaaatctttcgatgaaaaagggtcaggaaaggaagtctttgaaaagctccaaaagggagggattcgttttaaggagttccactattatggcctgaacgctcatctcctctatcttcttccccgagatacttctcgcaagactgttcagagtttcatcgtcgagcttcaggaagaactcctgcagttttttatttctcctcagccttgctccaaagtctgacttccatcgctcgtcgtatctcctcagaaagcttgcagagcaatcattcgactcaatggcttccgcagcgactgttccagcatggtagccagcggca

>Bin11_66

gattccctcatccgcaagaacccttgcatcacccctgatctcagcatcgatcgaatagttgctgctcagctcctcgaccacacttcggatctcgaattccttcatttcccctgcctcgagctgtttaacgactttgattatatctatgcccctttctaaggatttctccgccttttcgagaaactcgggttcgttttcaaccctgtaaagctcgagaaagccctttacgcttgtgagggcattcatgagatcgtgtctcaaaaccctgagcaggaatttcagctcctcttcatccttctcaattctttcaagcattgaattcatgcttcttgccagactttcgatttcttcggtcccttcaagctcaattctgtcctttgtttttgcctttttcgcgaattcctcgagcttctcgatctttgaaacgatctgcctgtcaaccacaaagaccgaggctatcatcatgaggaaagatataagcccgaaaaatccgatggtaaagagcaggttgttgagatagaaagaatagagcggattttcctgctctattctcaggaatgctttatttccagtcaaatcctcgagctcgaggtagctcacgagcttttctccgaattcccttcctccagcaattggaacgagctccgccttcaatcccagcacgtctccaatctctccaatttcgtcttggtccagaattctacccatcaaaatgtatccttttggctctccacttccatcgctcctcttaatctgctcagaagctaagatcagaagtgatccgttgagcagaagataaccagaccttccaaacagttctctaagggtaaagatgggtgaaatttccatttcattccattccgaatcgtagaatttcgcaaatacaacctctccgttcctgtccaagaacaccatgaggttgatccttgcgttcacgaaggtctcaaagactaagttcgatctcacgtaatcctcgcttttgctttcgatgaactcgtatgtgtcatcccagaaagcccagtccctgcatagggtttcgagctctcttgtcgaaagttcgagcattttcttaaaagcaatctccttctgctctgccatgacttcaaggctcttttcaagatttcctctcgtgacgctaaactcgaagtagcttagggaaaggaacaagatcagtgccagggaaacaccagccaagagcattagctccctgatcttcattggcgctcaatgagcaaagcgcttaaaatatttttgtctcaaagaaaaaattacactgctataagttggagttttaaaattaaaataattataaaagcctgtaggtgcggaaggcatctgcttctccgttgtaagctcttttcggtattatcgcccgaccgattgcagaaaccgcgaggatcttcgcaagatcacccggtataaaaggtatgacgccgatcgccaaaaggccgtatatgtcgggcggatacatcaggcttaaccatagaatgcctaaggtgtagatgcaaagattcgcaaacagcagtattgggaaaagcgtaacgaaatatctcgctccgatgtacctgtcgatcaagaagcctacgagaaacgctgcaagaacgaaaccgagcaggtatcccatggttggaccctgaaggactgcaaaacctcctttaaagcctgcaaaccagggtatgcccgctatgccgagagaaacgtagagggcctggctgatccctccccagtttctgccaaggactattgcagaaagaaagactgcaaaggtttgcatcgttacgggtacaggggtccagggaagataaagccttatctgggcacagataccagtgagcgccgcgaatgcaagggcaagcgcaagcctctgctcgaaattcgcctcgtacctccaccggtaaaacctgtagctgaggtatatcatggcagtacggttttaaaatagtatataaactttttgcttaagagaattcctgtccctcgatgctcggctcgtgtccgggaaggattctgttcctttttacgaggctcagggctttgaaacagctctcataccattcgctgagatcaacatgaagtcccgggggcagaaatggaagctttgagctaacagaaaccttctgaccgctgctgtcctcgaaatccagcgtatcttttggtggctgcatgttcacgtacgagtaaaagtgatcgccggaaagaaggtagtacccgctctcagccctcaccaaaaccccctgaagacccttagtgtgtccgggaagcaaaacgagtcttataccttcttcaatctccacgtcaccgttcacaaggcagagatccatatcttcaaggggaaaaaagagctctctctcgtagatctctctgtagtgcatcgggggattcatggcagacttccactcccttttctgcacgtatattctcgcgttgtggaaaagcctcgcattggcaacgtgatcgaagtgcagatgcgtgattatgagcgtttccacttcctctggactcaaattaacggttgagagagcacttctcaggcccctctctcccccgccccctgcaaggaaagccgtgaacaaaacctccttttggttcagaaacgcccgcgtcgactaagatcttcctgtctccgctttcaagcagccagacataaacagggcctgaaacgagcactcgcatgtcagcgaacattgcaacagctcctaagggaacgctgatctcggcctgctttaaaggtctgatgcgatacatggcgatgcgtagttaacgatcaatataaacctttttgcaatcctgcgataaaaaattttagccctgcatagagaatcatgctatggcgagaattctgttcgtaacaggcaagggaggggttggaaagtcaactatttccgcagcaacagccataaggagctcggaactcgattacgatactcttctcatttcaacagatcctgctcacacccttgcagaccttttcgagcaaagagtaaatgctgagatcacgaaaattagccaaaaactggatgcagtgcaggtcgacgtcgttcacgaagtttcagtgcattattccgagatcatggagtttatagtcgaagtcctgaaatcgaggcagatcgatgacgttgtcgcttacgagatcgcgaactttcctggagtcacgggtgcagcggcgctgctgaagctcctttcataccttgaagaagatacacatgacgtctacattctggacatggttccttccggagatgcgctcaggatactctatctgcccttcatcttcagcagattcagcaggaggttcatgaagctccttactccattcttcgagtttggaaagccaatatcatacttaactaacatcccagtgccttcaaaagatgtgatagagagtgaaatccgtctgcttgacatgctcgaaaggatccacggatacataacagatccgaagattgcgagcctgaggatcgtgatgaatccagacagcttcagcatagagaacgcaaagagaactctgatgcaggcatcggtctacgggctaaacacagacctcgtttttgtaaacaagatttttccggaacagcttgctgaaggatatcttagcacatggtacagggcgcagagggagtatttggcgagatgcgaactcgagttcagcccgattccgataaagaaggtaaggctgctaagccaggaagttaaagggatgcaattattgaagatcttggcgcaggaaatttttgcgggagaggatcccacgaagatataccatgaaagcagagcaattgaaatcttccaccattcgaggggcttcgagatagtcttaagggtcccaaatgtgagcagggaagacctcgaggttgaaaggtttggtgatgagcttttgattcatctcagcactcctgcaggaagagcagccgtgatactgccccttccagccatcgcttacaagtactcgatgaagaaggcaaagattataaatggagaactccacgtttacttcggtgaggaggatgattgaggatcgtataaggctctttctggctctaatgccgactggtcttgaagagccgatgaggcatttgctgagggcaaacatagagtatctaaaagctctgaactctttcatcga

>Bin11_67

gcttcagcttccgcctctgcggttctttcaatctcctctttcttttctacaaagtcctttctcaaaacgggaagttctgtttctttgcagatcctcttgagagtatccaaggatccattgaaagaccttgcagttatgtaggaaattgctatgcaaccagcattctcgtatgccttcagtatagccaaggggtctcttccacgtaggagatcgccttttgttggtgaaaacgcctttatttccccgatcacagcattcctccttctatcctttatggcatcaataaacccaaaagcaatcacctgttcctttcatggatgactattcccacttagagtatttaagcttaacggatgggaaaataaaaatgcataaatgtagctcggaatgatctgcttcgtgaatcgcctgctaattctcggcctttcagcccccatcgtcgccctccttggagcaacaatctccatttctctgagtggaaattggaacttcatggagaacagcattagggatctcggcagacttggaagcgattcagccgctgcattcaactcatcgctaatatgtgcaggattacttggatttctgtacgcctttagaatttacctcagatttccgaatcccgaacgcgtttcaatgagctttcttgtatctgcgttcatttttctcattctcatcggcatttttccagctggaacgcaatcccacagctccatttgcctcggcttcattctatctgccttctcaagcatgctcctgcttgcgattttttcgataaagaagaacaaatttctggccaccatcactactgcatttctgctcctttgctcgatttccggaattttcggcatgctccatcttgtgaggatcgcattcgcagaacttgcggcgatctctggatttttcatctggtactccatgatcgcaatcagcctctggaatgaaagcgatttatagccaaaacttcagggttctgcatggcagaacttgcaatcgagggtgggctttgctttgtcagcggaaagttcgagaagtgcaacatcggaattgaaggtggaaagatagtttatgtgggcagggagaacttaaaggcggatgaaagaataaacgcgaggggctgcctcgtcattccaggcttttttaacgctcacacacactcagccatgacgctccttaggggttacgcagagggtctaccgctaaaagagtggcttgagaaggtctgggagatcgagagaagacttgatgagagagccgtatacgtgggagcaaagcttgcatgcgttgaaatgctgaaaagcggaattacttgcttttccgacatgtacatacacatggacggtattgccaaggctgttagagaaacaggtataagagccgttctcggttacggaatggcagacagaggaagcgaggatagggctaaagaagagctcaggattgccgagcgctttattcagaaatggagtgaggaggaaaggataaagtgcatgctcacaccgcacgcggtatacacgtgctctccggatttcctgattagaatagcagactttgcaaaagaaagagacttgataaagcacattcacgcttccgaaacgctctgggaagtgagagagacgaagaaaaaatacgggatgagccctgttgaactcctgaactccacaggctttctcgatgacaagactgttttggctcacggagtgtggatcagcgaaagggacatggagatcatttcgaaagctaagacgagcgtcgcccattgcccatcaagcaatctcaagctctcttcaggcattgcaaaggtttctgaaatgctggataaaggtattaacgtatgcctgggcactgacggtgctgcgagcaacaacatgctgaac

>Bin11_68

atactatcttttccgggaatttttcgggttcagcgttatagagccccttatggttgatccaaacgtggaggacatttcctgtgacggctacgatattccaatattcgtttaccacaagagctacggatatctcgagacgaacatcaggatccctaaaactgctctggacagactcgtgatgcttctaactcagaaaagcggaaagcacatttccttggcgaacccaattgtcgactccacgctcccagatggtagcaggcttcaggccaccctcgggacagagatcacgccgagaggctcaagcttcacgataagaaaatttgcgacccagccactcacacctctcgacttgataagatttggaacaattcccttggggatcatggcttacctgtggctcgcaatagagcacaagctatctgcaattgtcgtcggagagaccgcaagcggaaaaacgacaacgcttaatgcgatactgatgttcgttccccctgatgcgaaggtcgtttccattgaggatacgagagagataaagctttaccacgacaactggctcgctcaggttacgagaacaggcattggagagcaggagatcgacatgtacgacctgctgagggcagcattgaggcagagaccggattacatagtcgttggagaggtccgcgggcgggaagcgcagaccctgttccaggcgatgtcgactggccacgccagctactctacattccatgcaggagacataaaccaaatggtttacagatttgagagcgaaccgcttaacgttccgagaagcatgcttcagtttttggacatcgtgctcgtgcagaacatgtatttaatgagtggaaggaggataaggaggacaaaagagataaacgagatcctcggtattgacccagcggacaagcacttgctcgtaaatcagtttgcaagatgggacccgaaaagagacgagcatgttgaggtgagcgttccgaagaagctcgaaaaaattgcgatgtcgagagtcgacgatgtttacctcgagctcgagagaagaaagaagtatttggagtggatgatcaaaaaggaagtcagggattacacggaatttacgaagctaatccacagttactacagaaatcctgaactggcattttctaaggtcgtatgatcgcaccgaatttcatgaaaaaatacttcagagataagctggaggcta

>Bin11_69

cgatatccgtctcaccttccctgtcttcaaaatcaaagatcaaaactggctttcccattctaaaagccctcagggcttcttcaatcataagaccacctccaattccacggtatctccgtctttcagattcagggcatccctgagcttcacttcagagatgacctcaaccacatctcctgggtaatgggttctctgcgggataacgataaaggcctgcaaaccgttaaccttgcatttaaatgcctttacgggtccgaaagttctgttctctgcagagaaaccctcaattttaattccctcctctttgtcgagccttttcttgataattaggtcttctctggcaaccctcaggttaagggtgcctggaaaaggatctatgcccagcttctctctgaactgctttctgtagccctcaagtgacacgtagtagcttccctctccaagaccgctgaaaaccgtgcagtgtagctttattgtgttcgcattctcgaatatccttcggtaatcggagtattctgcgtagagcagatccattcctttttcagttattgcgatgaactgtccgtttttcgttatcgttcgctctatcagtccgcggtcttcgagcaccttcagccttctcgaagccgtctgggtgctatgcccgagcctgtctccaaggtctttacagctgaccctaacaacctcccttgaggcgttcatcaatgccagggccttgagcatctcaatcatatcaattttgagatgcttaccgcaggtgatataaattttttgcaagcaaatagataaattaattgtttaaaattttaccctacagttcgcgtctctcccttttcagactcgaaatagcttttttcgacattcccttgtataggatgttcagatcctcaagaaacagctctgggatacttaaaactgcactccttgttggctggccgaattcatcgtaaaaacttagccacgggaacaaaagcagagacattaaaatagaaattgaaaataaaactaaaaaggcattttctccgagaaaaacaaagtttgagagcactaaacctccaaaaatactacctaaagcggaaaatatatcctgcatccagttgccgattgcataaaaaggagcagagtgttttcttcgcgtcatatctgcaacagcgttcatgaaggctatctgtggtgccgaggtgtagaagccgtgcaagatgtaggcaaggattaggaagtaaagggaaaattcgaaatgcgataggatgaaaagcgtcgcagaaatgatgaaagaaaacatcgagatggctatcaaaggcctgtttccgaatctgtccgatattgtcccagcaagccttagcgagtagattgaggaaacgtgggccacgaatgcaaaaaggagaatgagccagagcggatatcccaggacttcgagcacgtaaactgcgaagaacgtgcgtgatgcattcattgaaaagcaaaaagcagagttcaggagagtccacttcataaaattggcatccttcagcggggtgataagccttggctcgctcattctcctggacttcacgtcctcgatgttcttgaggaaataaacgctcgcaatgcctgatagaaaagctacgaagaaaaggatcggaaaggcggatttgtcaaacatttcgaataaccagatctggagtataaaaacaggtattgctgcgatttttccataggccaatcttctggaaagagtctcccccattcttgcaccaaaaaa

>Bin11_70

accaggaagttctcggaaagcctctccgcagccttgaatgctttcctgtattcttcattgacttcgatgatgtgatcagacgggattatcgcgaattttccaggatttgctctcaaaacccaatatatcgctggaagcgtgcttttggtctgaggttccaaataaacgttctcatctggtatctcgacttccaaatccttcaaatcgtcgagaatcctgaatttgtactccctgttcgctacaataaatacctcctcgggctttgaaaagatgagggccctctgtattgtcttctgaaataaactcatgctgtcaaagatttttatgaactgctttggcataagctccctgctcaatggccatagcctcgttcccttgccacctgcgaggatcaaagttttcataatcaaaatctattggttattaatatttttcttcagcctctcaattgcgctcaaagcttttccaaagatgccgaaaagtgtttgaagttcgtaatccctgagccttgctctcccaaggaccctgcgaagggtaacaagttcccttctttccctttgctttggaaagcctatgagatcaagcaattcagagactttatctatgagaatttcaagctcctcggccttcgccagattttccacccttggcacttccctcctgctgaggaaataaagaagaactgctgccgcatggcttacgttcatcactggatatgcgtcgctcgtgggtatcttaaccagcagatggcatctctctatctcctcattcaaaagaccaaagtcttctcttccaaaaagaactgcaacatcgccctcaatatattccagaagttcttcaggagtcaacagcggctttctaaagaatctgtaatctcctccagaaatccccgttgttcctacgatcaggttgaactgggaaagaaattgaaagaggtctttaacgacaacggcattctcaagcacgtctctcgcattagcagaagtcttaaagctctcctccgttactttgcaattgtagaggtagagtttttcgaatccgaagttcttcacaagccttgctatgaagccaacgttttcagggatcttgagttcaacgagcactacatggatcattcgcttagaacgtttgctaaatggggatcatggcagtaggagcatctaactcccttgccgtgagcatcatcgaccgttgctatgttcctccctgctatggtcttatggcagaccatacatgcatccctcgtgtcttcgacgagaatgctttccttagtcctgagcttcgcatgctccactccagcgccatggcatgctgcacattcaacggttgagtgttttccagaggagatcgcaatgaatgcgtcaatatggcactgcctgcagacctcgctctgaacaaaaacaagtttgttgctaatttccttctgaatccagctttcaatgctgctttcctttatcatgggagacagagcaaaggtgtaagccaaaaccgctccatatactgcgaccgcgaagagaagcacgagcagatatggcctcatagctccacctcagcatcgaggtttacgtaaaatactttcggcagggtcccggcttcaggttttagaactcttaccctgctccttttcagaagctcatagatctcgcttccctctctcaactccccaaacttccttgcacctgtgggacaaaccaggacgcatgcaggctttaaacccctcttgatcctgtgatagcagaaggtgcacttgtcagtcactttctcctcaggatgcacaaatgtcgctccgtatgggcaggcggtaatgcagtacttgcagcctatgcaggtttccttgtctacaaggaccactccctccttcgtgtgaaatcttgcataaacggggcacacgttgatgcacggagcatttgcacagtgattgcaaagctttggaacatagaatccactttcagcctttgaattcagaaatggattatccttagtgttcatcacgactttcctgccgtcgaggtagccctcaatccaggtcctcgagatcgggagctcctctggcacgtggttctcaattttgcaagctatcacgcactttccgcaccctatgcacctctcgacatcaatcaccatcgcatactgtggcgaatttttagagctcgcaggcgtagagagagctggaataaaaggaattgcaagcagggtcttgataaaatctctacgcagcatgctccacctccactggcaggagtttgtaaactaaatagtaagctaagacggccattgcaagggctccagcaacgaacatcgcctcaaacggatgtatttgcgttggtatcgaaactcccgaaatgttctttccatactgccccgcaaaaacgaagtcgaatctgaaaacgaagagcccgatgaccacaagaagggcggaaacaaataagaccctgatatttttgcccgcaaagactatcagaagcagcggaattatgagcccgagggttaattcgaagatccagaagttgagggcgaagcttccaaacaaaaggttatgcacagcgttagctgcagctaaattcacggcgggatagccaaggattatgaacttccatgcgttgaaaaggatggcgacgatcagagtgaatcccaaaatatttctcaacgcaggaagagctatcagtttttctgcagaaccctttagcaaagatgttatgactgctgctaagatcaaaatcgcggacccgccgattattgcggttattatgaagtaaatcggtgttgttgcatcatgccagagcgggatgtaaggcgcgctgaagagagctccaaggttgctatacgccaagatcgccgtaattagcgcaacgacgccgaggggcatggcaaaactcatgtcgagctccctgttcggtgttacaaggaactttccgagtttacgcagtttatgagactccacaaattcgatttttagcagaaagtctccaatgaacctgagggagaggagattccctaagaatcccctgaggcctctctgccttgattgagctatcagatcggctctgaagcagaaccatccctcgataaccagaaagacgacttcaagcacgtagaacatgatcatccagtacatcacgctcgttggatttgcgtgtccgagcagtgcgtatgttcctctctcaattctctcaaggtccactgcaatcgtgaggagtccgactgcgattgagagaaccgcgataacgatcgcatccttaacgatcacctcaagctgcctcacgtgcaggatctcagcagccgaagctattgcagcagtccctatgaggacaagaaaaacgtaaagggctacaagagacctccaaggaattccgagagcgctctcaaatggttcgacgggaatcatcacctgaaaatgcatctggtaagctccaaaaactcccaaagctatcaaggcgagcatcgttaaaatgaaaacgatctctgttagcctcaatctctcacctctccccttgctcaaatcatagctttttaagtttttcccaattgatcagggatccctcaacgaattctaaaaccgcttaaaatctttaaggcagtagtatggcccataatttctggcttttgaagaaagaagatttaaggaagaagaaaatgcttataagcccttattctctaaggaatccgtaggagaagtatggatgaaacaaaatacacctctgagggcaatcatgccaaaaactgggctaaaataattattggtgacagcagaaaattgctggaggttgaaagcgagagtgttggacttgtaataacctctccaccttactggcacataaaggattacggggttcccaatcagataggctatggacaatccctgcacgaatatctgagagatctttacagagtatggaaagaatgctacagagtcttggaacccggaaggaggctctgtataaacatcggagaccagttcgcaagatcggttgtttatggaaggtataagataatttcacttcacgctgaaatcatagcccagtgcgaaaaaattggcttcgactacatgggatcgatcatctggcaaaagaagaccacgatgaatacaactggcggtgccgtggtgatgggatcctacccctatcctccgaacggaatggtcgaaatcgactacgagttcatcctgatttttaaaaagccgggaaaacttaaaagtgtagaaaagaagattaaggaactatcgaagcttacgaaggaggaatggaaagaatacttctctggccactggaaatttggaggtgaaagacagatcggccatgaggcgatgtttccagaagagctcccgaggagactgataaaaatgtactccttcgttggggataccgttctcgatccttttcttggtagcggaactactgtgaaggctgcactcgacttggggagaaatgcaataggctatgagataaacgaggagtttttgccagtaataagggaaaagctgggtataagtagaaagcttgtggattatttatatgacattgaaataataaaaagggatgaaaaaatagagatcgaagatgtggactaccatccctcaataaaggatgcgagacccgttgcagagccaaaggcaatgtctcaaaaagccgaacaatcgcacaaagtagttgaaatattggatggagaaacgataaaaatcgataatgggctgttagtaaaatttctgggcgttaaaatcgtcgatgaggagggggctctccagtacctgaagaactaccttctgagaaagagagttttgatcagattcgagagcgatgaaagcctcgaggagggtgatgtggtttacggatacgtctacatgccgaatagaatttttgtcaacgcatatctgataaagtctggaatggcgcgcgcagatagagagaaagaatacaggctgaaggagaaattcatagaaatcgaaagaggtgtcagttttggcgaaggaatggatactcaacatggcgaccaaccgttggggcctgaataaaaaggaaagtgtaggacctgttgcagaatggatcagagagtgtgctccgaaggacgtaaaggagtggaaggaattctacttcagaaagttgagcgaatttctctcaaagaagggaatcagattaaaacccgacgaatacttggggcatctggggaagaaactctacataaagataactgaagtcataagagcggaaatcgatgaagtcgatgaggaggactgcatcgattacatttacaacctcgttatcgaaaggactttcgaagggtatcagactgaaagaaggacagtttactagcttttggagaaggaacttggggtgagattaaacctgctcccgacgaatgggatagaagatacaacgttgatttctacatagaagttgggaaaaagtacattggactgcagataaaaccaatcacgtacaaccaaacacctgaagtccacaagtggaaggaatggctgagcaaaacgcacggagaattcgagagagaaatggggggaagagttttcgttgtctacagcataaagaagggagacaggaaggagatttggaacagggaggtaattgaagagataaaaatggagattgagagaataaggaaagagtaattctcccaatgctagggaaaacttgaattttcccaaggttgatcaaaaacatttttcgcaaaatggagccattttttgcggcaataagcagtaagcgccgccggcaggattcgaacctgcgaccatgtggttaacagccacacgctccaccagctgagctacggcggcattagcgatagtcatccgtgaagacttaagagattttcggaatactgcgatttaaaaaattaaccagatctgaatatctttgttggatgatttttgagagttggccaatataaaaatttgccgtttgagtttccaaaaacatttttgtctcatataaagacttctgcaatcccaactgccaccttttctctgttttggttttctgcccaaagctcaacatcgcaaaagcttctatcttttcttggatgctttgcaacaacttttcctttgaatttgattctgtctccaacgaatattggcttaataagctttatgtcaatcgacttcagcctgccgtgtggataacaccagttagttattacctgagttaatttatcaatggtccattgtccatgtgctatcgtttttccatagcccaagagattgacccttttcgcccaaacaggatcgatatgaattggattgaaatccaaagaagcgcaggcgaatctcgttatcatttcctgcgtcacctcaacttcaatcgtgggcagctgctggtcgagttcaacctcctcaaatctcatatctcatctcctcacaccttcaatcctgtgctcctcctcgctccgcgaaaggggaagtatgagcgttccccaccatttcgcaacgatctctcccttctggttcacgaattcgttgtaataggtcaggtaacgctttcccctctttatgaacttgtcatgagccctcattttaactctgatcacatccccaacccttattggctcataaaattcgattttttgtcctggatttatcgcgccaggagtttttatccagtttccgataaaagagtcgccgaccagccagaatgcaatcgtggttaagaacatgggaggagcaatttgttcttcccaaaaaacaaggtttttatttcccgttgcctctaaataagcttcgatgtcttctttcttaaccaaaaactccctttcactccaagcctccattccaatttcaacatcctcccattttatgtacttctcctcatccaagaaaaagtcgaatgtgtaagtcttttgcttttcggtcctcaggtattttcttaccagccgctccgcttcttccgacagcctgacatataccatgatcaatctaaatgtttgcctttatatactttctccaagtgggcgcttagatttcgggtaagaaaagaatcctgccgtcgtatcttacaatctccgccttgatcccgtagacagctgcgatcacatcctcgttaatgcattccctgcccccaaaggcaaatacctttccgtccttgagcacgaggactttttccgcgtaattaagggcgagatttatgtcgtgcatcgttacgatggctgaaattcccctctccttcacagcattcttgatgagcctcatgacctcgatctggttccttatatcgagattgttggttggctcatcgaaaagcagatattttggctgctgtgcaaaagctctcgcgatgagcacaagctgaagctcaccgccgctgagttcattgagtcttctcagggcaaggtgttcgatcttcagcattttgatcgtatcctccactatctttatgtccctctcactcgcctcccaatctatgtgcggccttcttccgagcagaattgcgtcaaatacggttagaaagctaatttctcccctctgcgggacatagccaaacctcttggctatttccttctgcgacaatttgctgaagtccaatttgtccacgaatattgccccttttggcttcagaattccgttcaaacacttcagcagcgttgtcttcccgcttccgtttggccctattatgaaaagggtctctccttcgcccaatgagaaggctatttctttcagaacgtctttttggccgtaagagtagattagattttcaacctctaacacctttaccacctctcaggagaagatatatgaacatgggggcgccaatgatcgacgttattattcctacaggaattatcacgggagatattacaagccttccgactgtatcggaggccagaaggattaaggctccgacaagggcgctcgaagaaatcaaaaacctataatccccgcctataaccagccttacaacatgaggggcaaccagcccgataaatccaatgatgcctgtgaaggagacgcatacagcggttaaaaaagatgagatcagcatgccctcaagcctcaatttcctcgggtttaccccgagggatatcgcggtctcatctccagcaagaagggagttgtaatcccacctgcgattgaagaagtacaggaagcataccgcaaaggtggcgactataaggtagatctcgatccatctcgccctaccaacatcgccaaaggtccagaacactatggaggcaacctgcacgtcagttgcgaagtactgaatgagcatgactgctgcctgaaatagcgctgacattgcgacaccagcaaggattatagcttcgggcgtaagatctcgaagttttgctaaaaccaagattacgaatgcactgatcagcgaaccaagaaatgcgaatattggaactacgtatggattgaatatcgtcagagactcaccgaccctgtgcatttgtccggcgccaaagtatactatggcaatcgccgctccaaaagccgcaccctgagatatgccgagagtgaatggagatgcgagtggatttttgagtacgcactgcattacagctccggcgattgccaagcttgcgccaacgaaaattgcggaaactattctcgggattcttacgttccacaacacgggattaccattcaaaatcgaatttaaaagctcatcaaacgacatttggtatgcaccaagccccattgcgattgcggaaagaaaaataaggaatagggctaaaaatataccgaaggaaattctcttccagatcaaccttcgatactcaccagctaccgacatctattttccccaactcaccgtacttcgaggaaatctggtcgtaaagctcttttccaacgaagaacctgtatatttcattggccttttccttgatgtccgtgtctgcaaaattctgcgggtacagaacctttcccacccagtagctgttaacgagagcctgctctacgtttgtgttgtagaagttgaaggagtagattccgtacacattgccctccctgaatgcctccagggactggtagaagctcctgtcttttgagtagtcttccttcacaaggtgaaggttgccaagatcgaggaatattatctcaggctgttctttgagcaggaattccttgtctatgaaaacatgcgccgtcgtgttgaccctgcaggctatgttctcgctggttatgttgttcacctcaaatggcggaaatctgcagttcgttgactgtattccatgctgccccttgaagccgagagcgccaacgtaaaccttcgagggtttctctgcattcatagttctttcattcaggtccttaacaacgccttctatgtaggctatgacttccccggccctcttttcttttccaagcacttttcccatgagtctgatggagtcgaaaagctctgttgtgcggaagtttccgagagtgccgtagttaagcactatcactggaattccagtcttttcctgaaggttttcagcatactgggcactcacaactgcgaatataacatcaggctttaccttcagaatctcctcaacgttgggctggggatcttctgggccaccaacgccaattgttgggagctcagcgaactggggattcgcaagcctgtaggggcgggtgtatacctcccatgtcttttcggcattttccacaccaacaaccatatcagctgcctgcaggtagacgacgagccttaaagctccgggacctattgcgactatcttttccacgtttttgggaacctcaacgctccttccagcaagatctgtaacctttatttttccctcaacagtctgcttttgctcctgctgaacacatcccgagaatatcaaagcaatcagaacaaaaatggccgctaatctcttcattttaccacttcctctattttgcctttttcaaatttaacaatcccccttccgactacggcataataactcccggcgcatttcaggcatagtccatccttgattcgagttgccatcgtgtattctccgcattttttgcacctcctattctcaaaaattggggccctctcgatggggtcaacgtccacaaattcaattttgaactcatcctctggcagttcaagcatcttccagcctatcttctcccacagagcagacattctccttgcatcttcctcgctaccctttctctgaactactaccttctcaaaaagctccaaagcctctctgtcaaagtatttctccctcagcttttccgcatcaacgtagattctgacccctttccagtcctttcgcttaacaagagtcagagcgttctttccgaggtcgagatagatcaggctgttgtttccgagcgtgcagcctgttgcaacctgaactccatcagttaagcagttgttgcactccaaaattgccagaatttcctcaccaatgcttgcgatctcgtctctgctaactccaagtttatccattgctaaaaccgacatccgaacgcctacagcaacgaacgggcatacatggccgtggaattccttagctttcaacaaaagctccctcatcctaccaccttcaaaccagcctatcttagcatcctcaacaccatcaatcttcgatgaatacggcttaatatccaacacaggagtattatccagtgcatccagccatttaaccttcagaaaccttcctttcctttcaagaagctttacaactgcaaatcctatcggatttggtctattcggcgatcttgttgcaaaaaccccgtgctcccttccatcgtggggtggaatggctatgagcgtgtctcttcttgccctgtcgagccagtagagaacgatgaggtgtgagcacgtctcgatgtccttcaggccttcctcgtattcgggaaagatctcgatctcggagatctcttcagaaaaccttccctgatgcggtgcatcgccgttctttttgtaaggggatctcaccatacccacaggcttgagctcaaagtccattctgcctctcctcccagaagctctataagaacgcacgggaaactcttaccgcaattttatttattaacttcgtcgattctatcatcgcaataagccgaccttggtgttaataaatcttttcattttgttatcaatttcgccgatttgccgatactattaacgaaaaatatctaaaaattttcactctctaagccatctggcgatctccttggcgtagtatgttataattatgtccgcgcccgccctctttctgcacacgtggtattccaaaacatttcttttctcgtccaggtatctggaggcaattttcagcatcacgtattcgccgctcacgcagaaggtcgcggttgggaagcggagttcagacttcaccctgtatattatatccaggaaagaaggtttgaccattattatgtccgccccctcctctatgtctgcccttgcctctcttatcgcctcgtcagaatttcttatgtccatctgatacgatgctctatcgccgaacttaggagcactctgagccgcgtatcggaagggagagtagagaacagtgttgtacttaaccgagtacgacattatcggcacgtcggagaaaccttctctgtccaaagactctcttatggccataaccatgccatccatcattcctgagggggcgacaatgtccactccagcttcagccaagctaacagcgatttttccgtaaagcttcagcgtctcgtcgttgtctatctttccatttcttatcactccgcagtgaccgtggtcggtatactcgcagagacatagatcaccaataactatcagatcctccccggatacattttttatctcctttaccgccctctgcgccactccgtcttttgcatatgcaggagagccaatggaatccttgtacgatggtatcccgaataatatcaccgccggtattttaagctcgaggacctcttctgtcagccttgcaagttccgcaccaaggggataagtaaactggcccggcatttcttcgatctccctcttatctctcaaattctcgtcaacgaagatcggataaacgagatctttcacatccaaagagctctcgcagacaaagtccctcaacttcttgttctttctcagccttctgagccttgttctcggaaacatcaaaaccaccccgcgattatatgctacttagcttaaaaaatttcccgaagggtttctttaatcctgatgaaaccctcagctgtggagagggaagctcatgaaaactgcgaaagaacaaattgaaaaaggtatcccgcctcccggatttgaaccggggacaccgggatctccgcttgcccgcggagtggcaaactacagtcccgcgctctcccaggctgagctaaggcgggttgaagatcgtcgggccttttgcttaaaaaagttacgtttactggaatggtttcgagcatgaaaggatttatatttcatgggataggaaagcccaatcatggaggcagagataacaagagccataatagaggaggcgacaaaggactggttgagctattcaaagagtgacgtggtcatagctggcgctggaccggctggactgacagctggaacttatctttcggaaatgggatttaaaacactgatattcgagagaaaactcagtttcggcggaggtattggcggaggagggatgttattccacaaggttgcactggaaaaggaagttaaagatatcctcgagcacttcagcatcagattcgtagagaaggagaaatttctcgtctgcgacagctcggaactgatggcgaagctggcagcgaagtgcctcgattctggtgcgaagatcattcccggcgtcggggttgatgacgtcgtattcagagaaaacccgctgaggatcaccggagtctgcatccagtggagtgcggtcgagctctctggattgcatgtggatccaatcttcgtcgaggcaaaagctgttgtcgacgcaacggggcacgatgcagaagtcgttgcaattgcttcaagaaaggttccactcggaataaacgttccaggagaaagatcggcctttgcagaagttggcgaattgaaaatcgtcgaaaaaaccgggaaagttgtcgagggactttacgttgccggaatggcggtcgcgagcgtttacaatcttccgagaatgggccccattttcggcggaatgctgatgagcgggaagaggatcgcagagatcatagcgagcgacctgaaatgaagtgcagctgcggaagggttgcagagttcgtatgcaggagatgcggtaacaaagtgtgcaggaaatgctactttcatcacggactgtgcaatgattgtttcagaaaactccgctaatcgctcttttctttaaaaaaatctttaagcttctcttttacagcctccggatcttttgagtcgaatccaaagtaggaggcaaatgcctcggttgttgtcagaaccgtggatctgcccttcttctccatttttattagccttagctcgctgagcttcttcacgtgctcgtagcatttattccccctgatctttgcgagccttgcaagggttataggctgctttacagcaatgaccgctaaggtcctcatcgttccacgatcaagttccctttcggtgaatttttccacaatgctcgaaagttcgggcttcactctcatcagatatctatctccaagcttcacaatctcgatcgcagaatccatttccctgtatttcttggaaacagcttctatggccttttcaacctcttctcgcttctccttcaatatcctgcaaatttcggaaatgtccagagcttcaggagatgaaaaaagaaccgcttcgacagccttttccagcatactaagctccaaacttcttcgatagatttagcagatctaaggcgatgttgagcagatccattcccctgatacggcagagtccgccttttggagcttcaaattctgtgaatctgcttacatcgtccactcttacgccctcatgcaggattgagactaccacgggatctgcagtgtgctctccaacgcttactggggtggaatgatcggacgtaacgatgaggcagttcttcgagaaatcaagctcgaaaatcctgctcagataaggatcgagtctctctatgaactcaactttgccttcgaagtctctgtcatgtccaatttcatccggagcctttatgtgaagtaaaacgacgtcgtatttttcaagcaattccaaggatttttc

>Bin11_71

cgttaagccgagaaaagcgatagatgcaacacatgaagcttttgagttggcccttaagatctgatcacttcaaaaccatttttgtttttgcagaccatatcaggaggtcgagctcggcaacactcattcctttggcctttgccacttccctcattctgctttccgcctccatgtatctcttcctcgtcgtgcatctcagctcaagaccaagccagcgaagaatgtgcctgtcgagaattgcgatgtccgtaaagccagtatttctcagaaaatggctcgcctccttaagaccgaggccctggaattcagaaacgagaaaatccctttcaaagtgaatttcgattttattgaacttcagcatggcatggtagatgtaatgggctttttttctgtggaatcgaacccctgcatctttaagaagtctttcgagcctttcaacgtcgagcctcgagacatcgacatcttcgagagatttctggaatttaagcccagccttcgcggaggagttcgcagttgagatgcaaaaagcgagctctgtctgcaaatttgcctctatttctaaatctaaaaaaggtctgaagtcgaatttcgtagctccatactttccaagcctttcgaactccttcagcctcctccttattgcctcccttatctcttcgcttacttctggaatcatttcaatcgattctcaggtctctgacaaatactccctctccctcctccacgtagccaactctctttgctccgaacttttccacgcagtcagcggagtcttctggtacaatgacgagaaaacccattcccatgttgtatgtgcggtacatctcgagttcttccacgtttccgagcttctgtataaacctgaatatctcctgaggcttcagcggttcgtcgataacgtacttcacatccctcctcagcctcttcagctttctgaatgctccgcctgttatgtgcgcgagtccgtggacctcgcatgtcttaagaatctcgaggatttccatgtatatccttgttggcgttagcagttcttctccgatggttttatcaccgaatttatcaaaatagctcagcccgtttgcttcgataacttttcttgcaagagtaaggccattgcagtggattcccgagcttgggagagccattattacatccccgggcatgacatcttttcccgttatgatcctgtccttcttcacgactcctacagcagttccagccaaatcgaagccccttatcatgtcaggaagcgttgcagtttctccggccacgagcgtgatgttggcaatcctgcaaccctcctccagaccctttgcgatctccgcaaccacggcctcatcgacctgctgcatcgcaatgtagtccaccatcgcaagaggttccgctccaattgaaagcaagtcgttgacgtttgcggcaacacagtcgatgcctagcgtgtcgaatttgttcattgcgattccaacgagtatcttggttccaaccccatctgtggtgatggcgatcccgaagtcaccgcagtcaatgactcctgcgtaatgcgagaccagaataggtgctccaaatccccttctggtgaacttaacgatgcttgtaagcgcacctatcgccttttcagccctgcttatatctaccccggcctttgcgtagtcgaacttcatgcctttagttggacagctttttaaaaacttattctacctctgttatggatcccttcgagcttgtcaggctgatggaaggcgaggccaagaaaagagatgctcccgtatttctgcacagcaagaaactcgaaccgtggcagatcctgatagcaacggttttaagcgcaaggactaaggaccagcagacggctgaagccgctgaaagacttttctctaaggcaagatctcttgaagaactcgcgaagatgaacctggaggaaatagaaaggctgatcagacctgttggattttatagggtaaaggcgagaaggattaaggagatagcggaaattctcaggggaagaaattttcccgggactctggaggagctaatggcattgcccggcgttggaaggaagaccgcaaatatagtcttagcctacatgggaaaacctgcaatagctgtggacacgcatgtgcacaggattgcgaacaggcttggaattgtgaataccaaaagaccagagcaaactgaggaggaactgaggaggatctttcccgaagatctctggaacaggttaaattcggcatttgtcggctacggtcaaactgtatgtcttccgaagaagcccaagtgcgaggaatgcccgctgaggagtttctgcaagagctacgggactggataagatttttaattttaatgctgaaatcgatgcatgaaggttgctactttcaacgtgaactccataaacacgaggctccacatcgttatcccctggctaagggaaaaaaggcccgatttcttctgcatgcaggaaacaaaggtcgaggacaggaagttcccttctgctgacttccacaggctcggataccaggtctatttcaggggtaaaggcggacagagcggggttgcaatcgcaacgctgagagagccgttgaaggtgagctttggcctgaagggggaggatgaggacaggctgatcttcgcggattttggtgatctcaagcttgtaaacgtctacgttcctcagggcttcaatatagacagcgaaaaatatgcctacaagctcaggtggctggaaaagttctaccagtggctcaggggtttcgatctgaacgaaaacatcctgatctgcggtgacatgaacgtggctcctgagccgatagacgttcacagtcccgagaagctgaagaatcacgtatgctttcacgaggacgcgagaagggcttacaggaagatcttagaccttggattcgtcgatttgctgagaaaaatgcatcctaatgagagaatttactcattctacgattaccgtgtcagaggggctgttgagaggggtctcggctggagggtcgatgcattactagcaacaaaaccgcttgctgagagatgcatttactgcgatgtcgatttaaagccaagacttgctgaaaagccttcagatcatctaccgatcctcgcagagttctcctgagcctttttgcaaccttctgtccttggggacataattcctgatcttttttccatcgccctttccgcaccccaagaaatactcaccccatttcagaatgcagtatccagtgtagctcccctctatgtcctctccggaaagccatttaatcgccttttcctcgtccaattctaaaacgcccttttttgctaccttcccaacgataaagcttccctcgatcgaaagtcgcagaccatctctttcgatttttccgaagtatagacccgggctcttactcctcagcggaaagtcgcacctcttgcgtgcgtatattcttccctttccgcctaaagagaattcgagctccaattcagcgtcgaactgactcttaagcagctcccttattttctcaacttcgcaacgaagaatccctccgtgtcgttcagctggggatgtattctgagacattttctcacctctcccagatattcccttccctcaaattcggtgaacccctccattgctttcacaggaagatttatccgctcgacttcggcttcggtattccttaacaggaaatccacaacttcttcgttctccatcggctcaaaagtgcaggttgagtatactactacgccccccggcttcacgcacctgtaggcagatagaagaagctctttctgaagcttcgagagaccgatgccgactttgatattccatcccttcgcgtacgaaaagctcctccttatcatccccagattgctgcagggagcatcaacgaggaccgcatcgaacctgttctcaaatcttgcaaattttctgccgtcggacatcgttatctttgcgattaaaacaccgcatttctggatgttggagaccagcatgtttatacgctcgatcttaacatcattggcaaccaagcaacctttgttttccatgtactgtgctatctgtgtcgtttttgaccccggagaggcacagaggtcgaggacgagcatcttaggctcgagctccatcagtagaggaggaatcatggaaactgaagactgggaaaagatgatgccgagctggtgctcaggaatcgcggagaagtcctcaacattcacgtagtatccttcatcacaccagggaaccttctccaccagcacttctgaaagcctccttaagacgtagtccaggtctgctttaagcgtgttgattctaacactctttctgaagggcttttctaagtaatcaaaaaactcctgagagccatcgatcttcgatagtcgttcgaacagtgcgggattgagctcttttagcataatgagtaggcaggacattcctcgaacgtgaacttgacggtctgaaagtcccgtctgaaatccgcaacttcccttcttatctcctgaaggctctttttatctctcagcgtcatgtcgatgaattttgcaatttcgaccatctcttcctcctccatccccagtctcgtcacctcttgcaccccaatgcggatgcccgagggattcgcagttctatctatggaatcccagggaagaaggtttttgttcaggattatcccaacagtttcaagctttcttgcaaccggatctccccctccgaattcccttacatcgacagcaatctggtgcgtttcggtaaagccgaacttttcgccaagaaccctgtagcccaggccgtgaagttcttctgcgagctttctcgcattccttaccgtctgccttgcgtatgcctcgccgaattggagcatctccattgcggctaccgcataagcggctaaggaatgtagatggtggttgctgacaacgcccgggaacactgctttgtctatcctcttcgcaaccctttctttgctcaagatcagcgccctctgcgggccgaagaacgttttgtgcgtcgaggcagtgagaacatcggcttcttccttgatcggattcggaaaaacctttcccgctatcagaccgagcacgtggctcgcgtcaaacatcacgtaggcgtttaaatcctccgcaatctccacgatctctctgactggctgcctgaaaagtatcaggctggagccgagaatgaaaagccttggtctgatctcttttgcgagcttctccgttgctggaacatcgatgttcatggcttcgacgtcgaacggatagtaagaaactttcaaaccccttaatccagctgcagatatcctgtcgtggcttatatgtcctccgcatggaacagaaatgctcaaaaccgtgtctcctggattcgtaagtgcaaaaaatgcggcaagattagctaccactcctgaaattggctgcaaatttacgtgctctacgtcaaaaagccttctcgtcagcctaatcgccagttcctctatctcatcgatgtacttgcatccggcgtagtatctttctccgaccttgccctctgcatatctgtgcccgaaatctgaaagatagcattttctaacgagttccgaggtcatattctcgctcgctatgagagggattgagcttcgcatcatttcattgtgcttctttattatattgaaaacttccgaatacatgcgaaaagcagtttacgagaatatttaagttttcctcgtgactgaggggtgaaccgaaatcgatttaattcagcaccctgagcatttatgtggagagagtcgaagagtatctcgaggcgatctatgacatacagaaaagcggaagggtggcaaaaactggagatctggcaaggattcttggtgttaaaccgccgagcgtaactgaaatgctttcgaagcttaaggagatgggatacgtcgattataatccttacagaggagcgattttaacgaaaaagggtgaggaggtagcggagaggataaagaggcactacaggatagcttcgaacttcttcaagcttataggtgtcgatgaagaggttgcggaaaaacttggatgcgaactagagcaccatatggacgacgaagttgcgaataagctttcctccattctcgaatacagatgcggaggatgtgagagggatataaagaggttaacctgcgtttcagacggaatttacgaagtagtctcttctccagatccggagctgaagcagggagagcttctgagggtggtaggcggaaaggctgaaacgctcgatggtgcgcgggtgagagaagagatcatggatctcgttctcgttcgcaaattttcatagcaaatagcttctcgaagaagagctttttctcgccagcgatctcgtatctgaagcctcttgaaagcacttcttcgaaaatcctcggcgtgttcagcgaggagaagatcagaatggccctcccttcttcaaccattatttcacggagagagtcgagaaatctgcaaataacttccagcccctctttcccaccatctatggcaatgtcttcgagttctccccttctcacatcttcttcgagctcaatgtaaggcggattgaagagaactagggtaaaaatcctcttcacaccccttgcgaggtctgttctcaagacttcaagccccctctttttaagctcctttaccgcgaaaggagatatgtccgttaccacaagaagtctgcattttcccaagagcttctcagctacaaatccgcttccagccccaatttctatgacatagtcgtcctctcttacctctctcagtgcagtctccaagagcagttcgctgtcctcagcaggctcgtagatcatccacgatcctcgcgaagttcgaagctccgagctcttcgggacgcatatccgcgaattctttgccaaggttgaaagaaaatcctgctctcttttcaaattctctgaggatctttccaagcttttttctcctcatggagaacgcaaaagtgacgaaatgctcaaaaagttccctgttcttcacctctacggtgggcataggttttagcctgacgatggctgaatccaccttaggcacaggcctgaagttctctctgccaacgatctcgagtaactctgctctgcagtaggcctttgatatgactccgagtctgctgtcttcgcctacaagcctctccgcaaattctcgctgaagcattattactgcaagcctgaatccatgcctcagaagtttgaagatcagcggagaggagatttcgtagggtatgttcgaaacgaatttggtgaaaggcgggaattccgccttcagggcgtctccttttatcagcaatagctttccactcgctatctgccctgcgaatctcttttcgagcagagcaacgagtctggcatccttttcgataccgatcactttggccttcgaaagcaacgcttccgtgagattcccggttccacagccgacttcaaggacaacgtcgtcgctttcgagttcggcgtagctaaggatcctctctatgatcgccctgttcaccagcatgtgctgacccagtctcatagagctttccgagctccagaccgatttccaaccaagcccaggcccatatcacgcactcaaaggccctgataaaatcggatttcgcgagaaaaaactcagaatcgcttgtatacgctctaatgttctccaagaatcctttatctccttttacctcactgagtttctccttcagccttctcagccacttttctgtctcctccttcagctccatcaacccttttcacctcaagggtaaggtctctcctttcaatgacaactatctcgtcccccttcctcagctcgtcctttgactcgatgctccagatctctcccctgacctttgcaaaaccctttccgttcctgaactcgataacttcgcccctcaaccccgcaacttcccccaccgagcttctctttctcctgatctttatgatctttatcagaacgaatgtcataaaactggctatcccaatcccgagccctactgcgagcttcgggaaaacttcgtagaattcattcggcatcatcggctcctcgaccagcattagcaaacccatggttatcgagatcaccgatgccacggctaaaggaccgtatgtgggcgttaaaagttctgcgatcaggaaaagaatccctaagaggattaggaagattgcggcgtaatttatctgtattactcccagacctgcaaaggcgagtatgagcagaatcgctccgactacttcagccatcattcccggcgacgtgagtccaaagatgagaaggtatactccgagcaggagcaggattaccgcgatctgagggtttgagatgatgtcgtagacaacagcttggagtggtctctcgatttcgacaaatacataattgcttgtgttcaggacaacttctttgcccgctatggagatcttttttccatttattttctccataagctcttctctgctgtccgcaagcagatctacgactttcagctcgtaggcttcatgcgcagtaaggctaagggcctccgtcacgaaacgttcggctatctc

>Bin11_72

cgacaggacggctgagatgactcaggagaagatgaagaggttcaaggtcatcatggattcaatgacagaagaggagctgctgaatcctaagatcatagacagctcgagaataaggaggatagccataggtagcggcacgtcccagcaggaagtcagggagcttttgaggtattacgagactgtaaagagcttcatgaagaagatgaaaaagaagaaacttccaattaagggtcttaagctcgggatatgaaagccgggatagatgaggcgggaaagggttgcgttataggtccactggttgttgcgggagttgcgtgcgattccgaggaatatctcaaatgcatcggagtgagggattcaaagaagttaagccaaaagaagagagaagagcttgcggagaagatcagagaggttgcgagggttgaagtgataaagatacctgcaaataagctcgacgaaatgatggaaacgaagacaataaacgaaatactgaaggagtgctatgctgagctcataagaaggcttaagcctgaaatagctctcgtagacagccccgacgttaagcctgaaaggctcgcatcccagctcagggaaatgacaaacgtggaggtcattgcggagcacagagcggatgataggtatcccttagtgtcttcagcttcgatcattgcgaaggtggagagggatagggagatagaggcgctaaagcagagcttcggcgacttcggaagcggatatgcaagcgatccgagaacaagggattacctgaaaaagcttaaggagattcccccattcgttcgaaagagatggaaaacgattgagagactttcacagaaatctataaccgattttctttgagcttatccttggagctctccagctctttcagccttttgagaaggatctccctgatcgcaagcaggtcttctctgagcatttcaagttctctgatcttttcctcgatctccttcagctttctctcgccatacttcagcgtcagctttatctgctccgtttctccagcaacgtcgtacatatcgatcatctccttaatctcgctcaaactgaagccgagcctttttccacgcaggatgagcttcagcctcgccttgtccctcttcgtaaaaattctctggtttcccgaagttctgcccggggatagaagaccgagctcctcgtaataccttatggtcctcgtgcttatctcgaactcacgggcaagttccgaaatcgtaaacgtttccccttcttcacccgggggcatcgaaatcgaataactccgatagcataaaaatatttctgccatataaataacgttaacgtaaacgtaaaatttaagtattgttcgtccaaaaatggtgtggtgagagaatgctcgaaaacgatttcttgcttaccgaggaggaggcaaaacttcgtgaagaagtgaaggagtttgccgcttccatagatccagagctgattcgcaggatggacagaaatgaagtggattatccattcgaattcatcaaagcctgcgcagaaaggggtcttctgggcttgagatttccaaaagagtatggaggcaggggaatgccctggacagcagaaaccgtcgctcttgaagagattggagttctcggaatgggaattggctgcgcgtattcgatggtcagcatagttggcgaggcgctttacagattcggcaacgaatggcagaaggaggaattcctaaagccgatagtgaagggaaaaaagatctctgcagaggggctgacagagccgaggggtggaagcgactttttcggcacaacaacgagggcggagaaaaaaggaaacgtatgggtgctgaatggctcgaagaggttcatcgcgggcggaaaggttgcggacttttaccttgtatatgcgagaacagatccgaacgctccaagccacaaggcgttaacggcattcatcgtggagagagatagaggggtggaaatcgaggaaatctacaacctgatgggcttccgtggaatgggtacggcgagaatagttttcaaggaccttgaggttccaaaagaatacgttatgggggaggtcaacaacgcgagggtgatcttcaacagaatgatgatccccgaaagactcacatcagctgcaggctccttaggcgtgagagctgcgcttgaggttgcaatgaagtattcggagaaaaggaaggcttttggcagaaagatcagggaatttcagggcgttaacttcatggttgcggaagcgcttacaaagctcgacgccgcaagggctctcgtctacaacgctgcaagggcggtggacctctacgatcaggggaaatcaacgcttgacccgcgaagactcgtgagcgaggcaaagctttttgcgaccgaaatggcctgggatgtcgtgaacaaggcaatgcagatccttggcggaataggctacacccaggtatatccagtcgagagaatgctcagggatctgcgccttggcttgatatggaccggcagcaacgagatcatgaaagtcctcattcagcacgaggcctacaatctcatcgggcttccctcaaaggacagggattacgagaaggacgcgatggactggtataaggagtgggaaaaagtttacgagtgaacttctattatttctcctttttcgaccgcagacttttcgagttcctcaaattcctcgaaatcctcgacgatttctcgttttgcccttgtttctggatgctttgcaaccatcagggagcagcaatcttcatatggaagaattgaaatctcgtaggtgcctattttctttgcaagttccacaatctcttctttgtcgaaacccaggagcggtggaagcactgcaagctttgaggcggcatagatcacgttcaggttctcaagcgtctggcttgcaacctgcgagatgttgtctcccgtaatgatggcctttgccccttccttttccgccaccatgttcgccatcctcatcatgcttctcctgtaaacgaccattctgatcttcgggggcactattcggattatctccatctgaacatcttcgaatggaatcatgtagagcctcaggcttccctgatactcggcgagcttctccgctagcatcaggattttctttcttacctccttggaatggagggttttgttgaagaagtgcaccaaaacagtttcgcaacccctcttcatcgccaagaagcttgcaactgggctgtcgatgcctcccgaaacgagcgaaactactttcccggcgcagccaattgggagtccgccgattccctcgaccctcttcgaataaacgagtgcgaatttctcaccgatctcgacccaaacggtgacatcgggattatcgaggtcaaccctttttccagttctctcaacgacgactcttccaagctcccggtttatctcaacagagttaagaggaaaattcttgtttctcctcgaggtagaaattctgaaggtctcaaatttttctgggagaaccttgaggacggcagactcaattgcggacatgtccagttccgttttatagccgactccaaagtacctaattccaggaacttttctcagcctctcctcgattccttcatagtactccacttcgatccatccatatctcctctttgcctttgcgaatctgctgatgttctccacgagttttctctcgaaaaatgccctgttctctcccttggtcccgatctctccgtagtgcacgatgcagatctccacgagcagaatccccacaaagatatttaaagagttctgccgagatctgacatggatatcctcactgcagcggagaagataaagtcgatggaaataaggggggcttcgaggatcgcgaagttcgcagcagaggttatgaaagagcatgcgctgaggataagggaaaactttgacgaagagatgctgagggcatcgcagatcctcctgaacacccgtcccactgctgtgagcctctacaatgccgtcaactacataatgcagtatcgaggggaaagcatagaagagaaaagggaaagcgctgtgaagagggccgaggagttcattggctgggttgagaccgctcagaaaataattgctgagataggagagaaaaggataagggacggctctaccgtactgacacactgcaattcatcagcagctttagccatcataaagagagcccatgaagttggaaaaagagtcgaagtccttgccacagagtcgagaccgcgctggcaggggcacataactgcaaggcagttgagagaggcaggaatagacgtcacgattatagttgactcggctgtgaggtatttcatcaatgacgttgactgcgtccttgtgggtgcggatacgatcacggcaaatggagcactcgttaacaaaatcggaacatcgcaggtagccctctgtgccaaggaggcaagagttccttttatggttgctgcagagacgtttaagttcagcccgaagacgctgtttggcaatctcgtggtcattgaggagaggtcgcaggaagaggtagccccaaaggagctgatcgatctcggggtaaaggtgaggaatcctgcctttgatgtcactccgaggagctacatcgacgttataattacggagatcggcgctatacctccagaaatggcatattttgtcattaaggaaaggctcggatatgcgatgataacagctgaggaactcagaatcgatgcggagcactacgattgagctcttcataatttcgacgaaaagcataacaaaattttctcattagtagtaaatttaggtgcagtctcttcgtacagcacaaggtttatatagtttgacatactcataattgtcatggtgtttagcaagctcatatcagaggctgaaatagtgctgagacatctggaagtcttgcaggcagtcctcgagaaacagccgatcggtatcttcaagctttcggacatgctcaacatgccgaagcacagggttcgctactctctgcgagttctcgagcagtccggcatcatagtgccaacgcagtatggtgcgatggtgaaggacgagggttacgacaagatagagcagctcaaggcagaaatggaaaaaataaaggggcttttagctcaaatagaggaaaagattcagaagctctaatctactatctcggcaaagccgtatttttttagaaccttagttattcttatcttcacgctgtcgttcagctttgccccgggcacgaaaactacgaagccttctatctttgcaatcccatctccttcactacctacagcctcgattttaacttcccttacgtctccaaccttcacgggtggtttcccaaatccaatttccctcaaatctaacacctcacattcgaattttattaacttctgcatggcaatatataaacttttccacaaaatttgaggcacagtttttggccgcgacctatcaaaatcaaaaattttaaattgtccttaattgcattttaatcatggctgttgtgtttccttctacggactggctaaacgaactgaagcagagggttaacagcgacgaactctacaaaaaggtcgctgcgaactgggagggggattacctctgcatcgtcgagatcgatcaggaagccctgagggatttccagaacccgaagattcttggtgggttaatttcaatgctggcggcgatcccgaaggatagaagggcgagtttcagggggacaccaacagagaaattcataaaaatgctcggtctggagctggatcacgatttagatccctcgaagctcaatctggaggagatggcaaaaaaggtcgcgcagataaagctcgaagacataaagggtgccgcaacgtacatttggatggacttctggcacggagaaatgaggcactcagagccagtagctcctcaggatgtaaagaatccgagatttatcctcagcgggcctttttccgcgttcaaggaaatcgtgcttggaaaggtcgatccaacgacccagatcatgaggggcaagctcaagctgaagggagatctgggttacatgatgagaaatacggcaacggtgaacagattcagtcagctgatggcctcggttccgatagagaagagttagtgggaatatgtggaccttcgtctcgccgaggcttatcgtttacggagatgacgctctcacatttctcgaaggcgaaaaagcaagcagggttcttatagtggcagacgaatcaatattaaagcttggattcgctgaaatggtcaagaacagcctcagggctgaaaaaatagaggtattcagcgaagtggaaccggagccgagcatagacactgcgctcaagtgtgcgaaattggcgagggaacttcagccccagctgatagtcgctgttggcggcggaagtgttatggacgttgcaaaggctgcgagaatcctcatggagctggatatagatccgatagcaattacacccttcaccgatctattcgagcttggttacacgaaaaaggcaaagctgatcgcaattccaaccacgagcggaacgggtgcggacgtaacctggacttccgtccttacagataaagtagagcacagaaagctaacgcctgcaaacaaggaagtagtgccagatgttacgatcctcgattacagattggttgcgaccatgccgaagaagctaatcgcaggctcaggacttgacgctcttacgcatgcagtcgaggggatcgtgtcgatttggaggaacgactttagcgatgcgctctgcgaaaaggctgtcgagatcattctgaataacctagaaaaatcgtacatgggtgatgctgaagctcgggcgaaaatgcacatagcagcgacaatggctggcttaggtttcggaaattcccaggtcggtctggcccactcactgggacacacattcggagctatattcaaggttcaccatggggtgagcgtgggcatctttctgccatacgtgatgcagttctacctgaagagtgatgcaagggaaaggatggattctcttgcgagaaaactgggtcttgaaagtgccgagatgctcatagacaggatttttgatttgatgaggagggttgaactgccgacaaggctttccgaattgatcagcagagaggacttcgagaaaaatcttgagagcttagttatgaacacgctgaacgactccagccttggaatgagcccgagaattccagattacgaccagaccaggatgatctacgaatacgccttcgaagggaaaagaatagatttttaggggtggaagatgttcgcgtactgtggaagactcctgagggtaaatttagcggatagaagcacttctatcttgcccttaaacgagagggatgcaaggctgttcgtgggcggagctgggatgggcataaggattcactaccagatgaggacttacgaaaaggatccgttatcagccgataatccgatcgtgctcatgactggccctctcaccgcgacacctgctccggcaacgagcaagctcgccttttgtgccagaagtccgctcacgaagatctggggagaatcgaattctggaggaaaaatggcagtttatctgaaatacgcgggatgggacggtttgatcattgagggcaaaagcgagaaaccagtttacctgaaagtggacaaaaacggtgccgagctgagggatgcagacggaatttgggggaaggggtgctacgaagctcaggagacgatagagaaagaactgagtgaaaagaggacttccaccgcggtaatagggcctgcaggagagaatatggtaaaatacgcgtgcatacaggtagataattcaaggcatgcgggcagaacggggatgggagctgttatgggatcaaaaaagctgaagggcattgcagtttgctacgatcccggagaaaagtcagaaataaagctttccgacagtgaaaaattcagggaaactgtatcccagttaatagagacgataaacaacgatttcacctgcaacatgctcagagaactcggaacctcgggatacgtcgagaatgccgaagcttttggagacctgccggtcaaatacttctctcagggacagttcccctccgcaacaaatatctcaggttctgcaatggccggctccattctgaaagggaacgacggatgcttgggctgcatagtcagatgtgggagagttgtggagctgaaaggaaggaaaattcacggtcccgaatacgaaacagttgcatcgtttgggtctctccagctcaacgaaagtctcgatgccatcgtagaactgaaccacatgtgcaacgatctcggtctggacacgatctcggcaggtgtctcgattgccttcgcaatgtggatgacggagagaggcatcgggaactttggagtcaggtggggggatagcgaaagagtaaaggaacttgtaagagagatagcattcaagcaaggcgtcggttccgagctagcagaaggcgttaaattcctggaagagaagtacaacgttaagggctggggagcgcatgtcaaggggctggaaattcccatgcacgatgcgagggccttcgcttctctcgcatgcgcatacgctgtgcacgtgaggggtgcatgccacctaccgcatcagatgtatctttacgaaatgggaaaggtcatcgacgaatacggcataatctcaaacgacagatttgcaaatgaaggcaagggagttcttgttgccaaagttcagaacttcacggagctcttcaacgctgtgactatgtgcgcctttatgcccgtaaccccaacgatgctcgcaagtatgctcaagttcgcaactggcttcgattacgatctcgagagcttggccaggactggagaaaggatttttacgctcaagaggctttacgatctcaagtgtggagtaagggctgaggacgacaggcttccgcaggtagttctgcagccccttgaaggtggcagtgcgggaaacgttcccgacgtgatgaggcagttgcaggagtactacgagtacagaaagtggattaacggggttccgagtgaagagaagctgagggagctcgagatagattagtcgatctccacaatttttttcctcgatcttaggcctgcgactatcctgatccttgactttggcacacgaaagtactcggaaagagcctctattagctccgaattagctctgccctctactggatgagccctgactgaaactacaaactcgtcttccaattgctctacctttgtctctctcgcatttggtctcactctgacttttatcttcatttcactctaccgtagcttgccgtgaattctgagaactgcaaaacgtgccctttcattgcattttcgagatcagccttagttgcggaagggcctaagcttagagtagtgtcaagaacgtaaagcttgaaacggtatttatgaattccgctcggtgggcaggggccattgtagccaaatttcccaaaatcgtttttgccctgaacagccctgatcggactctcaacgatgggctcttttggaattccttcaggtatattctcagtatctggaggaatgttccagataatccagtgggtgaacactccaaagggggcgtcgggatcatctaagatgattgcaaggcttttcgcgttttccatcacgccgtagatctcaacaggaggggagatatcttcgccgtcgcacgtgtatttcggtggaaaataagaaaatggaacggaaatgtaaagatcgggactccttggaatctcagctctatctgcacagcagattaccagcatcgaaagcacgagcaagggccagagtctcataaacagataaggcccactcaatcaatatatttttctatcagaagccgacagtttccgcgcttcccgggacctctcggaacccagtacaacgcggaacggaacggggcttaacttccgggttcggaatgagtccgggtgtttcccccgctcctatgaccgtcggcaaaacccccttaacctttcgatatttaatcctttcgcccgagaattctttcttctatgctcaaccttctggccttaaatatatctccgacatcctttctcagcagataccatatcacaagagctgcgatgaggacatttattactccgaggggatcgtaggggagaaggaggacggaataagtaagctggaatgcgaaaaggattactgtgagaattctggcccagtttttcagcatcaagatgccagttgcgcttaggagaaataagagggcgagtgccacgatcacaaccgagacgaggtaatagagctcctcgaaaatactgtctggaagttcataaccttcggcaacggtctcaaactccttctgaattatcggaagcacttcttccttgaataagaccatcccgagacctgcaagaatgtaaattgcggcgtttaggattagcagaattgagattgcaagtatcccgaaattgcgcatgcgagaagtattctctatgaagataaatctttacccatcaaaatccactgtctcgagaagaagtatttttcagcgagcttgttctcaaagaagccgtccacttcgaatccgagttttctgaaaaaagccacagcatcgctgtcagcaggaagtatgcagctaattcgacgcatcttttttcccctgaggtctccgatcactttctccatcaaagcggtaccgataccgctaccgcgtctcagtggagaaacagcaaggaaaacgagaaaacattctccctttttcggttttcttaaaaagaatttcagaagcaggtaagcccttaggccgtcgatgagccccaaacgttcccttaaaaaatttaaaggattcaccttttggatcataacccatgtgaaggccaagatccgcccatcttctgcaacgtagcagccttccttgttcatcttaaaaaattcaaataagatttcccttgcaacgtcgacgtccttaaagattgcattcagctctctatcgcatgatagggccagaactctgactgcatcgacgaggtcttcatccctgagcttcctgatcttcatagtagcgctttatcctatctactgctttccttagattttccattgagttcgcatagcttatcctgacgaaactatcgttccagcttccaaaggcttttccgggagtaacggcgacaccgtaatctataagcttttcagagaacttcatacagtcttcccttacgtctatgaacatgtaaaatgccccctctggttttactgtccagtatagcttgctgagctcaccgtatacgaaatctctcctctttctgaactccgagaccatcttgtccacgataaagtcaaactccccgctctgcatcacttctgcgactgccttttgagcgaaagcaggagcgcagactccgtttacctgatgcaccttcagcattgagtccattagctccttccttgcaattacgaaacctattctccaccctgtcattgcgagtgattttgaaaaaccgtttacaacaacaacgttctcgcgccccgcaagggaatggggtcttttatcgtagtatatcctgtcgtagatctcatcgcttatcacaacagctccatagtccgctgcgatcttcacgatctcctcggcagatctcttgtcgatcacagccccagtgggattattcggatagttcagaaacacggcgctcacatctctgctcataacctcggcaatgctttccggagtcggaacgaattcgctttcgtgcgtctttacctcctctatctttgccctgcagagctttgcataggtgaagtagctgagaaaggagggtgagggaatcacgactctgctgccttcctcaatgaaggcaagacttgcgttcatcagggcctcgctcccacctgttgttaccatgacctcttcctttgacacaccgtatcgctcggcaattgcaatccttagttcctctatgccgaagttcgatgtgtagtgtgtgaagccctcattcatcgccctacaagccctttcgattacctccacaggcgtcgtaaagtctggctccccgattgtgaggctaacgatgtcctttccctctctttttgccttctccaccacttcgaacatccttctaatcattgagctcgaaatatcccttaacctggtcggctccattgctgccaatgcaattgatggatgaaaatactttcgactcctcggttcgaaaccgttatttgccctgaagccctttggaaatcatggatttcgaaataactgcaaaggatgttcttggaagaatttgcaggctggatacacctcacggaaggattgagactcctgcggttctgcccgttataaatccgaacatccctttcatccctccgaaaaaaatgagggatttcggagcccaggctttgataacaaacgcctacataatctataggacgtcgaggagagaggccatggctaagggtgttcacggacttctcgaagtagatttgcccataatgaccgacagcggtagctatcagctcatgctttacggcgacgtggatgtgtccaacagagaaatcctggaatttcagcagaaaattggttctgacttcatcattcccttggacataccaacaccacccgacgcggacttcgaaacggcaaaaaaggacttggagattacgatcggaagggagagggaggcaaaggagttgaagggcgaatcccttctcgttctgccgattcagggctcaacgcatgcggaactcagacgggaaagtgcgctgatggcgaagaaaattggtggcgacgtttatgcgataggtgccgtcgttccactgatggatgcgtacaggttcagcgatctcgcaaggatcatactcgaagtgcgttccgtgttaagagttgaacccattcacctttttggctgcggccatccaatggtcttcgccttggcagtagctcttggctgcgatctcttcgactctgcggcatatgctctctatgccaaagatgaccgctatctcacagtctacggaacgaagaagctttcggaacttcagtatttcccatgcagctgtcctaattgctcaaaaatggagccagaggaggtgagagagctcgagaaaggcgagagggagatattcctcgcagagcacaacctctacgtgactttttcagagataaggacgataaagaacgcg

>Bin11_73

aaatgctatccttcggttaaggatattcccgacagcgtcgatgttgcgatcatagcagttcccgcgaagctcgttccccaagttatgaaggactgcgcggagaagggaataaaaggcgttgttattctgagctccggattcagcgaggagggagaggaaggagcaaagatcgaaaaggaggttctcaggatagcaaaagaagctaagatcctcatattgggtccgaacaccacgggaggattcaacaccgaaacggggttcataacgagctttgcaccacttctgggcgttaacaggggaaacatagggctaatagcgcaaacgggattgtttttaggagtgctaatggtctcaattttctcaaaccacccgaatgtgggtttcagcaagatcatcggaatggggaacaagatagatgtgcaagaccatgaggcgcttgattttctgctcagggatgaaaaaacgagcgttgtagggatctacatggagggtataaggaacggaagagcattttacaacgtcgcaaggaatgcaaagaagccgatcgtgatcttcaaaactggaagaaccgaatatgggcagaaagctgcgatgagccatacagcttcgatttgcggcaacgacgatatcttcgatgcggtctgcagacaggcaaatctcgtgagggtttacagcttcgacgagctcctaaatgtagcgaaagctttcgcatttcaaccacttcctaagggtaatagggtcggaataattcactacactggatctggctgcgttcagtctgccgatactgctttcttctcggatttaaagcttgcaaacttcaaaaaggagactgtggatagaataaagtccgtaactccagagtggcatggagtaaacaatcccgtggacatctggcccatgatagagtactttggtgttgaaaagacatacaacacagcaatggaggcgattctgagcgacgaaaatgtggattcgatggttgtggcgatggatgtgggccccttctggggggactacaggccggacttcaaaaggctaagatcttttggcaagccagtttacttcgtcctcgaaggacacagagaccttgttaatgagcaaaagaatatcttcgaagaaaacagatttcccgtttactcgaatgctatcaatgcgattcaggttcttggaaaggtgacgaaatatgctttgaaatatgctggtgcacgaagatagaccagaatacgatgcttggatgaaaatcatactctcttttgcccctgcagtaatatttttgcttctttttctgattcattacaatatcctccctcctgaaaacgaagaagaagccaggatggcaaaaatggttctcttggcctctcttatcataattttgttcctatactgggcaataatgccaaggaaatacgagatacatgaagaaagaataagaatcgttcttggcgcattttc

>Bin11_74

ctcgaaagaggccatttcaacgacataattcttcagcgagattattccgctccttctgaatcccgagttgttagcgaccgtcagcagattcttagcggaatccagatccctgcaggcaacatgtattattggcggatcctgaatcagccaaccctgacgtttacagctacgaatacactcgagcacatcttcaaatatggctaaattgtgccactttcccagaaatcgcgaggattttttatctccaaagctttcaaggtcgatgacagctatcctaccgctgcagcttgaaagcgttacatagctctccttaccgttaattctctccaagagtggaattatatcctcgtccacctttccctcctcttttgccttctcgtagcacaccaatctttttttcctgtactccgaccacatcacttctttgaaagcgtcgaaacgtaggccaaaaatgctgaaagctgaattctttcgtttgctccctccgtcagtctgaagtcgatctctccaagcctgtcgacgagaagaaccttcatcttttcgtttatattcgaattcgtgatctccctgaagagttgcgcaaccacatcctccccgctcattccgtattcgagcatgagtttgttcagcttttccctcgcctcttcaaactttccgttcagcgctgtttcgagaatttccgcgatctcctggggctgggcggtggcagtgatctgaaagatattctcccggtccacaacctctccaattgccgcagctccctgcaatgcgttgattgctttcctgaaatctccacccgagacgtaaaggattgcctcaatcccatcttccgttatcttaactccttccttttcacagatctccagaagtctcttcctcattgcctcctttggtacagcctttaatctgaagacggcacatctgctttggattggctcaataattctgctgacgtagttgcagcttaaaatgaatctgcagcttctcgaaaacatttccatagtcctcctaagcgctgcctgagca

>Bin11_75

ccatggactgcccactgaagtaaaggttgaggagaaatacgggatcaagaaaaatgaggtcgaaagggaaaagtttagggagctctgcatcaagttcactgaggagaacatagagaagatgcgcgctacagcgaaaagactaggtttcagcatagactggagcaaggaatacataaccatgtatccggagtacttcacaaaaacccagatttccttcgtgagaatgttcaggaatggacagatctaccgcggctaccatcccgtgatcatctgccccagatgcgagacaacaatagccttagcagagatcgaatacaggagggggaagacaaagctgaattatataaaattctccgaagacgttgttatagcaaccacccgtcctgaactgattcccgcatgcgttgccttggccgtgaacccttctgacaggagataccagcatttggtcggaagatctgtcagggttcccgttggaggacacgaagttaggatcatagcagatgaagcagtggaccccagtttcggtacgggaatagtgatggtgtgcacctttggtgaccgtcaggatgtgaggtggtggaagaagcacaacctcgagctcaggaacataataaaccgcaacggaaccctcaacgagcttgcaggtaagtacagcggtttgaagctttctgaggcgaggaacaggatacttgaggatttaaaggctgagggaagagttttgaagcaggaagaagttgatcacaatgtaggcgtttgctggcgctgcaagactccagttgagatctttccggcggagcagtggtttgtgaaggttgacaaggagaaggttatcgagatggctaagaaggtcaaatgggttccggaattcatgtttgacaggctcctcacctgggtagagtcaatggagtgggactgggtgataagcaggcagagagtttttgcaaccccgattccagtatggtactgcaagaagtgtggaaagataatggtggctgaagaggagtggcttccagttgatcctacgaagcagaagcctttgaggaattgtgaatgcggaagcagcgagtttgaaggtgagaaagacgtgctcgatacctggatggattcaagcataacaccattagccattgtaggatggcccgagacaaaagagtaccccgtctccctcaggccccaaggtcatgatataataagaacctgggcattctacacgatcctgcgatctgcagcccttatgaatgagattccgtggaaagagatcgttatcaacggtatcgtttttggcgaggatggcaggaagatgagcaagagcatcggcaatgttattagcccggaagaggttgtggagcaatacggcgccgatgcattgcgccagtgggcagcgagcggtgtgatcggcgaagatctgattttcagctggagagaagtagttgcagcaagcagattccagcaaaaactctggagcgttgcgagattcgttctttctcatatttccggatatgaagagagagatgaagacgttaaaagccttagaactgcagataggtggataatttcgaaactgaacaggcttatagccacggttcgcgaaaacatggacaattacagatttgatgaagccctgaaggcgataaggggctttgtgtggtatgaacttgccgacaactacatagagctcgtgaagggcaggctttattcaggcaaagaatcgaattcagccaagtttacgctcaaatacgtgctcgatagagttctgcgtctccttgcccccattactccttttataaccgaagaaatatggagcaggtttggaaaaggcagcattcatctgcagaagtatcccgaagtagaagggcggttcatagatgaagaggttgaaagggctggagaagagatgaaggagattctttctgcgatcagaaagctgaagcacgataaaggactggctctgaatgctccgctccagaagatcatcgttttcactcagctcgaacttgatacaatggatctcgaatttgccacgaattccaaggtggagttgactaaaaatctcccgaaaataaaggaagaggttagaaggctgaagccgaagttcgcgatactcgggcctctgtttaaggaccgggtgaaagacttattgagagcagttgaagggctgagcg

>Bin11_76

catctcaactggattcacgacgccagcagagggaatgaaaaccccgccataaactttttcttttatatttggttccatctctaaaagcttcttcctgctcaaaggcttcgccgtaacctcgtggaatctcagatatagcactattagtggaatcgcaagaaaagttaatggattcgttgccactgtaatcagtccaacgcgcctgaattcgaagccgagctcctcggcttcagcatccatcattctgtttccctcaaggcagagcttactccttaaaagcttgaagggaagctggaatggatgaatgatgcctgagcaggactttgtttgctctaagccagggccgagctttctctcaaaaaccacgacgcttagatcgaactttgaaagctctttggctatgaagctacccgtgacccccgccccgactatcgcaacatccttcaaattcccaccacccccaaagtcttcgaagaatatttattcttttctaataaattctgatataggtgatccgatgagctacgttctcggcatagatgctggcaccactggtattagggcaggcgtttactctcttgaaggagaacttgtagctcagagctacacggagtttccgagctactatccgcatcccggatgggttgaacaaaatgctgaggactggtggaagagtgtcgttaatgcctgcaattctgctgtaaagctcgcaaaagctgaagaggatatttttgccatatctgttacgaatcagagggaaaccatagtgcctgtctcgaaaaccgggtttccgctggcaagggcaatagtctggcaggacagaagaacagtcgaggaggtgagaaggataaagtcgataatgggagaagaggtgttcaggatcacgggcttgaagccagatccgtatttctcactcccaaagattctgtggtggatgaaaaattatcccgagatgattgagaaaacatggaagttcatgctcgttcatgactacatcgtttacagaatgactggaaatgtcgtcacagacttttctaacgcttccaggacaatgctaatggatctaaacaagagaaagtggtcagataaaattgcagagacctttggagttgatctggaaaagtttccagaaatcagaaattcaggcgaagttgccggagagctcaggaacagagagatcgagataagatcgagaccagtcgttgcagtaggtgggggagaccagcagtgcagcgctcttgggcagggagtcgttgaagaggggaagataaagtcaacaaccggcacaggaacattcatgattgctcccgtggagaaaatgcatggtggtgagattatttattccgcccacgttgtgccgaagatcgtcgcagaggtgagcattttcacaacgggaagtcttctcgattgggtaaagaggaatttctttcagaatgaaagctacgaagttcttaatgccgaggcaaaggcttcaggcgttggagcgagaggtttgatggtatttccattcttttctggtgcaggatgcccgcactggaatccagaggccaagggcacgatttacggtctcaccttgggccattcgaggggagacatagccagagcggttatggagagtgtggcctttgaagtgaaaacgaacatagacctaatggagagcctgggaataaaaataaacgagctacgtctcgacggaggtgcagcaagaagcgcactgtggaatcagatatttgcagacgtcataaaaaagccttgtttgatttcagaagacgttgaagccactgcaagaggggctgcaatgcttgcatgtctctcaattggattctcgttaaaatacgttcttgaaaagtttgtccctcgcttcaaaaccgtaataccttcgggcatagactattcagaaatctatgagagatacaaaaaaattagagagattattctaagcgcagcgggaagctaagtgacctttatttccactctgttctcccagactccatgcaggttgcagtaagctaaagcatacaagactccgcttttgctcaacttgagcttgatgcttgcttcctgttctgaaatttcaggtgtcagctcaactctcgctatgaacactggattaaattctctgccctcctcgtagaagaacagttcaatccatctgaaagagtgctgaactgtatttggatgcccttgtatgtaaactctaacgttaaaagcttccccagcctttacttcctttggtgcctcaatccttggcgtgtgattttctctcttcgaaatggcaagtctgtcctctacctcaggagtatagataacttctccaaacttcctcattttctccatatttctgatttttatctaagcaaataagaaactttcggagattttcagggatcataaattcaaaaacggaagtttattacagacttttatttcataaaatgcgaaaaaacttgtcaataacagattacatcatcataatttcgagaaaatttcaaaaacgtaaaatattcatcgacaacttcagatatgcttctgctcgaacacgaggctaaggagctcctcgagagctacggaatcaggactgcaaggggagtgatctgcgagagcgag

>Bin11_77
[truncated: 4,046,148 more chars]
